# Practical carbon–carbon bond formation from olefins through nickel-catalyzed reductive olefin hydrocarbonation

**DOI:** 10.1038/ncomms11129

**Published:** 2016-04-01

**Authors:** Xi Lu, Bin Xiao, Zhenqi Zhang, Tianjun Gong, Wei Su, Jun Yi, Yao Fu, Lei Liu

**Affiliations:** 1 Hefei National Laboratory for Physical Sciences at the Microscale, iChEM, CAS Key Laboratory of Urban Pollutant Conversion, Anhui Province Key Laboratory of Biomass Clean Energy, University of Science and Technology of China, Hefei 230026, China; 2 Department of Chemistry, Tsinghua University, Beijing 100084, China

## Abstract

New carbon–carbon bond formation reactions expand our horizon of retrosynthetic analysis for the synthesis of complex organic molecules. Although many methods are now available for the formation of C(*sp*^2^)–C(*sp*^3^) and C(*sp*^3^)–C(*sp*^3^) bonds via transition metal-catalyzed cross-coupling of alkyl organometallic reagents, direct use of readily available olefins in a formal fashion of hydrocarbonation to make C(*sp*^2^)–C(*sp*^3^) and C(*sp*^3^)–C(*sp*^3^) bonds remains to be developed. Here we report the discovery of a general process for the intermolecular reductive coupling of unactivated olefins with alkyl or aryl electrophiles under the promotion of a simple nickel catalyst system. This new reaction presents a conceptually unique and practical strategy for the construction of C(*sp*^2^)–C(*sp*^3^) and C(*sp*^3^)–C(*sp*^3^) bonds without using any organometallic reagent. The reductive olefin hydrocarbonation also exhibits excellent compatibility with varieties of synthetically important functional groups and therefore, provides a straightforward approach for modification of complex organic molecules containing olefin groups.

## Abstract (web summary)

Olefins are employed in many coupling procedures but direct hydrocarbonations of unactivated olefins remain to be developed. Here, the authors report the nickel-catalyzed reductive coupling of olefins with aryl and alky electrophiles under mild conditions and with a broad substrate scope.

## Graphical abstract

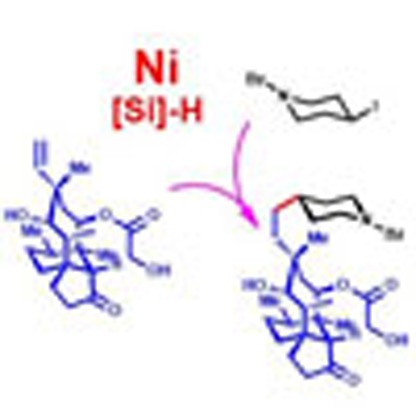

Olefins are important synthons in organic chemistry^1^,^2^. They are readily available as stable and inexpensive compounds with great diversity. Simple olefins are both raw materials and products in petrochemical industry. For example, ethylene is produced mostly through steam cracking. They are converted to higher olefins, polyethylene materials and various commodity chemicals^3^. On the other hand, olefin groups are also widely represented in natural products with complex structures and many functional groups. Not only the extensive source but also the unique chemical reactivity of olefins attracts chemists, as the olefin moieties are resistant to a good number of synthetic transformations. Some unique transition metal catalyst systems can activate the olefin double bonds leading to highly elegant as well as useful reactions. Famous examples include the Wacker process^4^, olefin metathesis^1^,^5^, olefin hydroformylation^6^ and Heck reaction^7^ that have been extensively used in the preparation of complex organic molecules both in laboratory and in industry. These reactions establish the central role of olefins in modern synthetic organic chemistry as well as fine chemical industry^8^.

More recently, unactivated olefins have been used directly as chemical input in some novel cross-coupling reactions (for example, carbon-heteroatom coupling reactions^9^,^10^,^11^,^12^,^13^,^14^,^15^ and few examples of carbon–carbon coupling reactions^16^,^17^,^18^,^19^,^20^). These findings suggest that olefins can be recognized as nuclephilic radical equivalents^9^,^10^,^12^,^15^,^17^,^20^ or alkylmetallics equivalents^11^,^13^,^14^,^18^,^19^,^21^,^22^,^23^ from a novel perspective. In some of these emerging methods that involve transition metal catalysts (Cu (ref. 22), Fe (refs 9, 20), Co (ref. 15), Mn (ref. 24) and so on), silanes were used as hydride source as well as reductant. New reactions that use olefins as chemical input are expected to bring new opportunities to organic synthesis. For instance, use of olefins to replace alkylmetallic reagents in traditional cross-coupling reaction fashion^25^ (for example, Kumada coupling reaction) with aryl/alkyl electrophiles would have appealing advantages such as better functional group compatibility and broader substrate availability.

We now report the discovery of a new catalytic reaction of olefins, namely, Ni-catalyzed intermolecular reductive olefin hydrocarbonation between olefins and alkyl/aryl halides in an anti-Markovnikov fashion. This reaction provides an efficient strategy for the construction of carbon–carbon bonds^26^,^27^ from more stable and less expensive substrates as compared with the existing methods using organometallic reagents^25^,^28^,^29^,^30^. In terms of practicality, the reaction shows high levels of ‘chemo'- and ‘regio'-selectivity, so that a wide range of sensitive functional groups can be tolerated (for example, epoxide, aldehyde and alcohol) in the transformation with minimal substrate protection necessary^31^. As Ni-catalyzed carbon–carbon bond formation processes have enjoyed great success in modern synthesis^32^, the present reaction is expected to find important applications in organic chemistry.

## Results

### Reaction discovery

We screened various Ni catalysts, base, silane and solvents for the reductive olefin hydrocarbonation reaction of 1-octene with **1** (see Table 1 and Supplementary Tables 1–5). The pincer complex **L1** was tested first, but only trace amount of desired product was obtained with large amount of alkyl iodide recovered. We then tested the terpyridine ligand **L2** and pybox ligand **L3**. Higher conversion of alkyl iodide was observed but the yield was only slightly improved. To our delight, we observed significant formation of the desired product with the phenanthroline family ligands **L4** and **L5**. We then tested **L6** bearing an *ortho*-methyl group but **L6** was inferior. On the other hand, a bipyridine ligand **L7** exhibited much better reactivity. Remarkably, when 4,4′-di-*tert*-butyl-2,2′-bipyridine (**L8**) was used, the GC yield increased to 96% with an isolated yield of 93% for the desired product. We also tested some monodentate phosphine ligands (**L9** & **L10**) and carbene ligand (**L11**), but they were not effective.

### Substrates scope

The substrate scope of the reductive olefin hydrocarbonation reaction was shown in Table 2. A variety of carbon electrophiles and olefins with different functional groups could be readily converted to the desired products with modest to excellent yields (30–93%). Not only alkyl iodides (for example, **3**), bromides (for example, **13**) and tosylates (for example, **30**) were good substrates, but also aryl iodides^33^,^34^ (for example, **28**) could be transformed successfully. With respect to olefins, both mono-substituted (for example, **26**) and 1,1-di-substituted alkenes (for example, **31**) could be used. Because of the mild reaction conditions, a wide range of synthetically relevant functional groups could survive the transformation. For instance, ether (**4–6**), ester (**7**), fluoride (**8**), trifluoromethyl (**9**), carbamate (**10–11**), sulfonamide (**12–13**), amine (**14**), aryl choride (**15**) and bromide (**16**) were well tolerated. Heterocycles such as thiophene (**17**), furan (**18**), and pyridine (**19**) could also be used in the reaction. Several base-sensitive groups, such as nitrile (**20**) and ketone (**29**, **32**) posed no problem. Even more active groups, such as unprotected benzaldehyde (**21**) and azo groups (**22**), were compatible with the reaction. As an interesting substrate, **23** containing a pinacol boronate ester^35^ could selectively undergo the reductive olefin hydrocarbonation reaction with its carbon–boron bond intact. To our surprise, the reaction could even be conducted in the presence of an epoxide group^36^ (**24**) or an unprotected OH group (**25**). Noteworthily, an internal alkene^11^,^14^ (for example, **33**) could have been converted in the reaction, although further ligand optimization was needed to improve the yields.

### Modification of complex molecules

**Figure 1:**
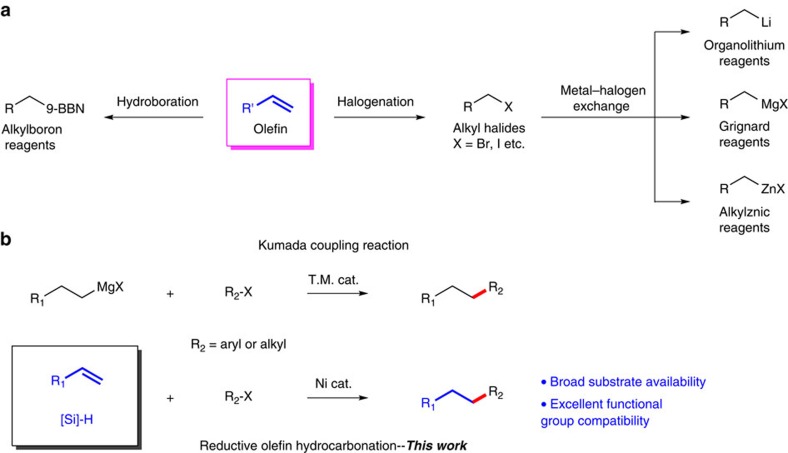
Carbon–carbon bonds formation from olefins. (**a**) Alkyl organometallic reagents used in cross-coupling reactions. Alkylboron reagents^47^,^48^,^49^ are usually made through alkene hydroboration. Grignard^50^,^51^, organolithium^52^,^53^ and alkylznic reagents^42^,^54^ are generally obtained through insertion of metals into alkyl halides. However, an often ignored problem is that most terminal alkyl halides are converted from olefins^55^. (**b**) Comparison of reductive olefin hydrocarbonation reaction with transition metal-catalyzed Kumada-coupling reaction. From a viewpoint of synthetic chemistry, the combination of olefins with silanes could be recognized as equivalent to alkyl organometallic reagents. 9-BBN=9-borabicyclo[3.3.l]nonane.

**Figure 2:**
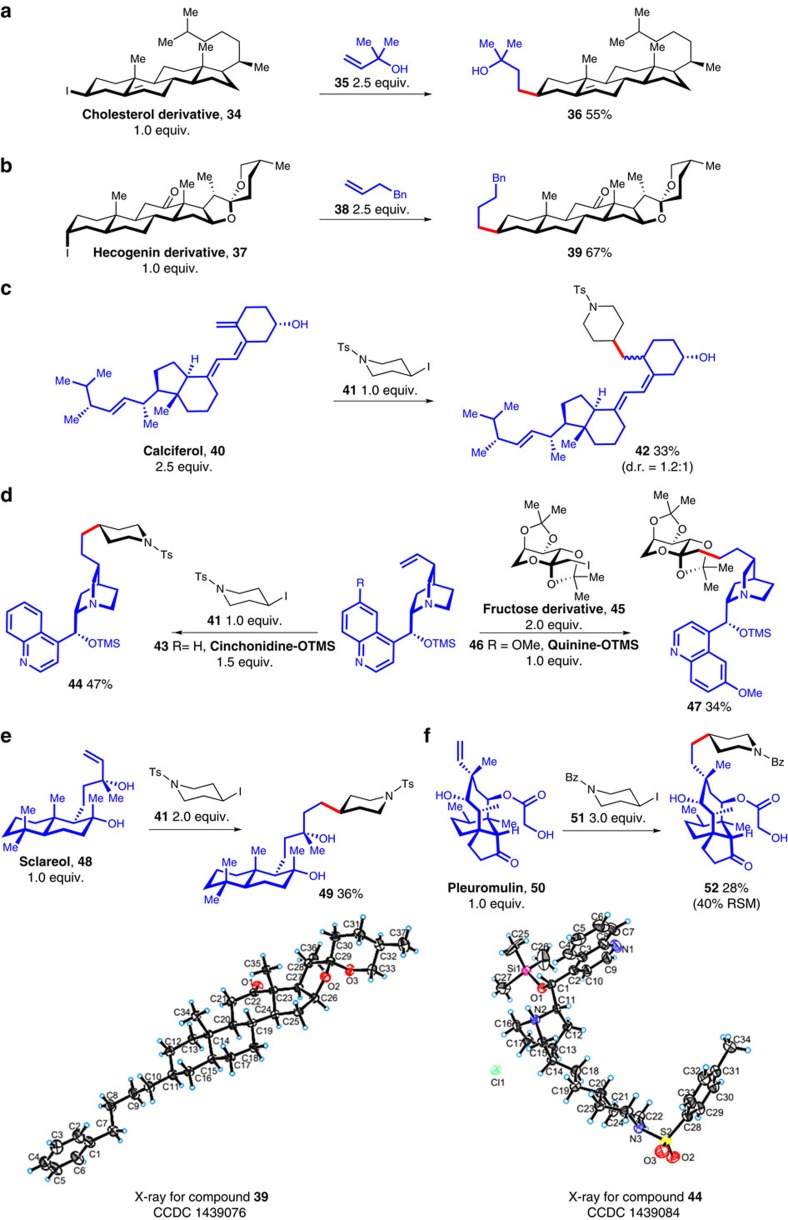
Modification of complex molecules. (**a**) 10% NiBr_2_.diglyme, 15% **L8**, 3.0 equiv. DEMS, 3.0 equiv. Na_2_CO_3_, 2 ml DMAc, 30 °C, 12 h. (**b**) The same conditions as in **a** the newly formed carbon–carbon bond was between C10 and C11. (**c**) 20% NiBr_2_.diglyme, 30% **L8**, 3.0 equiv. DEMS, 3.0 equiv. Na_2_CO_3_, 2 ml DMAc, 30 °C, 12 h. (**d**) conditions for compound **44**: 20% NiBr_2_.diglyme, 30% **L8**, 2.0 equiv. DEMS, 2.0 equiv. Na_2_CO_3_, 2 ml THF/DMAc (v/v=1/3), 30 °C, 12 h, the newly formed carbon–carbon bond was between C19 and C20; conditions for compound **47**: same conditions as in **c**. (**e**) Same conditions as in **c**. (**f**) 20% NiBr_2_.diglyme, 30% **L8**, 4.0 equiv. DEMS, 4.0 equiv. Na_2_CO_3_, 2 ml DMAc, 30 °C, 12 h. Bn, benzyl; Bz, benzoyl; TMS, trimethylsilyl.

To further demonstrate the high degree of functional group compatibility of the reductive olefin hydrocarbonation reaction (Fig. 1), we exploited its use as a novel tool for the modification of complex biologically interesting molecules (Fig. 2). As an example, a cholesterol derivative (**34**) could react with **35** to produce **36** without affecting either the internal alkene or alcohol groups (Fig. 2a). Hecogenin derivative (**37**), which contained both ketal and ketone groups, was also a good substrate for the modification process (Fig. 2b). Furthermore, calciferol (**40**) was converted to **42** selectively in the presence of the hydroxyl, internal alkene and even 1,3-diene groups (Fig. 2c).

Modification of a cinchonidine derivative (**43**) resulted in the ‘chemo'-selective formation of **44**, while tolerating both the amino group and quinoline structure (Fig. 2d, left). Single-crystal XRD analysis of **44** confirmed that the skeleton of cinchonidine was fully maintained during the modification process. In addition, the coupling of quinine (**46**) and a fructose derivative (**45**) enabled the production of highly complex molecules in a convergent fashion (Fig. 2d, right).

The reductive olefin hydrocarbonation reaction of sclareol (**48**) proceeded smoothly in the presence of different two tertiary alcohol groups (Fig. 2e). In a more complex example with pleuromulin (**50**) (Fig. 2f), we obtained the desired product (**52**) in 28% yield (with 40% recovery of starting material) despite the presence of carbamate, ester, keton, unprotected primary and secondary alcohol groups in the reactant. Therefore, the reductive olefin hydrocarbonation reaction presents attractive opportunities for the modification of natural products or other complex molecules.

### Other applications

**Figure 3:**
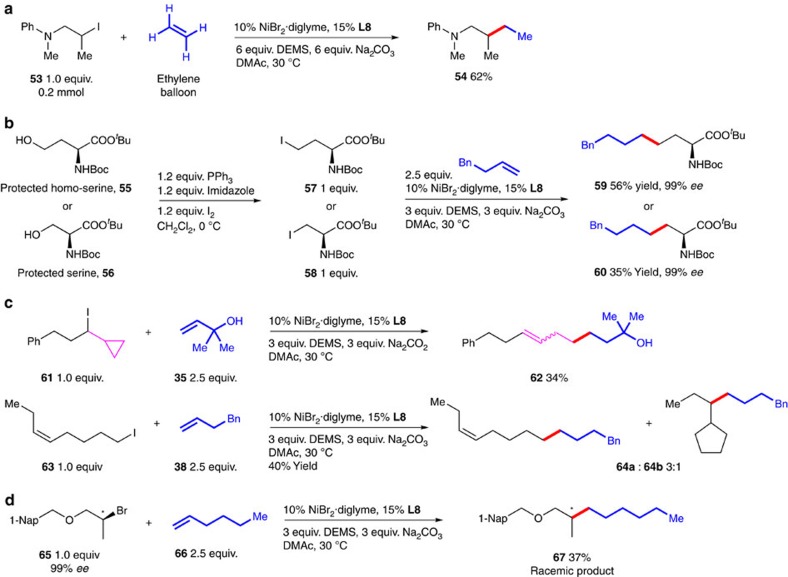
Other applications of reductive olefin hydrocarbonation reaction. (**a**) Conversion of ethylene. (**b**) Synthesis of non-natural amino acids. (**c**) Radical clock experiments. (**d**) Stereochemistry of reductive olefin hydrocarbonation reaction. Nap, naphthyl.

Ethylene, as the simplest and most abundant olefin, has been attracting increasing attentions in synthetic organic chemistry^3^. We were delighted that ethylene as C2 source was indeed a good substrate in the reductive olefin hydrocarbonation reaction (Fig. 3a). Compound **54** was obtained in 62% isolated yield.

The reductive olefin hydrocarbonation reaction was useful for the synthesis of non-natural amino acids^37^ (Fig. 3b). As an example, homoserine-derived iodide **57** could be converted to **59** with a yield of 56%. More interestingly, the reaction of a racemization-prone serine derived iodide **58** was also successful affording **60** in 99% *ee*. This finding was surprising because in our previous study^38^ on the Ni-catalyzed reaction of **58** we observed significant racemization of the amino acid.

To gain more insights into the reaction mechanism, radical clock experiments were carried out (Fig. 3c). Compound **61** containing a cyclopropyl ring was used as radical clock substrate (Fig. 3c, top). In this coupling reaction, we obtained only the ring-opened product **62** in 34% isolated yield.^39^ We also tested the reaction with (*Z*)-8-Iodooct-3-ene (**63**) (Fig. 3c, bottom). A mixture of linear coupling product (**64a**) and ring-cyclized product (**64b**) was obtained with a ratio of 3:1. The formation of ring-cyclized product (**64b**) revealed that this reaction proceeds through a radical cyclization process^40^.

Finally, we took advantage of optical pure secondary alkyl bromide (**65**) to study the stereochemistry of this reductive olefin hydrocarbonation reaction (Fig. 3d). When (*S*)-**65** was alkylated with 1-hexene, we obtained a racemic product (**67**) in 37% yield^41^. Furthermore, radical inhibiting experiment using TEMPO (2,2,6,6-tetramethylpiperidinooxy) as a radical trap was carried out (see Supplementary Discussion). The reaction was largely inhibited when 0.2 equiv. TEMPO was added, indicating a radical type reaction mechanism^32^. Nonetheless, details for the mechanism of this reaction are not clear at present^42^,^43^,^44^,^45^,^46^. Further investigations are ongoing in our lab.

In summary, we have developed a practical and user-friendly method for the formation of carbon–carbon bonds through Ni-catalyzed intermolecular coupling of aryl or alkyl electrophiles with olefins under reductive conditions. This newly developed reductive olefin hydrocarbonation reaction provides a useful and general approach for the construction of carbon–carbon bonds by directly using olefins as nucleophile precursors. This reaction exhibited excellent compatibility with varieties of synthetically important functional groups and therefore, provided an efficient new approach for the modification of complex molecules. Our next challenge was the development of asymmetric version of this new carbon–carbon bond forming reaction and its extension to internal olefins.

## Methods

### Materials

For NMR and high-performance liquid chromatography spectra of compounds in this manuscript, see Supplementary Figs 1–119. For details of the synthetic procedures, see Supplementary Methods. For X-ray data see Supplementary Data 1.

### Procedure

NiBr_2_·diglyme (7.0 mg, 0.02 mmol, 10 mol%), 4,4′-di-*tert*-butyl-2,2′-bipyridine (8.0 mg, 0.03 mmol, 15 mol%) and Na_2_CO_3_ (42.4 mg, 0.4 mmol, 2.0 equiv.) were added to a Schlenk tube equipped with a stir bar. The vessel was evacuated and filled with argon (three cycles). To these solids, 0.6 ml DMAc was added under argon atmosphere. The reaction mixture was stirred at room temperature for 30 s. To the reaction mixture, electrophile (0.2 mmol, 1.0 equiv.), alkene (0.3 mmol, 1.5 equiv.) and DEMS (0.4 mmol, 2.0 equiv.) were added under a positive flow of argon. The reaction mixture was stirred at 30 °C for 12 h. To remove the DMAc, the reaction mixture was poured into 50 ml of ice water and the resulting mixture was extracted with ethyl acetate (4 × 30 ml). The combined organic layer was dried over Na_2_SO_4_, filtered, concentrated in vacuum and purified by column chromatography on silica gel.

## Additional information

**Accession codes:** The X-ray crystallographic structures for 39 and 44 reported in this article have been deposited at the Cambridge Crystallographic Data Centre (CCDC), under deposition number CCDC 1439076 and 1439084, see Supplementary Data 1, the data can be obtained free of charge from the Cambridge Crystallographic Data Centre via http://www.ccdc.cam.ac.uk/ data_request/cif.

**How to cite this article:** Lu, X. *et al*. Practical carbon-carbon bond formation from olefins through nickel-catalyzed reductive olefin hydrocarbonation. *Nat. Commun.* 7:11129 doi: 10.1038/ncomms11129 (2016).

## Supplementary Material

Supplementary Information Supplementary Figures 1-119, Supplementary Tables 1-5, Supplementary Discussion, Supplementary Methods and Supplementary References

Supplementary Data 1 Data of X-ray crystallographic structures for compound 39 and 44.

## Figures and Tables

**Table 1:**
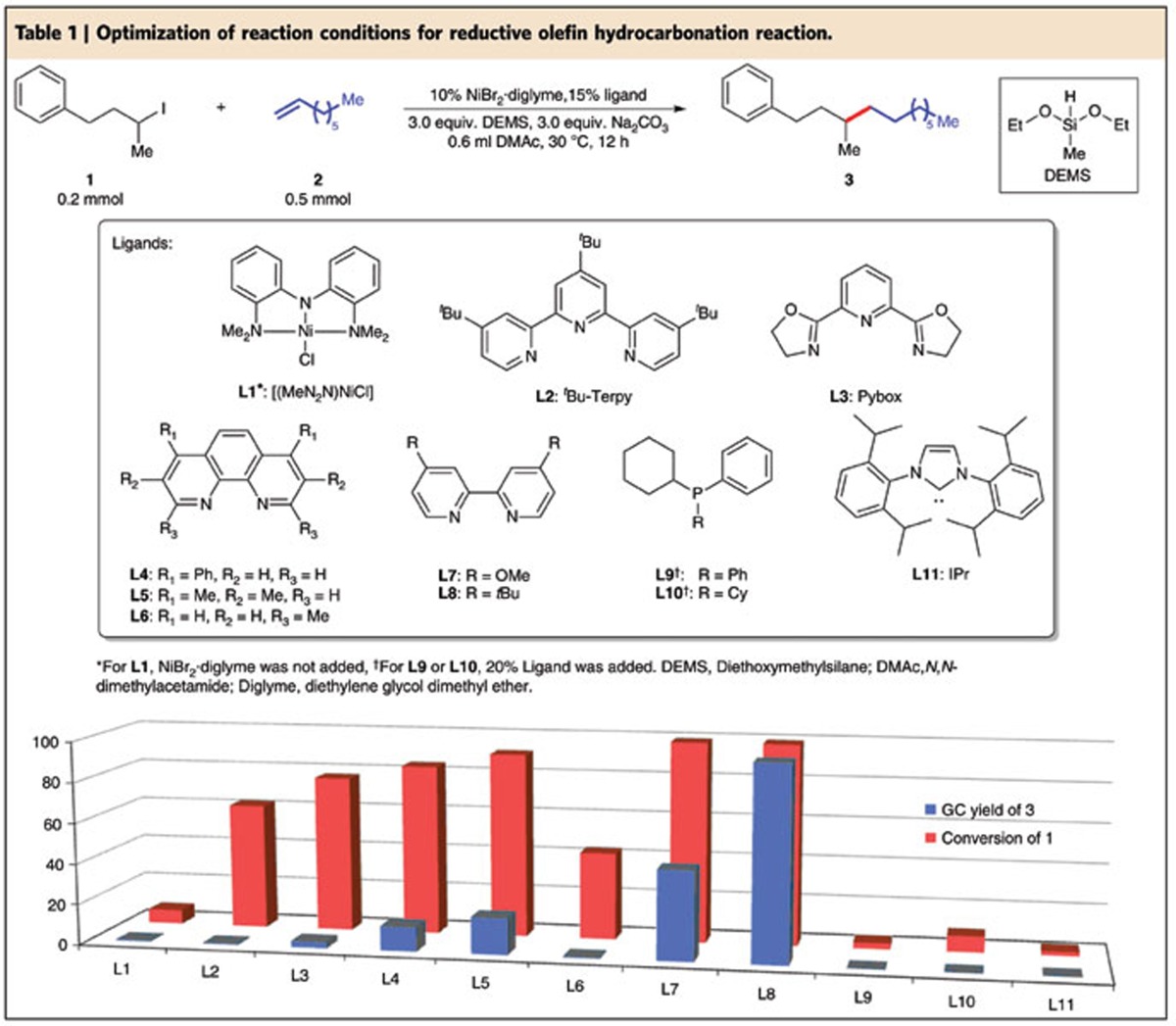
Optimization of reaction conditions for reductive olefin hydrocarbonation reaction.

**Table 2:**
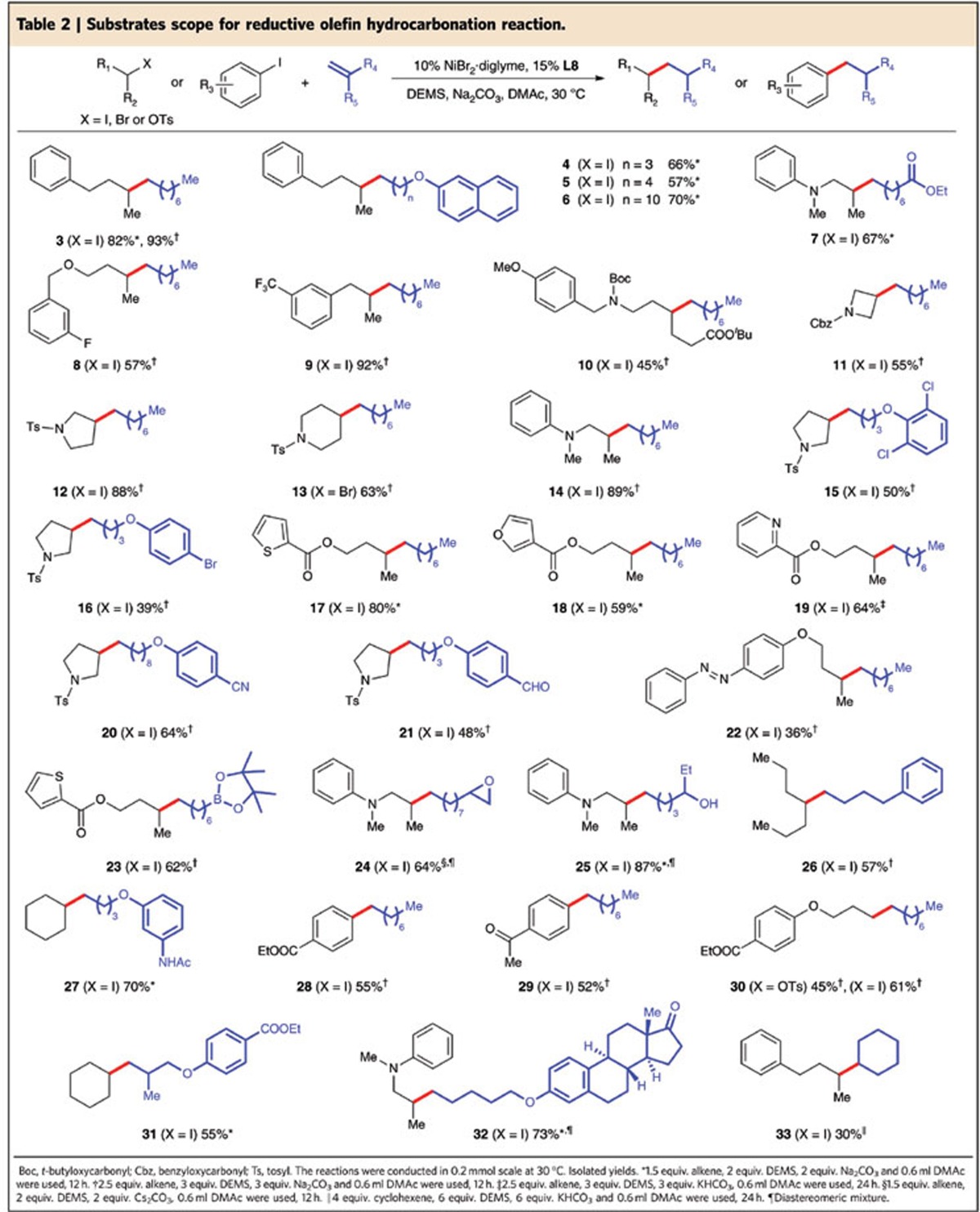
Substrates scope for reductive olefin hydrocarbonation reaction.
