# Supplementary material for: Practical carbon–carbon bond formation from olefins through nickel-catalyzed reductive olefin hydrocarbonation
Source: Nat Commun. 2016 Apr 1;7:11129. doi: 10.1038/ncomms11129 (PMC4821992; doi:10.1038/ncomms11129)
Supplement: Supplementary Information — Supplementary Figures 1-119, Supplementary Tables 1-5, Supplementary Discussion, Supplementary Methods and Supplementary References [file ncomms11129-s1.pdf]

Supplementary Figures

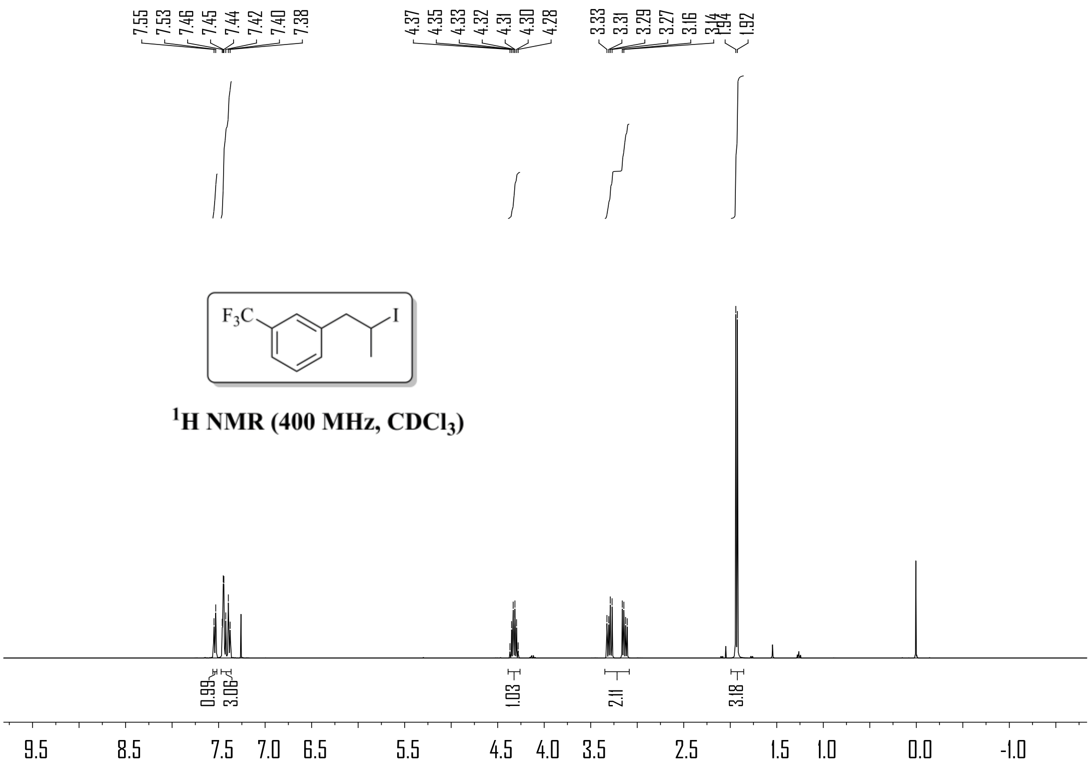

Supplementary Figure 1. ^{1}H NMR spectra for 1-(2-iodopropyl)-3-(trifluoromethyl)benzene

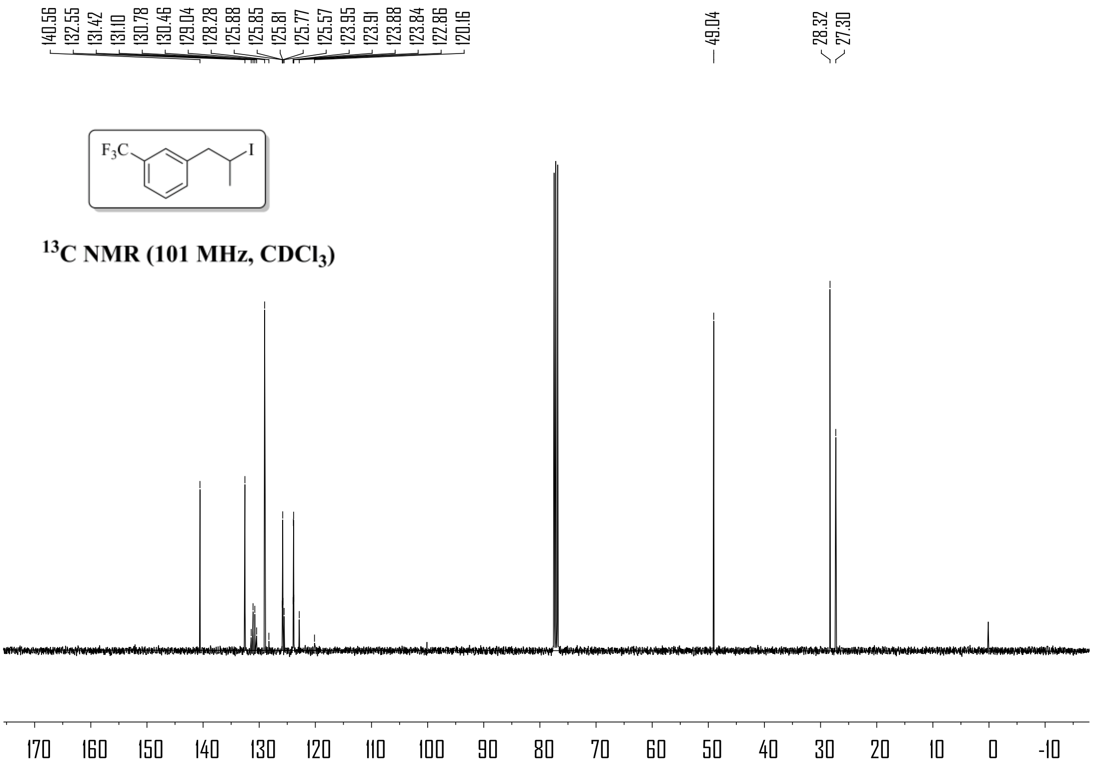

Supplementary Figure 2. ^{13}C NMR spectra for 1-(2-iodopropyl)-3-(trifluoromethyl)benzene

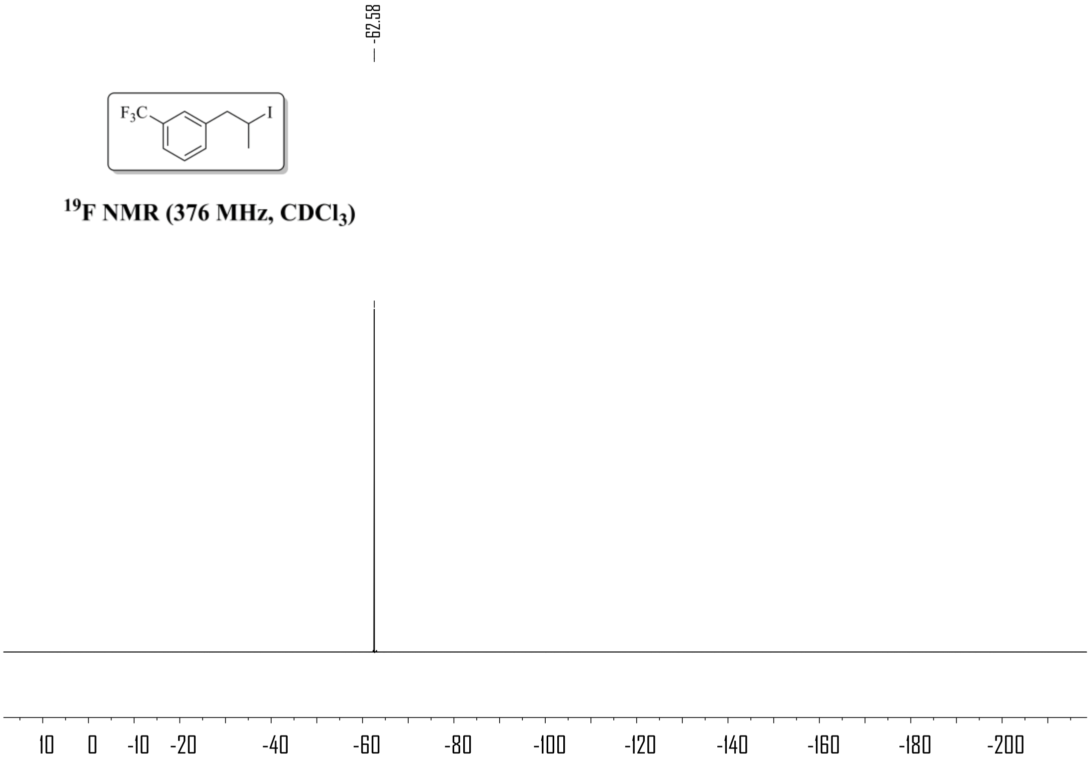

Supplementary Figure 3. ^{19}F NMR spectra for 1-(2-iodopropyl)-3-(trifluoromethyl)benzene

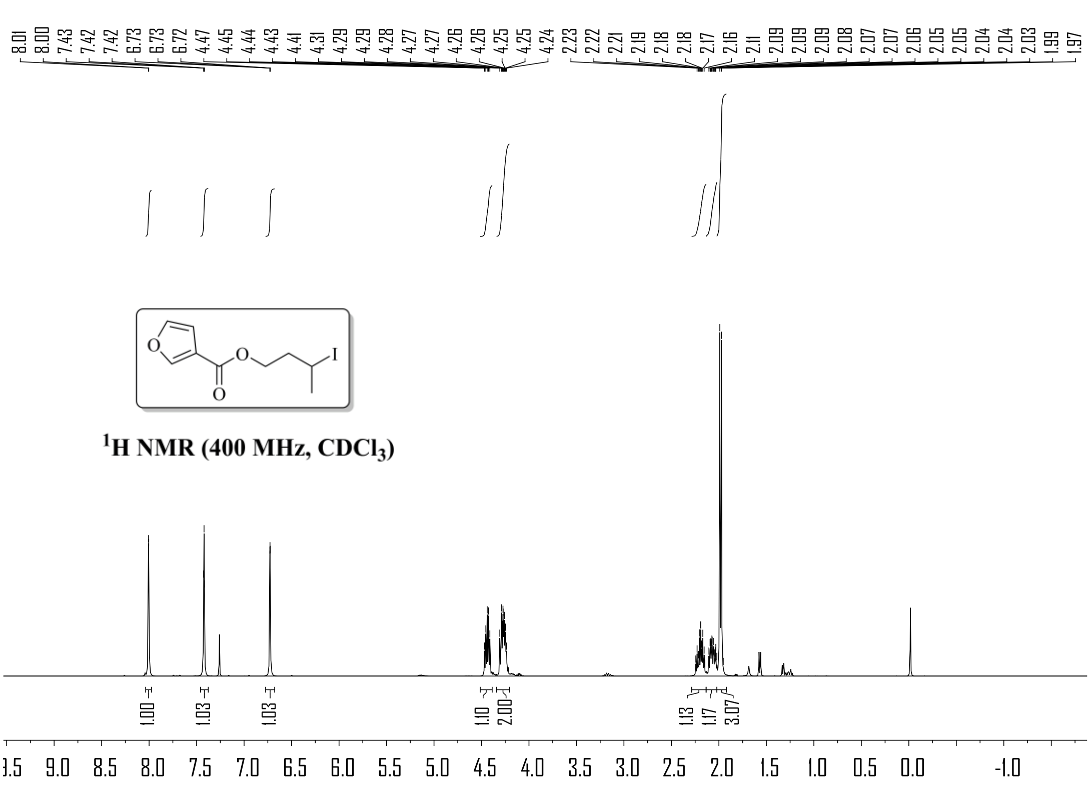

Supplementary Figure 4. ^{1}H NMR spectra for 3-iodobutyl furan-3-carboxylate

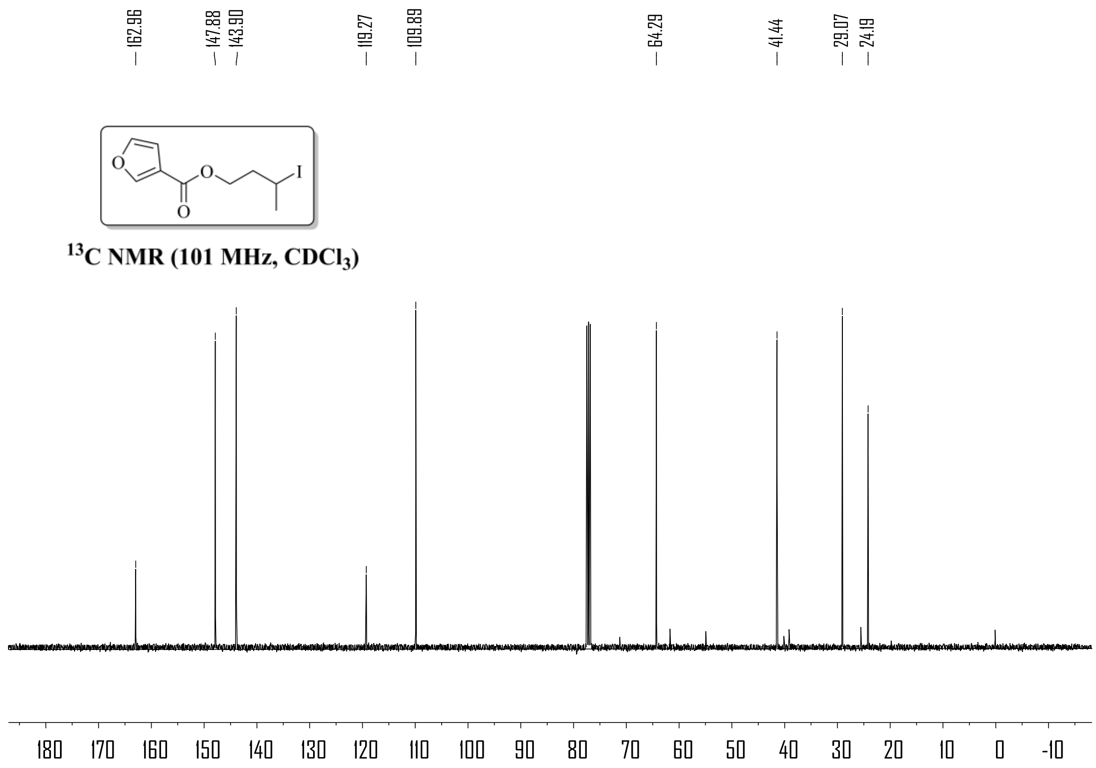

Supplementary Figure 5. ^{13}C NMR spectra for 3-iodobutyl furan-3-carboxylate

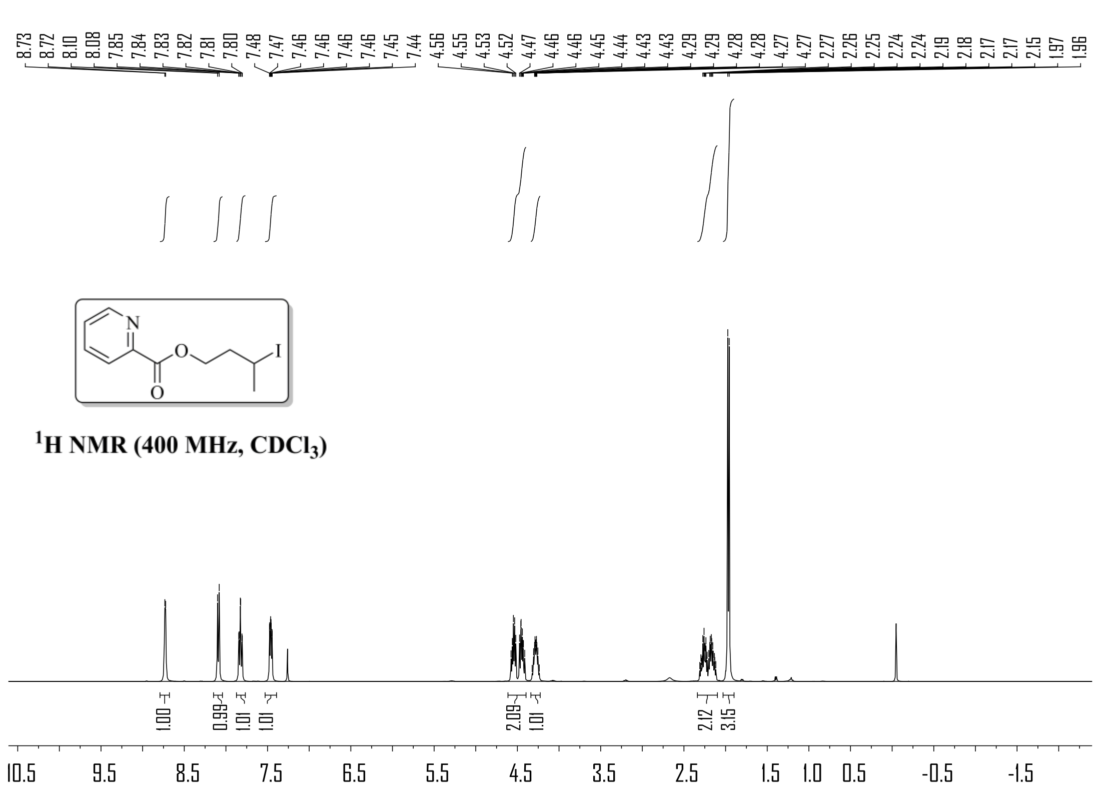

Supplementary Figure 6. ^{1}H NMR spectra for 3-iodobutyl picolinate

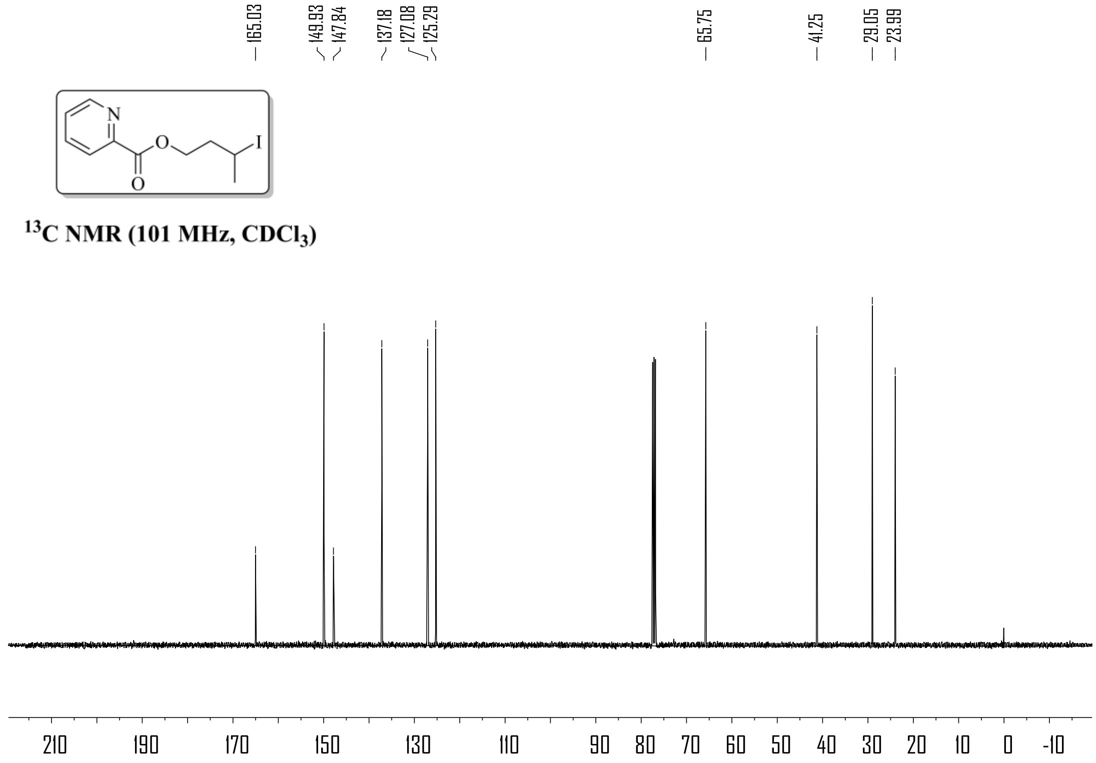

Supplementary Figure 7. ^{13}C NMR spectra for 3-iodobutyl picolinate

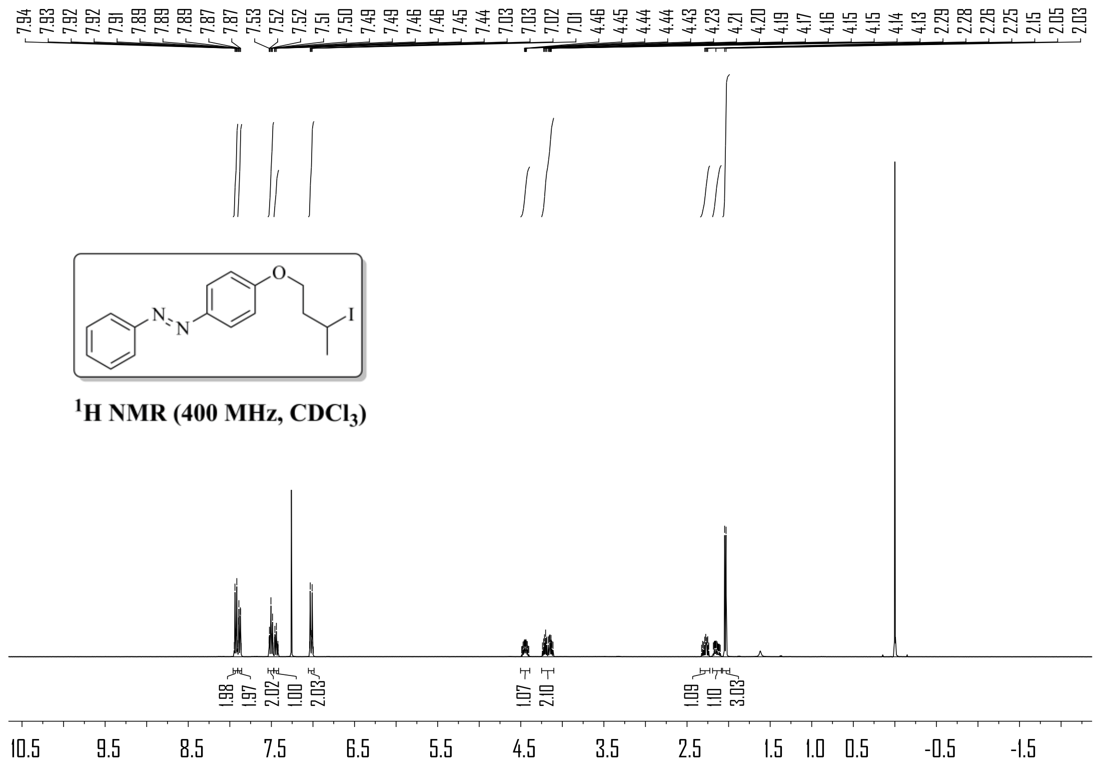

Supplementary Figure 8. ^{1}H NMR spectra for (*E*)-1-(4-(3-iodobutoxy)phenyl)-2-phenyldiazene

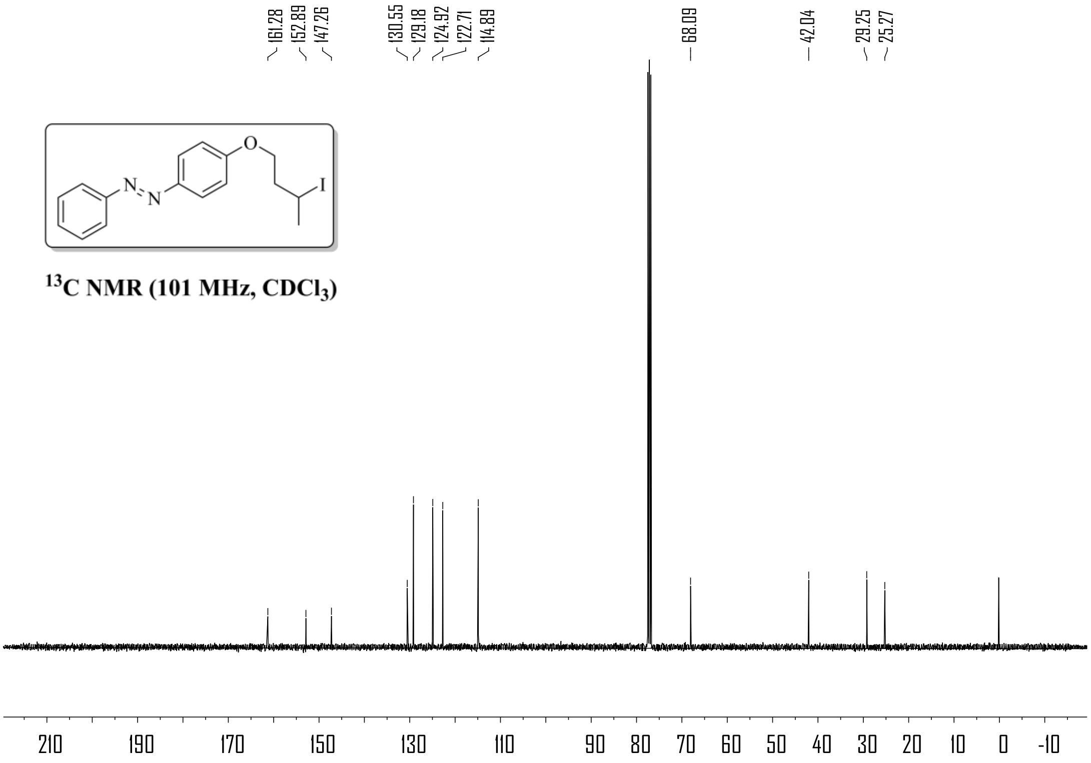

Supplementary Figure 9. ^{13}**C NMR spectra for (*E*)-1-(4-(3-iodobutoxy)phenyl)-2-phenyldiazene**

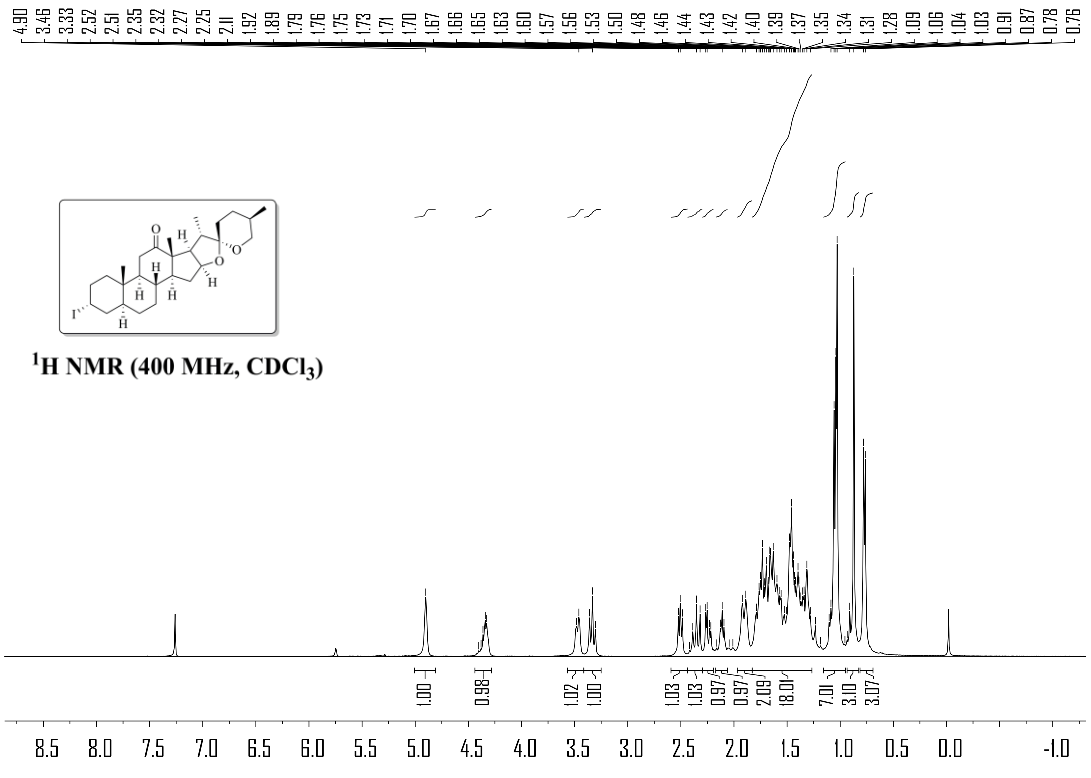

Supplementary Figure 10. ^{1}**H NMR spectra for (2a*S*,4*R*,5'*R*,6a*S*,6b*S*,8a*S*,8b*R*,9*S*,10*R*,11a*S*,12a*S*,12b*R*)-4-iodo-5',6a,8a,9-tetramethylicosahydrospiro[naphtho[2',1':4,5]indeno[2,1-*b*]furan-10,2'-pyran]-8(2*H*)-one**

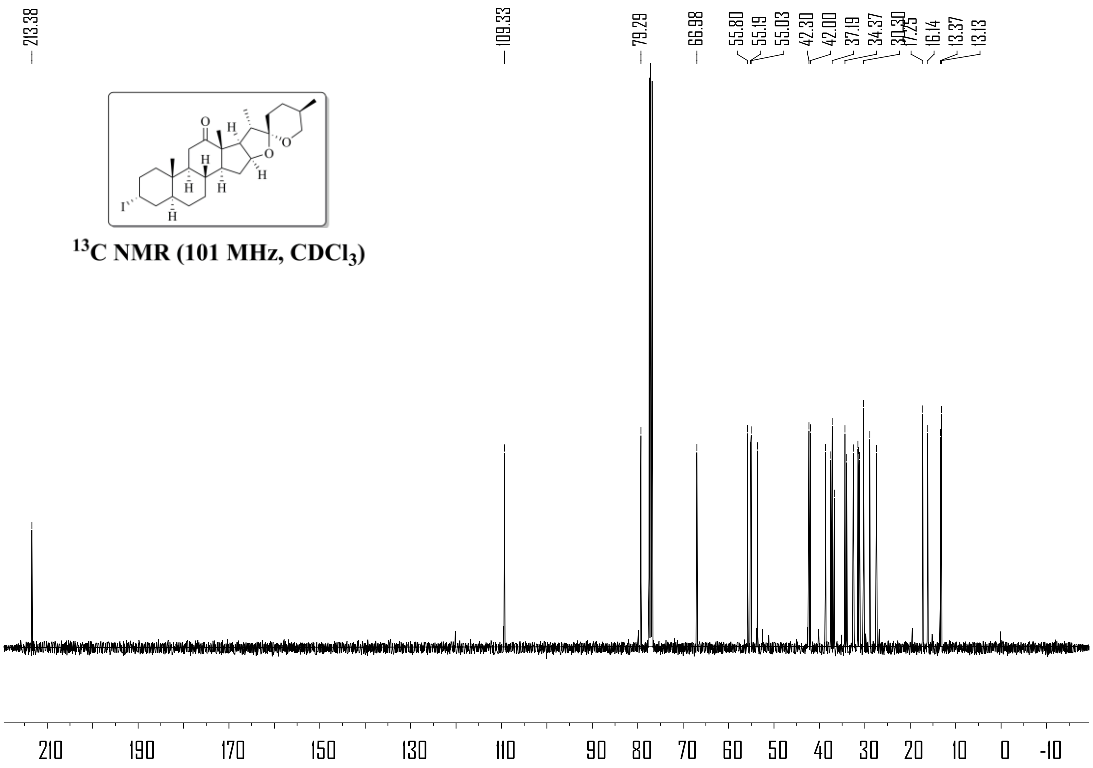

Supplementary Figure 11. ^{13}C NMR spectra for (2a*S*,4*R*,5'*R*,6a*S*,6b*S*,8a*S*,8b*R*,9*S*,10*R*,11a*S*,12a*S*,12b*R*)-4-iodo-5',6a,8a,9-tetramethylicosahydrospiro[naphtho[2',1':4,5]indeno[2,1-*b*]furan-10,2'-pyran]-8(2*H*)-one

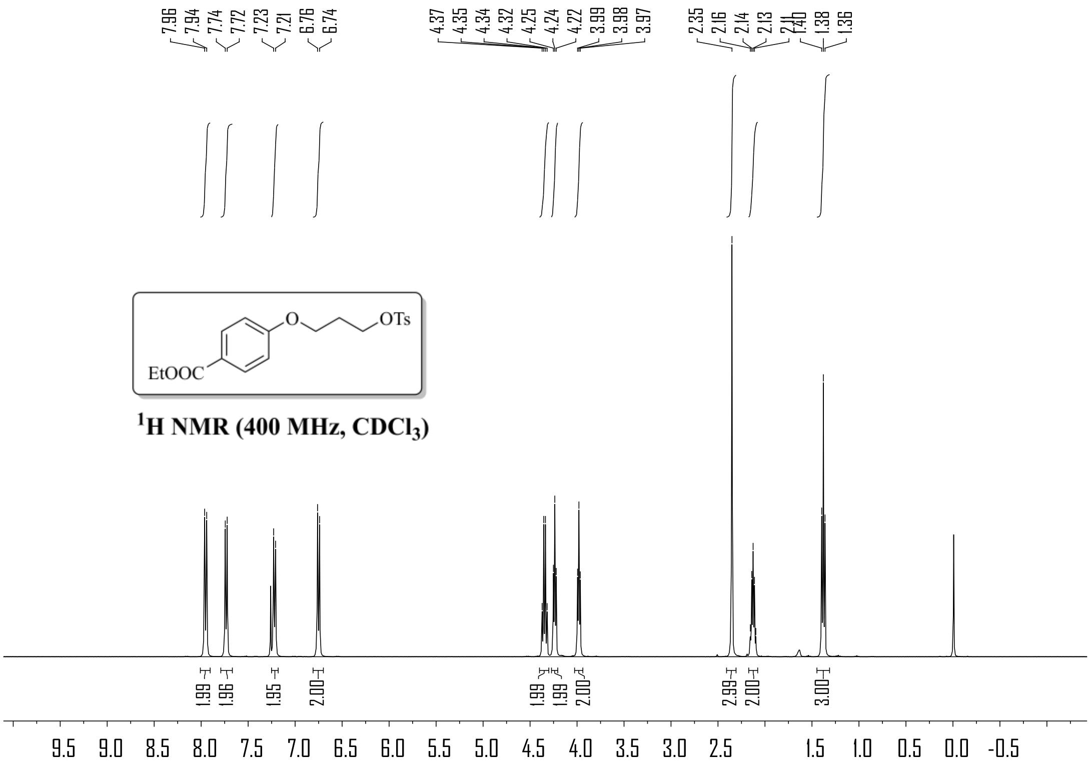

Supplementary Figure 12. ^{1}H NMR spectra for ethyl 4-(3-(tosyloxy)propoxy)benzoate

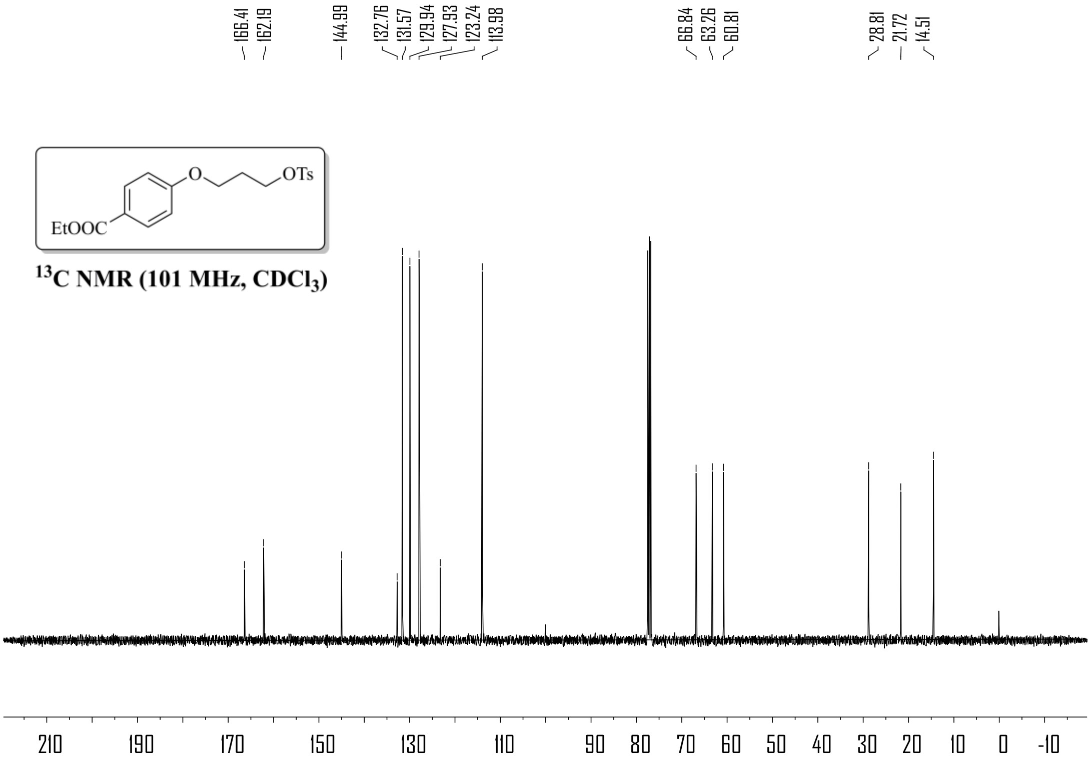

Supplementary Figure 13. ^{13}C NMR spectra for ethyl 4-(3-(tosyloxy)propoxy)benzoate

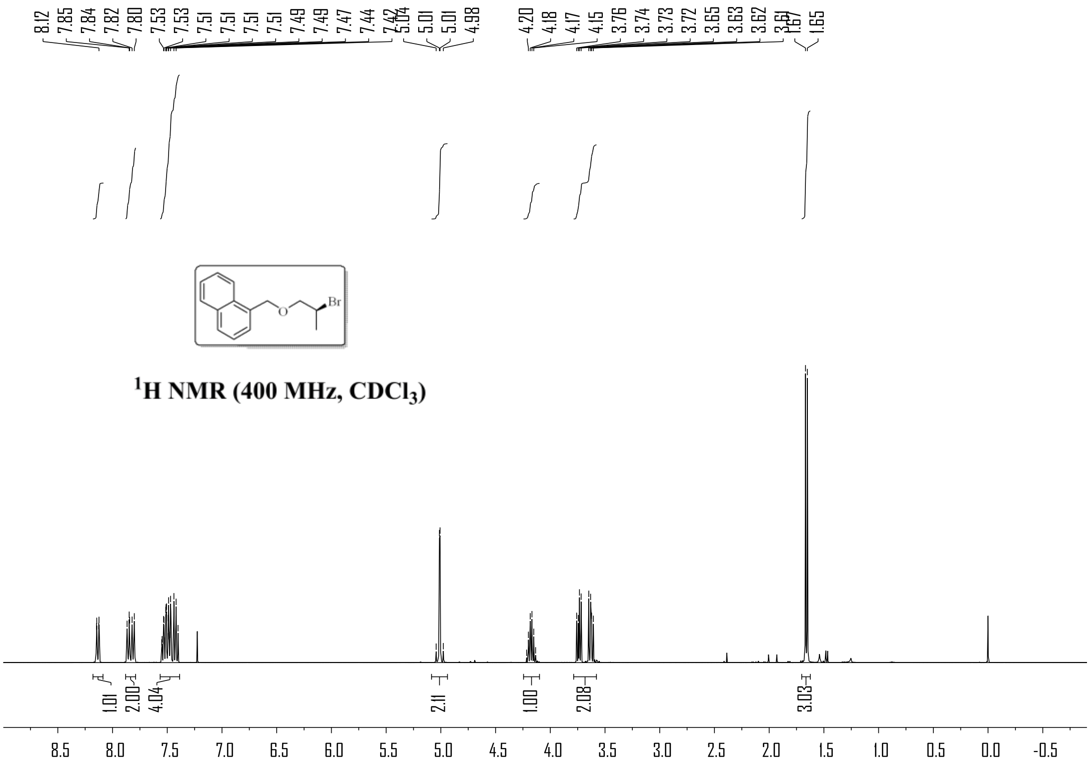

Supplementary Figure 14. ^{1}H NMR spectra for (*S*)-1-((2-bromopropoxy)methyl)naphthalene

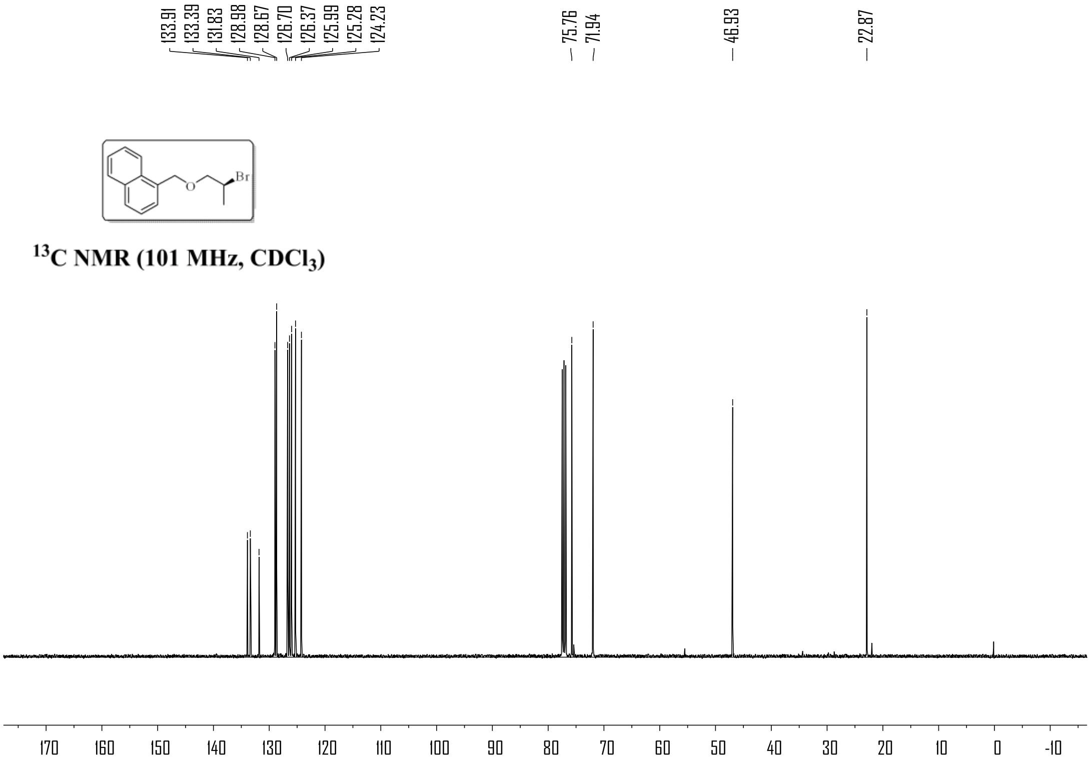

Supplementary Figure 15. ^{13}C NMR spectra for (*S*)-1-((2-bromopropoxy)methyl)naphthalene

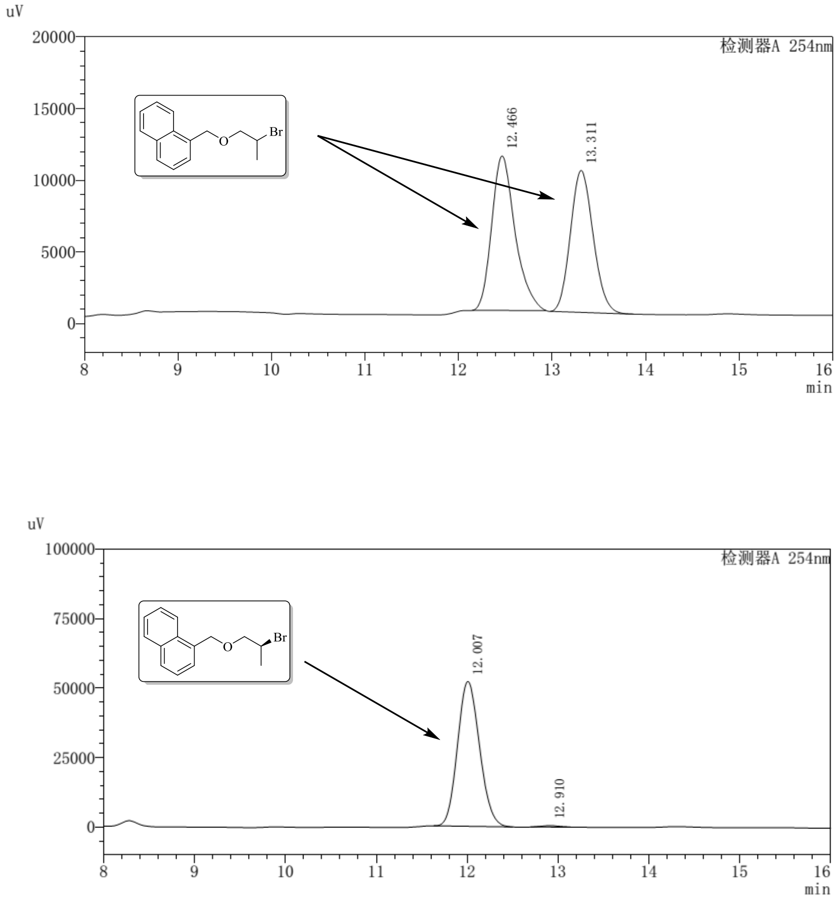

Supplementary Figure 16. HPLC spectra for (*S*)-1-((2-bromopropoxy)methyl)naphthalene

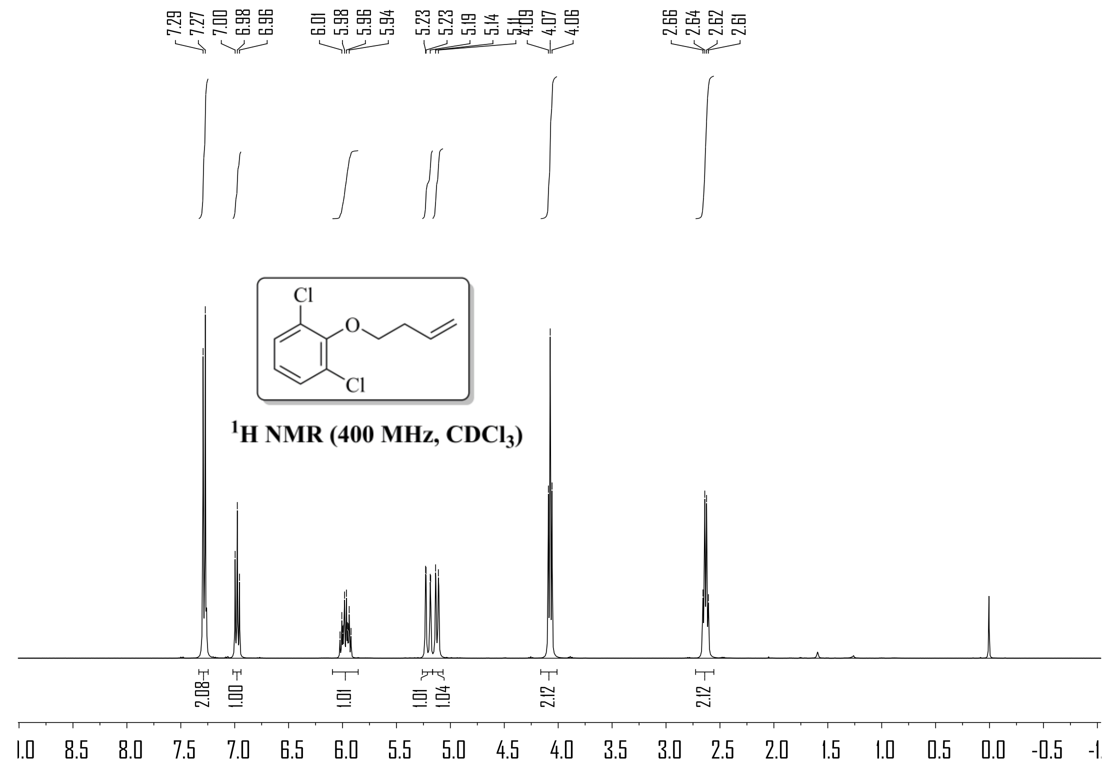

Supplementary Figure 17. ^{1}H NMR spectra for 2-(but-3-en-1-yloxy)-1,3-dichlorobenzene

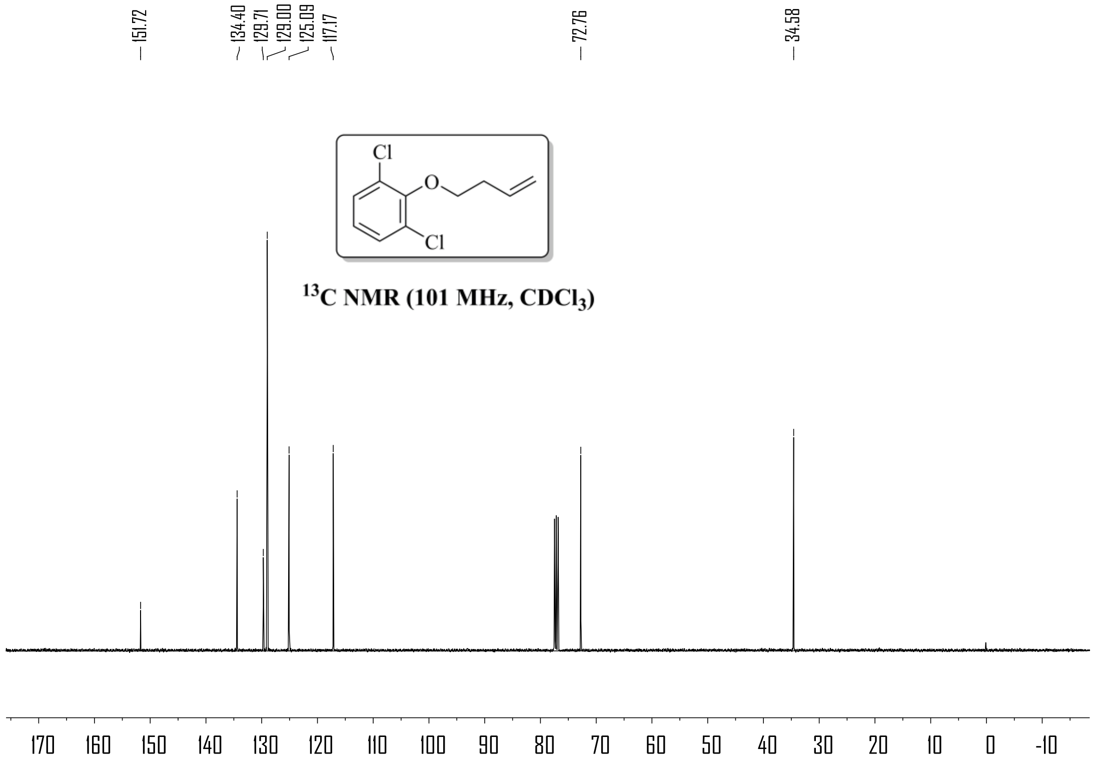

Supplementary Figure 18. ^{13}C NMR spectra for 2-(but-3-en-1-yloxy)-1,3-dichlorobenzene

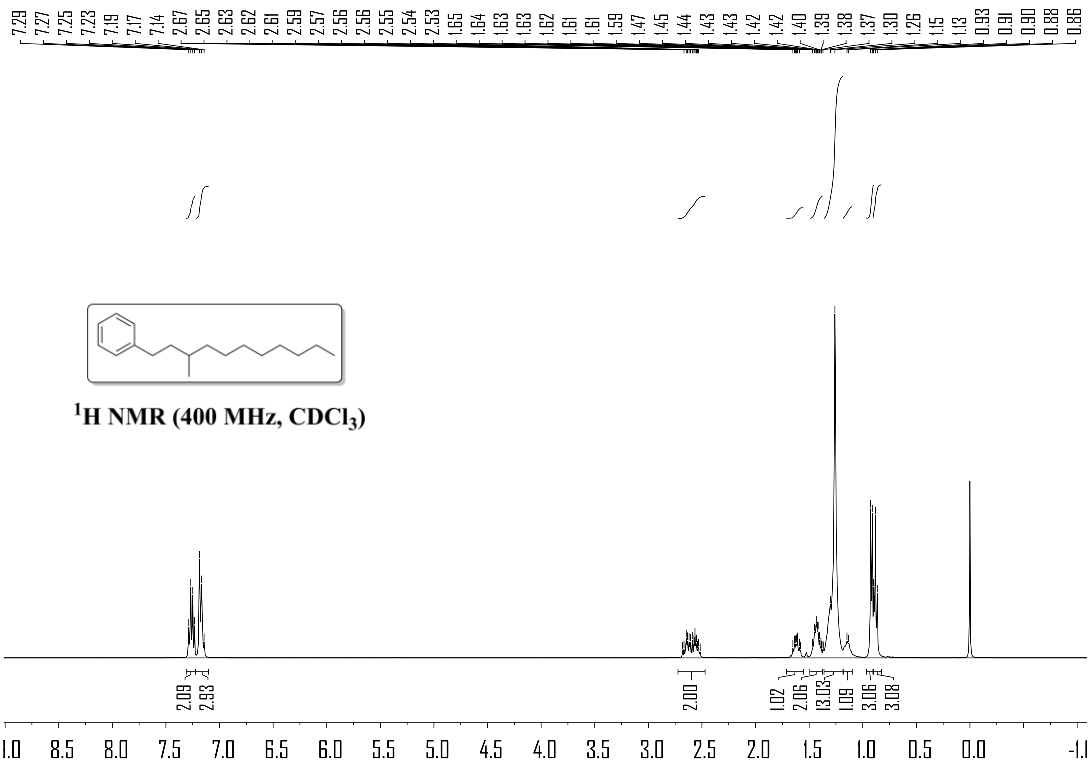

Supplementary Figure 19. ^{1}H NMR spectra for compound 3

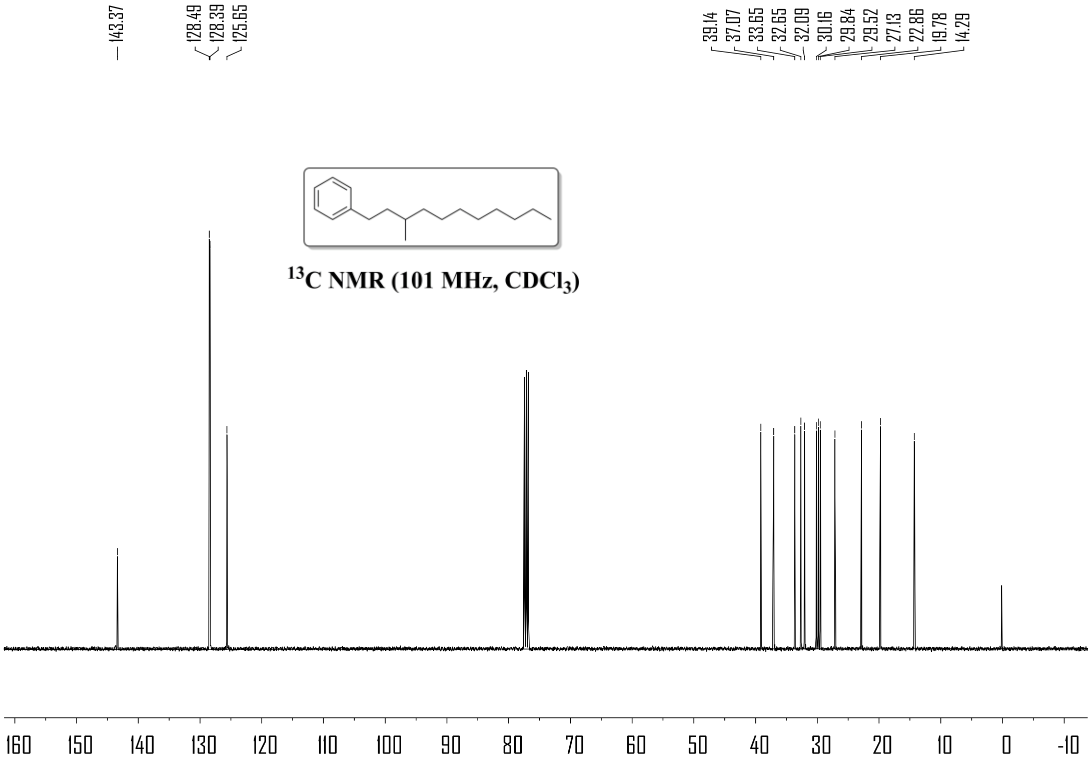

Supplementary Figure 20. ^{13}C NMR spectra for compound 3

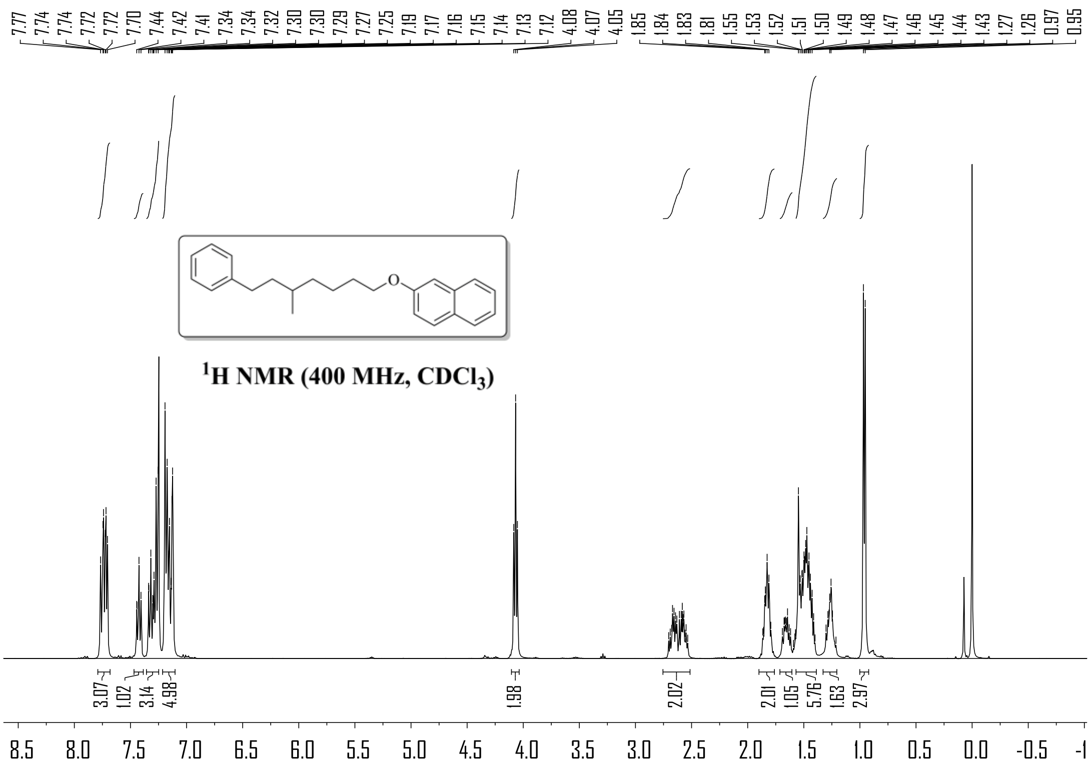

Supplementary Figure 21. ^{1}H NMR spectra for compound 4

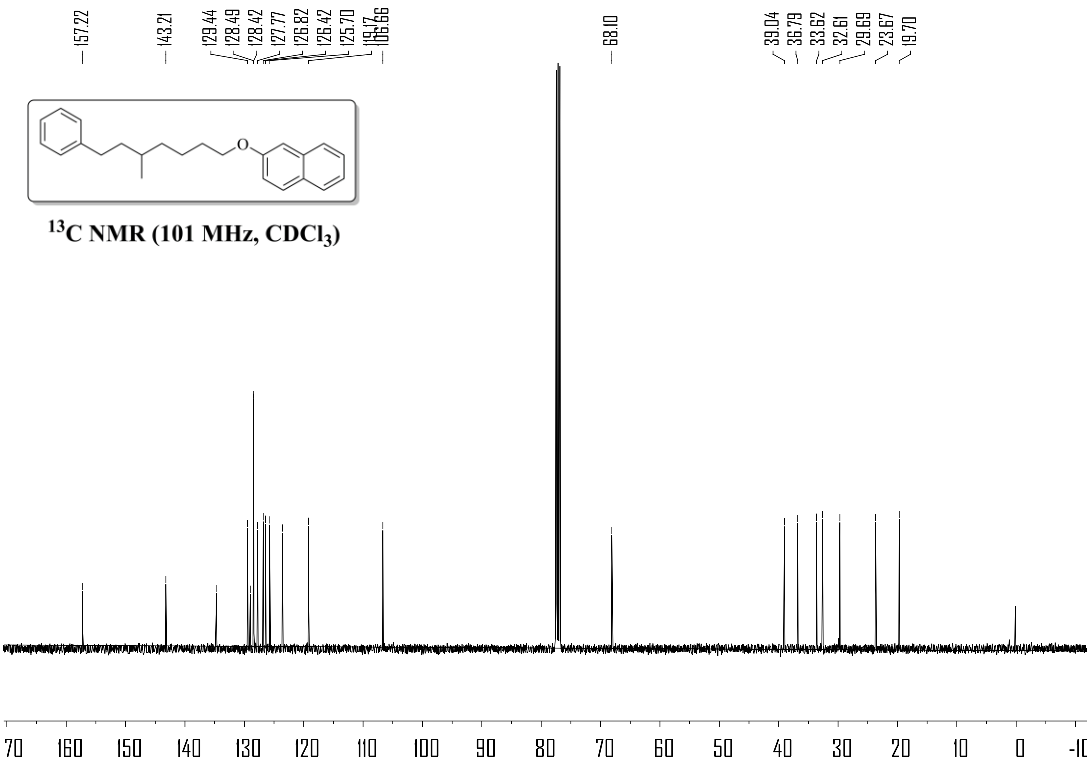

Supplementary Figure 22. ^{13}C NMR spectra for compound 4

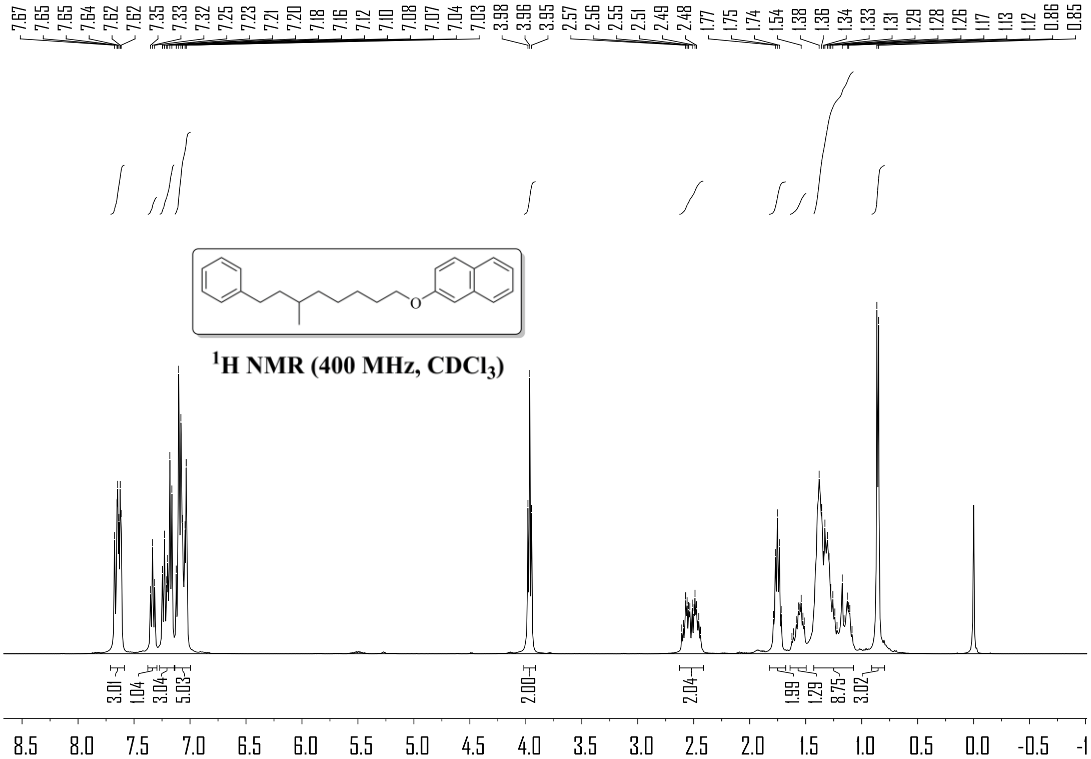

Supplementary Figure 23. ^{1}**H NMR spectra for compound 5**

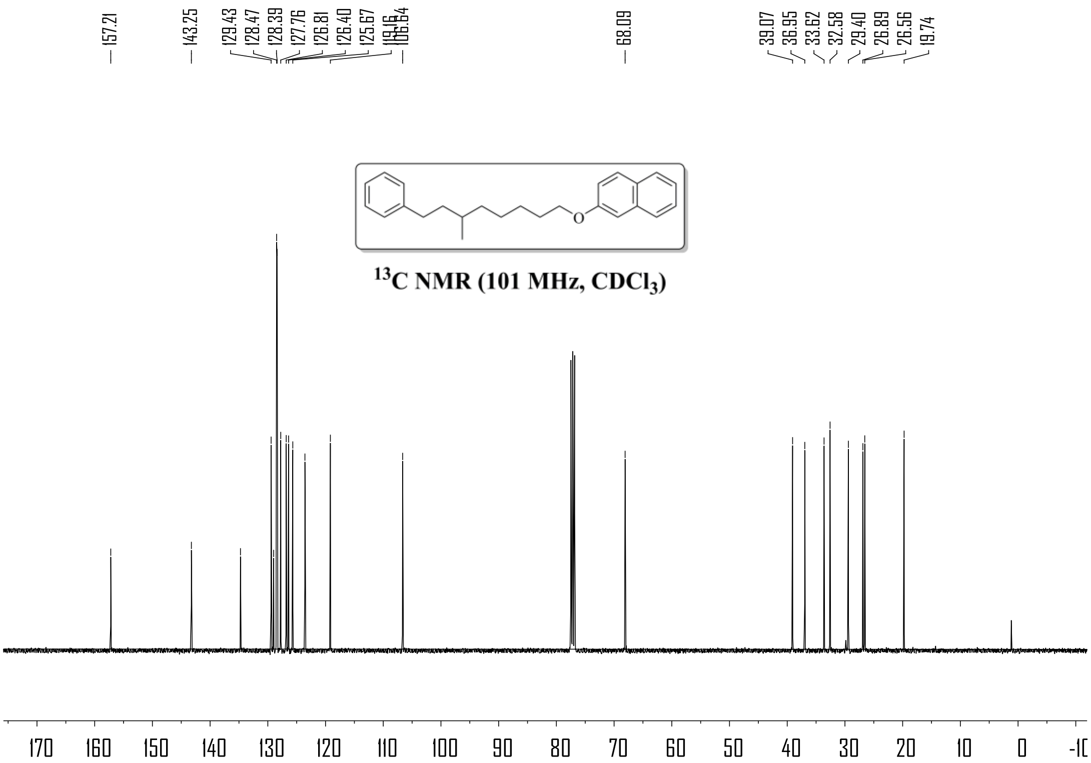

Supplementary Figure 24. ^{13}**C NMR spectra for compound 5**

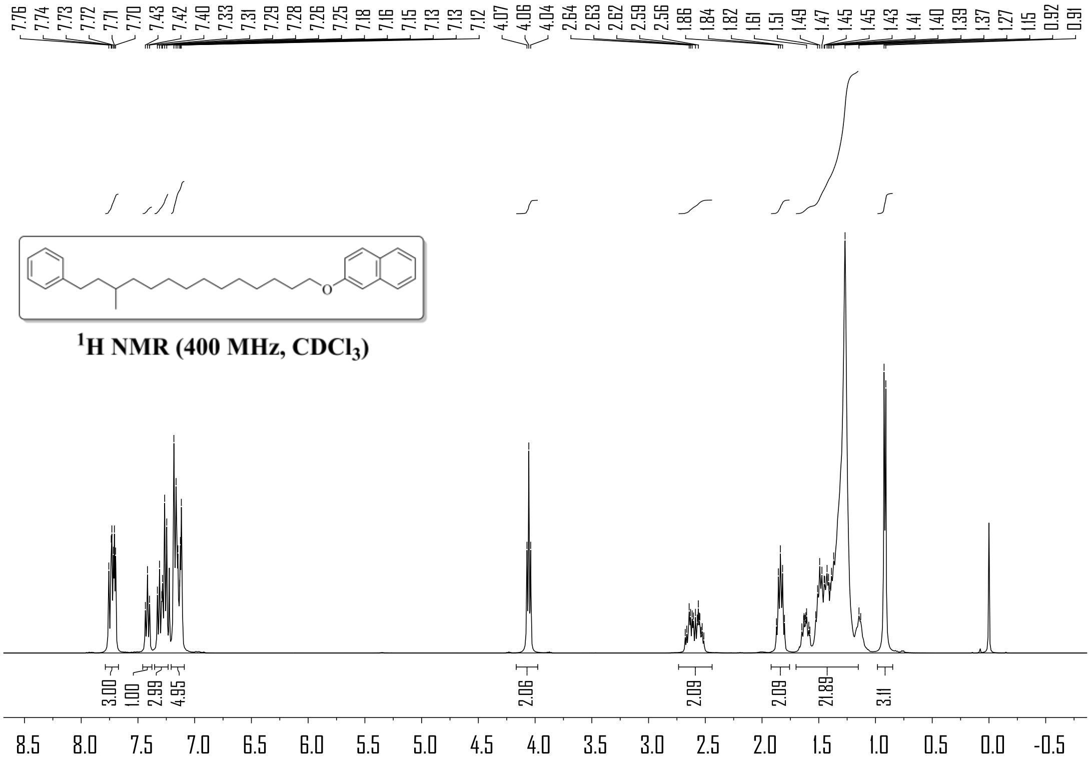

Supplementary Figure 25. ^{1}H NMR spectra for compound 6

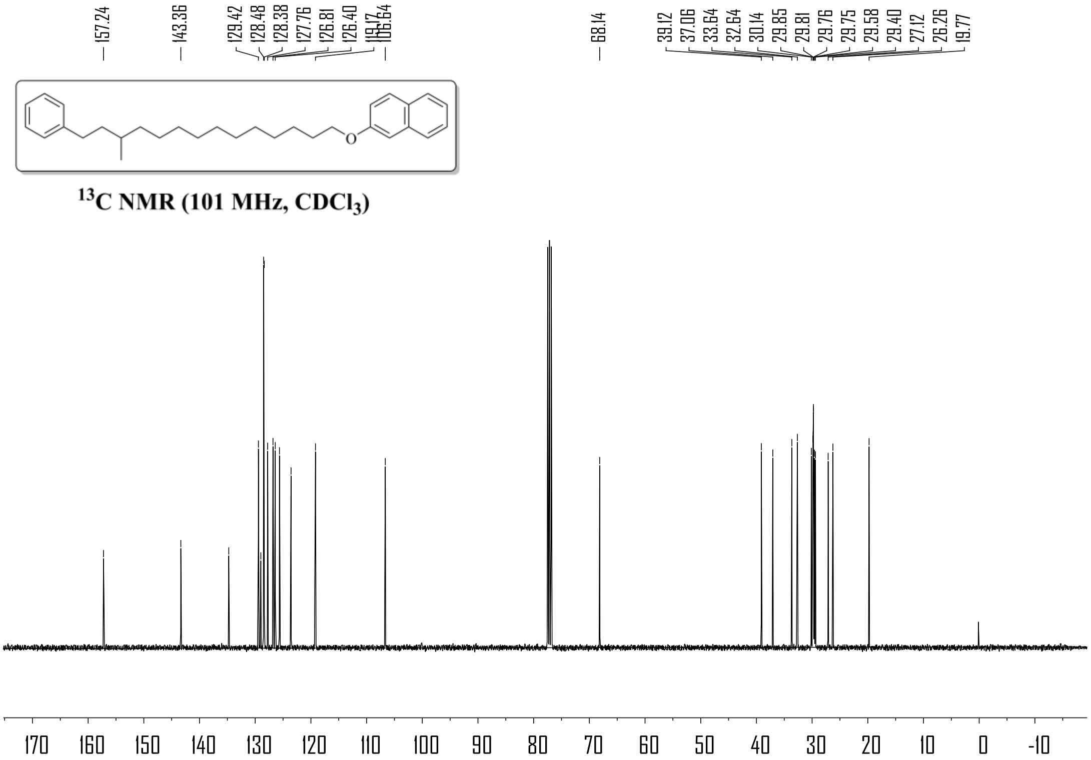

Supplementary Figure 26. ^{13}C NMR spectra for compound 6

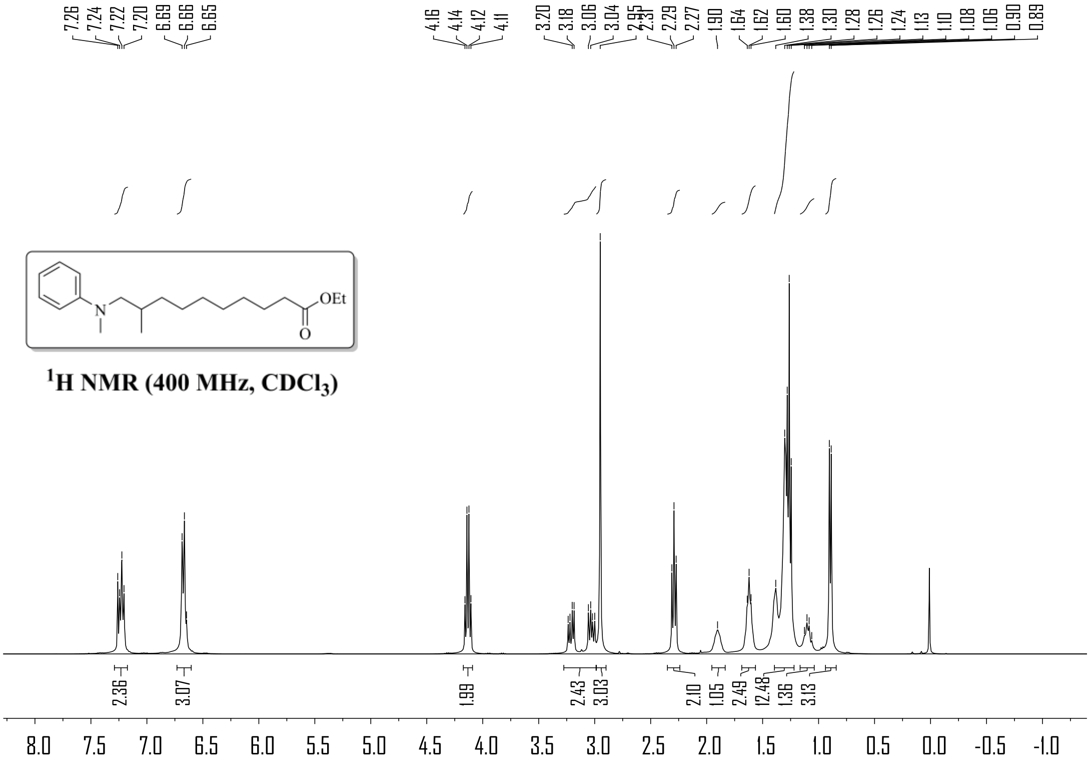

Supplementary Figure 27. ^{1}H **NMR spectra for compound 7**

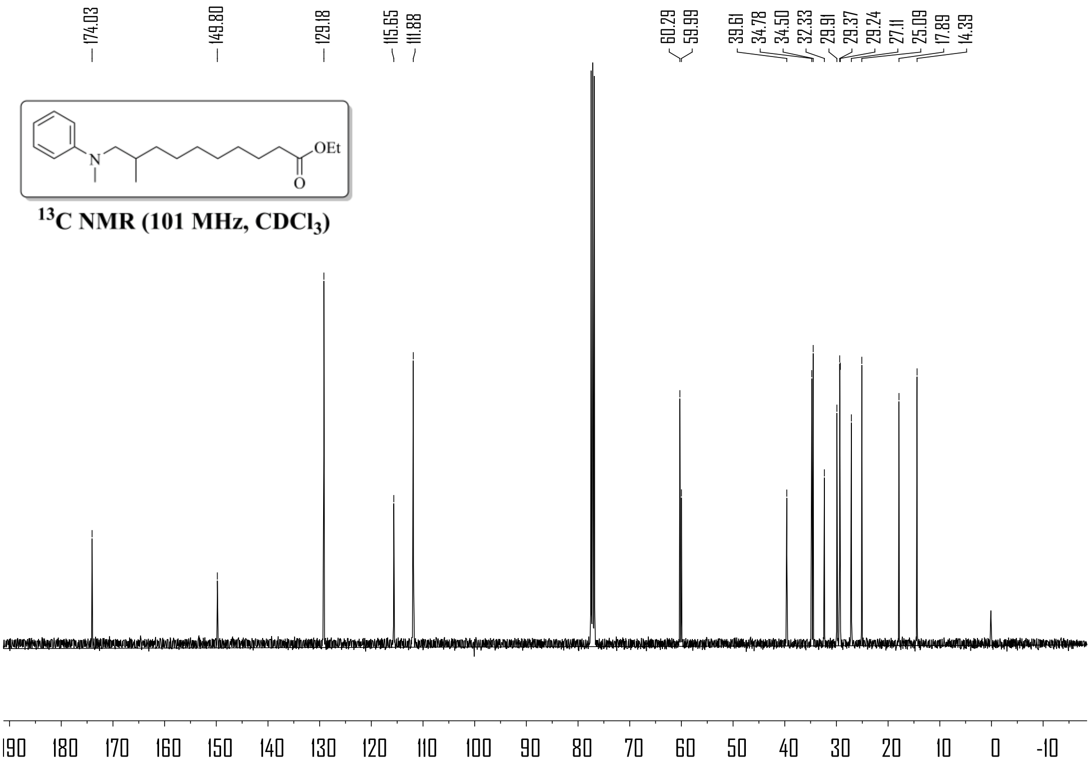

Supplementary Figure 28. ^{13}C **NMR spectra for compound 7**

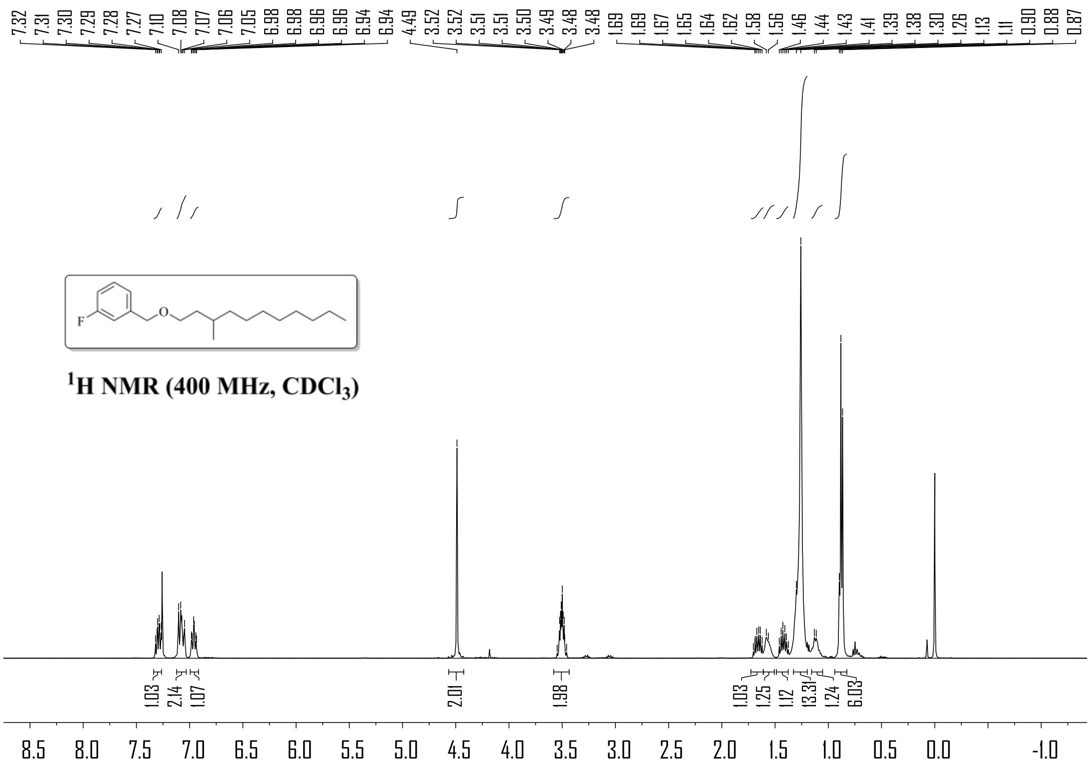

Supplementary Figure 29. ^{1}H NMR spectra for compound 8

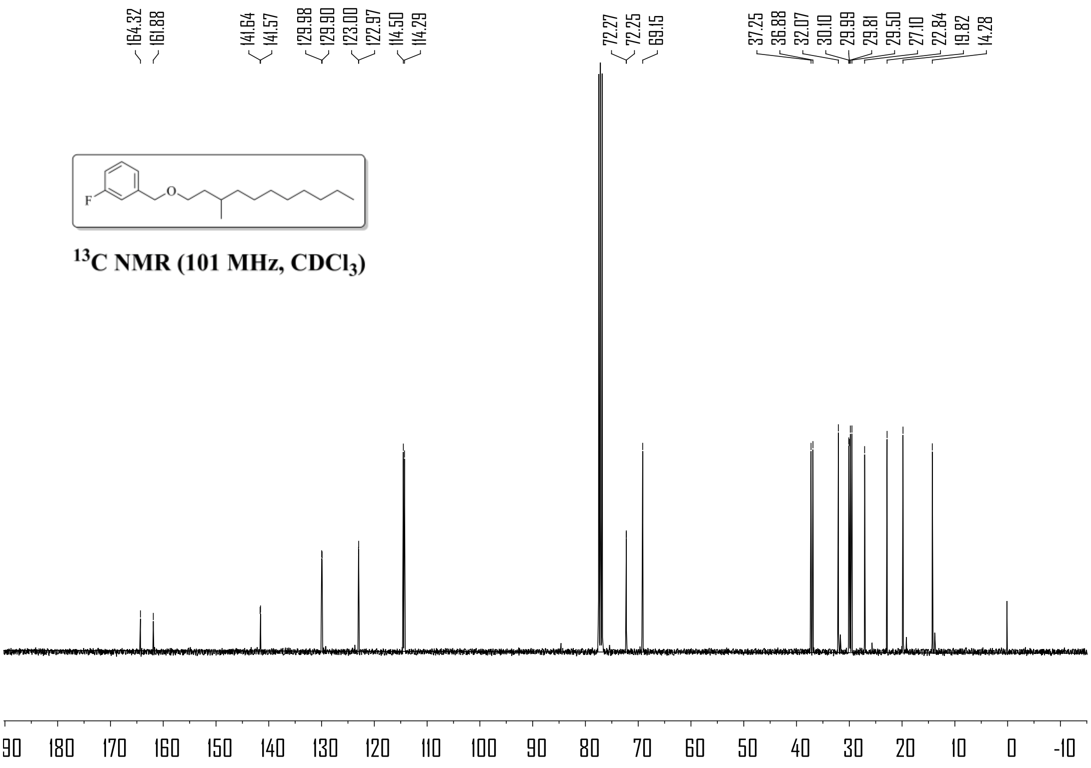

Supplementary Figure 30. ^{13}C NMR spectra for compound 8

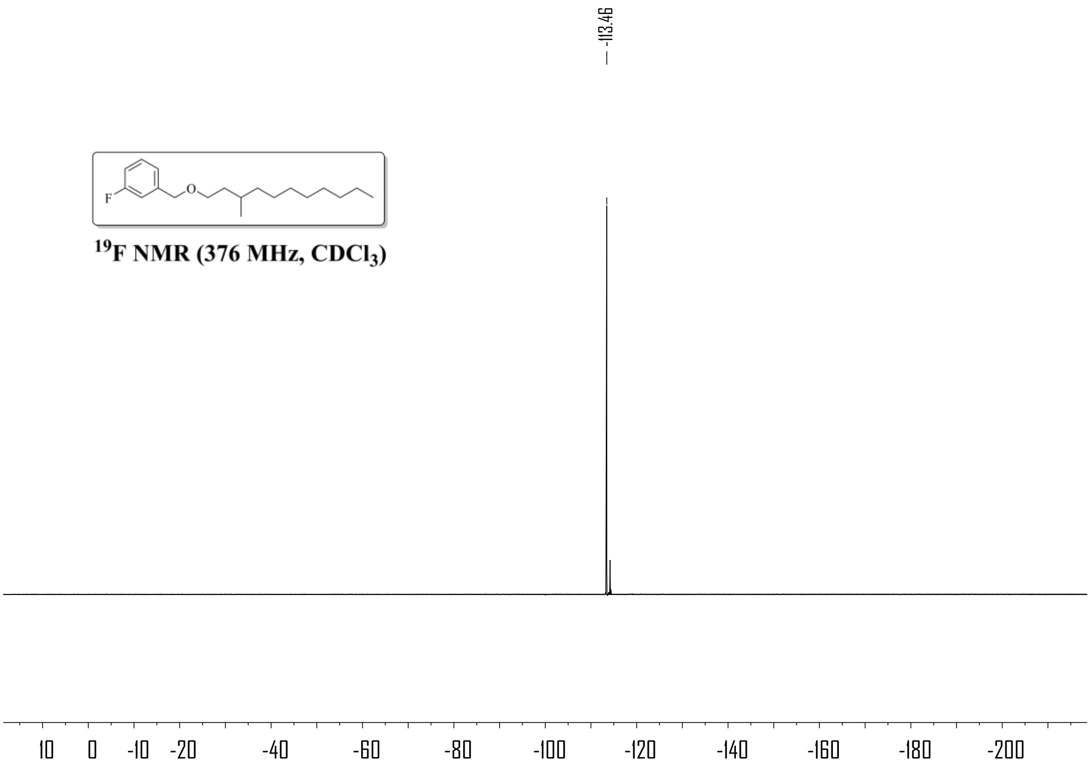

Supplementary Figure 31. ^{19}F NMR spectra for compound 8

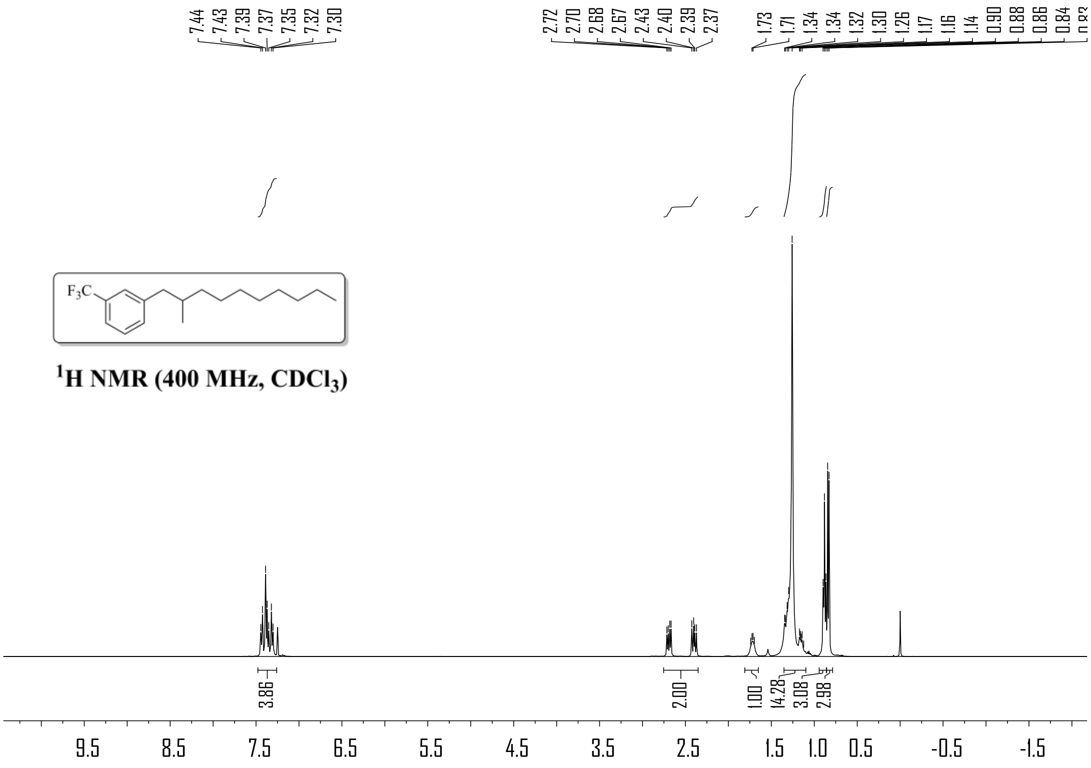

Supplementary Figure 32. ^{1}H NMR spectra for compound 9

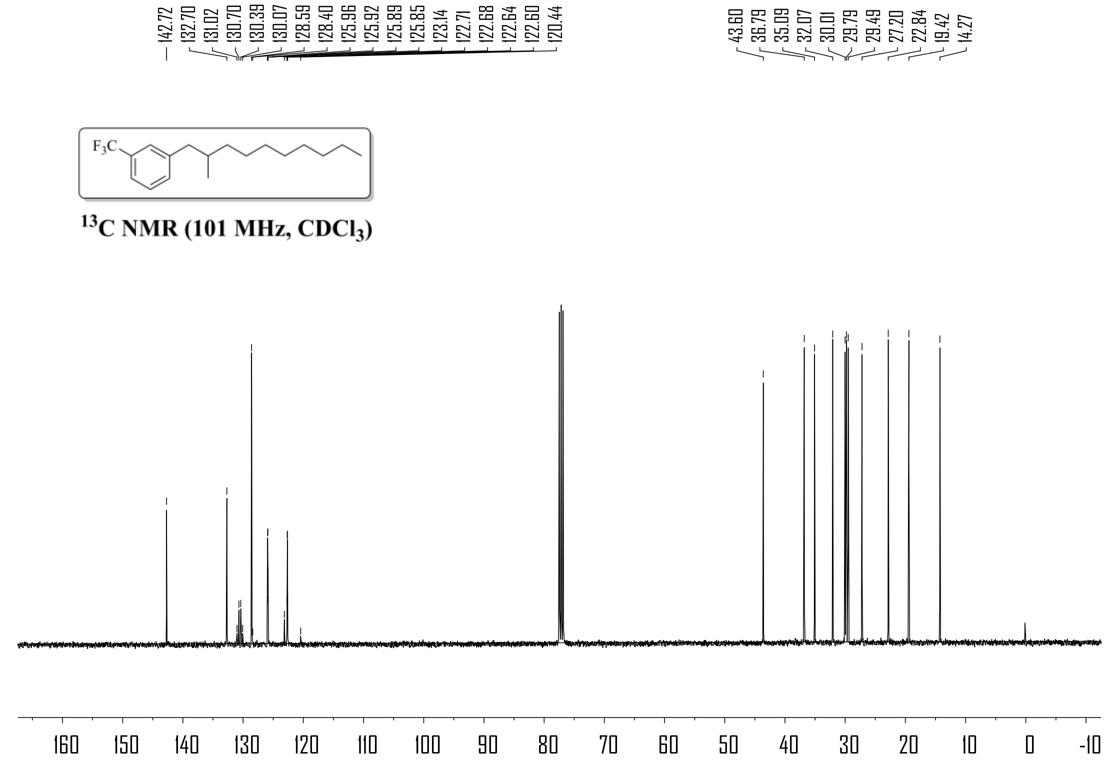

Supplementary Figure 33. ^{13}C **NMR spectra for compound 9**

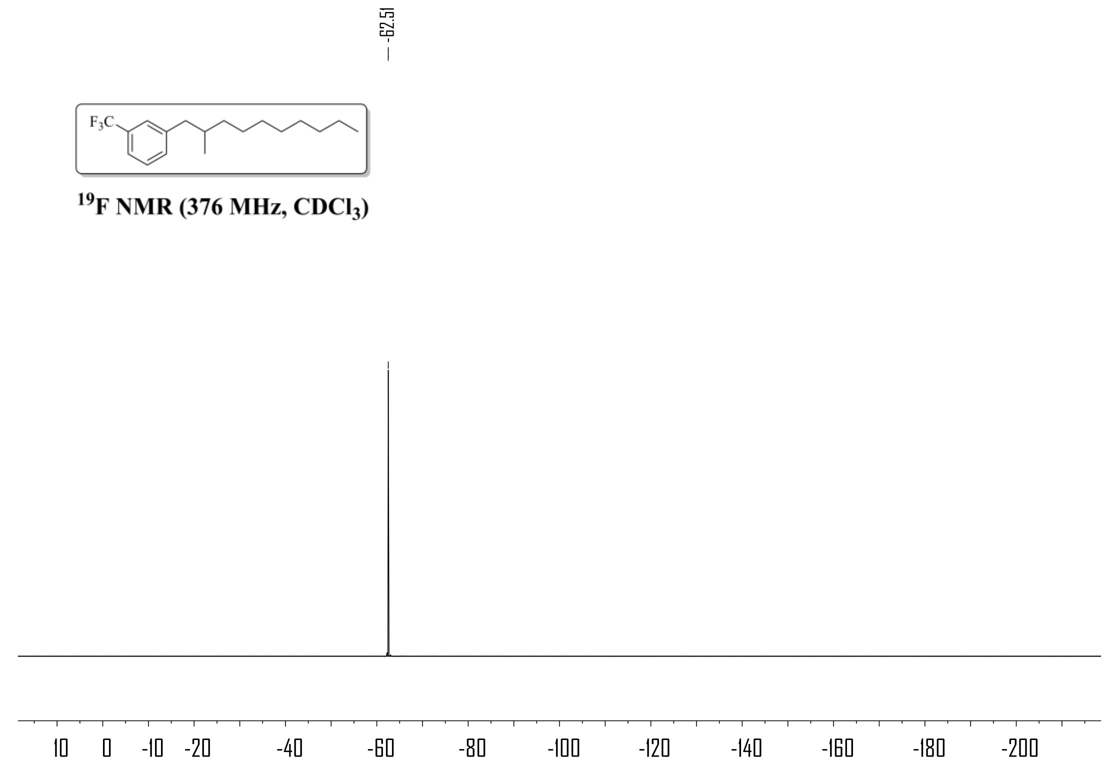

Supplementary Figure 34. ^{19}F **NMR spectra for compound 9**

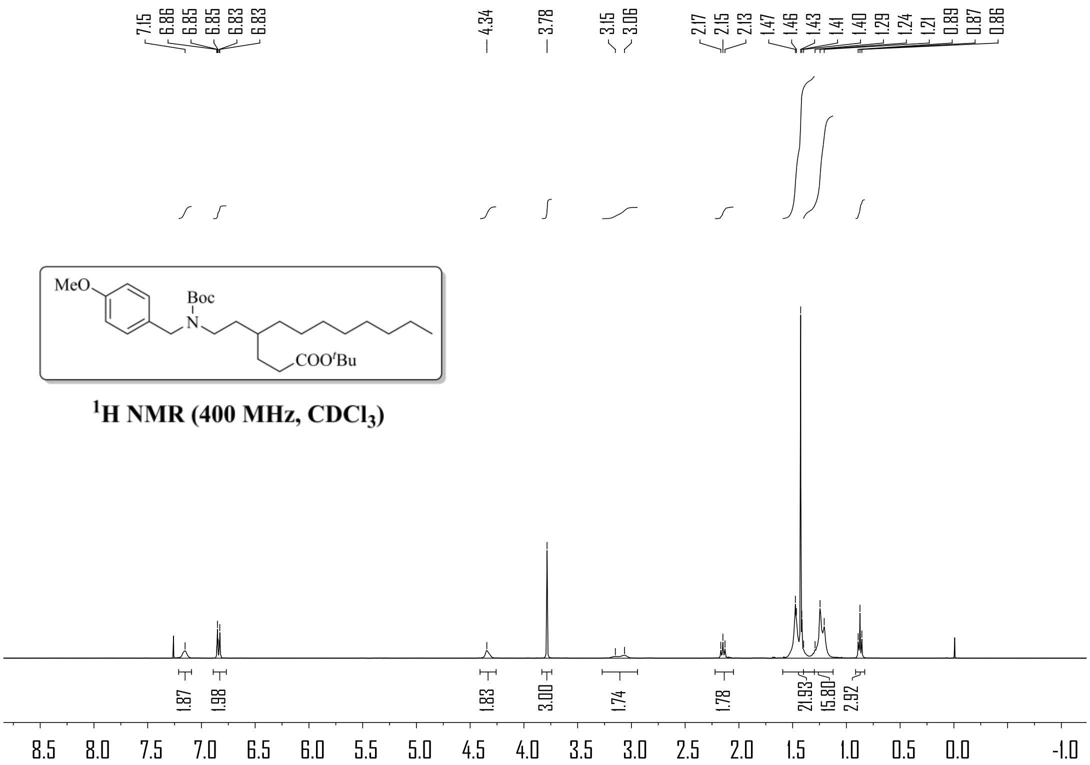

Supplementary Figure 35. ^{1}H NMR spectra for compound 10

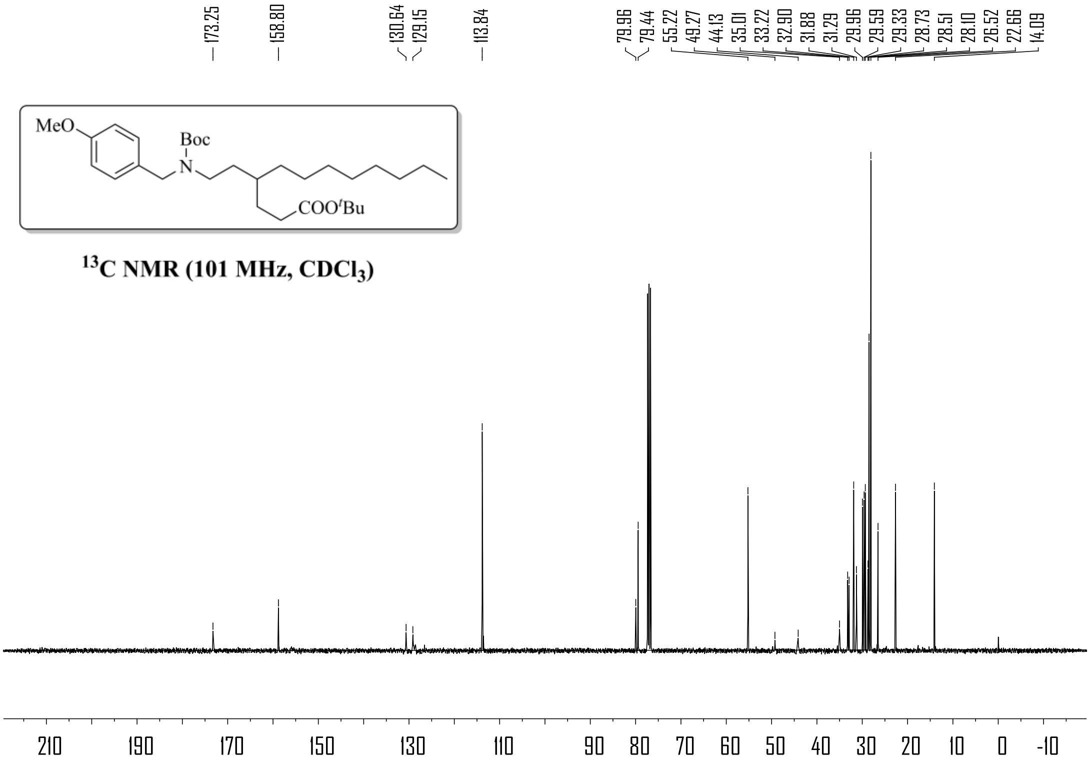

Supplementary Figure 36. ^{13}C NMR spectra for compound 10

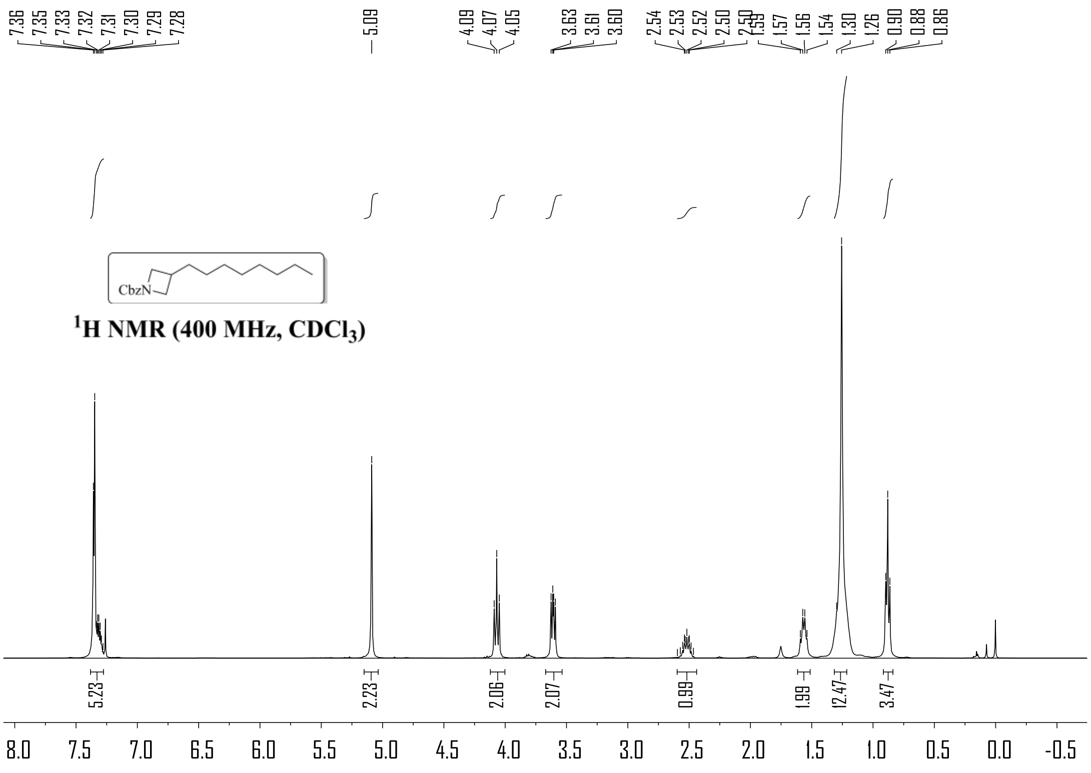

Supplementary Figure 37. ^{1}H NMR spectra for compound 11

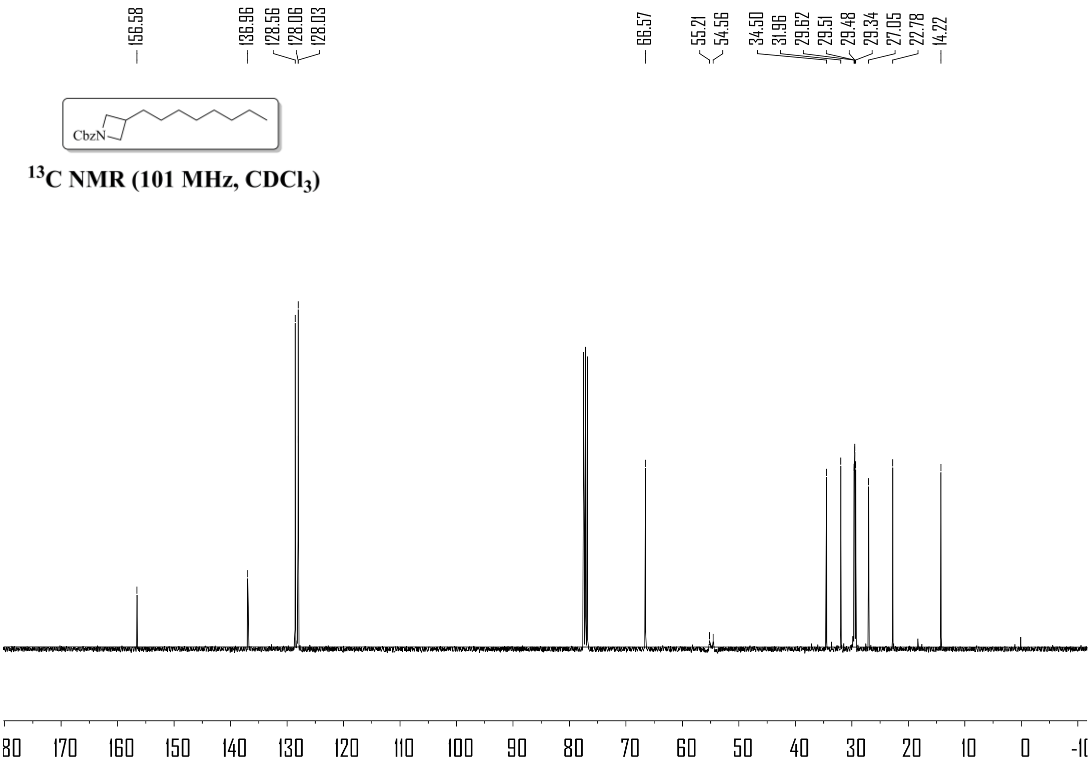

Supplementary Figure 38. ^{13}C NMR spectra for compound 11

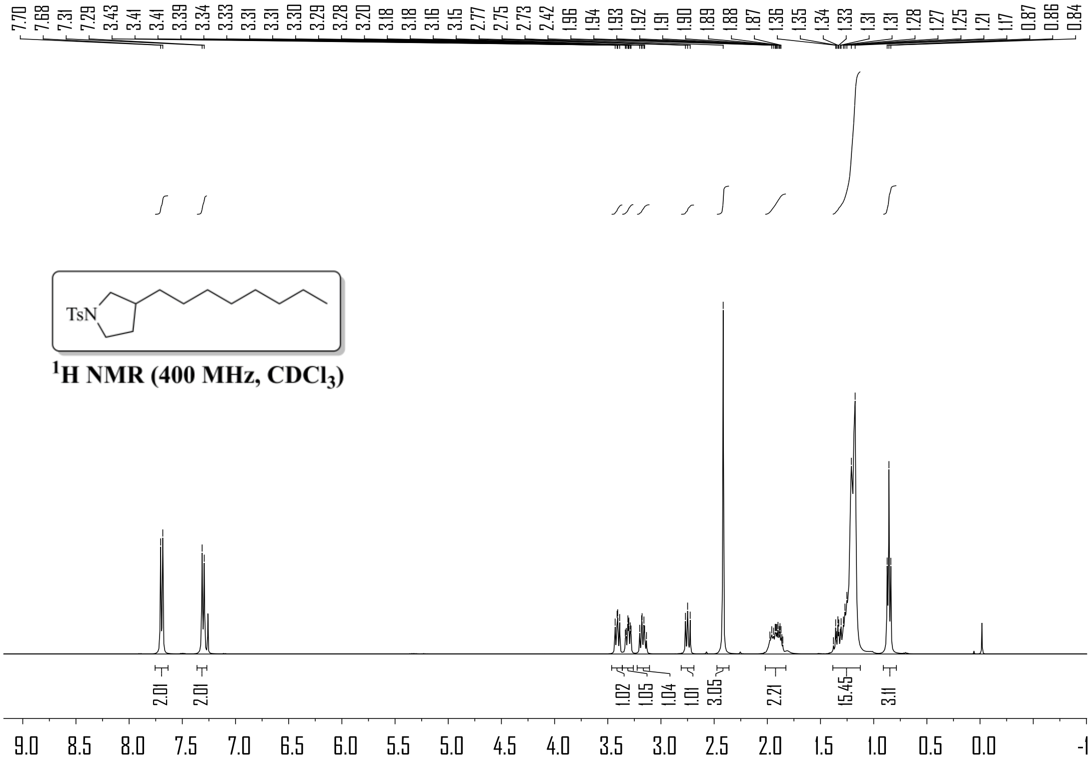

Supplementary Figure 39. ^{1}H NMR spectra for compound 12

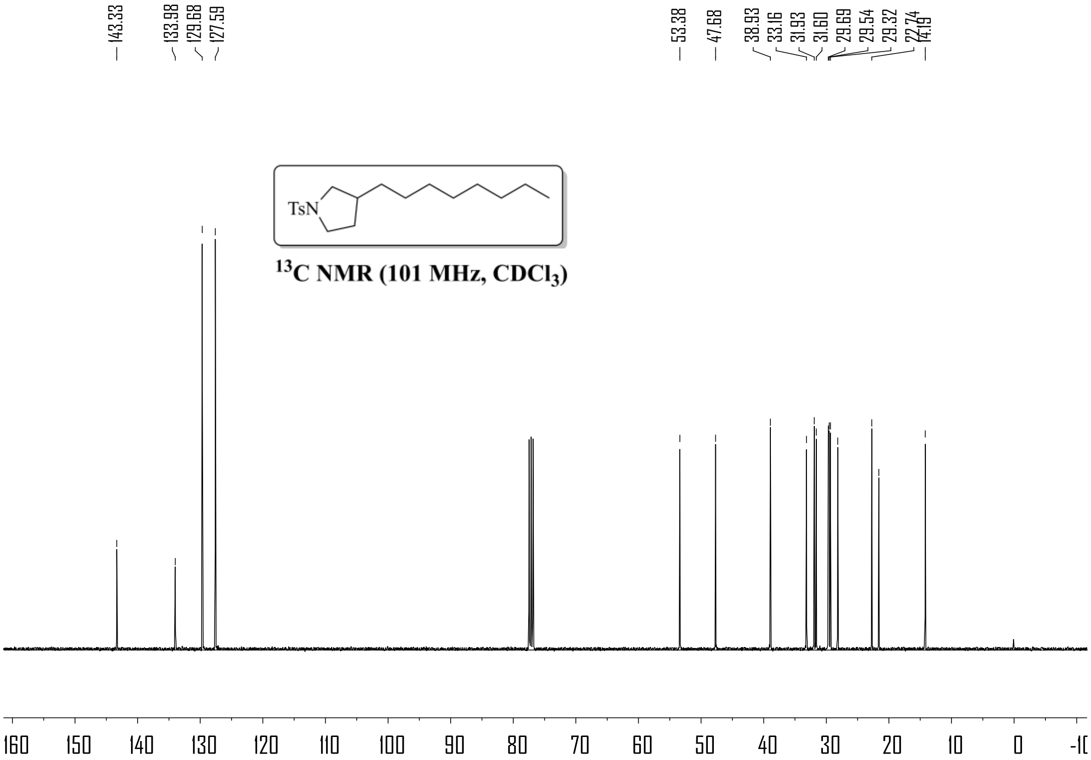

Supplementary Figure 40. ^{13}C NMR spectra for compound 12

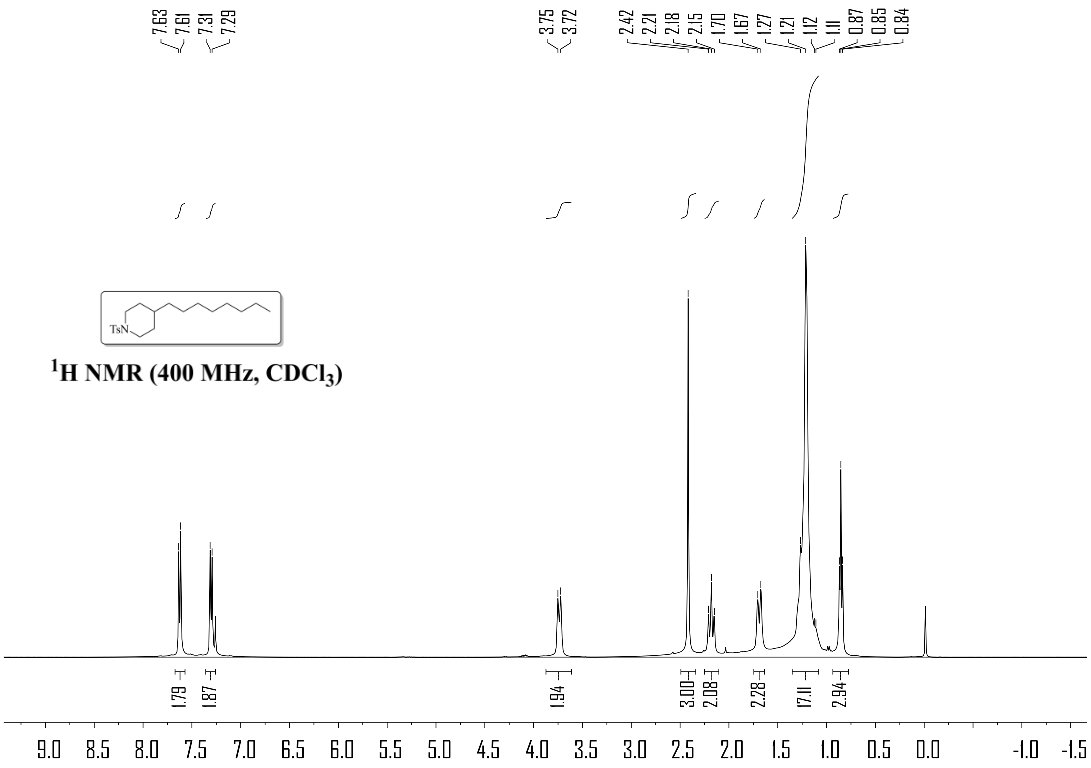

Supplementary Figure 41. ^{1}**H NMR spectra for compound 13**

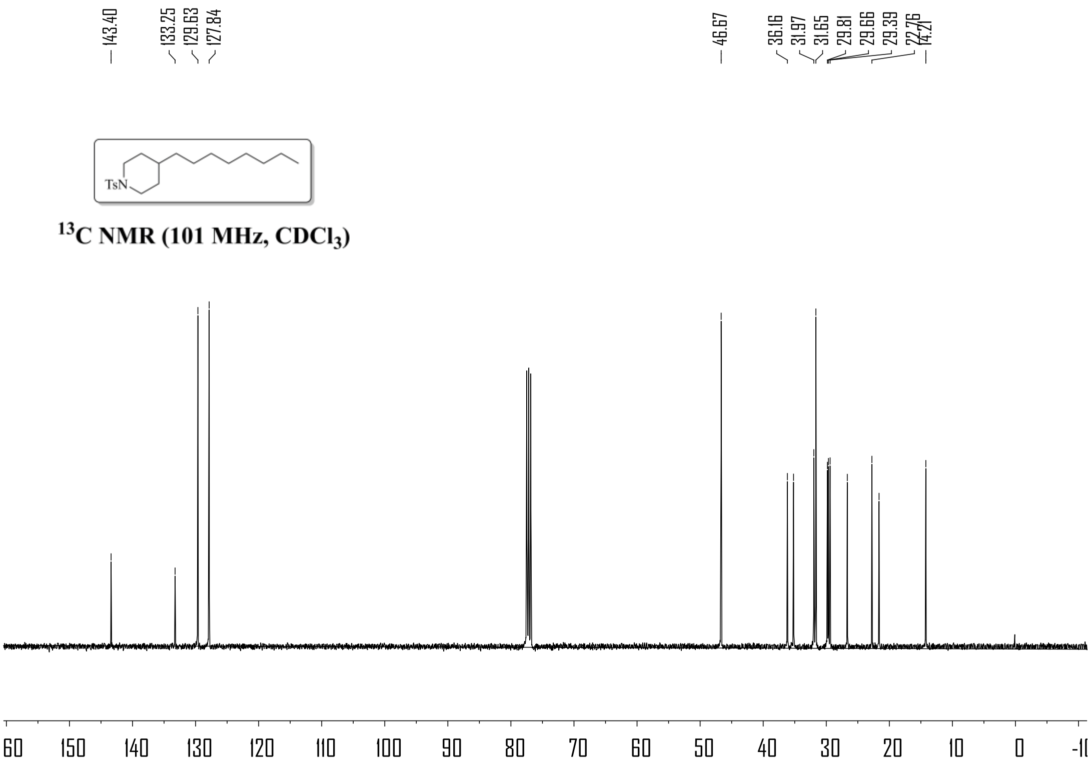

Supplementary Figure 42. ^{13}**C NMR spectra for compound 13**

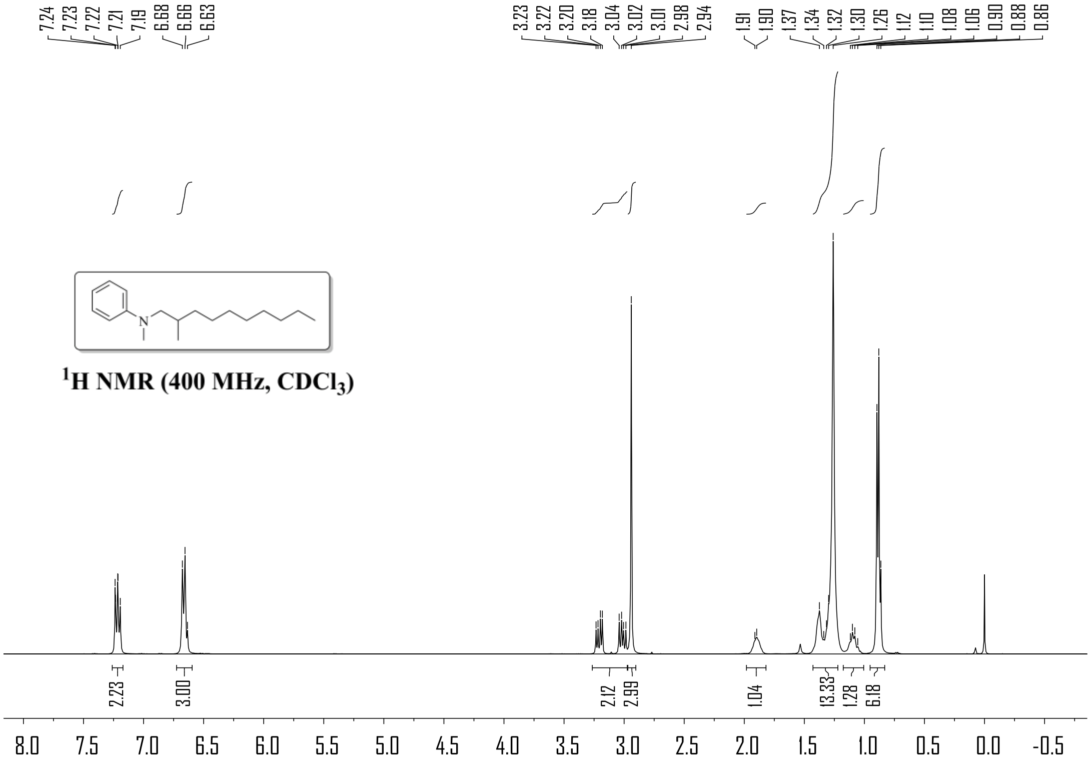

Supplementary Figure 43. ^{1}H NMR spectra for compound 14

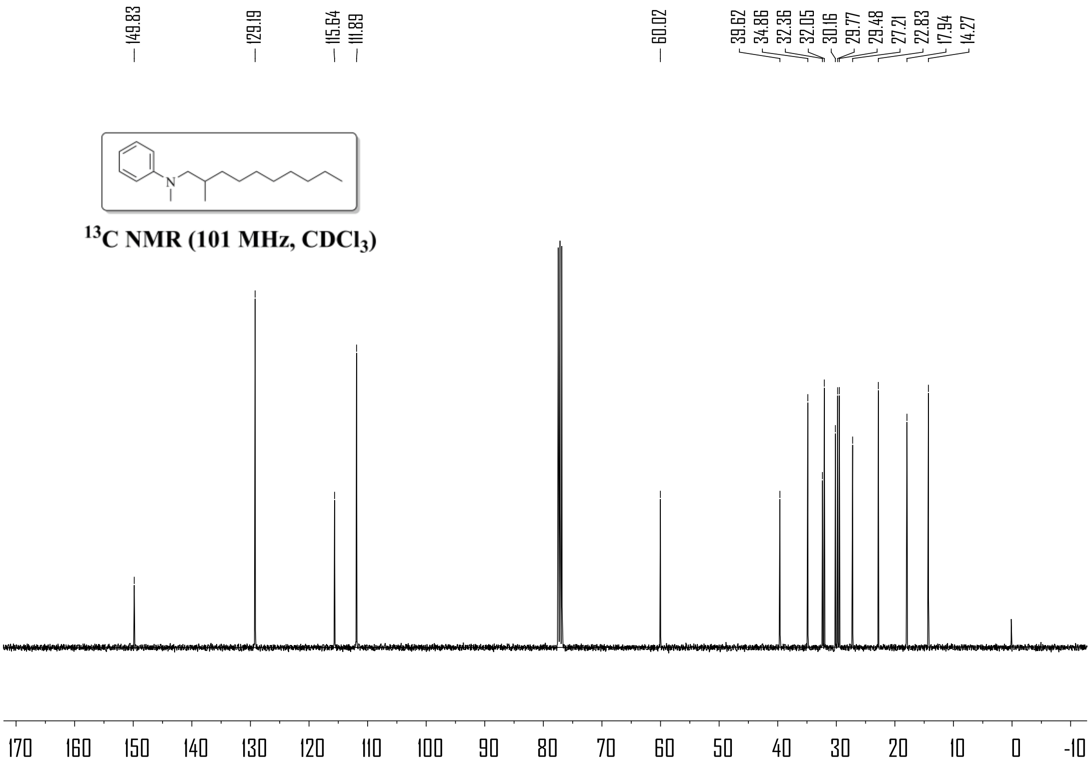

Supplementary Figure 44. ^{13}C NMR spectra for compound 14

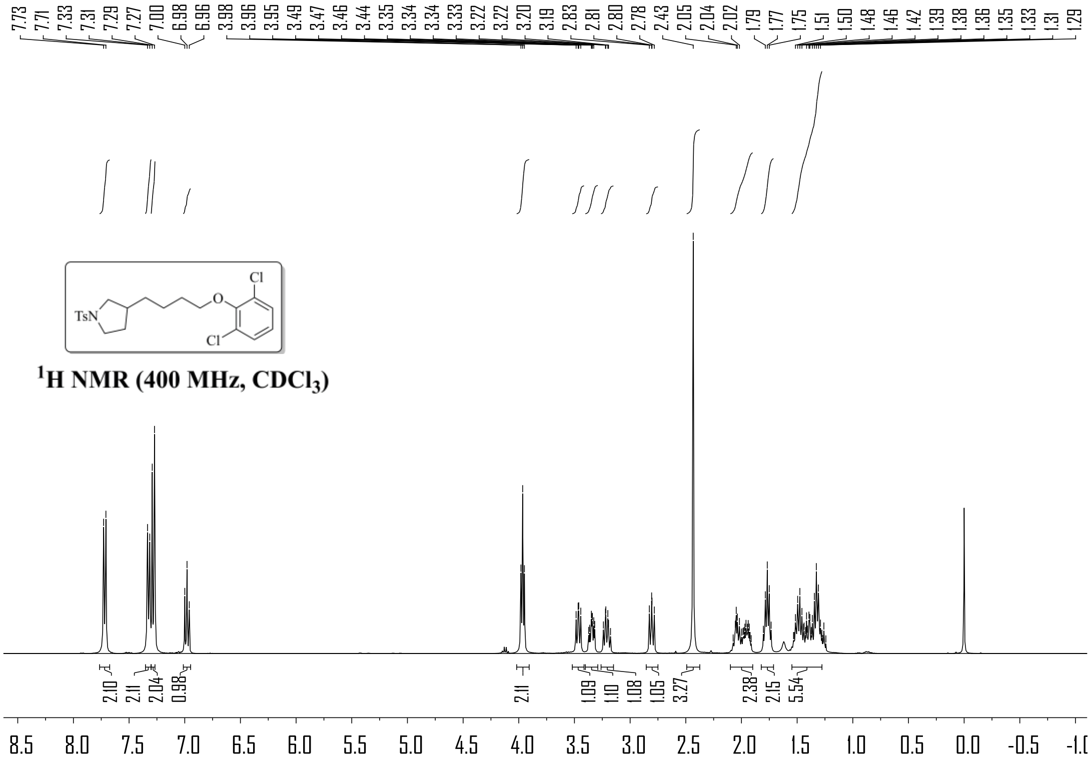

Supplementary Figure 45. ^{1}**H NMR spectra for compound 15**

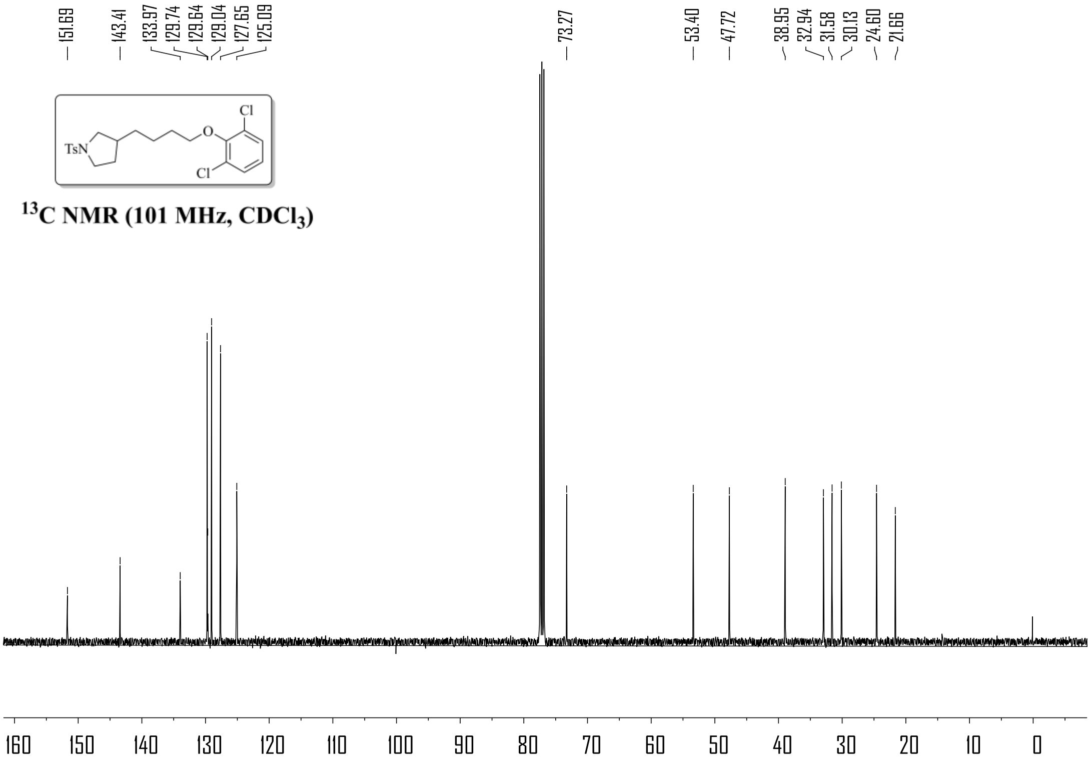

Supplementary Figure 46. ^{13}**C NMR spectra for compound 15**

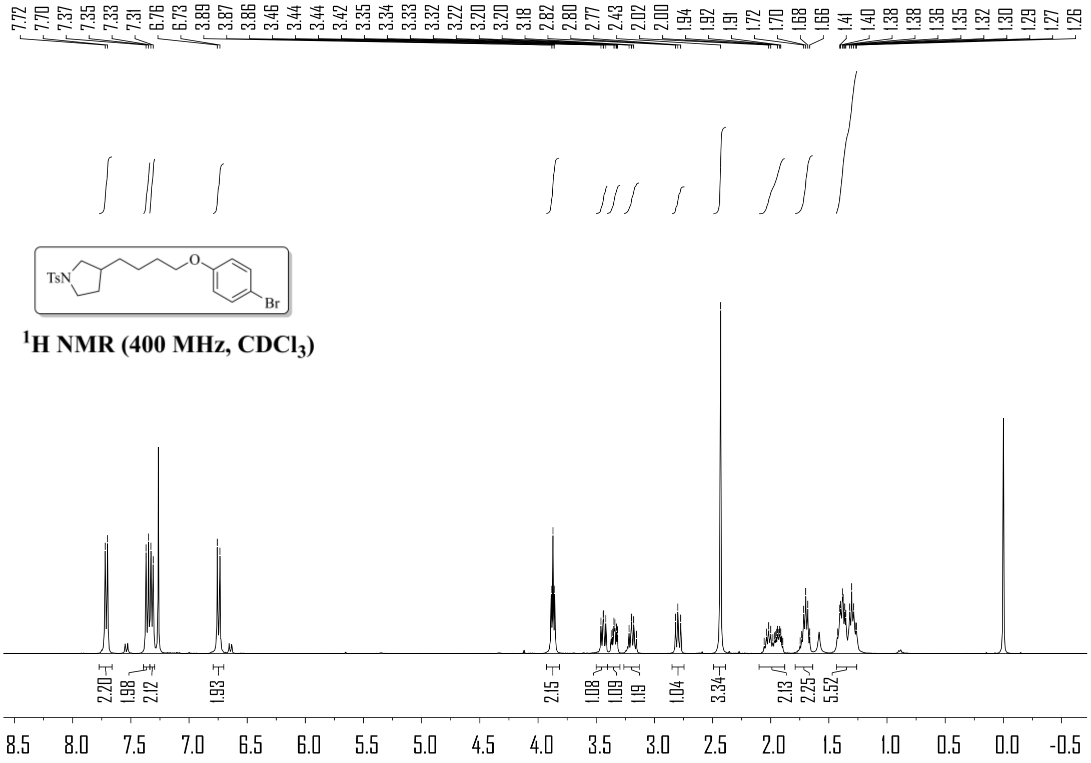

Supplementary Figure 47. ^{1}H NMR spectra for compound 16

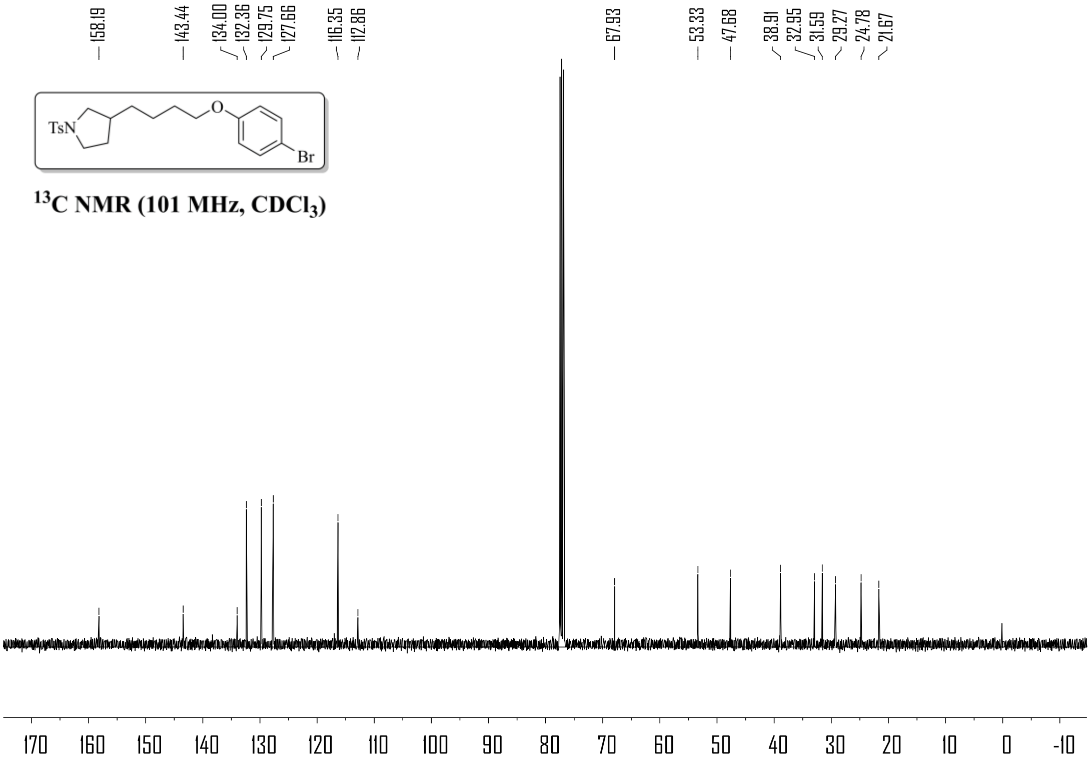

Supplementary Figure 48. ^{13}C NMR spectra for compound 16

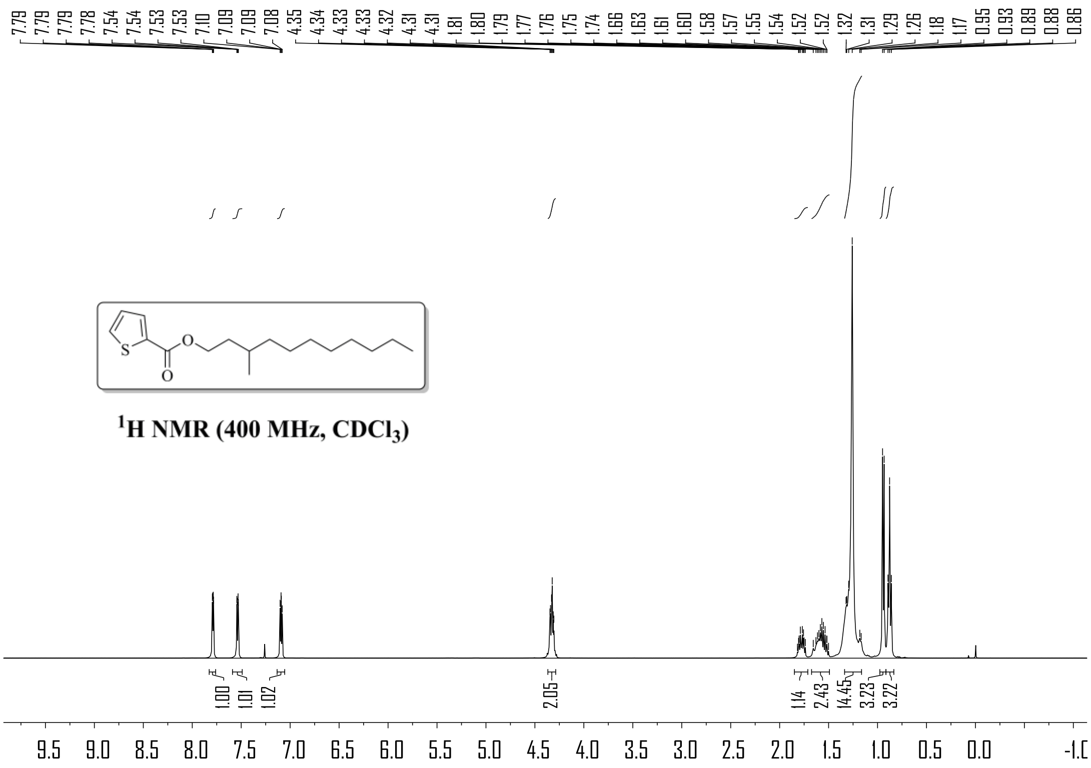

Supplementary Figure 49. ^{1}H NMR spectra for compound 17

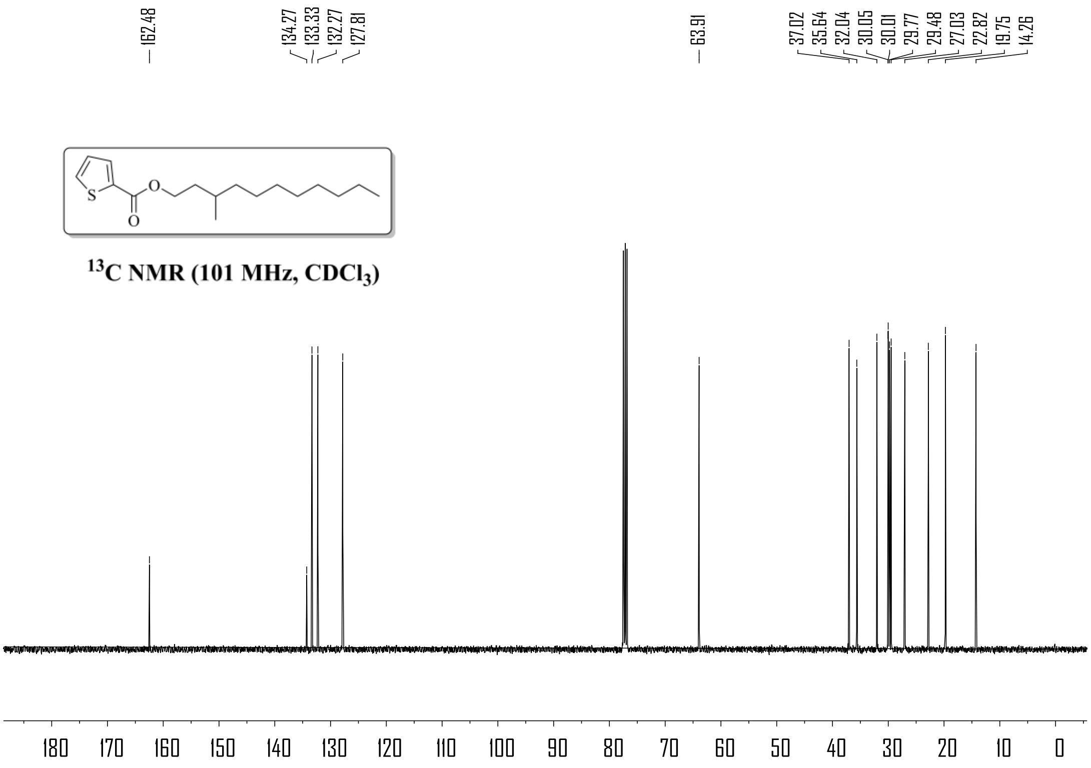

Supplementary Figure 50. ^{13}C NMR spectra for compound 17

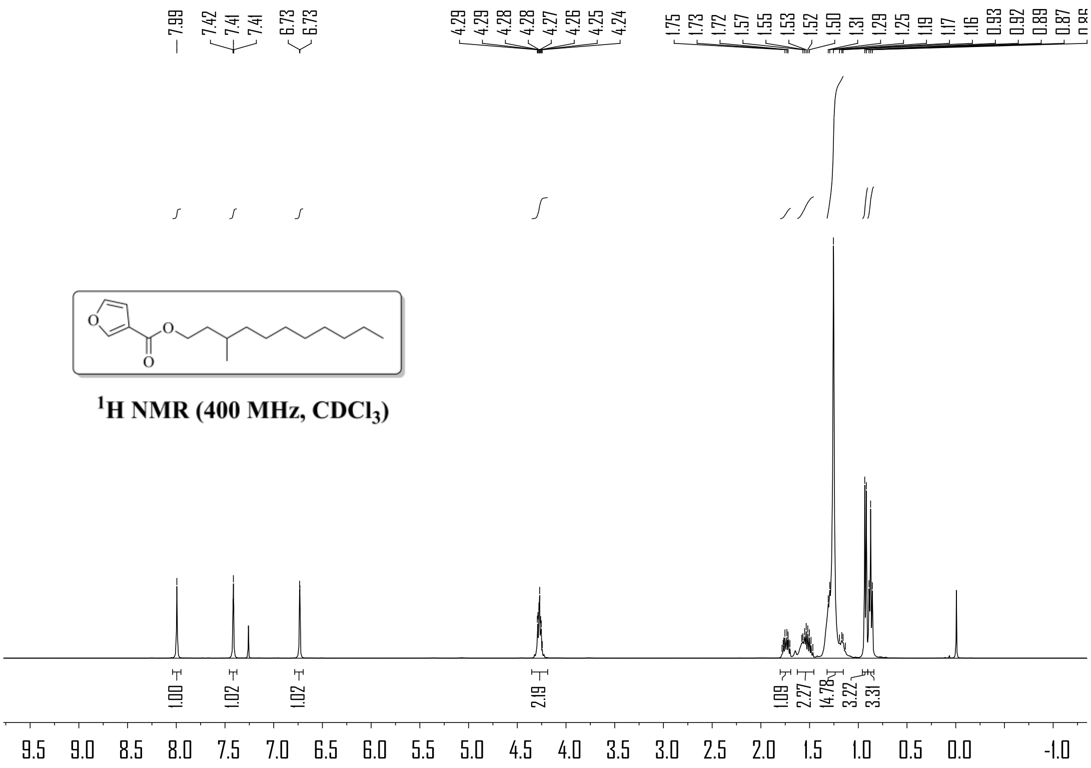

Supplementary Figure 51. ^{1}H NMR spectra for compound 18

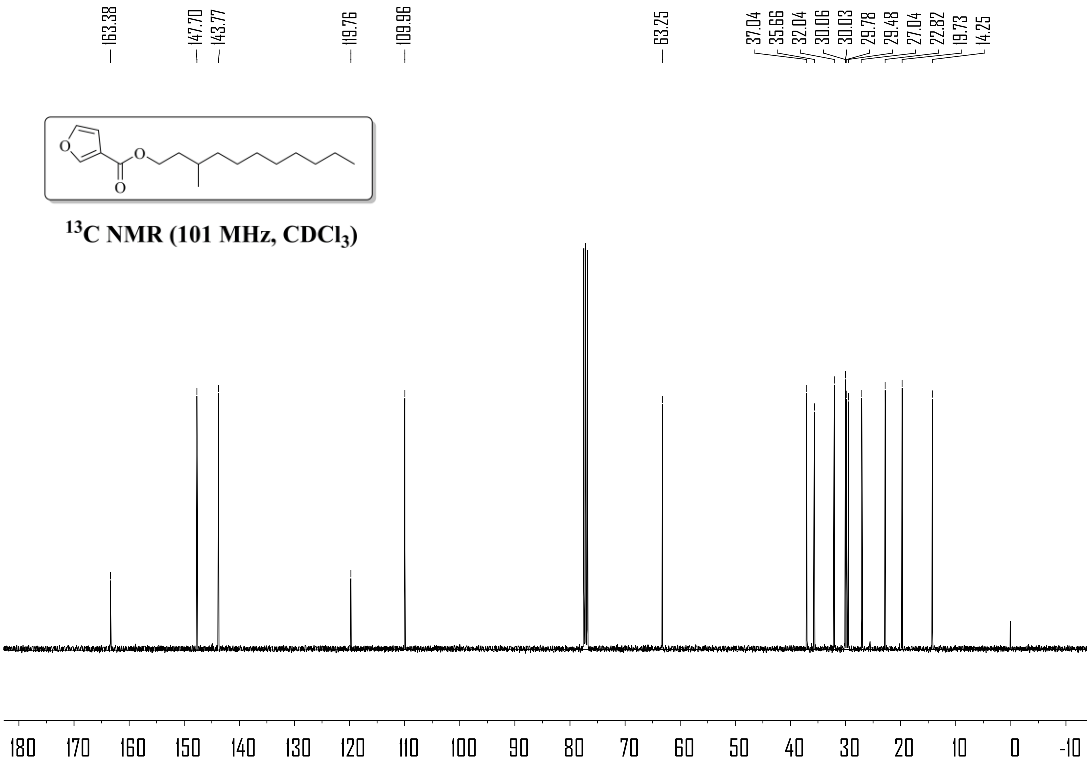

Supplementary Figure 52. ^{13}C NMR spectra for compound 18

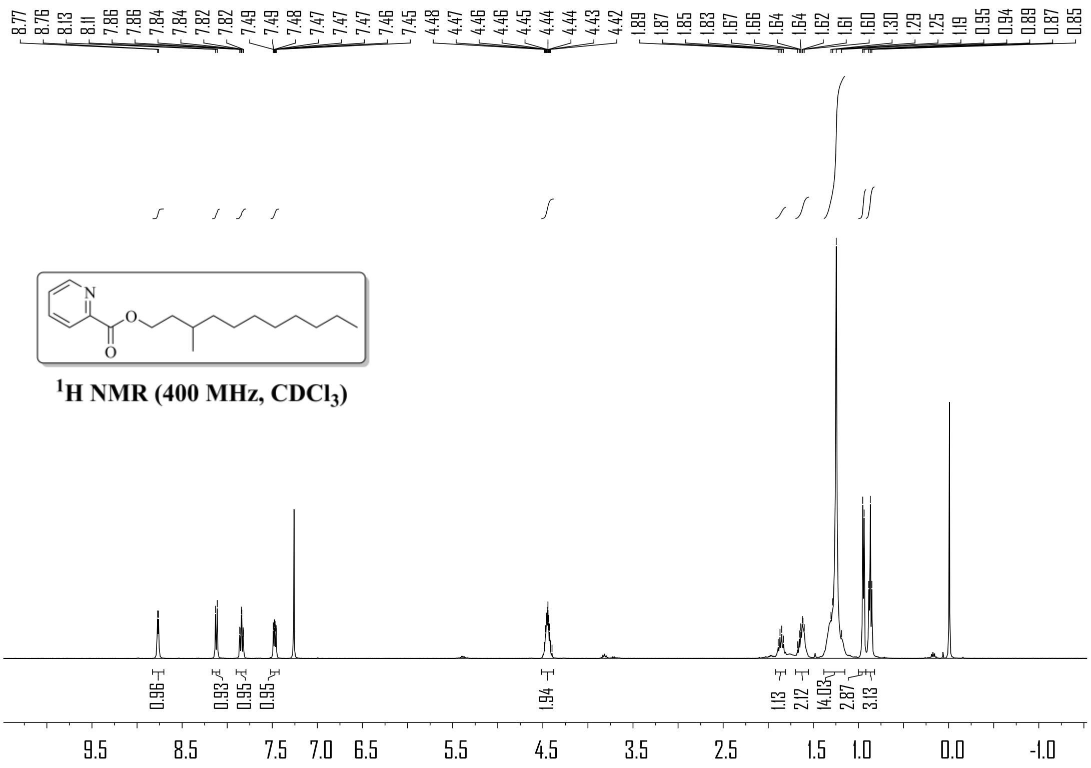

Supplementary Figure 53. ^{1}**H NMR spectra for compound 19**

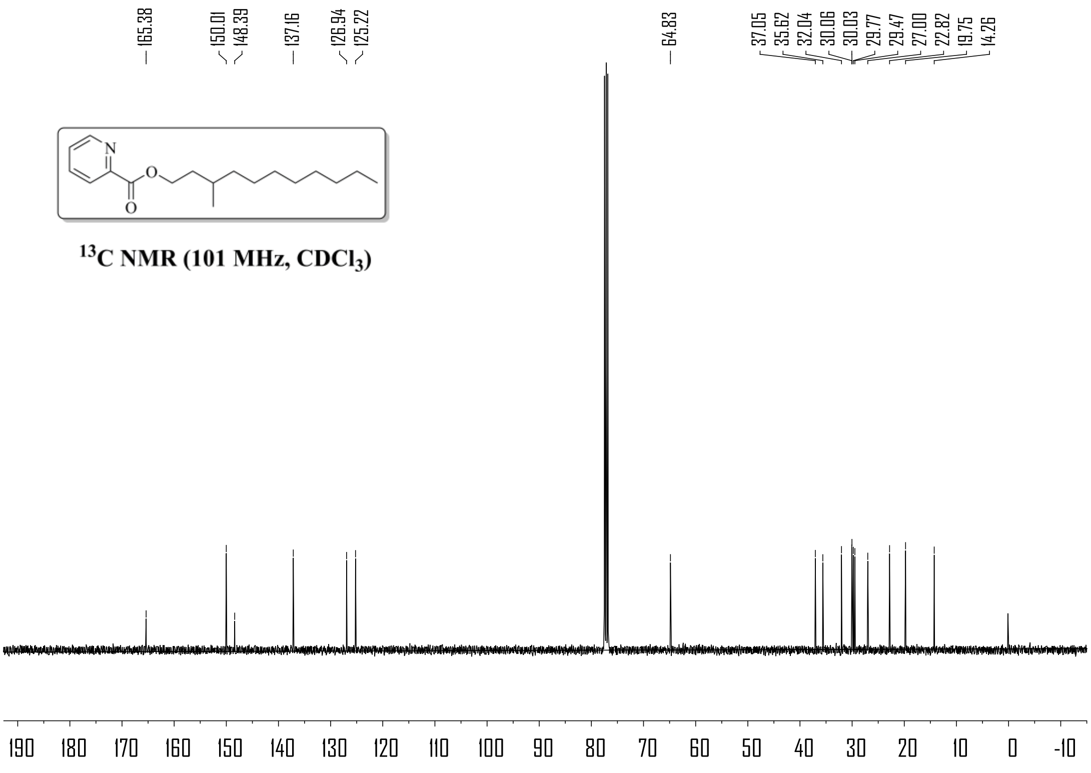

Supplementary Figure 54. ^{13}**C NMR spectra for compound 19**

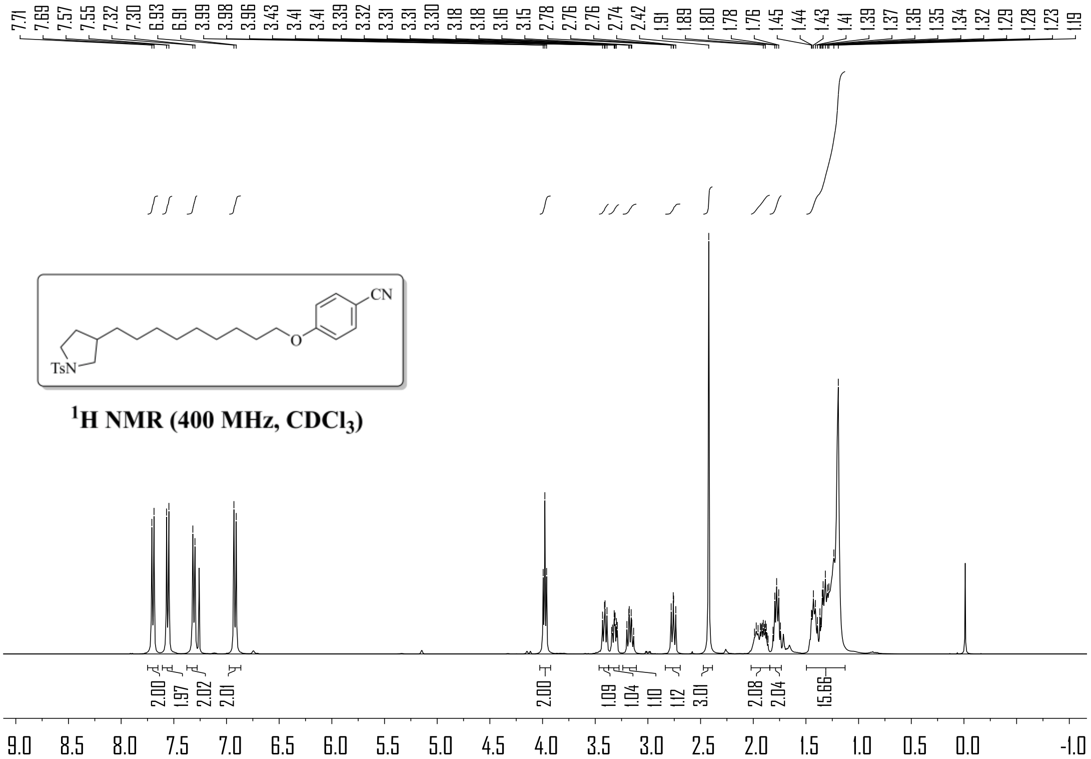

Supplementary Figure 55. ^{1}H NMR spectra for compound 20

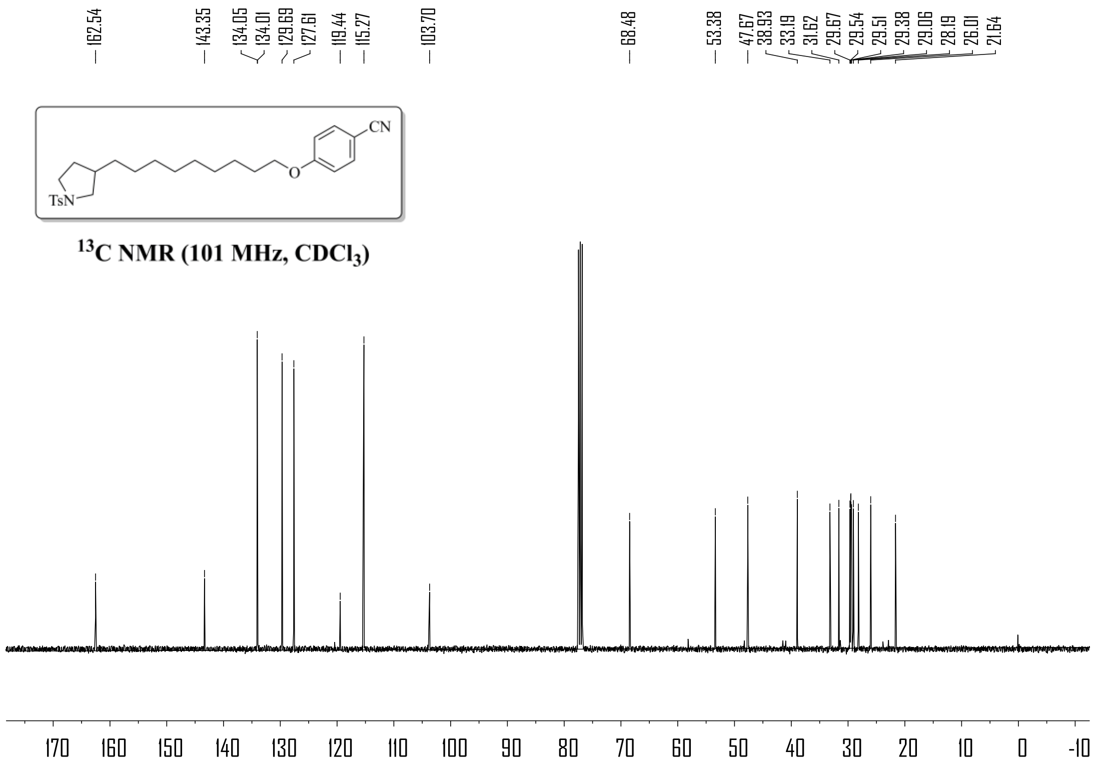

Supplementary Figure 56. ^{13}C NMR spectra for compound 20

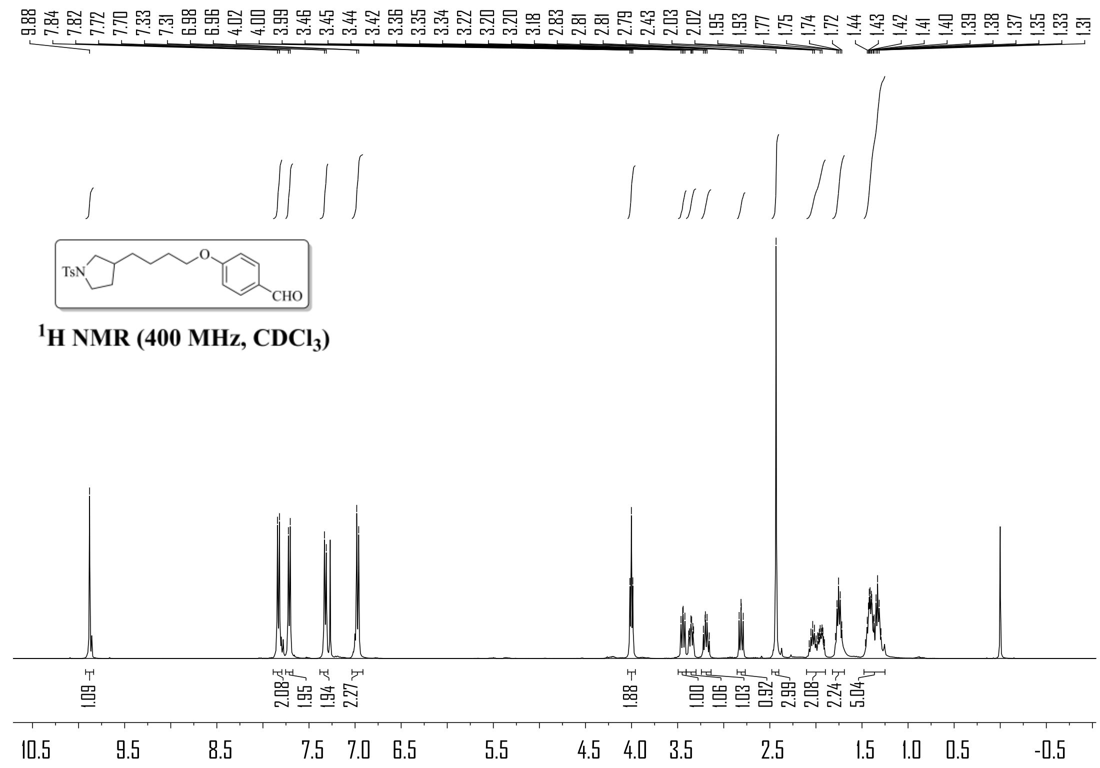

Supplementary Figure 57. ^{1}H NMR spectra for compound 21

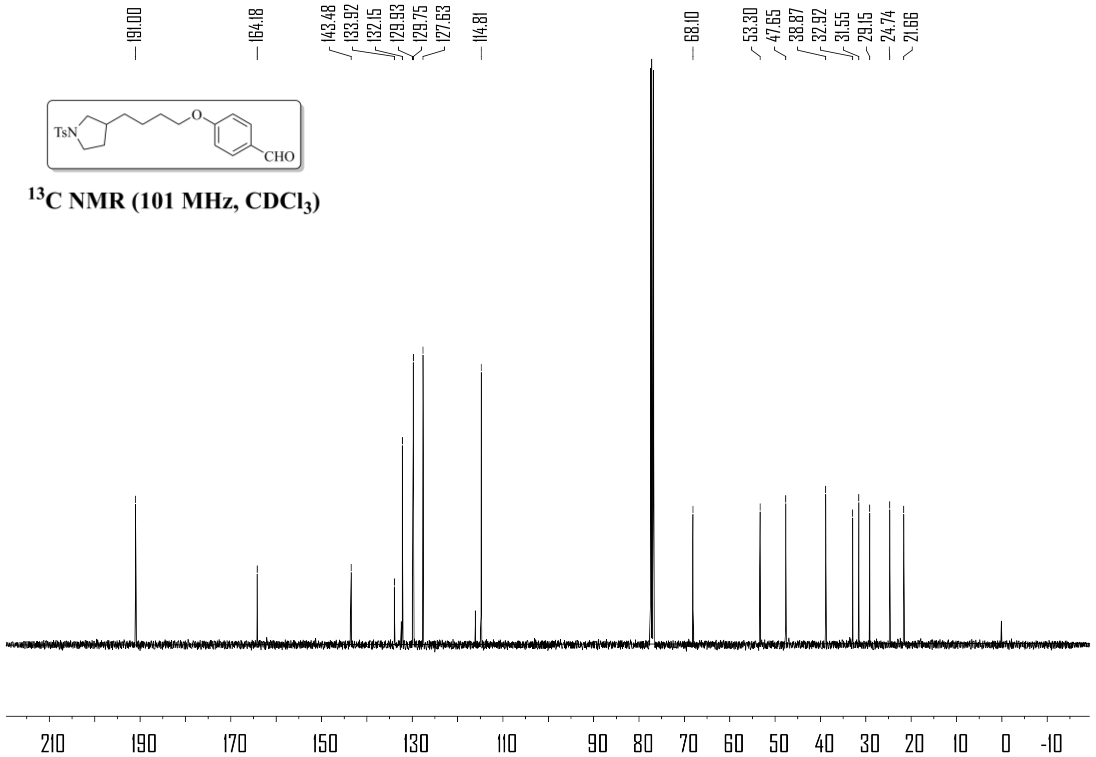

Supplementary Figure 58. ^{13}C NMR spectra for compound 21

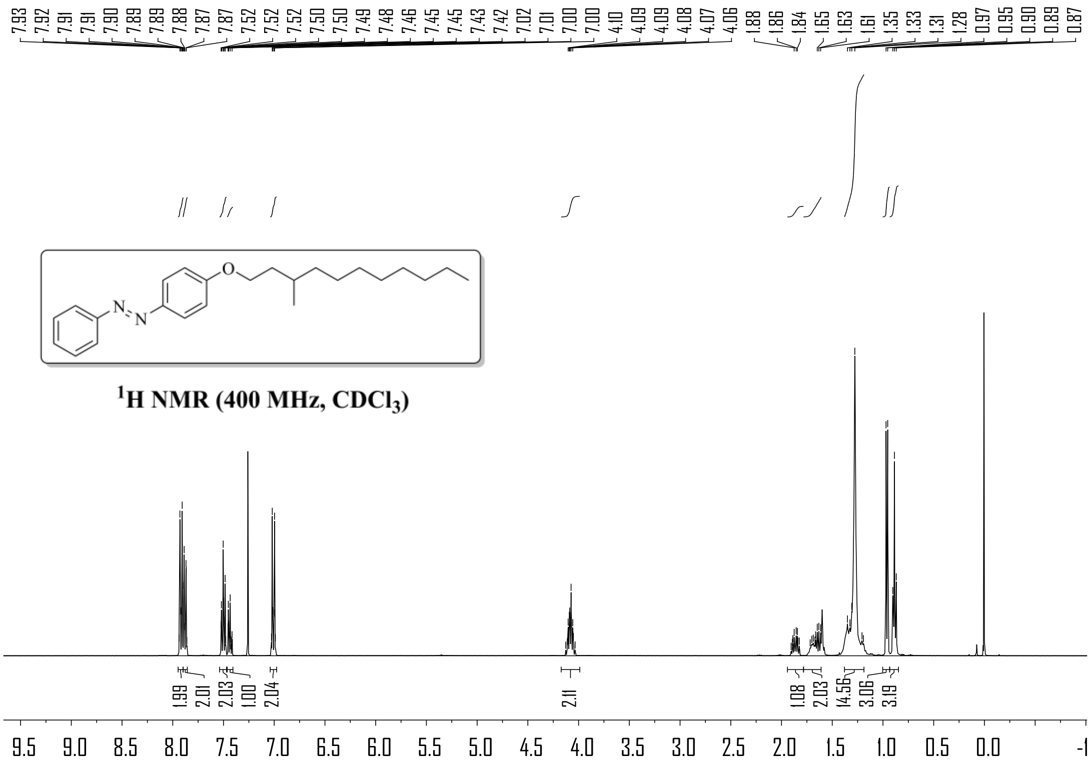

Supplementary Figure 59. ^{1}**H NMR spectra for compound 22**

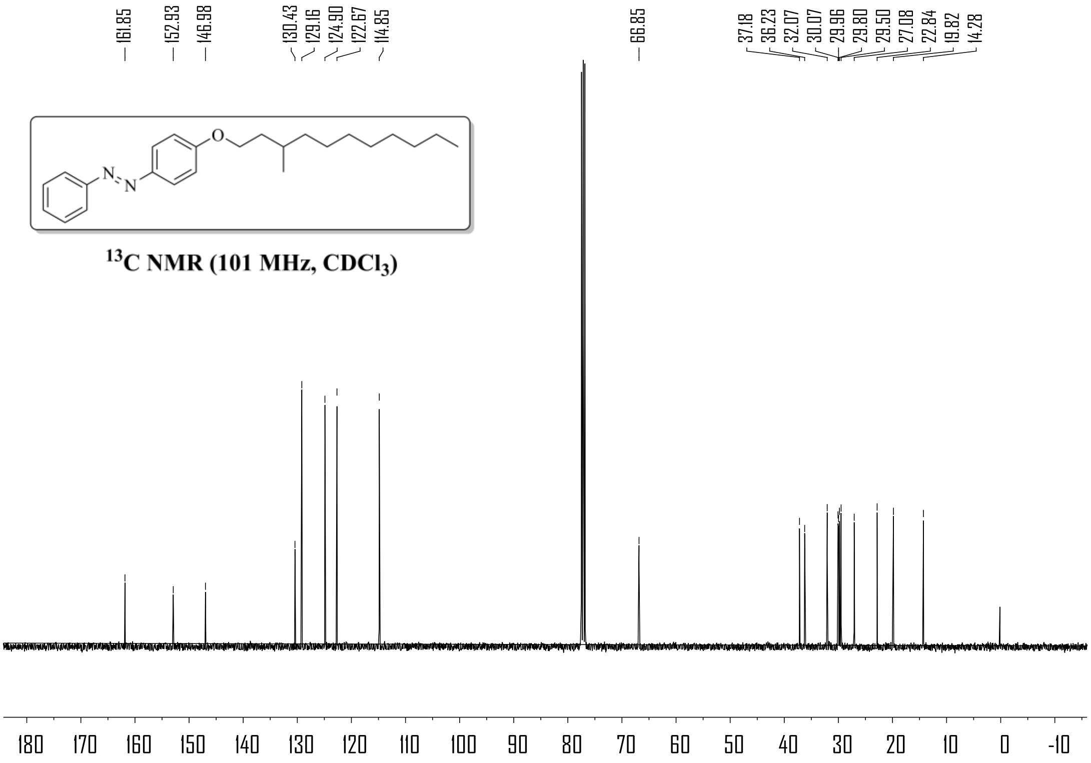

Supplementary Figure 60. ^{13}**C NMR spectra for compound 22**

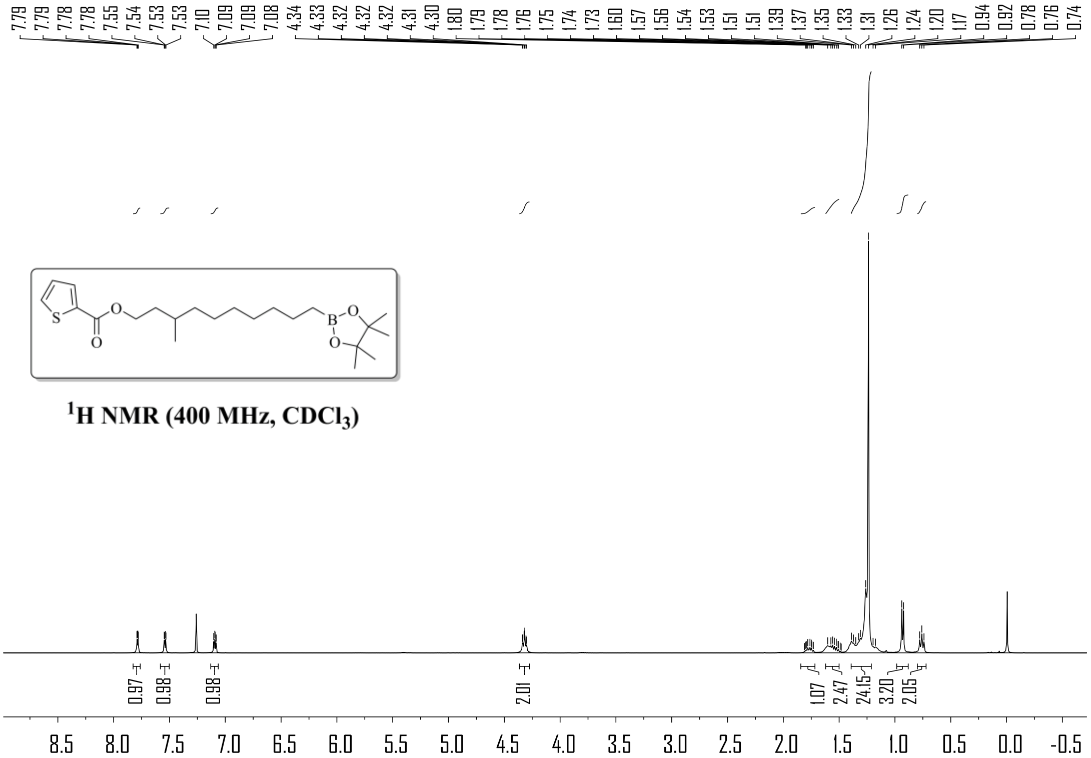

Supplementary Figure 61. ^{1}H NMR spectra for compound 23

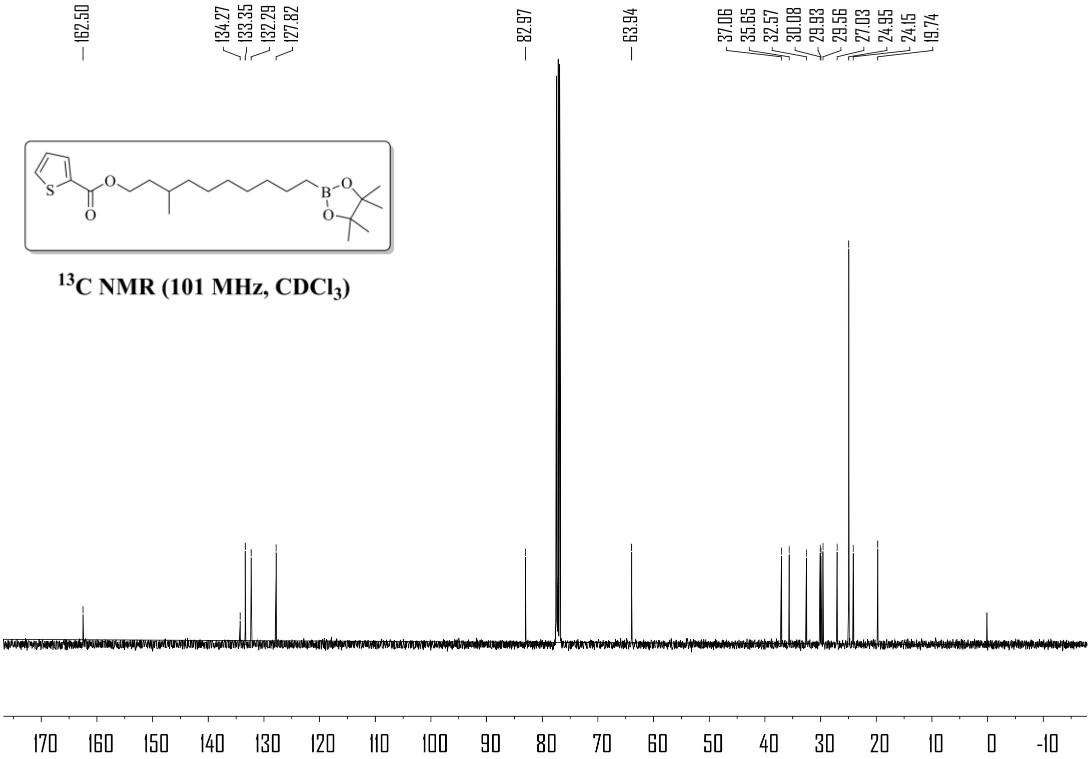

Supplementary Figure 62. ^{13}C NMR spectra for compound 23

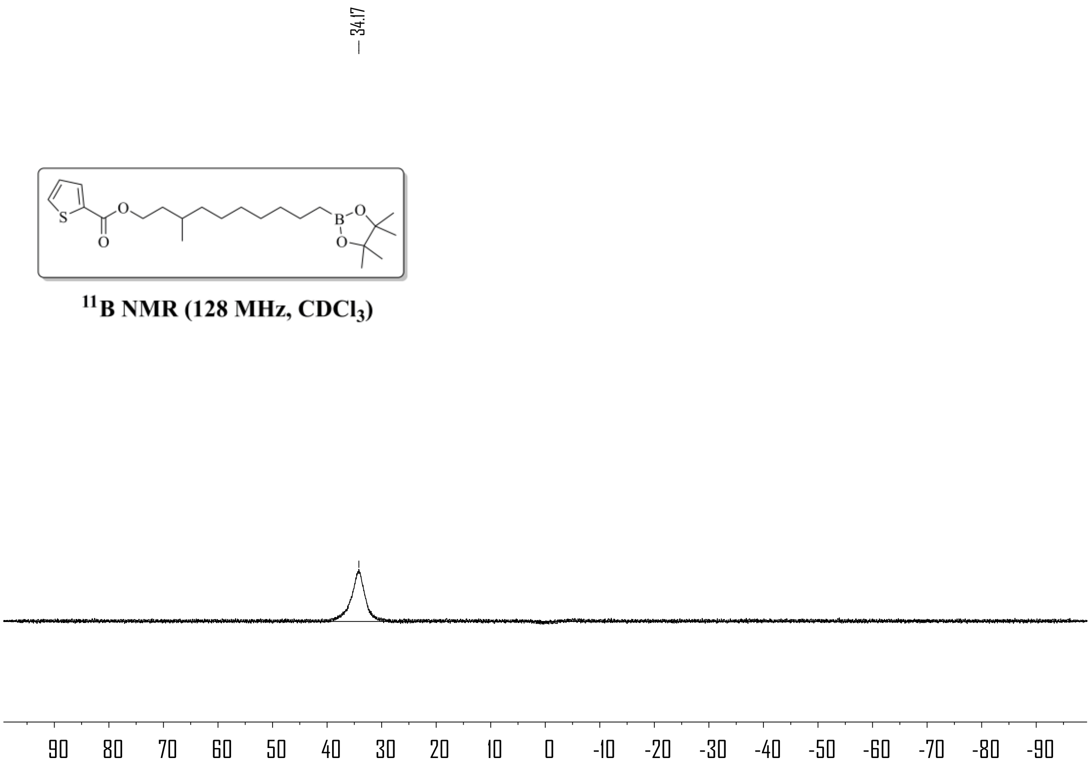

Supplementary Figure 63. ^{11}B NMR spectra for compound 23

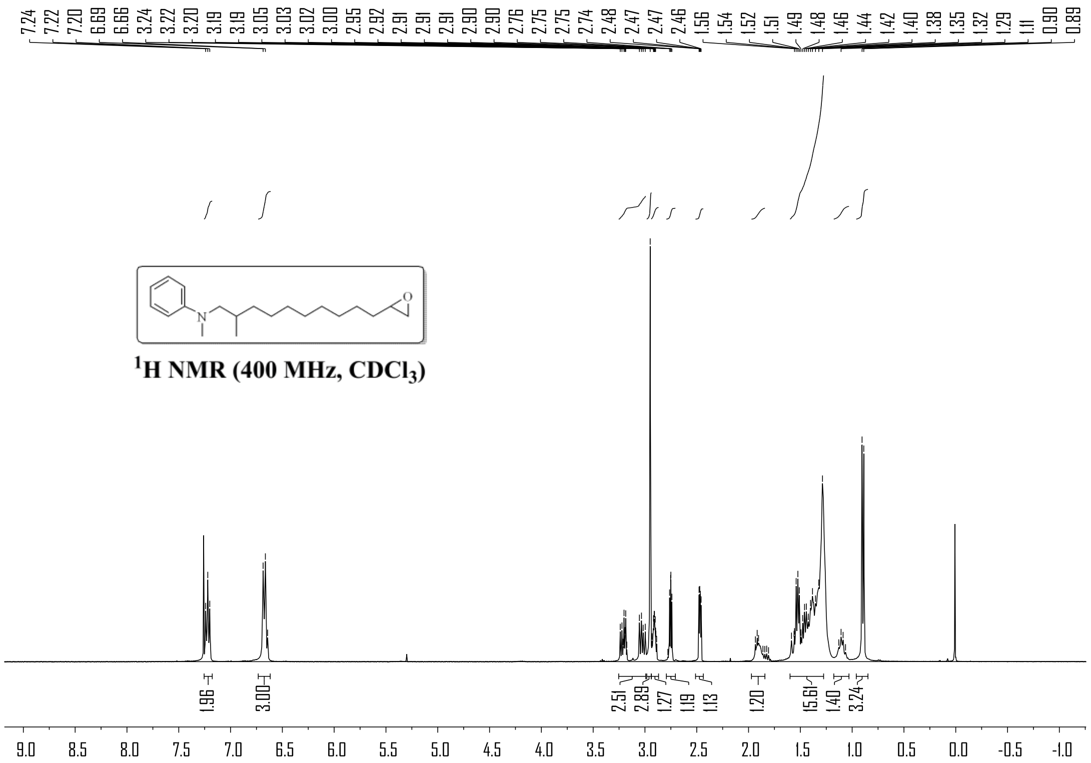

Supplementary Figure 64. ^{1}H NMR spectra for compound 24

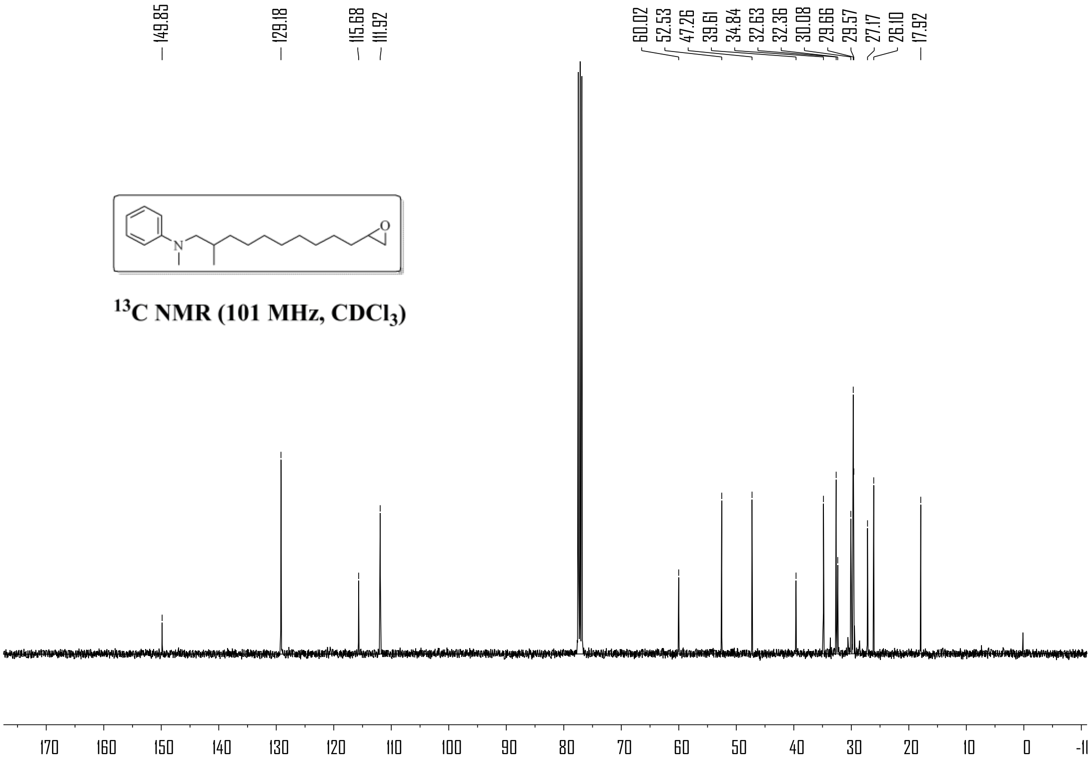

Supplementary Figure 65. ^{13}C NMR spectra for compound 24

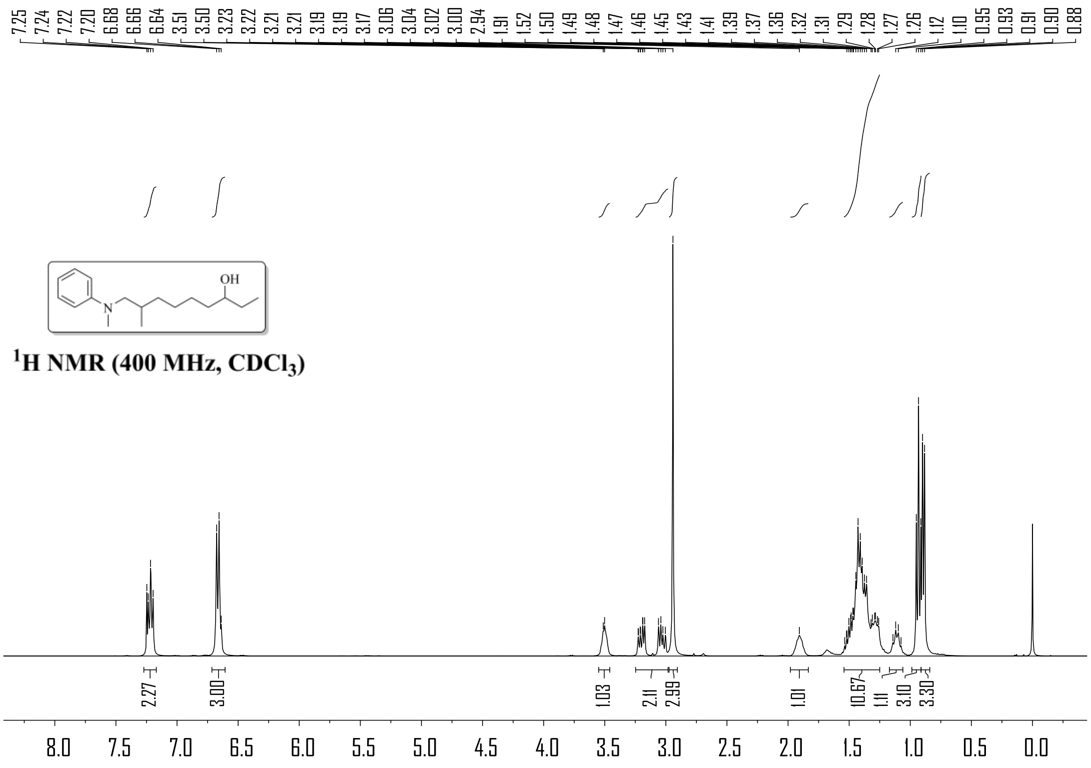

Supplementary Figure 66. ^{1}H NMR spectra for compound 25

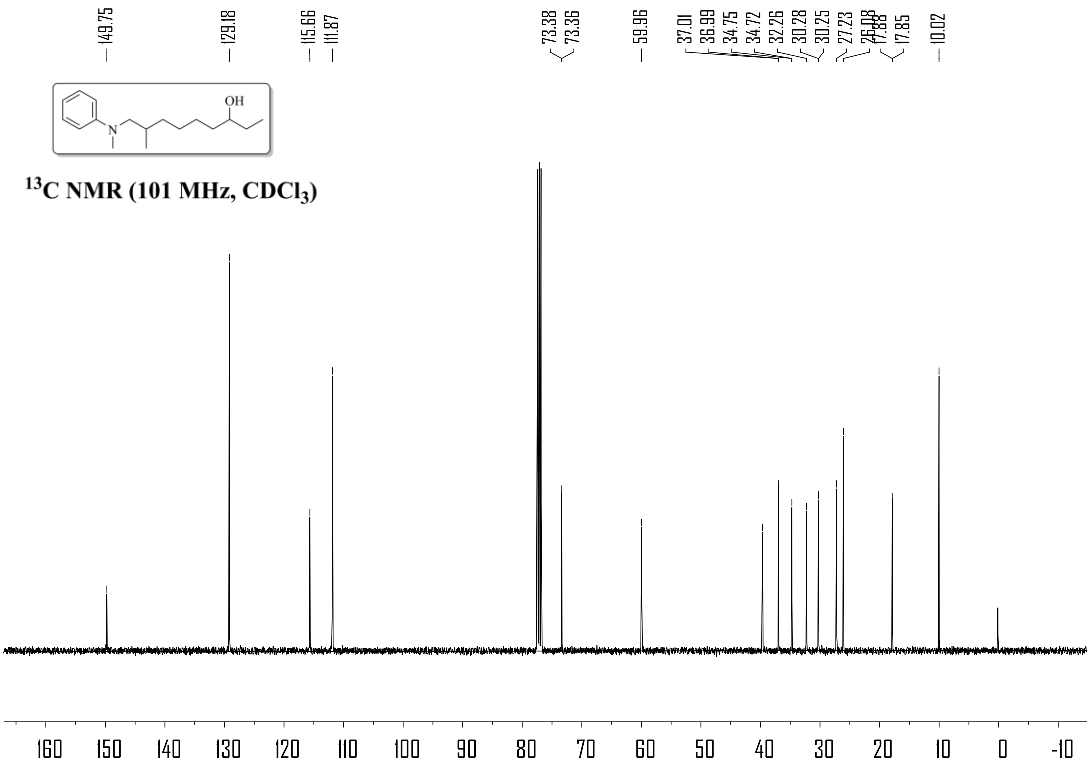

Supplementary Figure 67. ^{13}C NMR spectra for compound 25

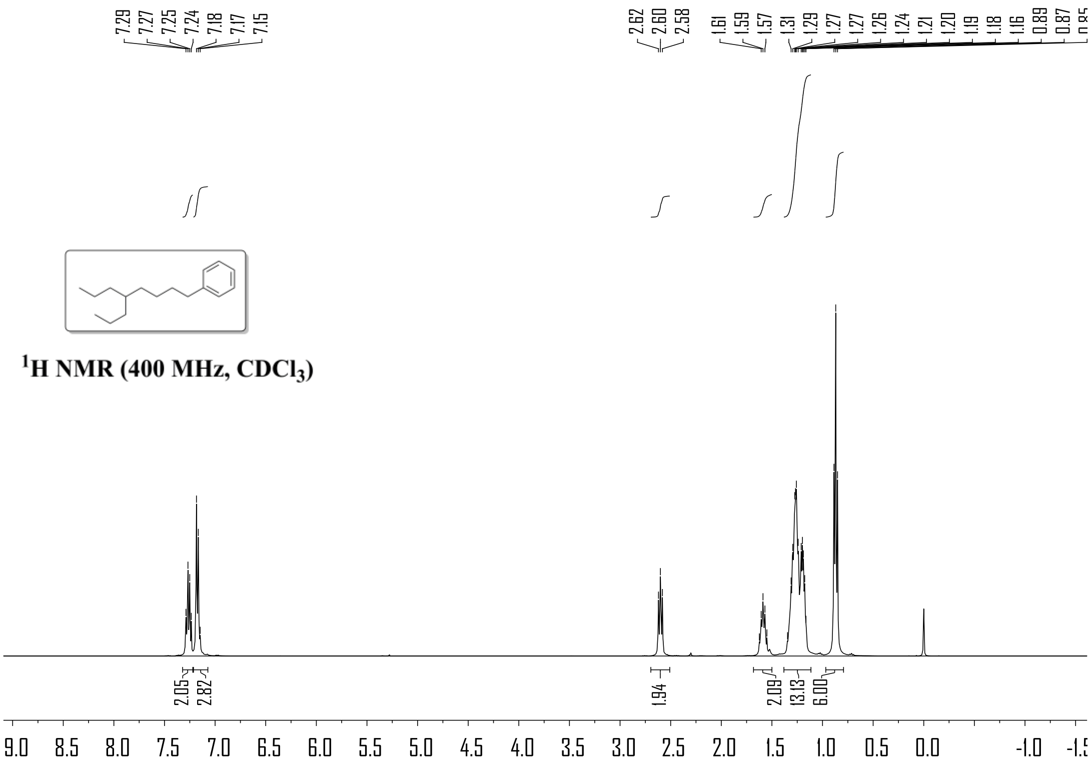

Supplementary Figure 68. ^{1}H NMR spectra for compound 26

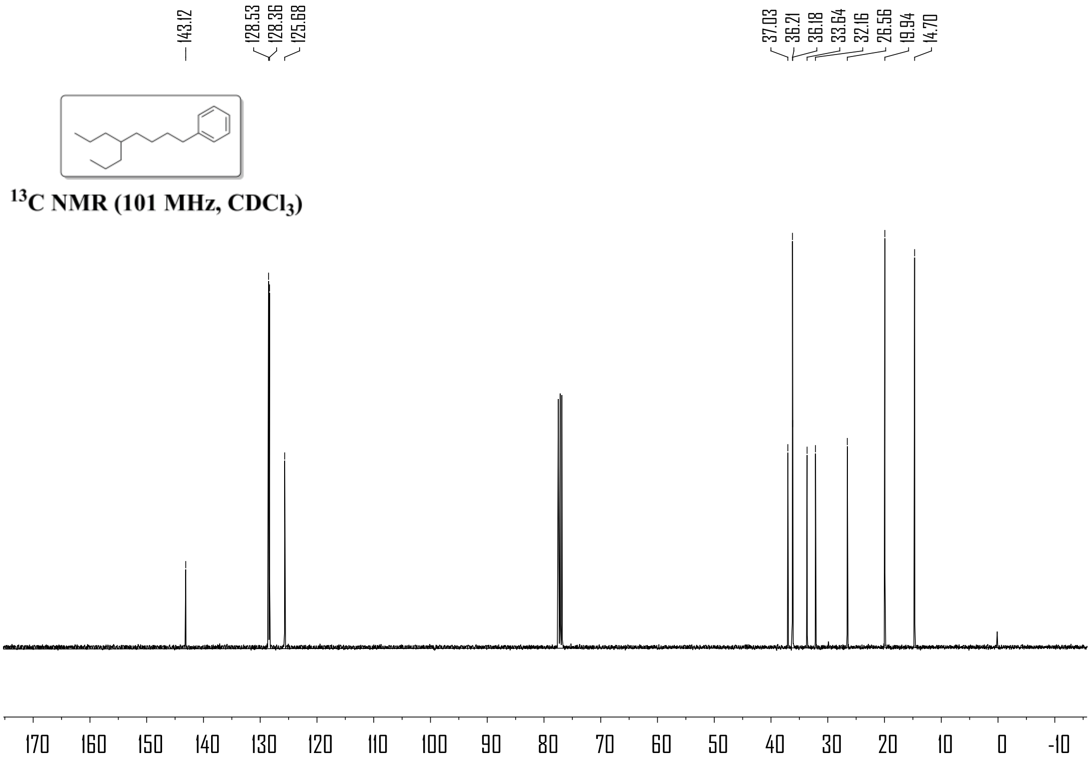

Supplementary Figure 69. ^{13}C NMR spectra for compound 26

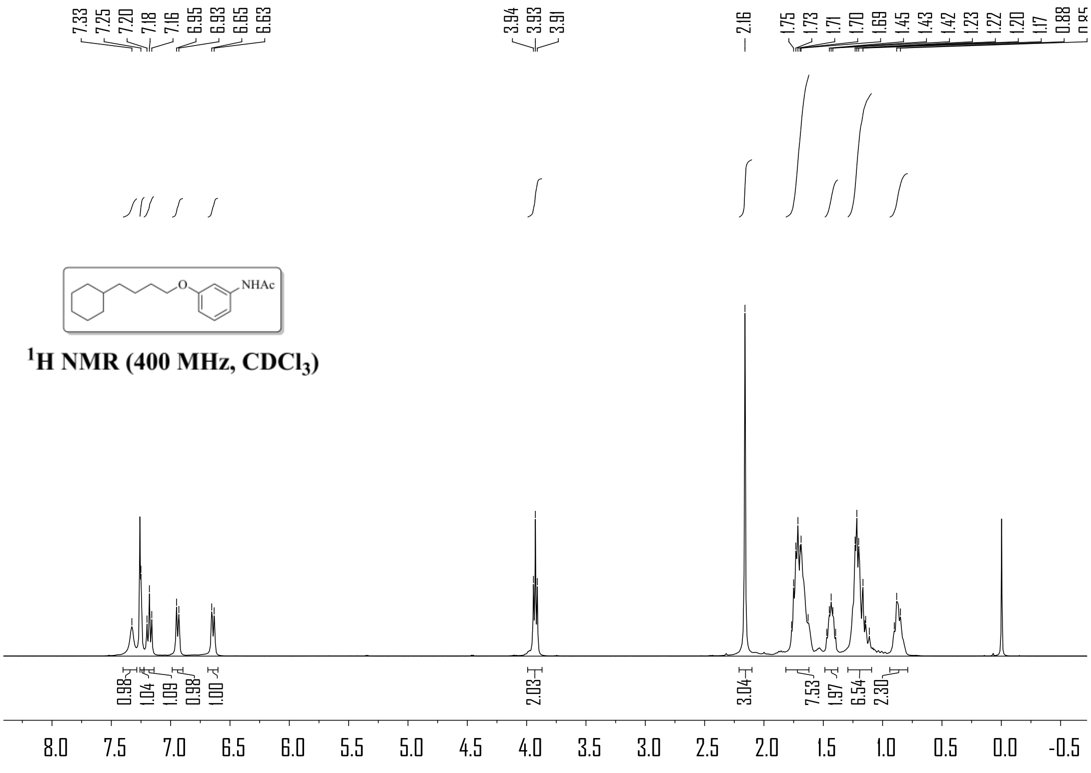

Supplementary Figure 70. ^{1}H NMR spectra for compound 27

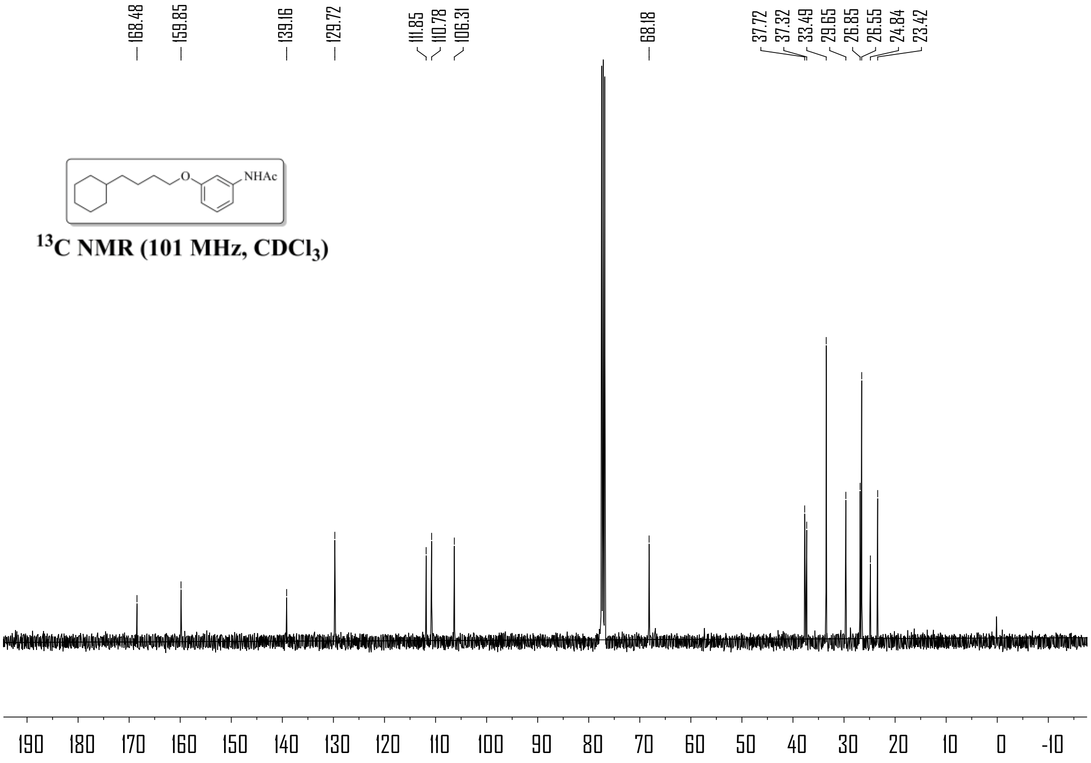

Supplementary Figure 71. ^{13}C NMR spectra for compound 27

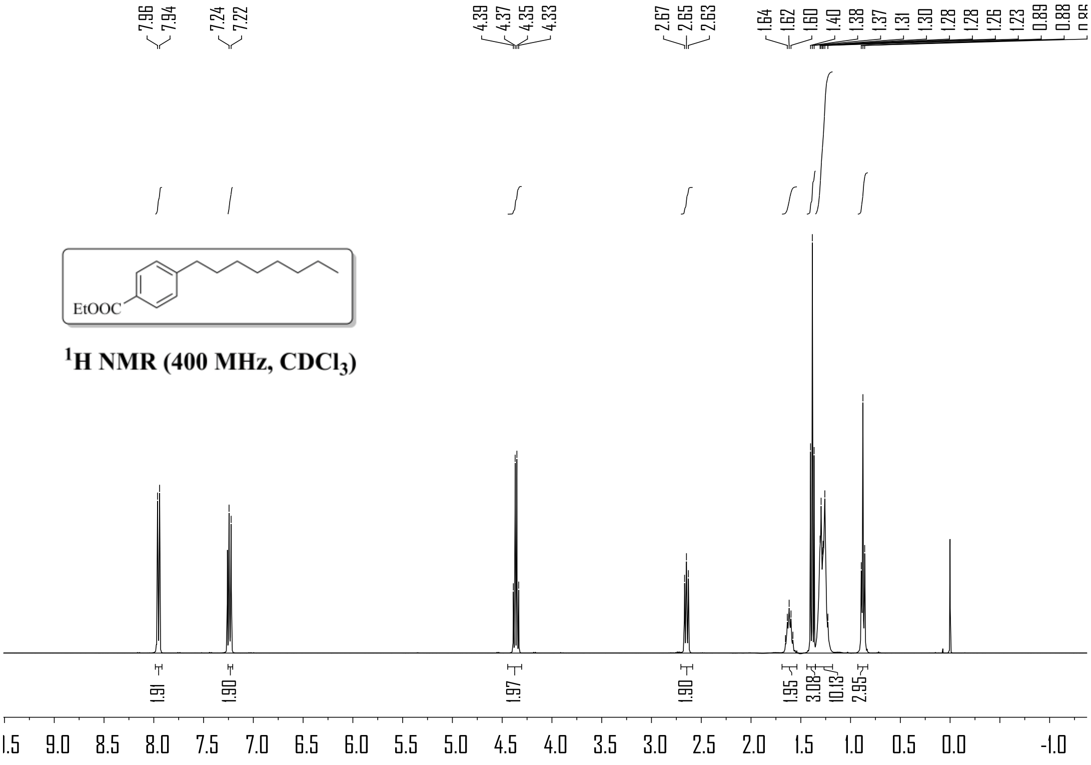

Supplementary Figure 72. ^{1}H NMR spectra for compound 28

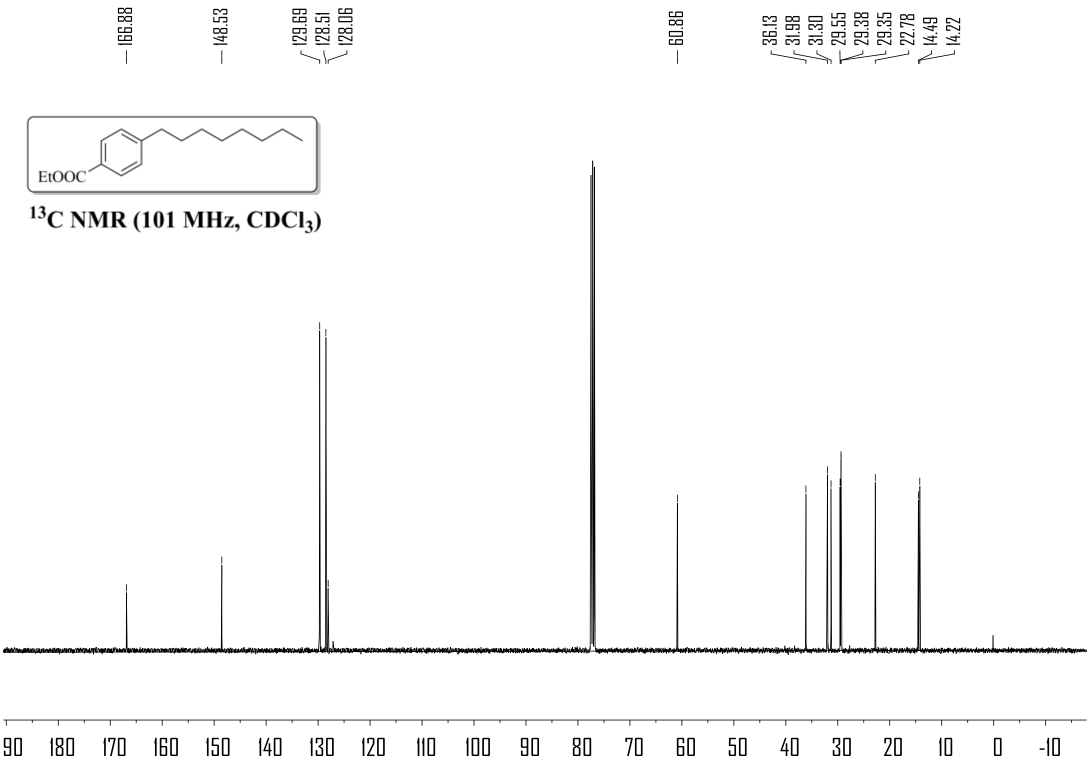

Supplementary Figure 73. ^{13}C NMR spectra for compound 28

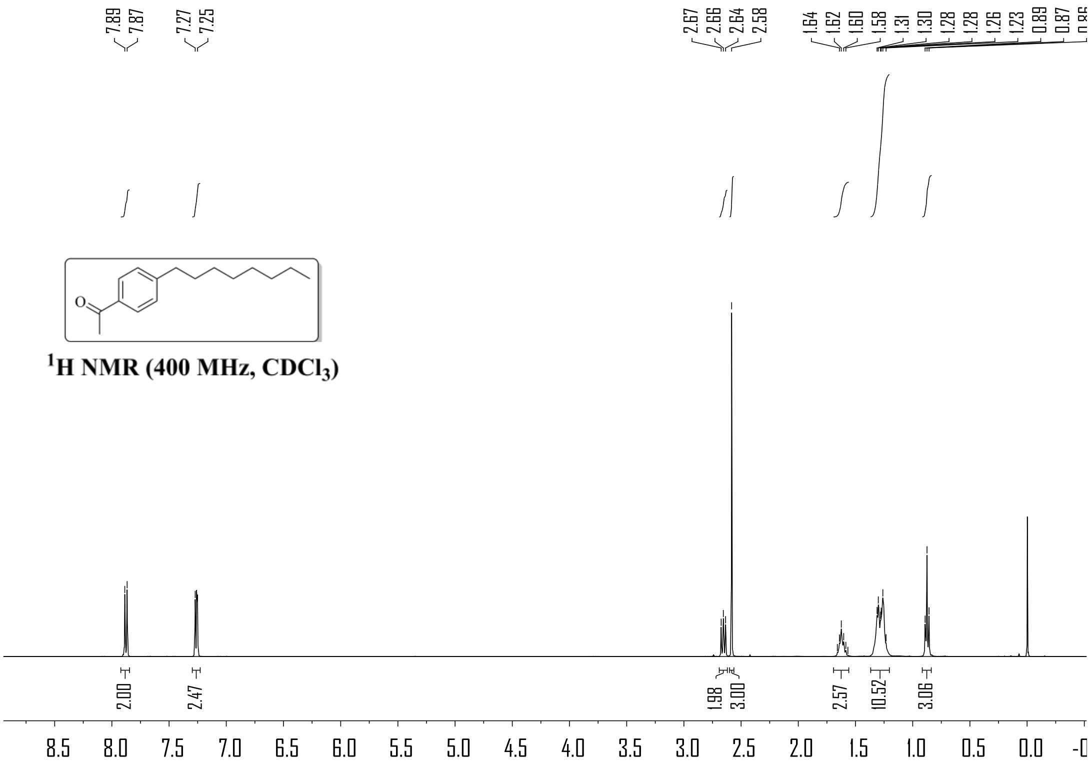

Supplementary Figure 74. ^{1}H NMR spectra for compound 29

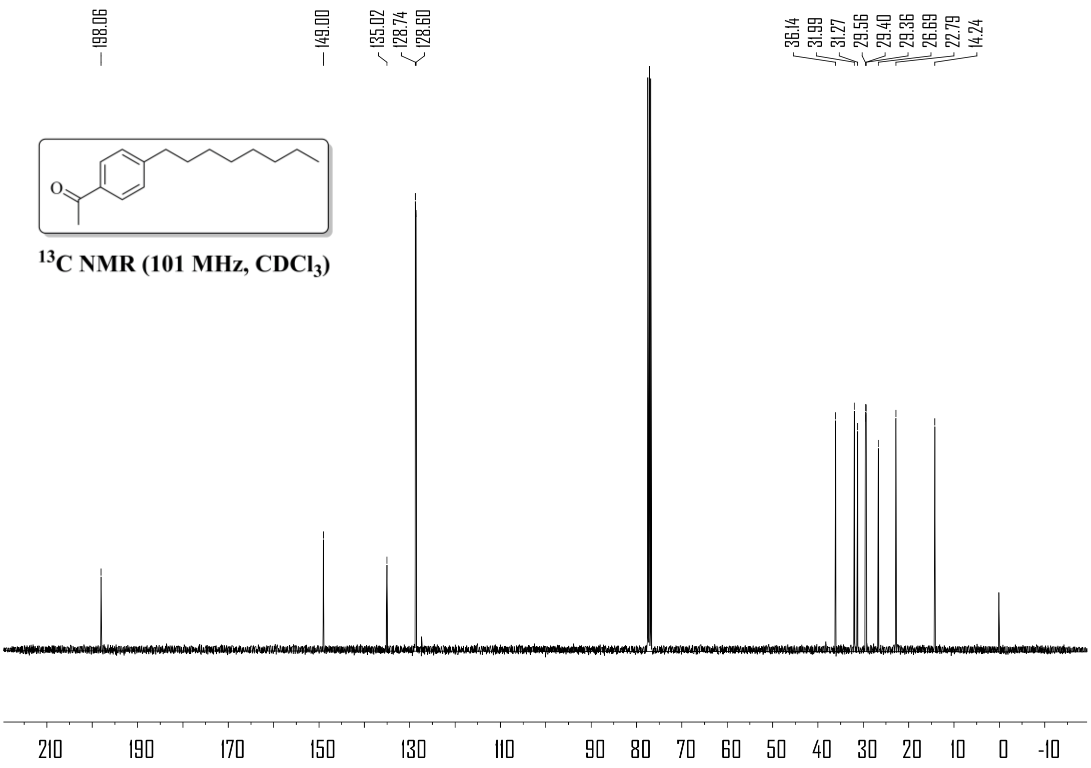

Supplementary Figure 75. ^{13}C NMR spectra for compound 29

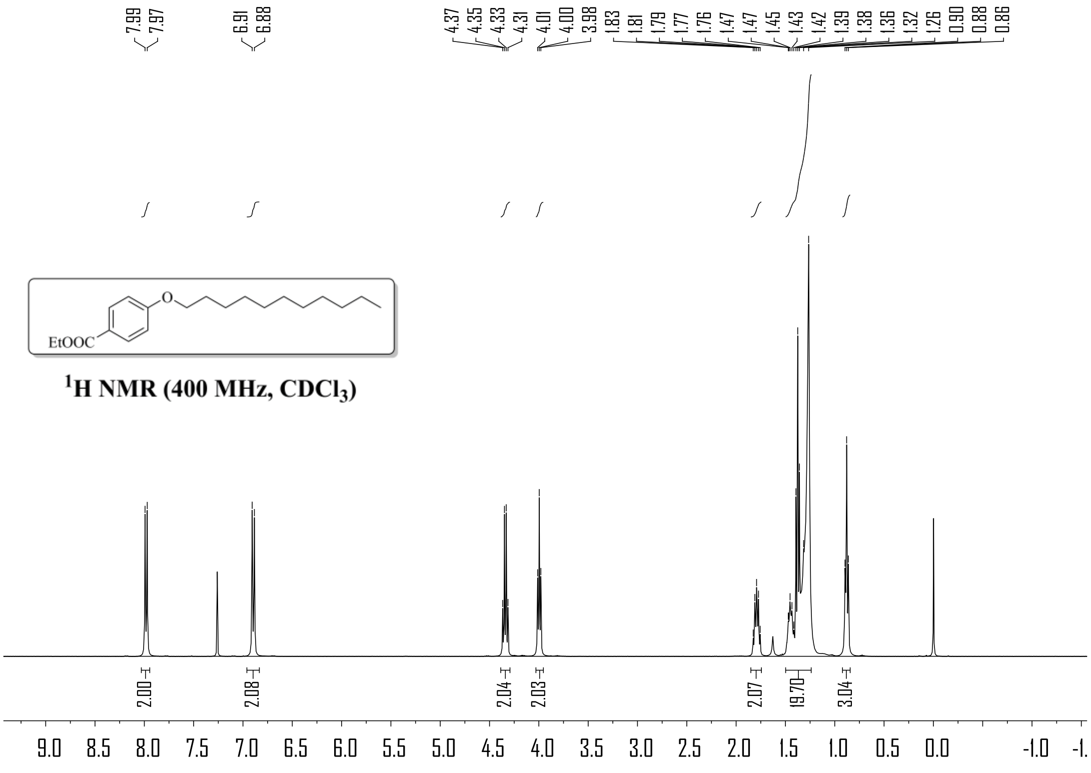

Supplementary Figure 76. ^{1}H NMR spectra for compound 30

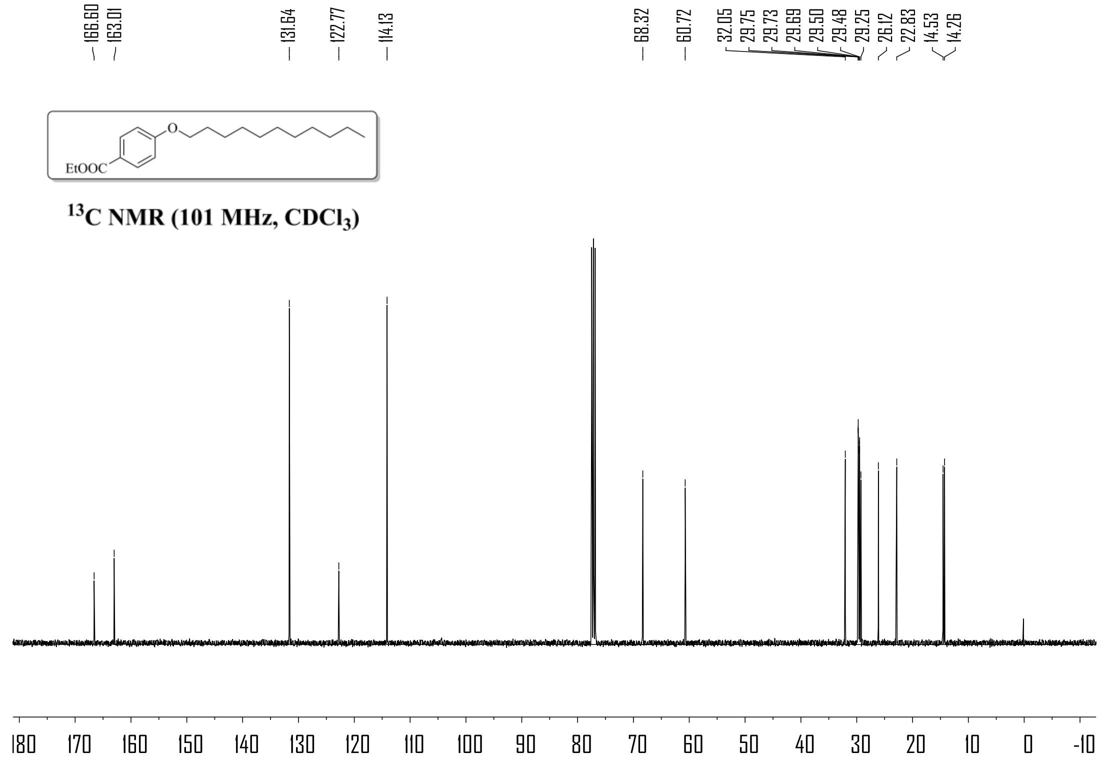

Supplementary Figure 77. ^{13}C NMR spectra for compound 30

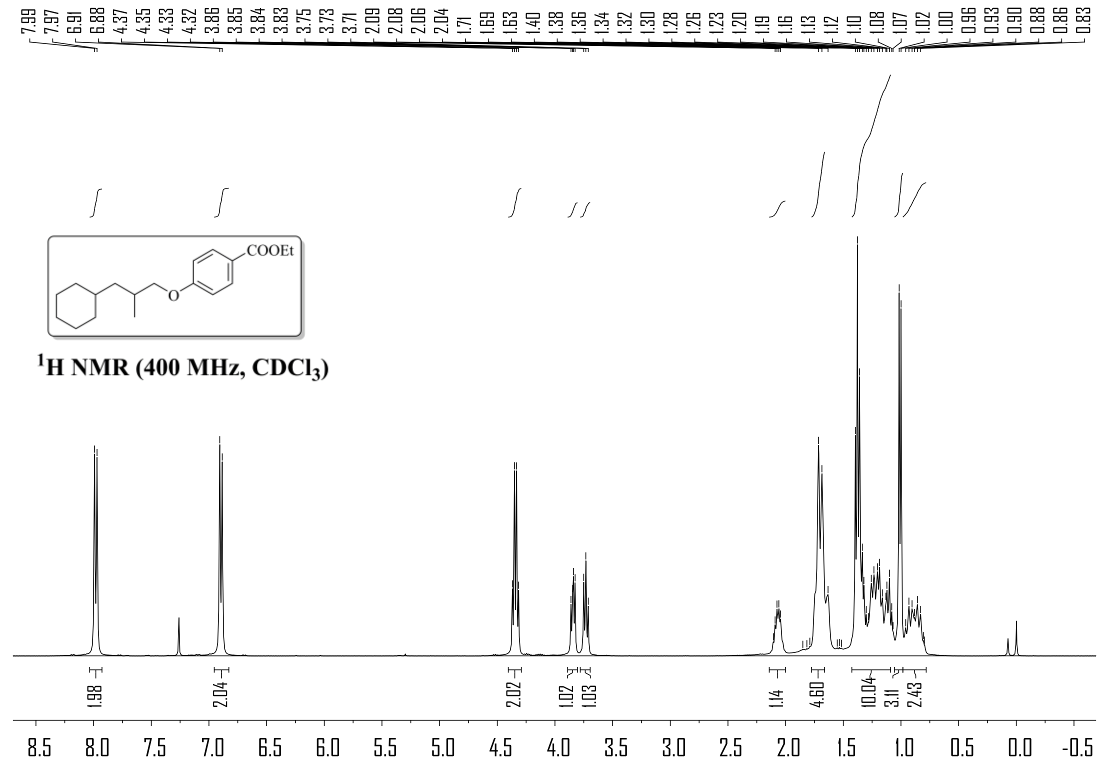

Supplementary Figure 78. ^{1}H NMR spectra for compound 31

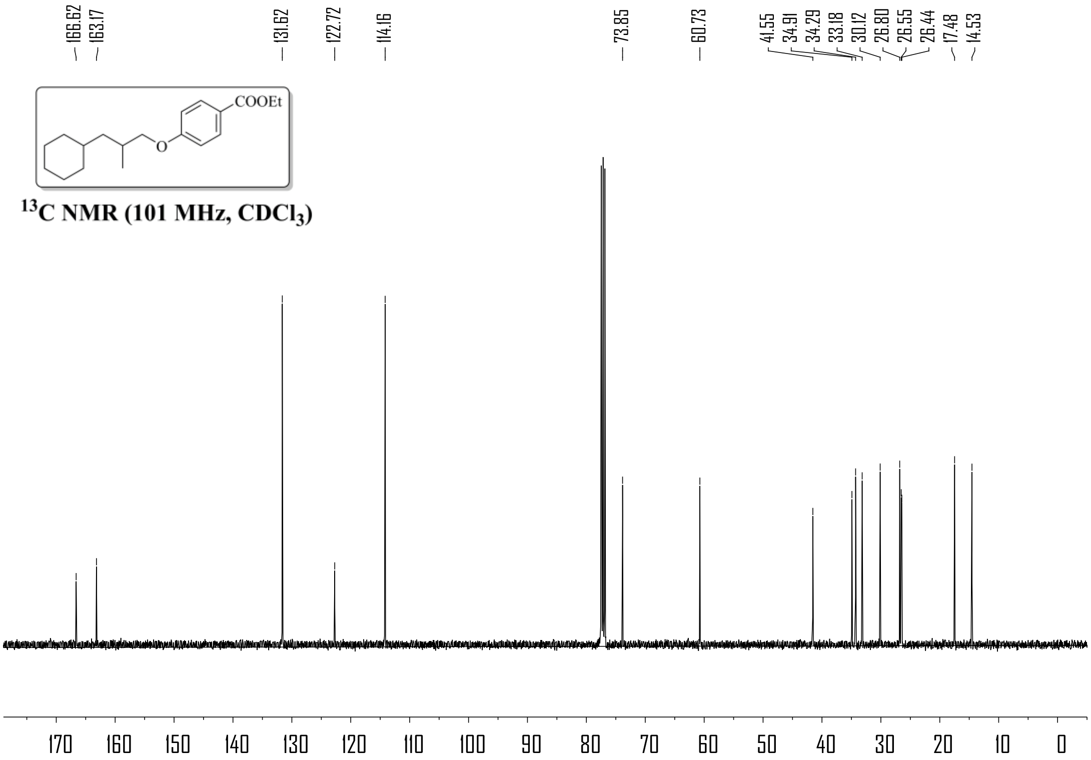

Supplementary Figure 79. ^{13}C NMR spectra for compound 31

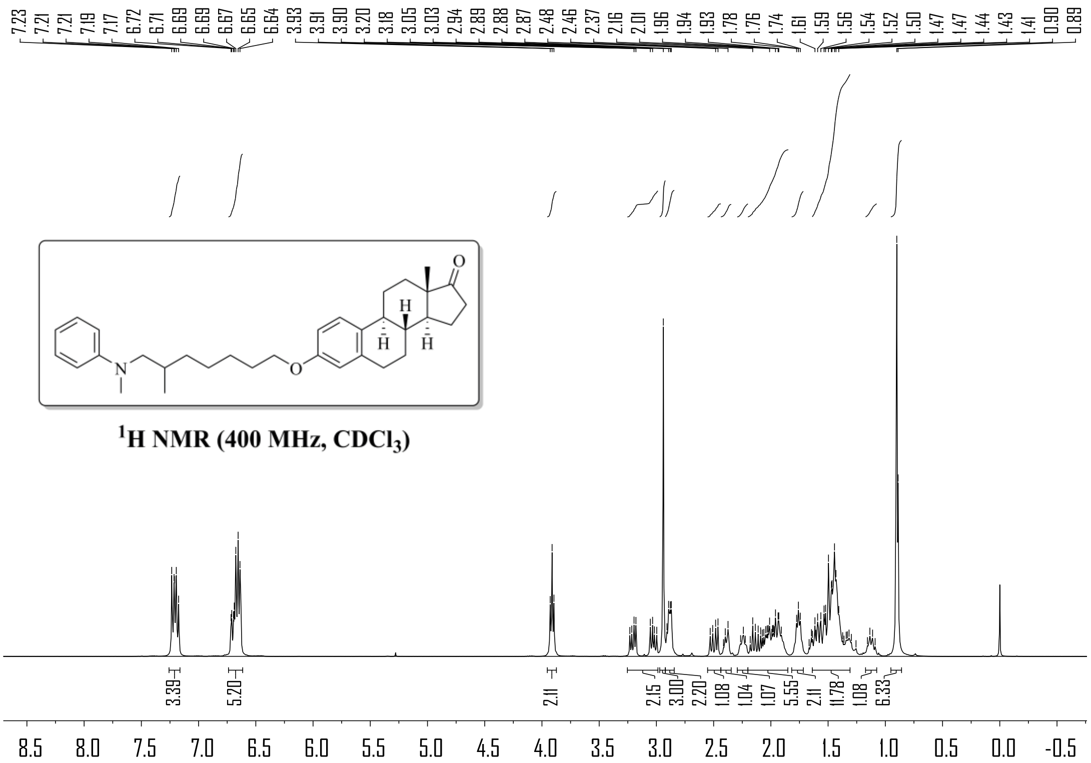

Supplementary Figure 80. ^{1}H NMR spectra for compound 32

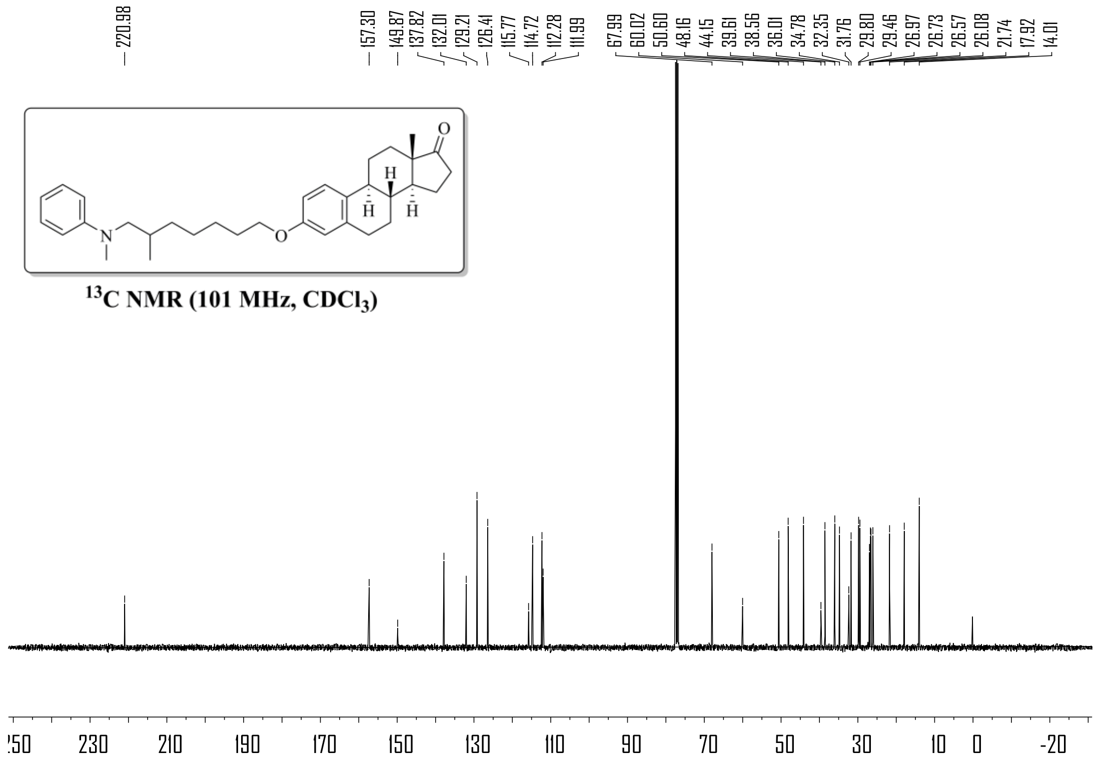

Supplementary Figure 81. ^{13}C NMR spectra for compound 32

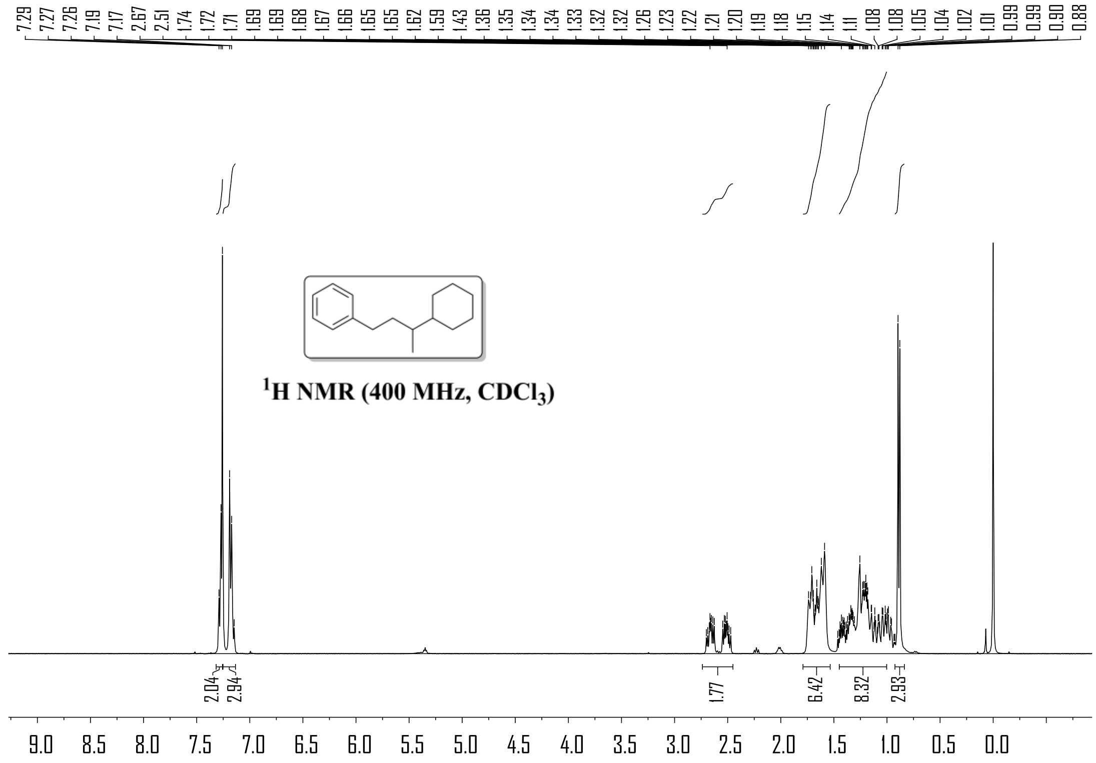

Supplementary Figure 82. ^{1}H NMR spectra for compound 33

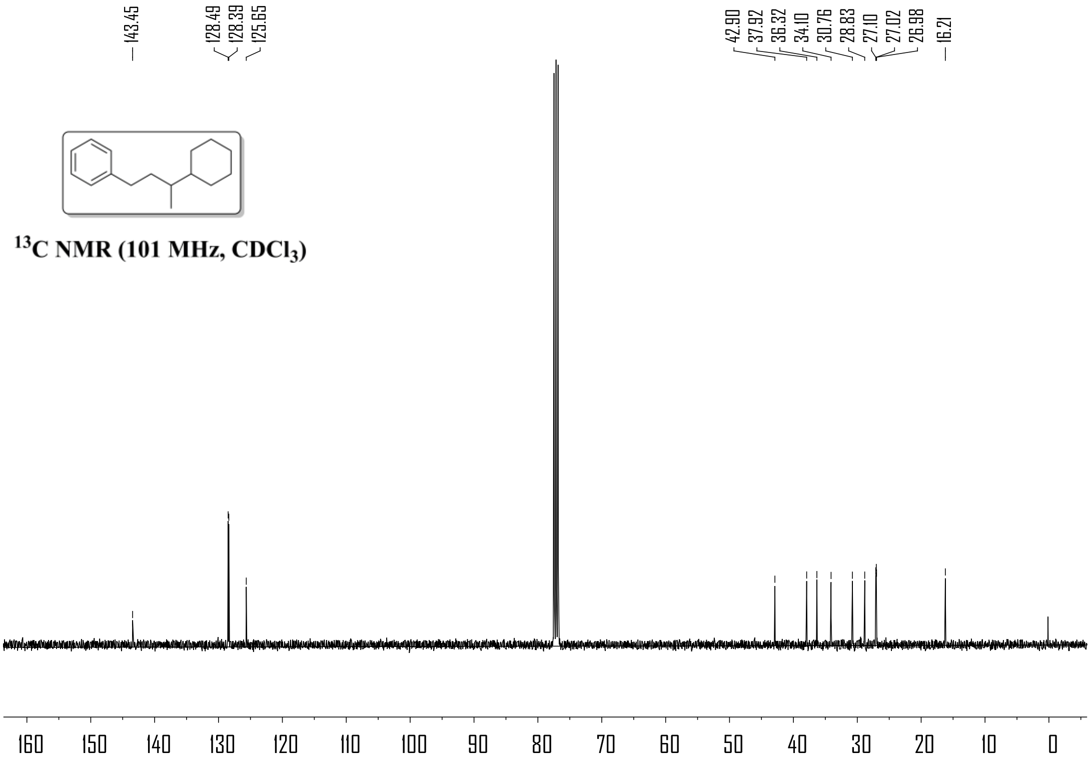

Supplementary Figure 83. **^{13}C NMR spectra for compound 33**

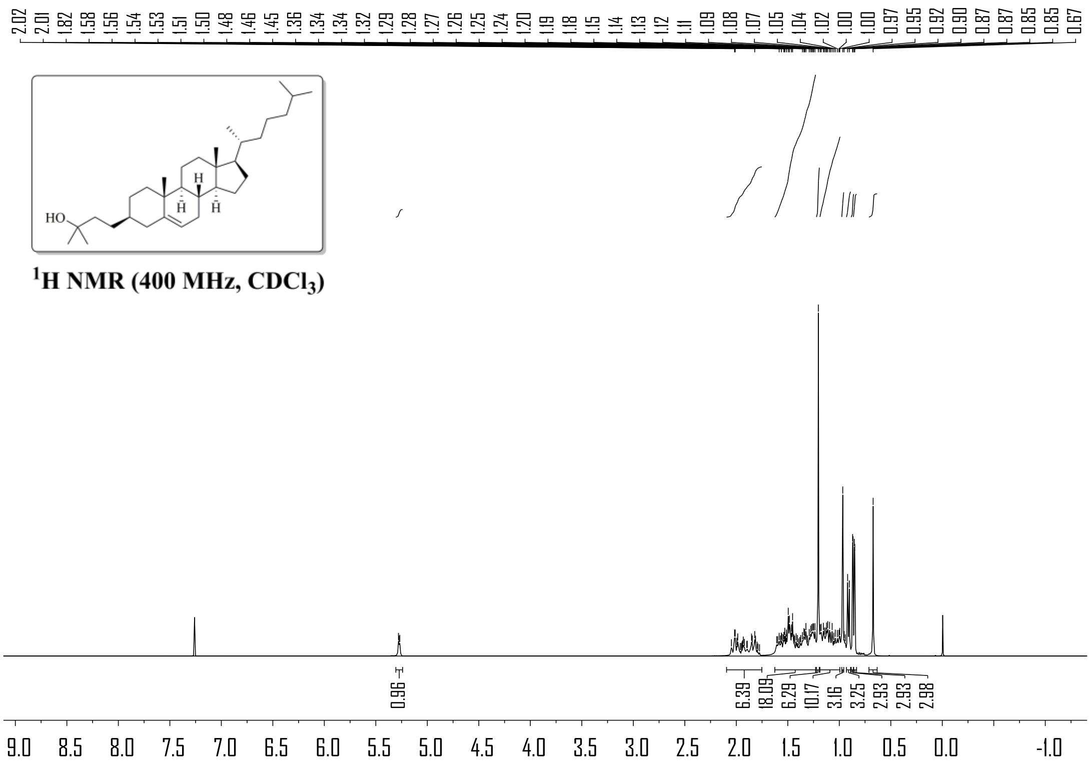

Supplementary Figure 84. **^{1}H NMR spectra for compound 36**

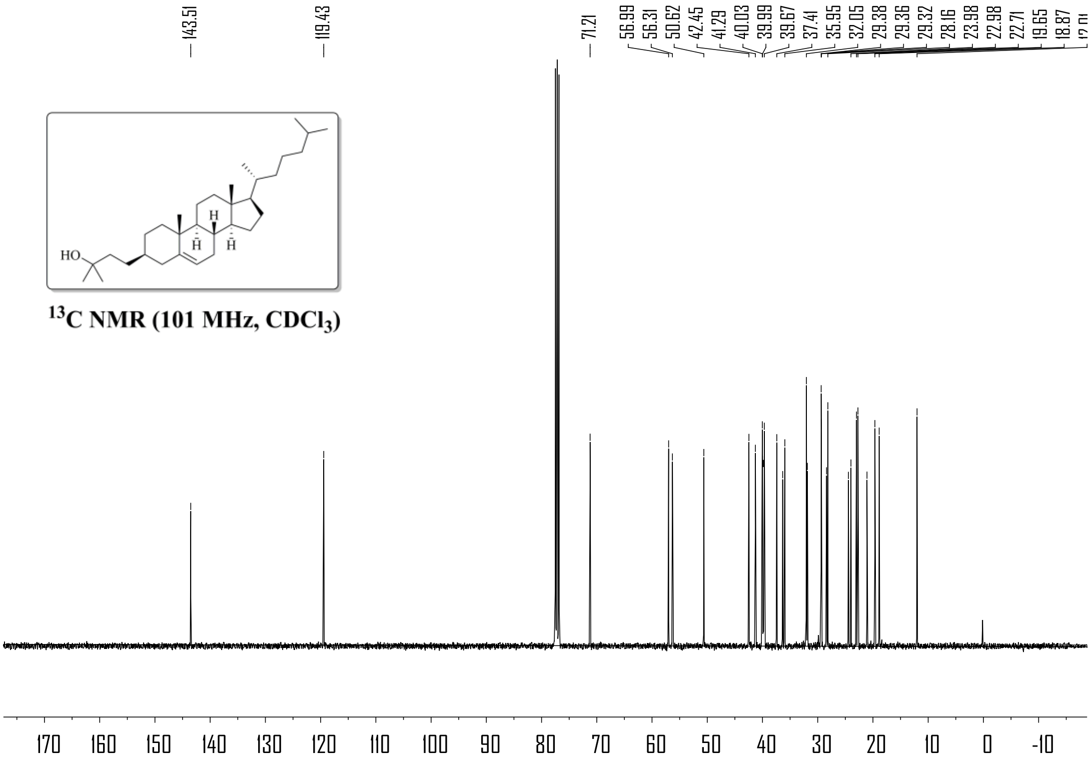

Supplementary Figure 85. ^{13}C NMR spectra for compound 36

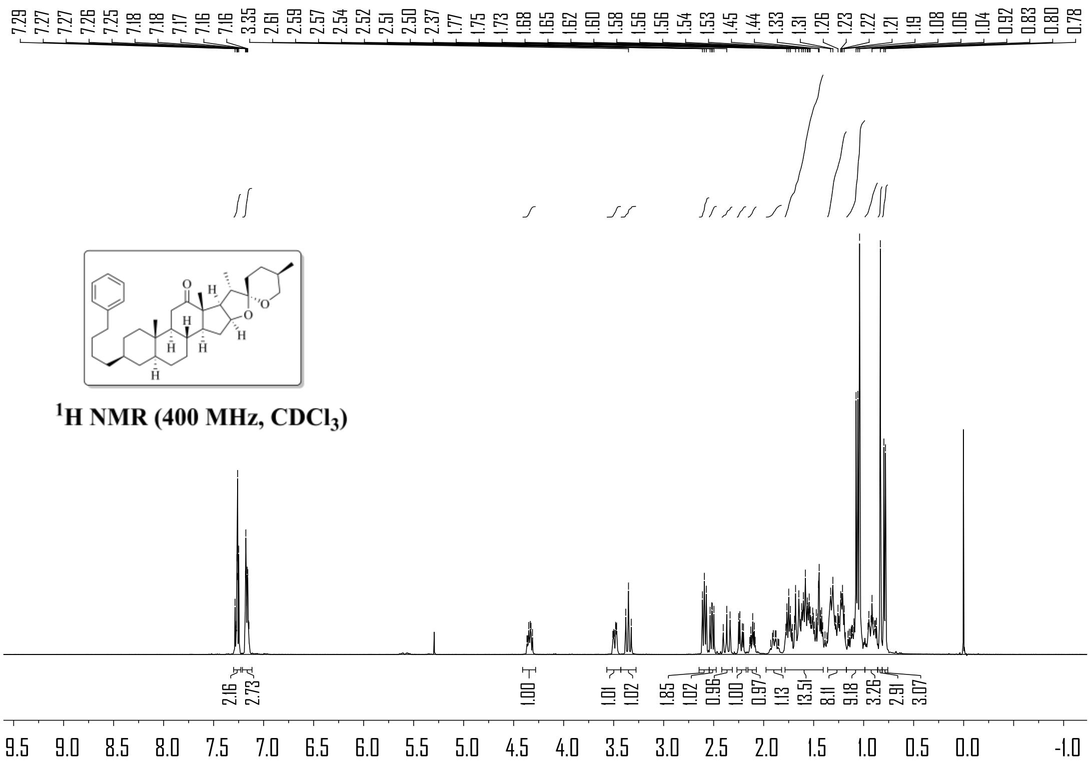

Supplementary Figure 86. ^{1}H NMR spectra for compound 39

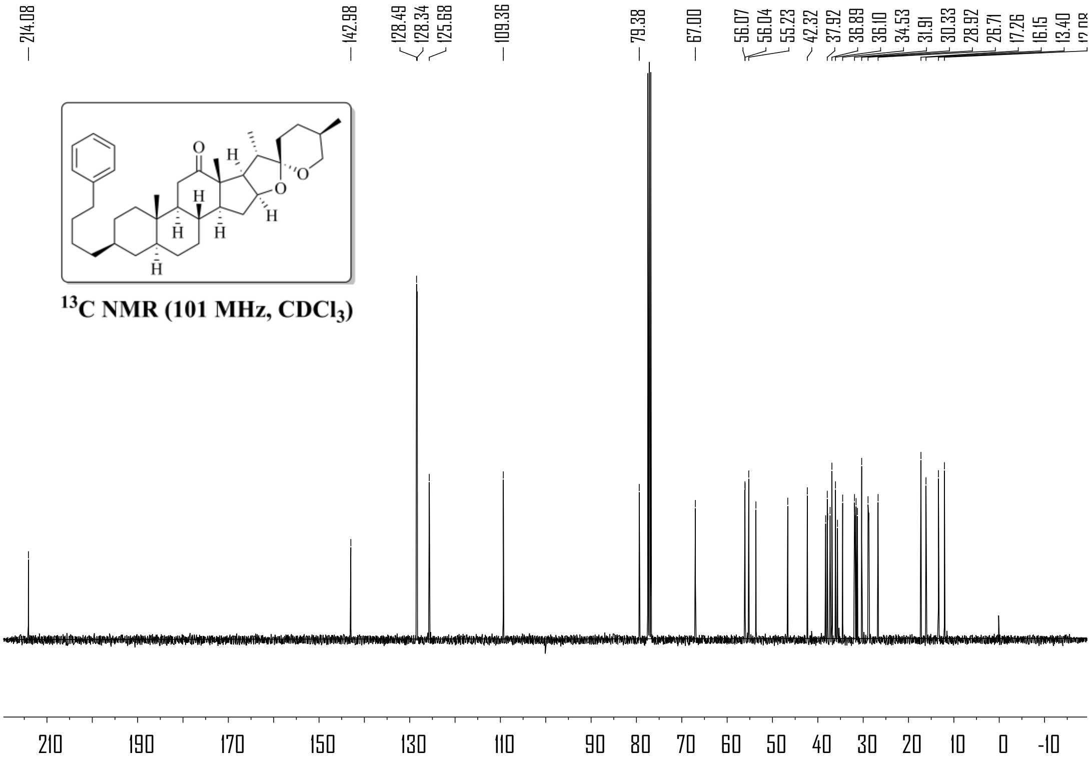

Supplementary Figure 87. ^{13}C NMR spectra for compound 39

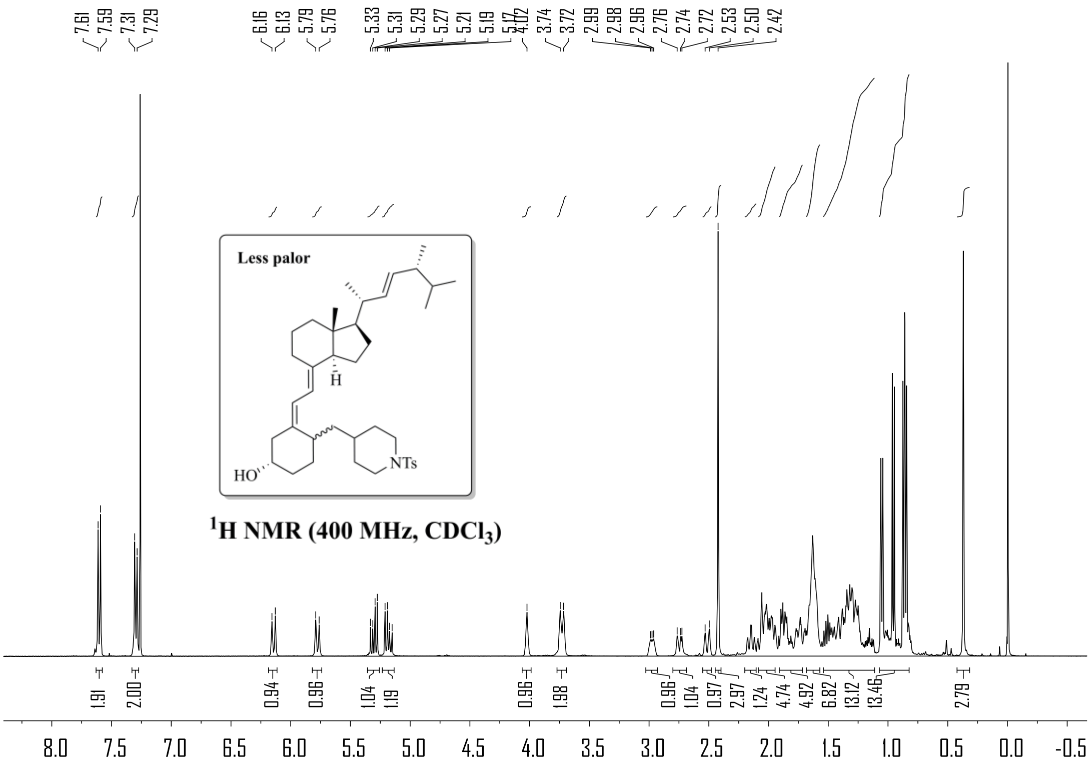

Supplementary Figure 88. ^{1}H NMR spectra for compound 42 (less palor one)

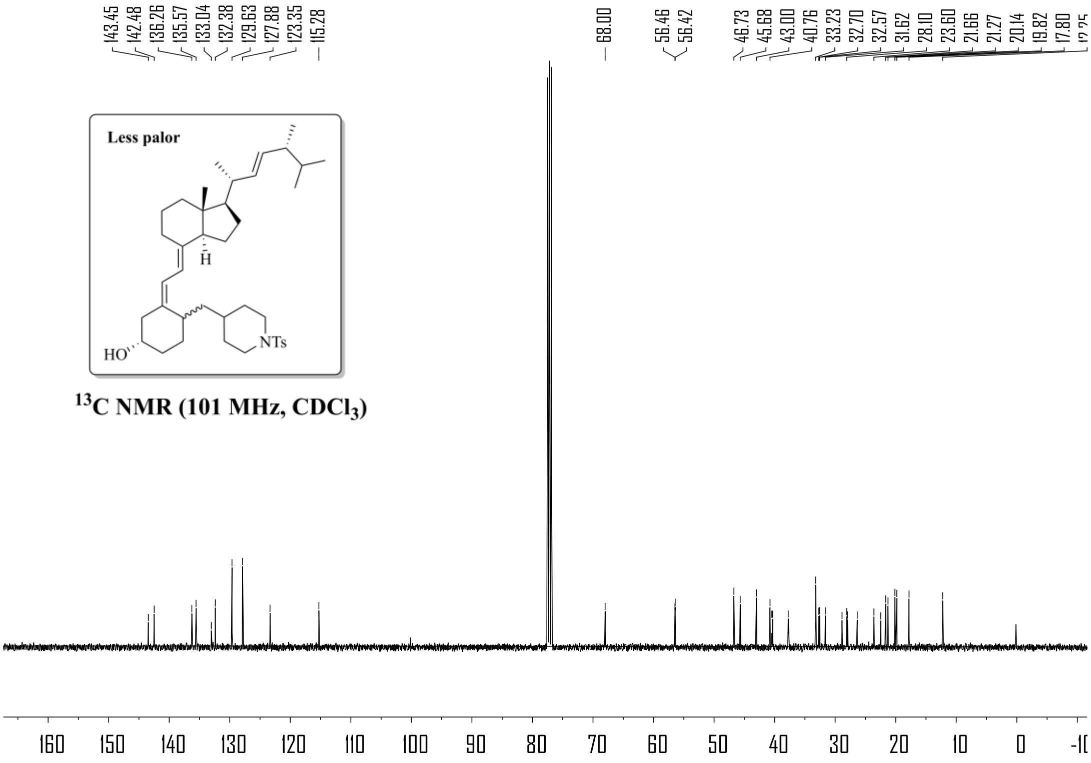

Supplementary Figure 89. ^{13}**C NMR spectra for compound 42 (less palor one)**

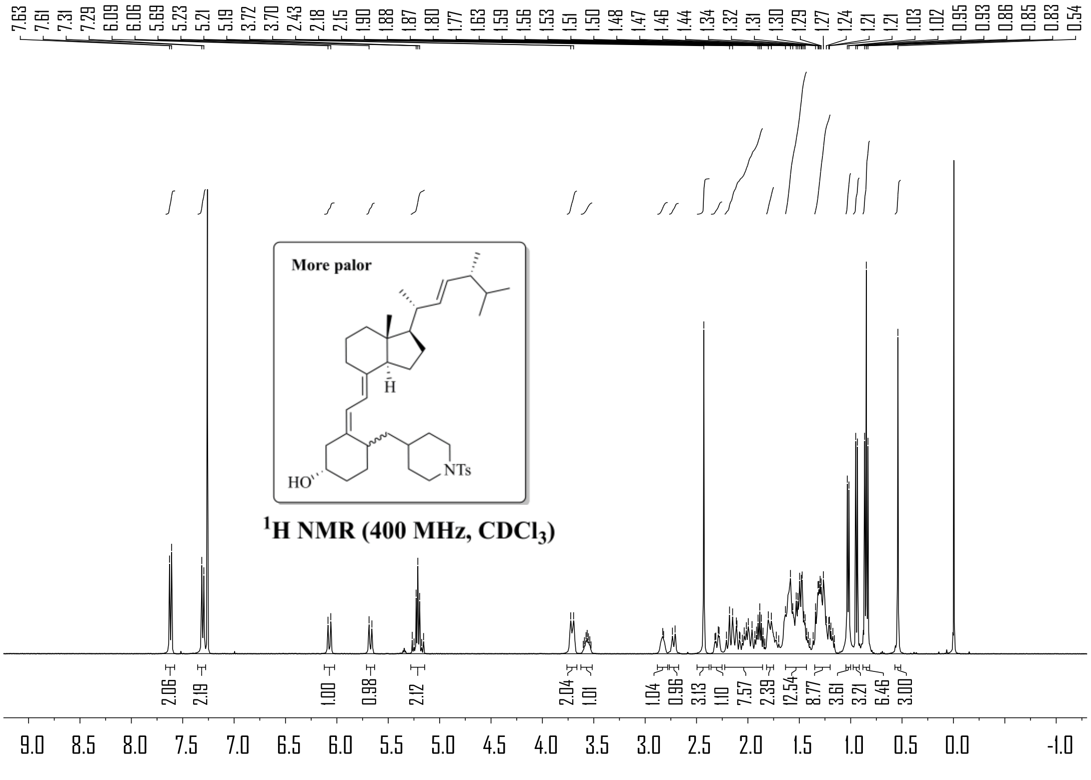

Supplementary Figure 90. ^{1}**H NMR spectra for compound 42 (more palor one)**

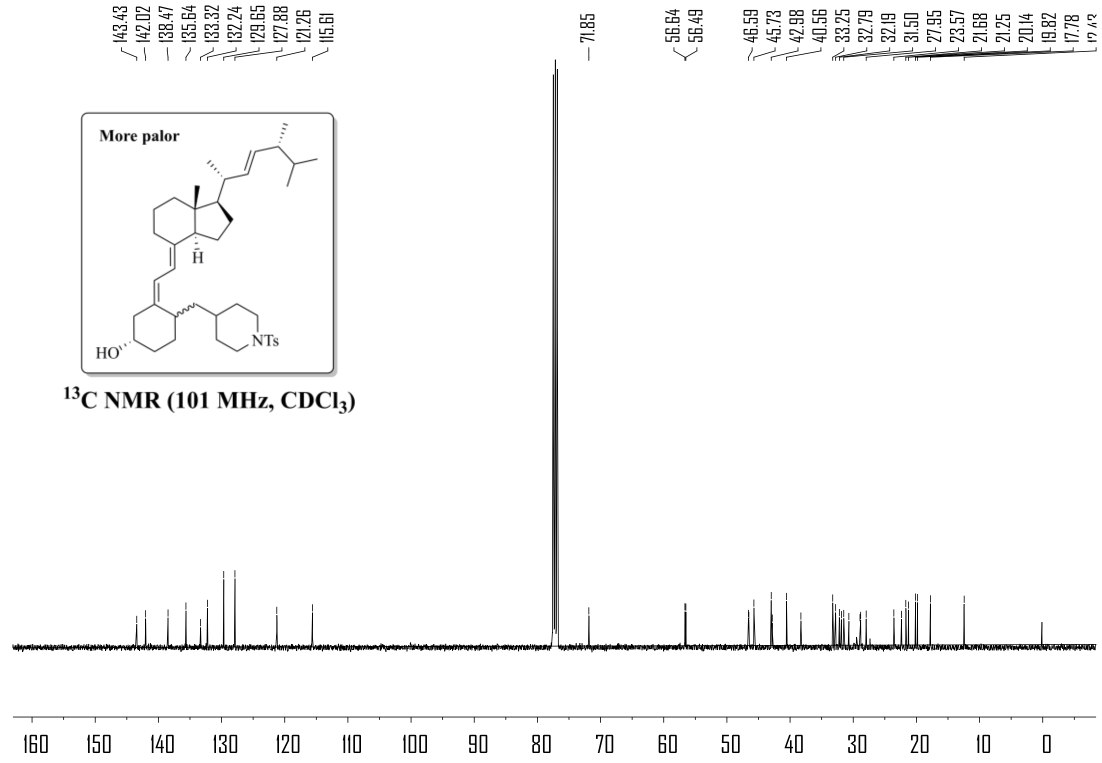

Supplementary Figure 91. ^{13}**C NMR spectra for compound 42 (more palor one)**

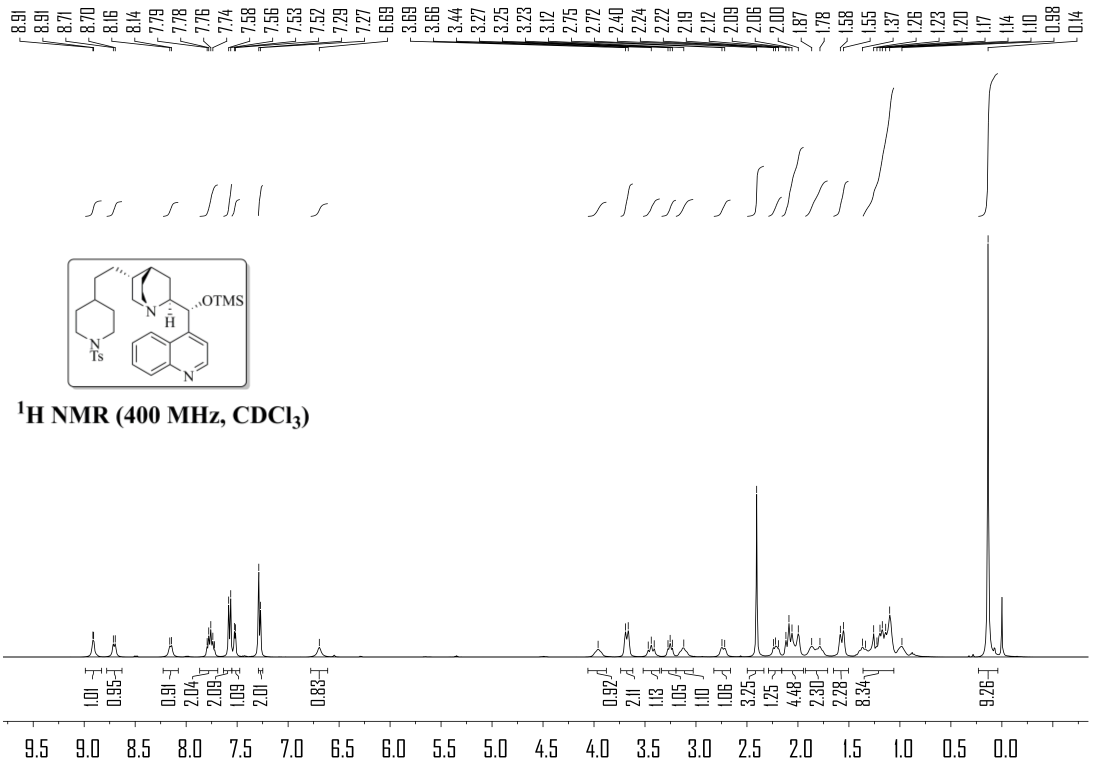

Supplementary Figure 92. ^{1}**H NMR spectra for compound 44**

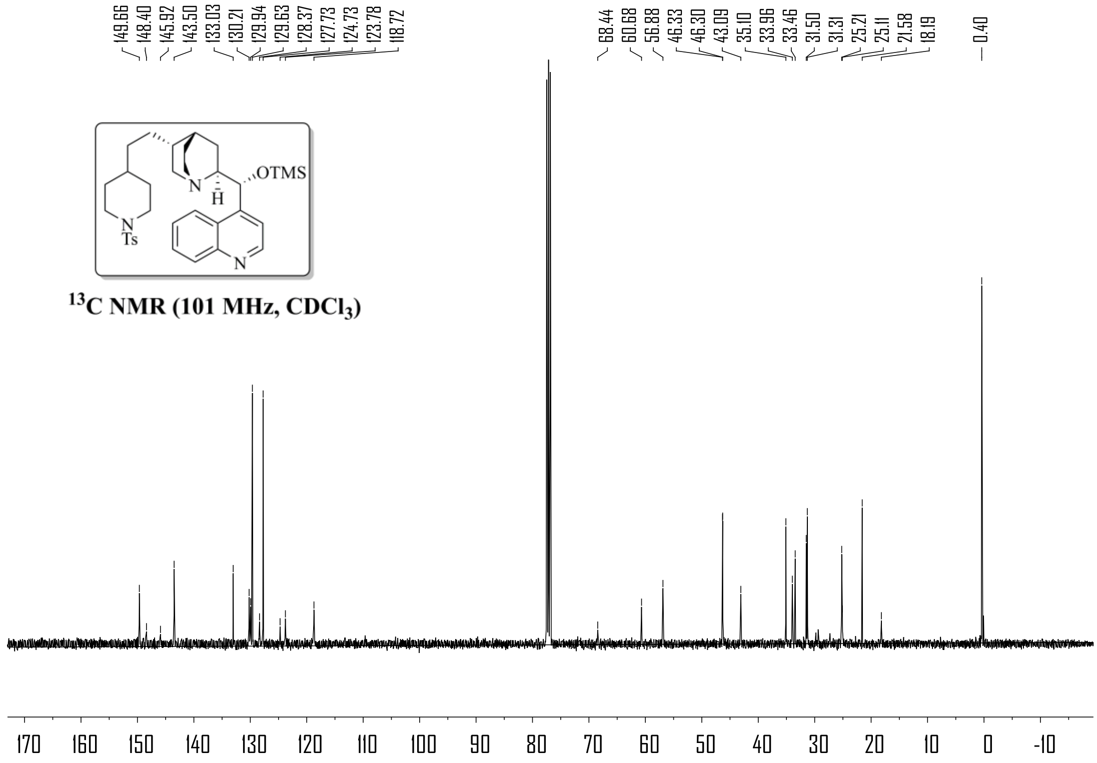

Supplementary Figure 93. ^{13}C NMR spectra for compound 44

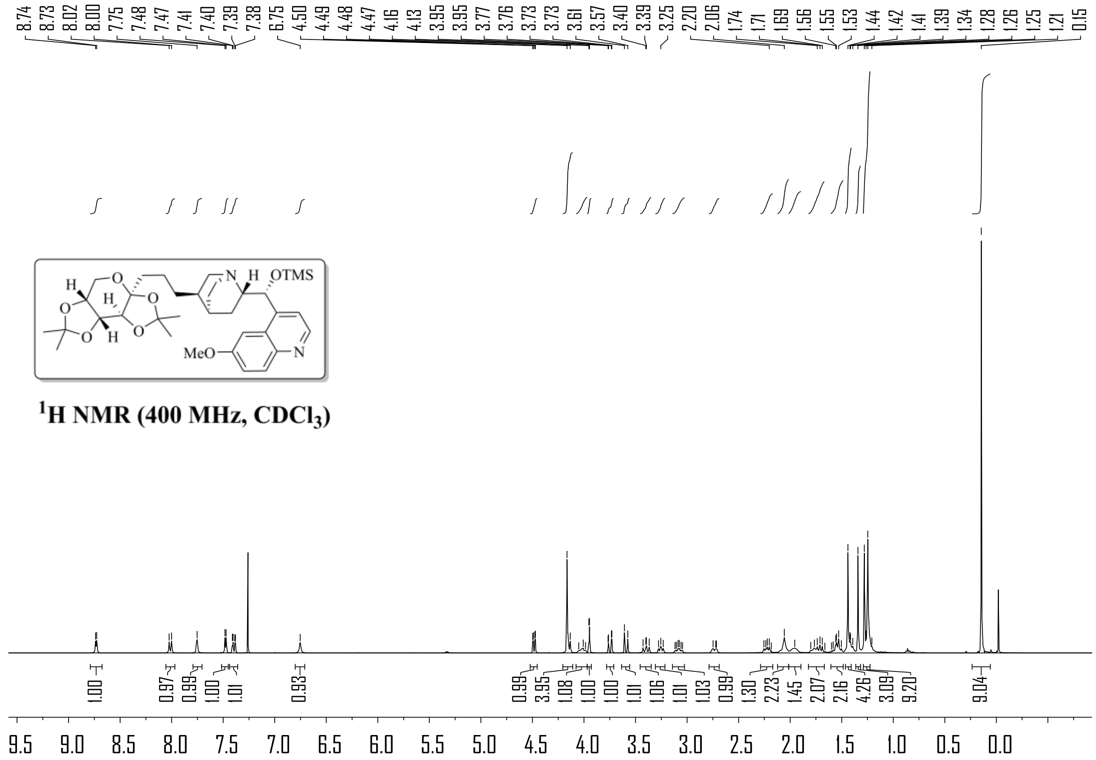

Supplementary Figure 94. ^{1}H NMR spectra for compound 47

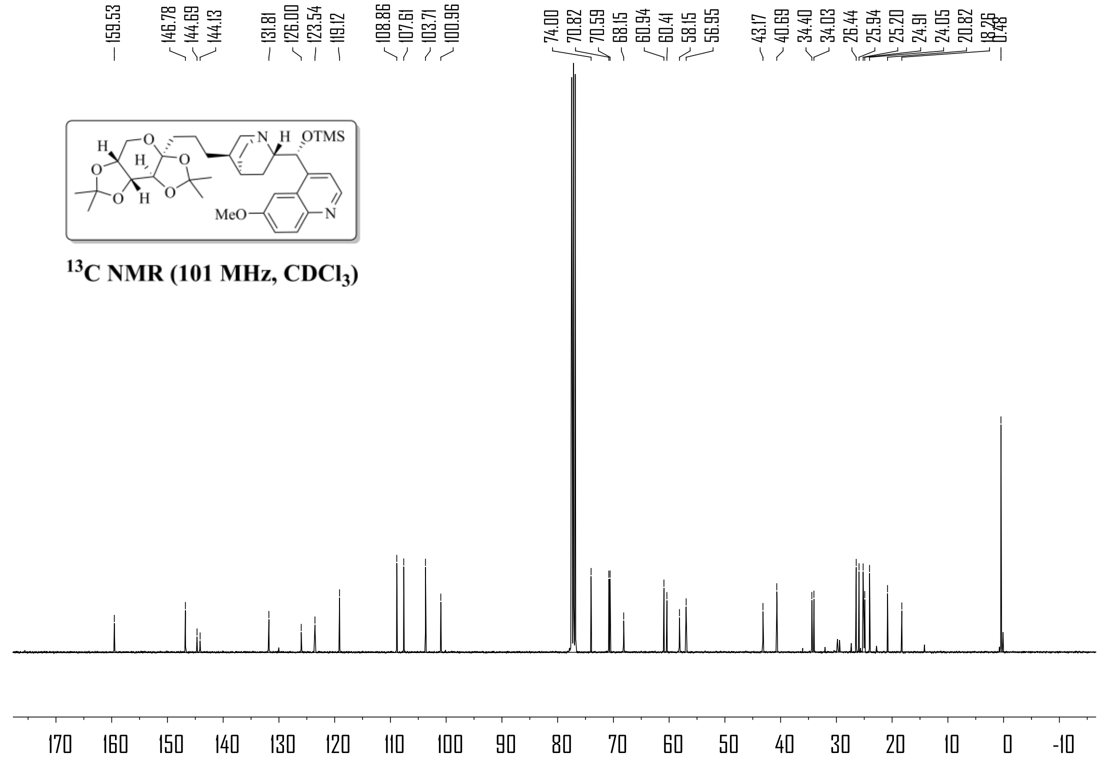

Supplementary Figure 95. ^{13}C NMR spectra for compound 47

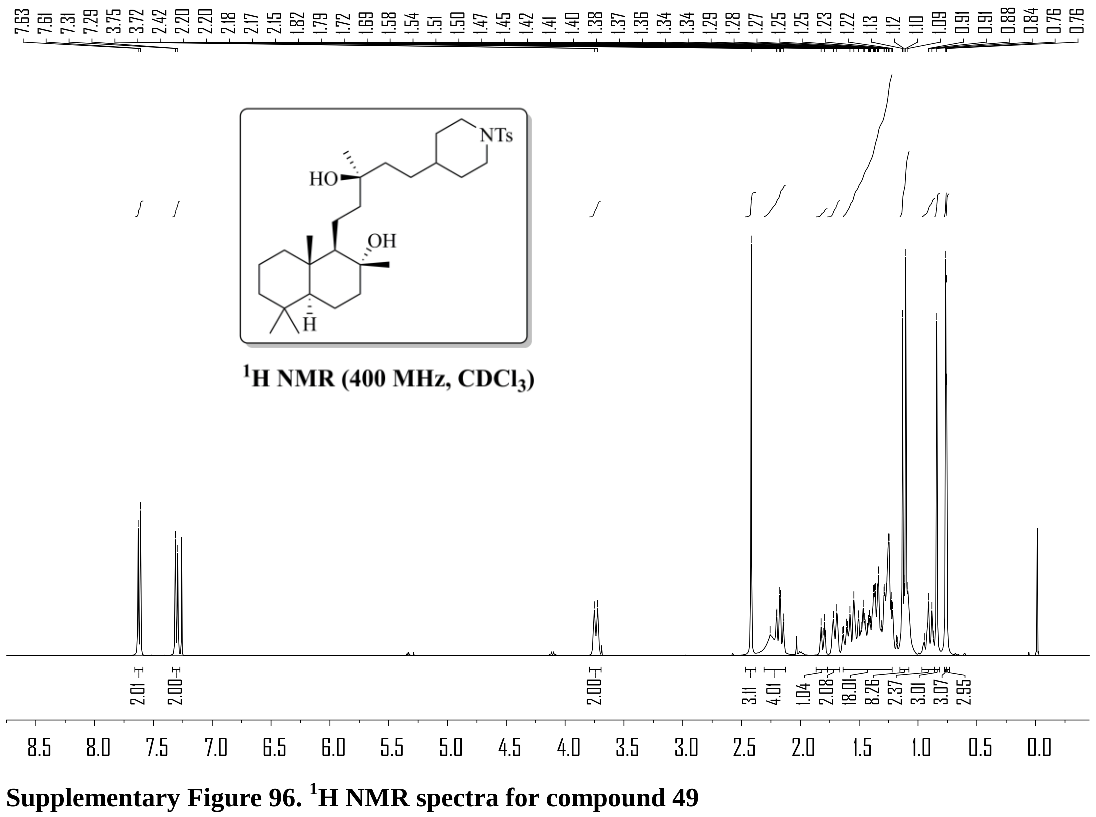

Supplementary Figure 96. ^{1}H NMR spectra for compound 49

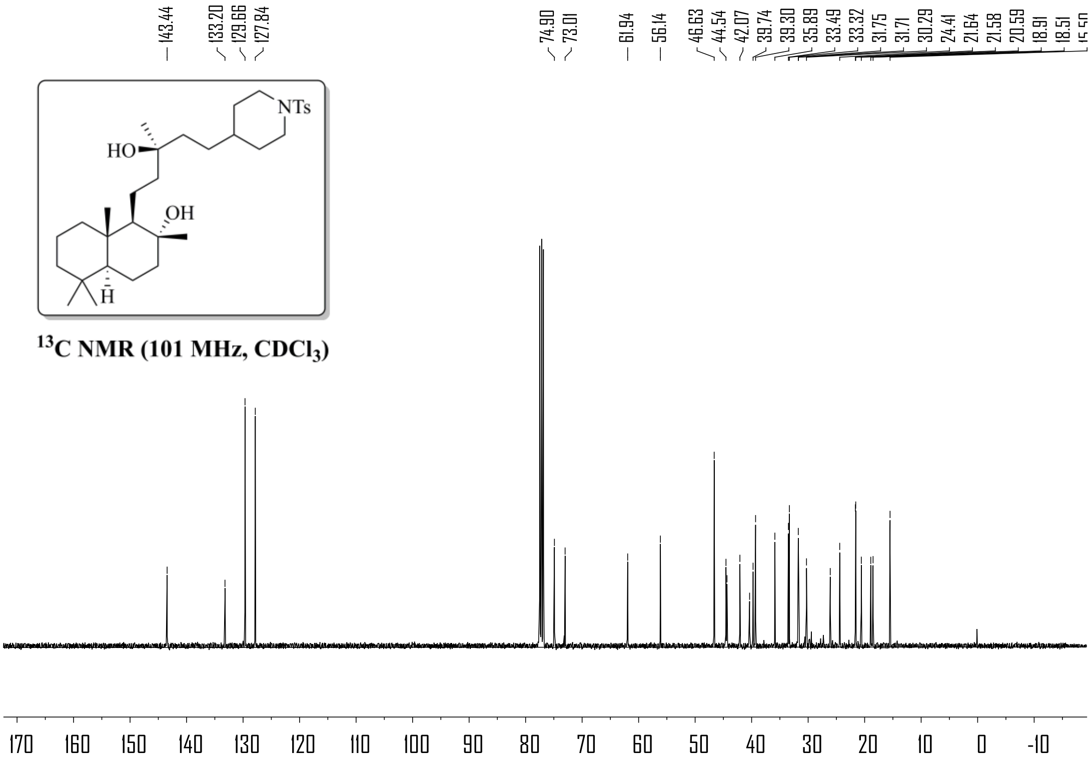

Supplementary Figure 97. ^{13}C NMR spectra for compound 49

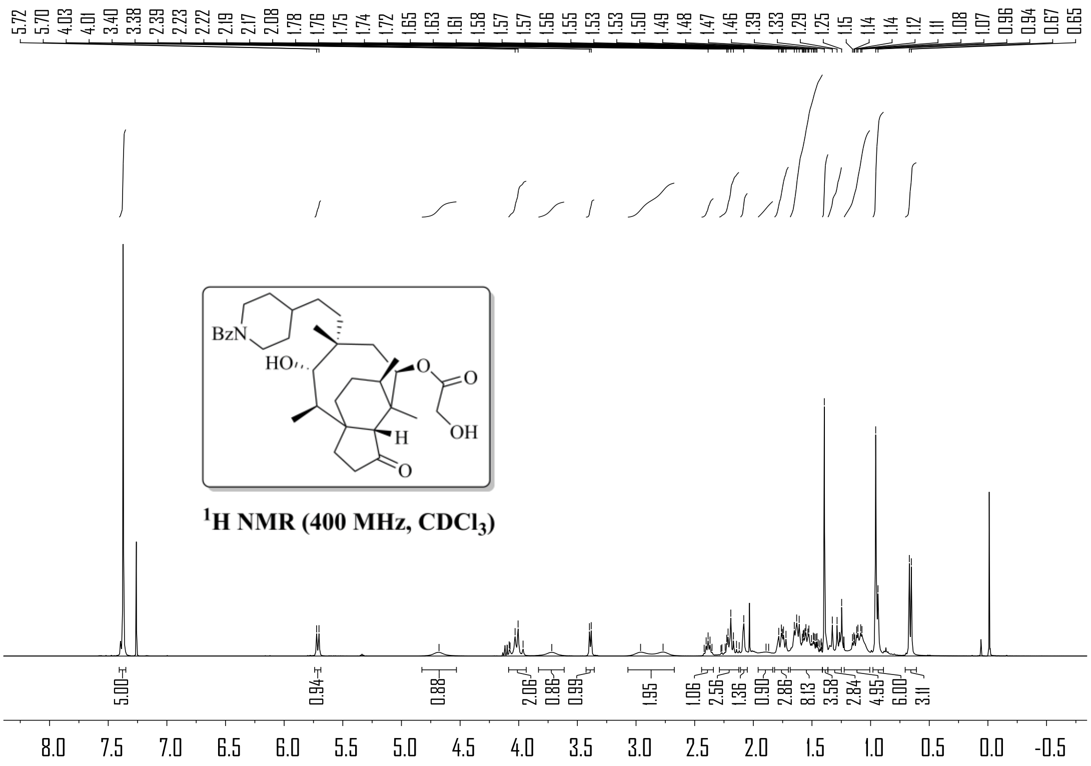

Supplementary Figure 98. ^{1}H NMR spectra for compound 52

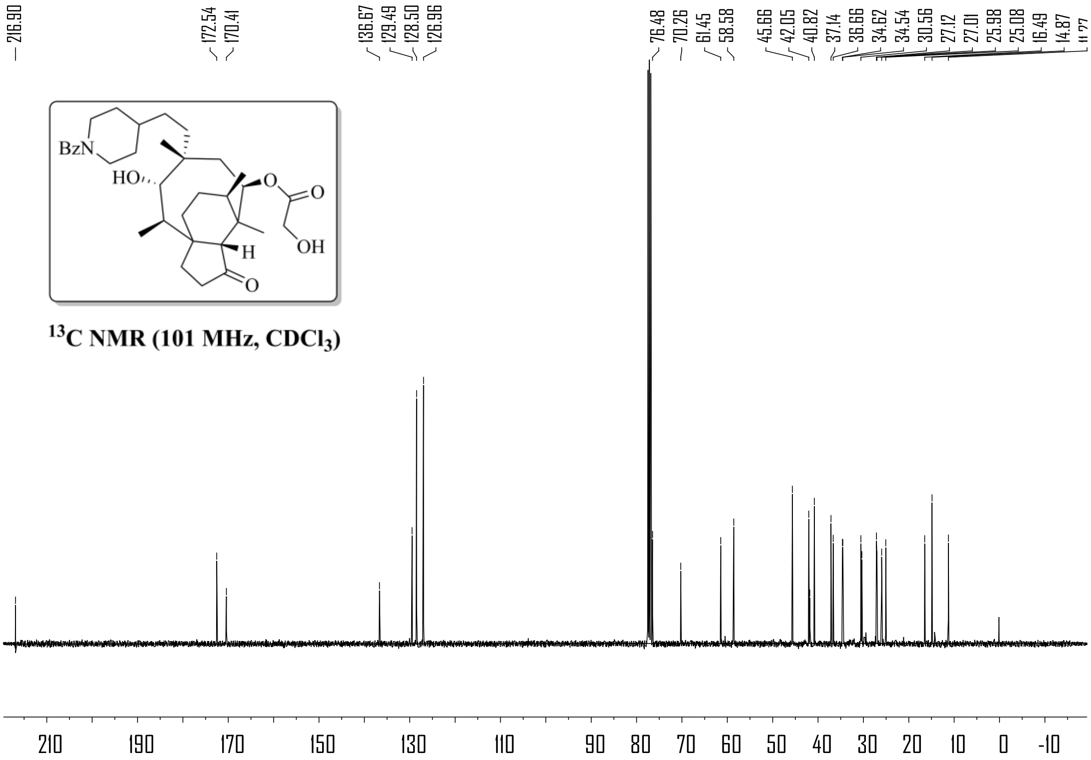

Supplementary Figure 99. ^{13}**C NMR spectra for compound 52**

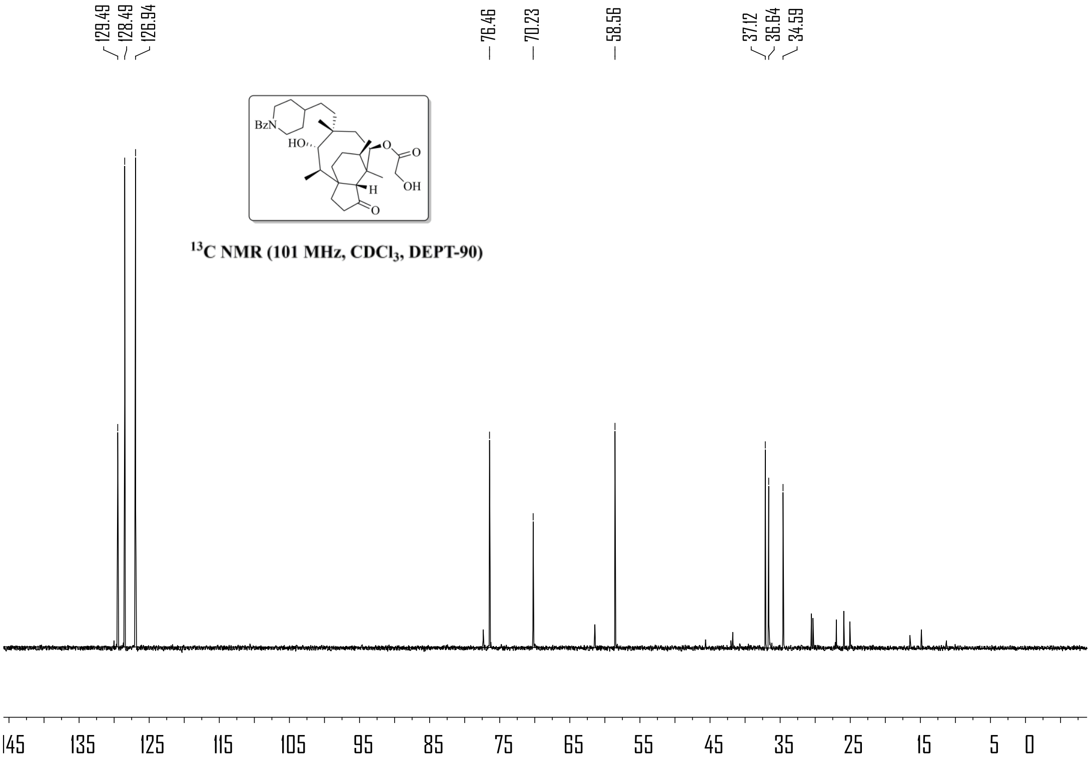

Supplementary Figure 100. ^{13}**C NMR (DEPT-90) spectra for compound 52**

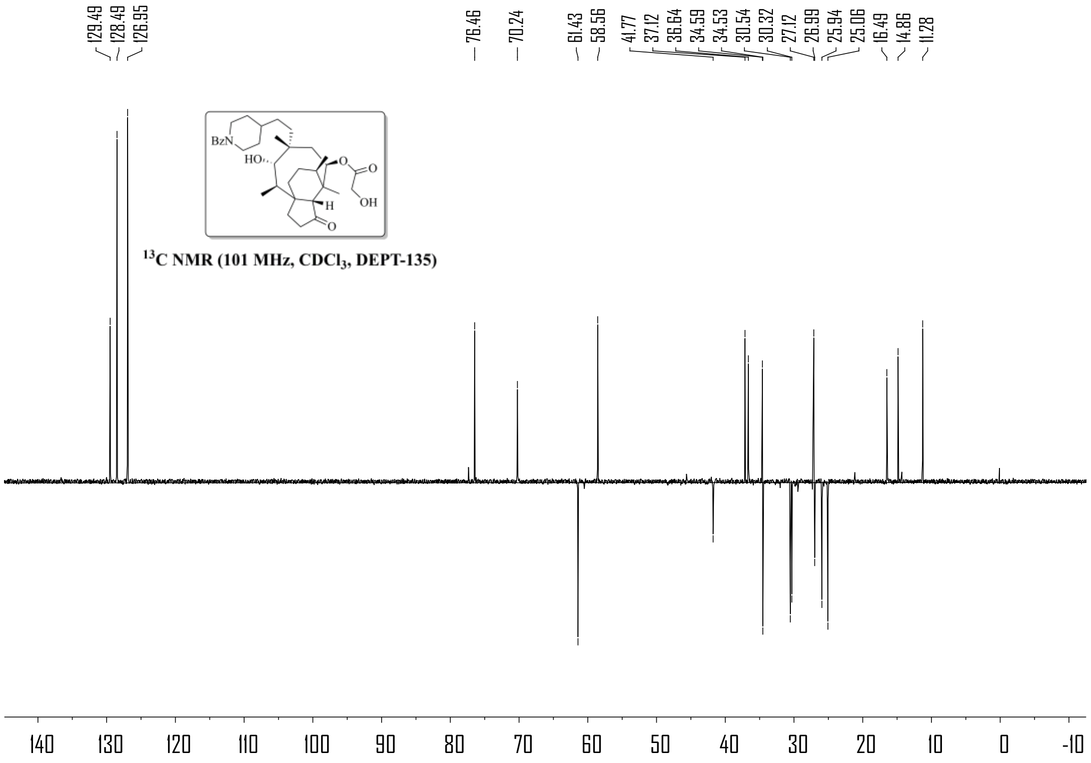

Supplementary Figure 101. ^{13}C NMR (DEPT-135) spectra for compound 52

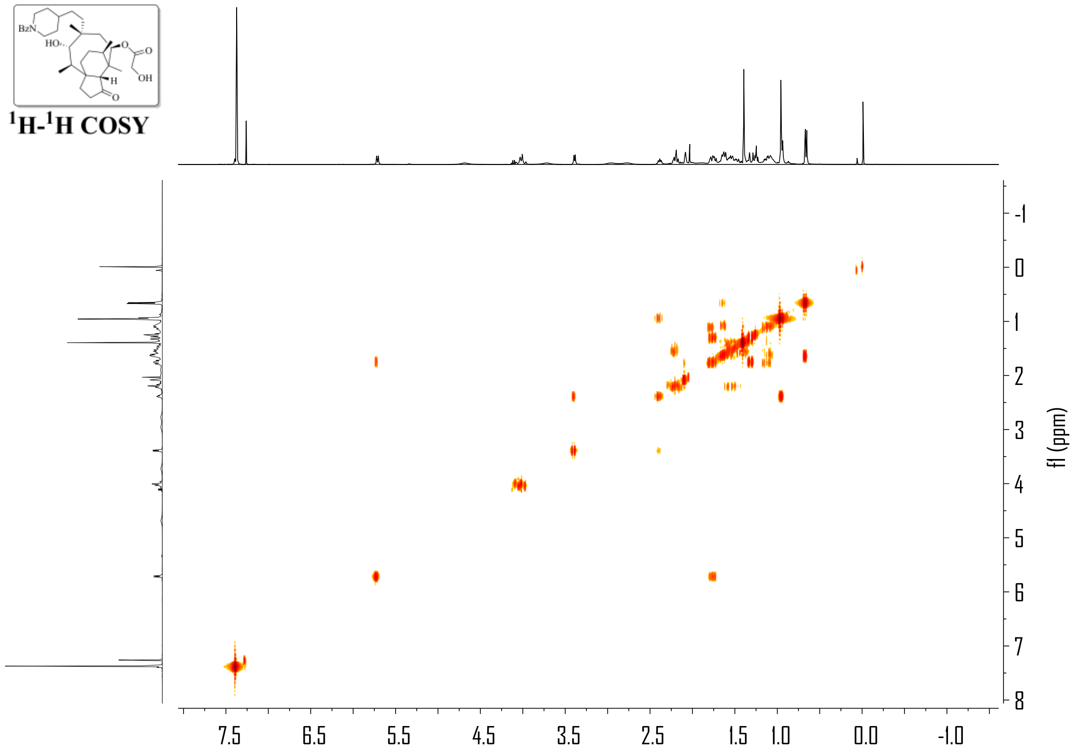

Supplementary Figure 102. ^{1}H-^{1}H COSY spectra for compound 52

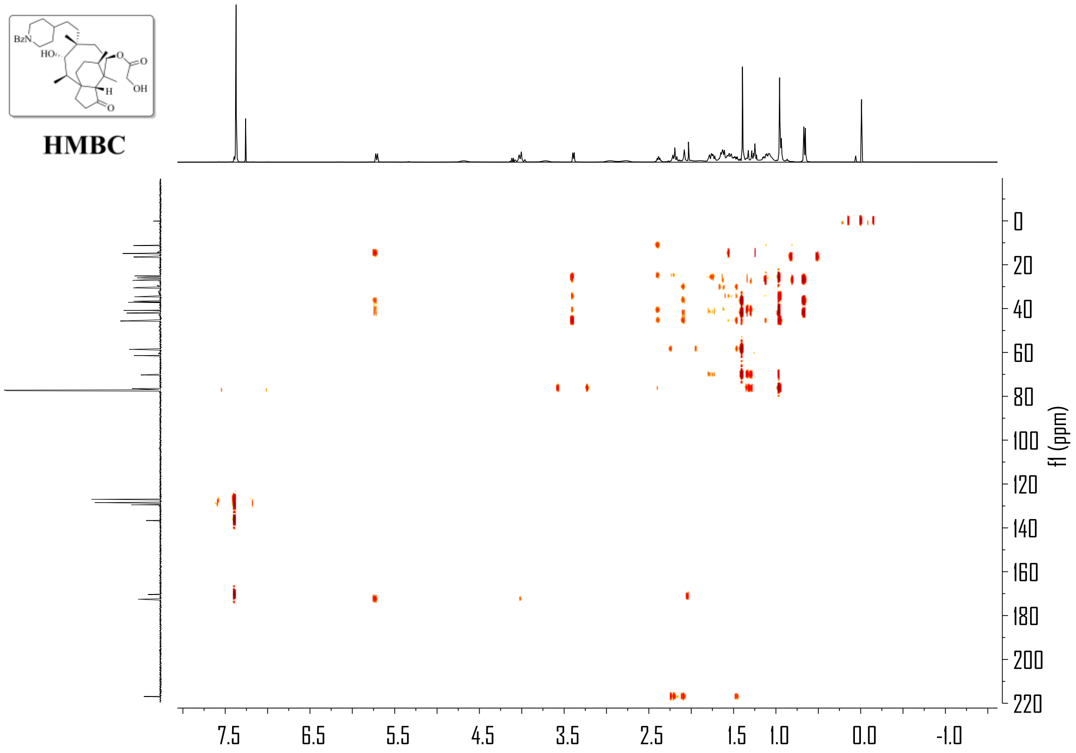

Supplementary Figure 103. HMBC spectra for compound 52

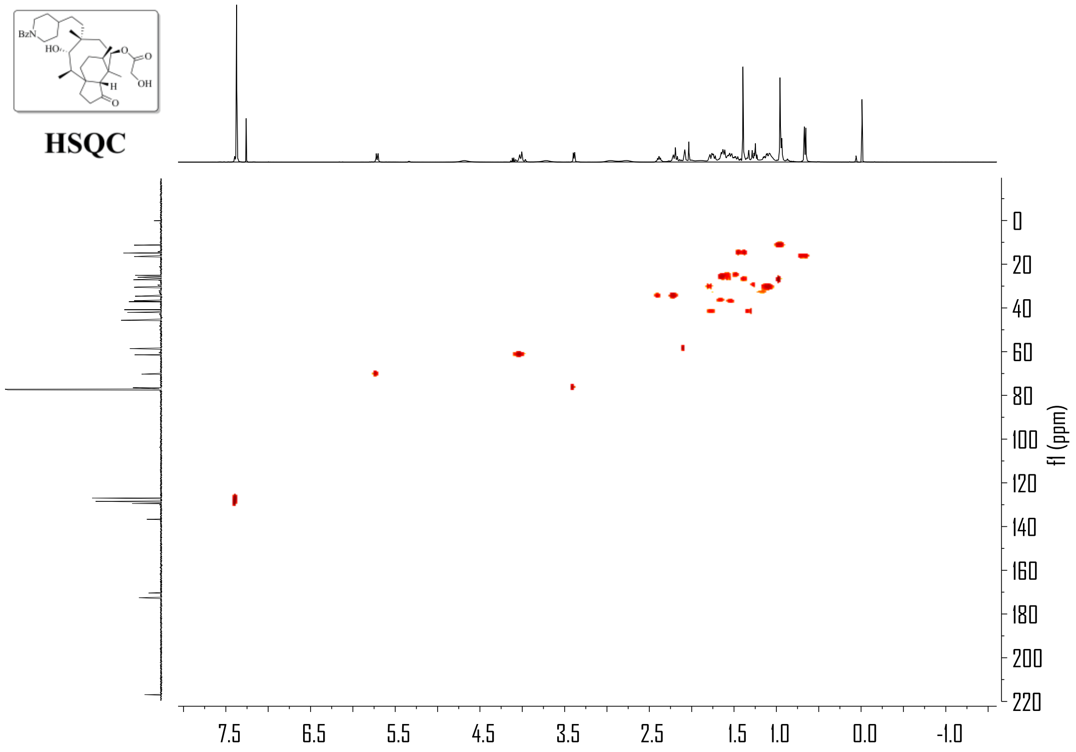

Supplementary Figure 104. HSQC spectra for compound 52

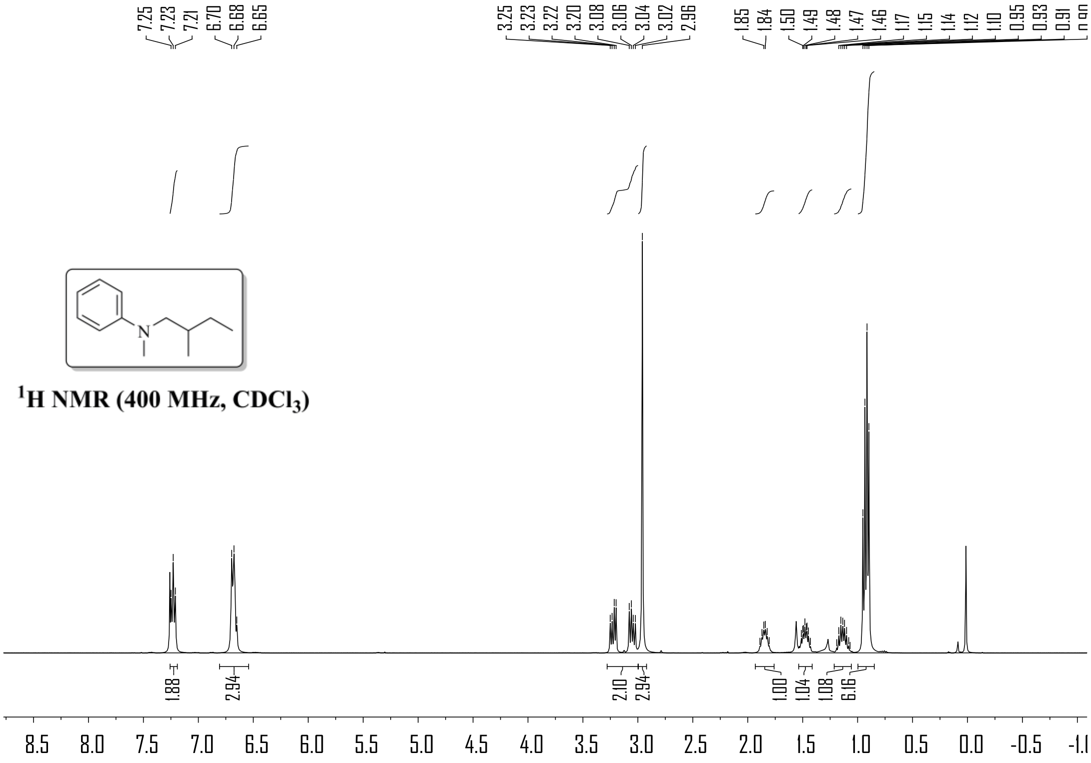

Supplementary Figure 105. ^{1}H NMR spectra for compound 54

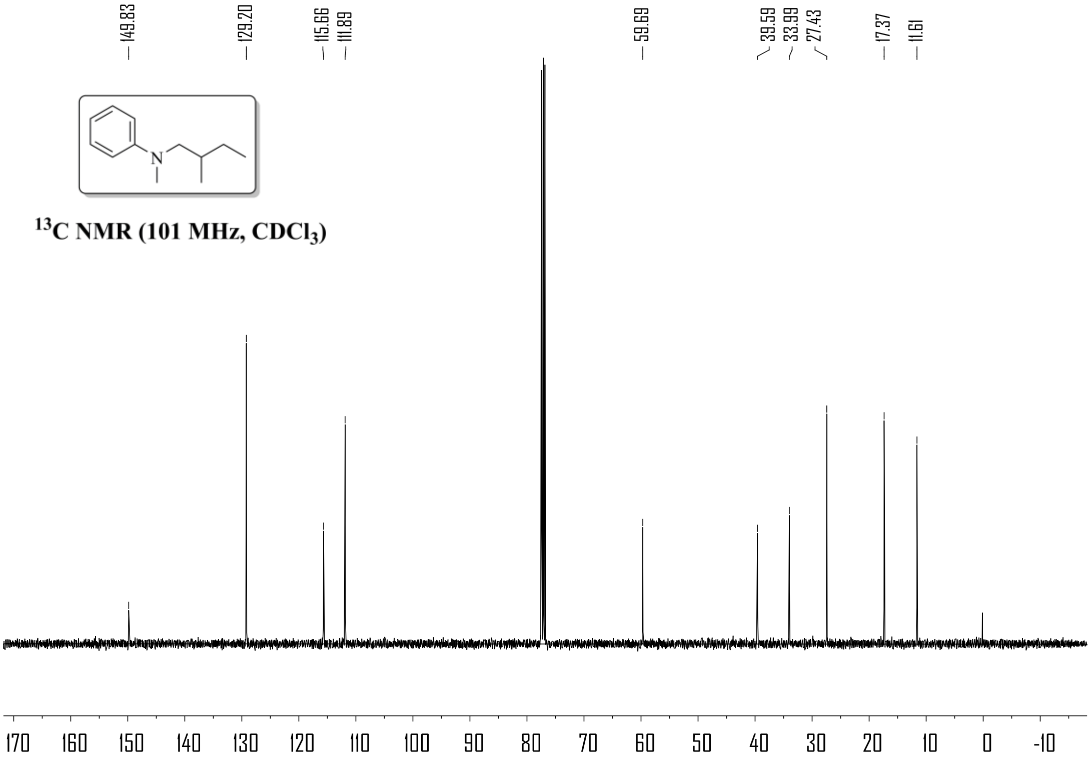

Supplementary Figure 106. ^{13}C NMR spectra for compound 54

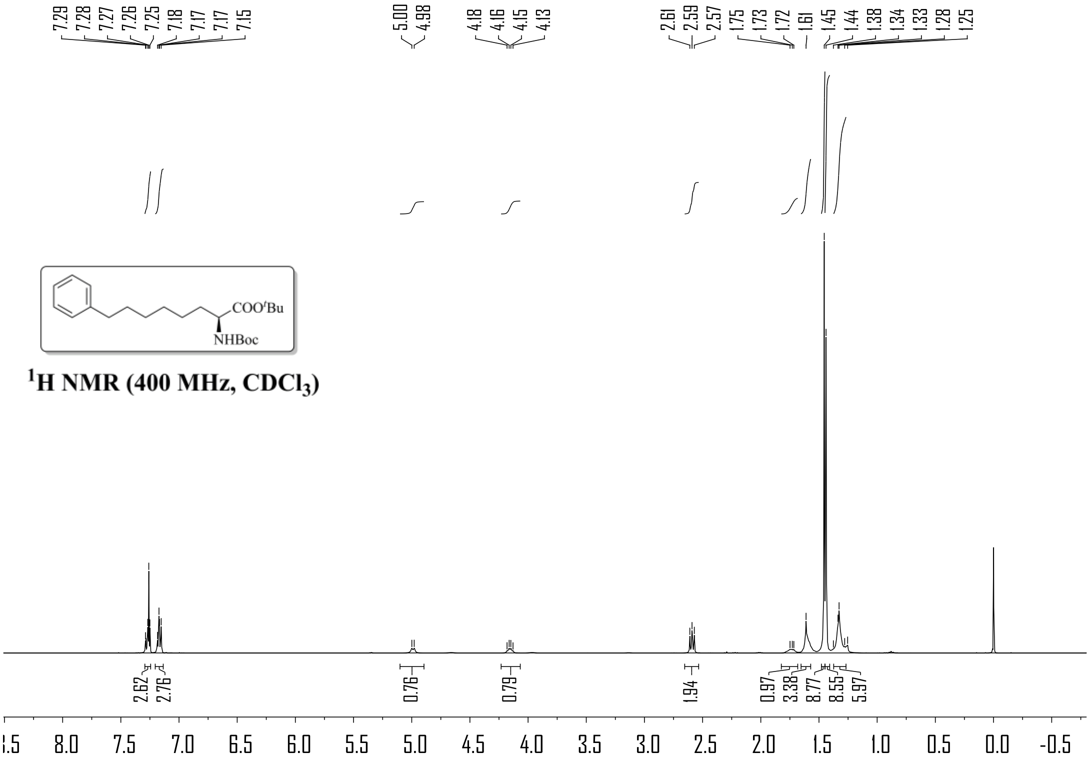

Supplementary Figure 107. ¹H NMR spectra for compound 59

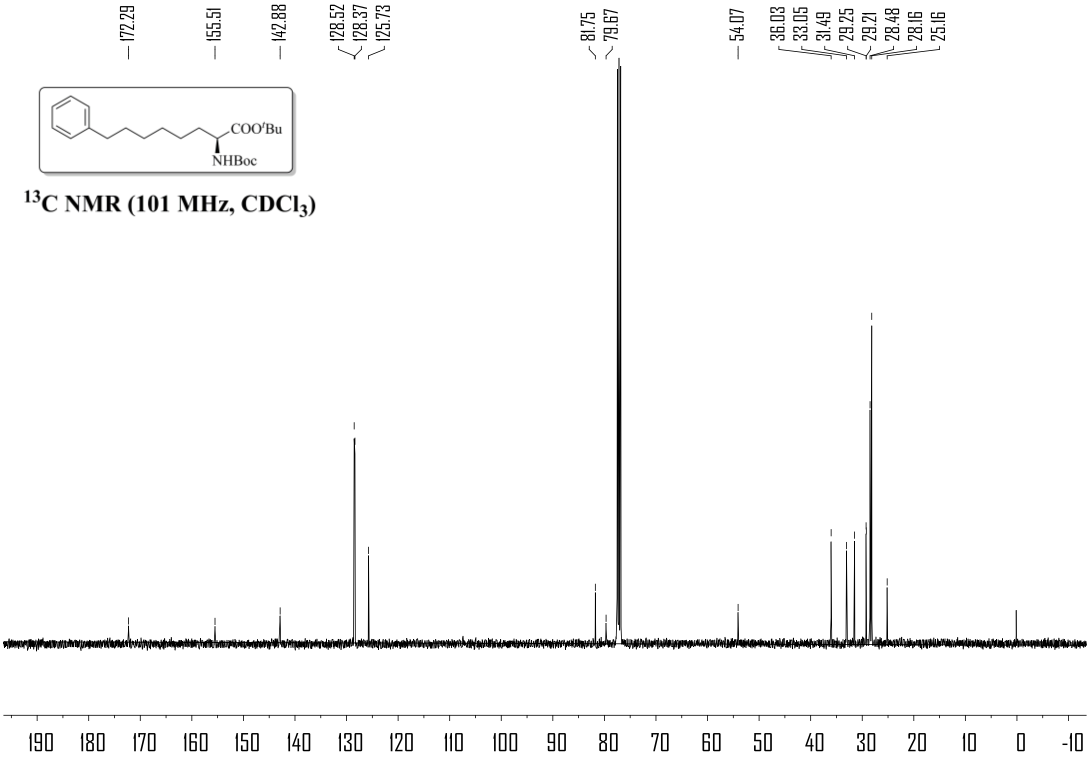

Supplementary Figure 108. ¹³C NMR spectra for compound 59

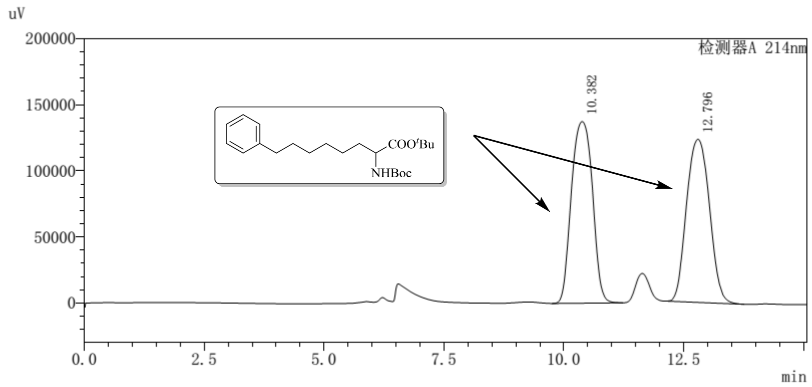

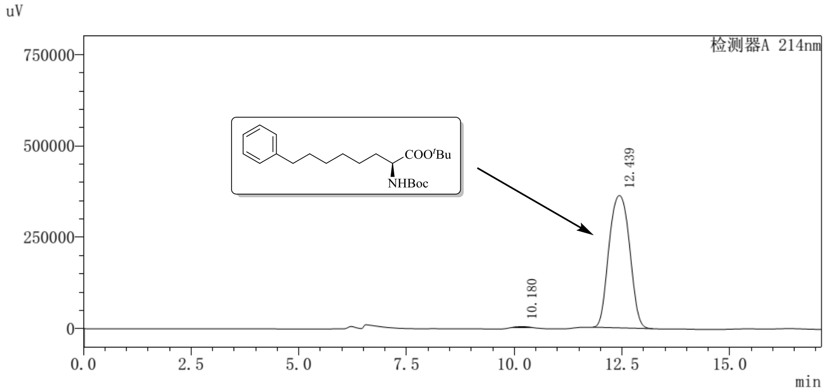

Supplementary Figure 109. HPLC spectra for compound 59

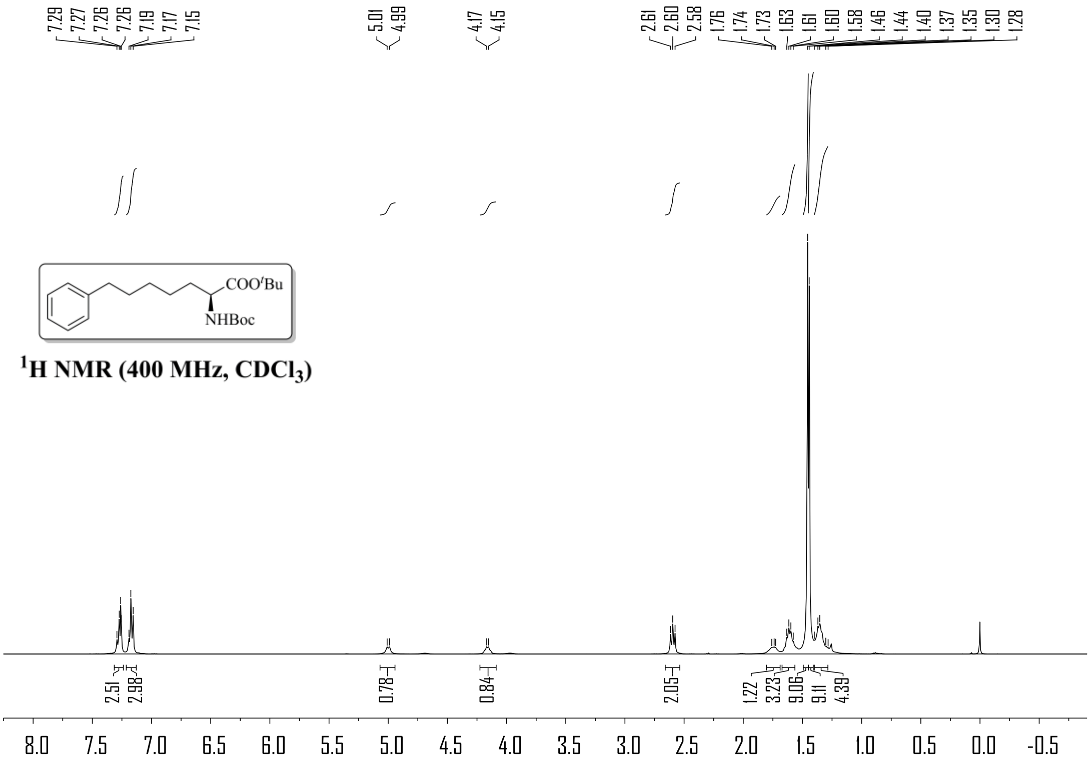

Supplementary Figure 110. ^{1}H NMR spectra for compound 60

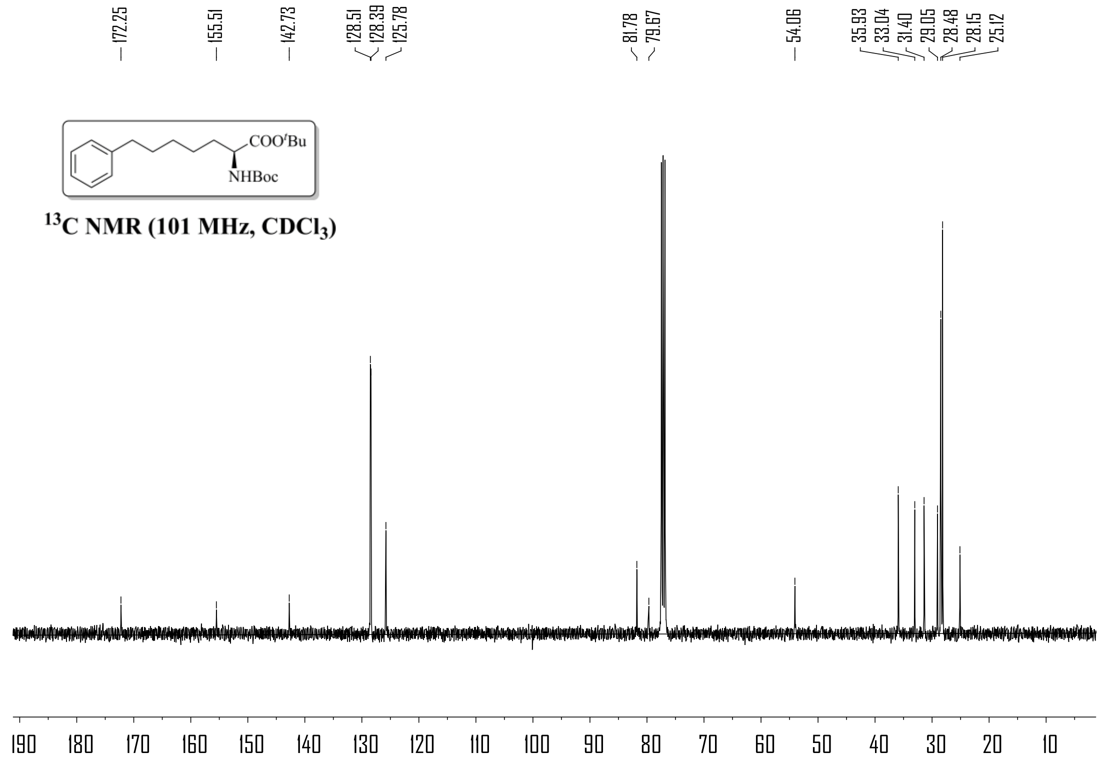

Supplementary Figure 111. ^{13}C NMR spectra for compound 60

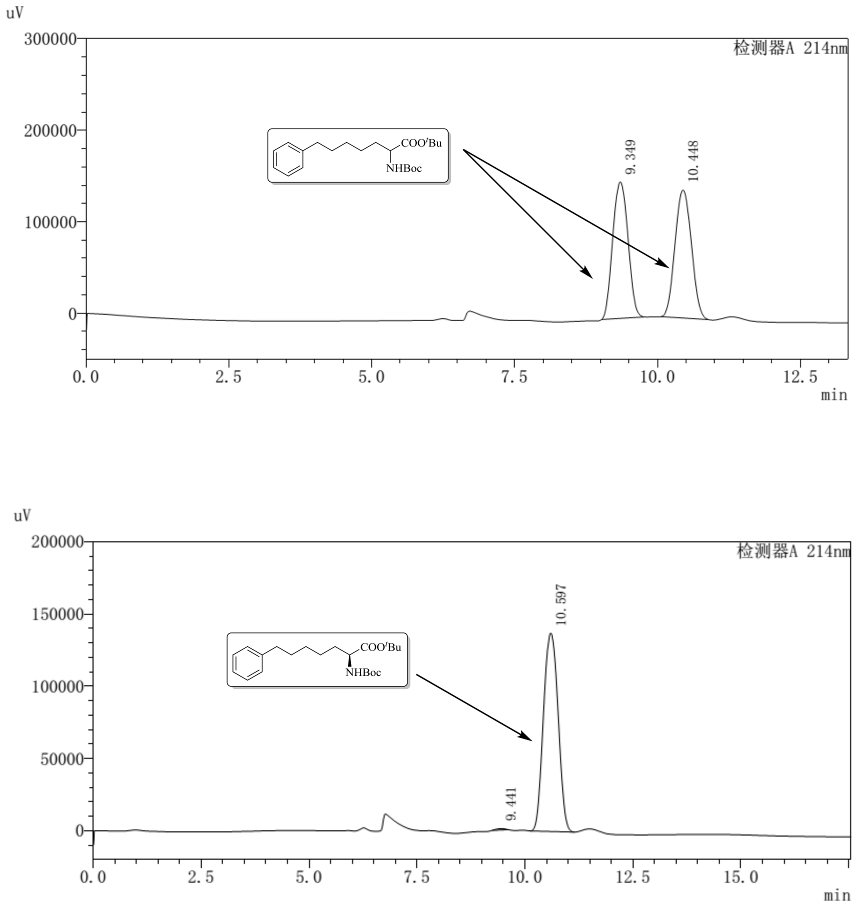

Supplementary Figure 112. HPLC spectra for compound 60

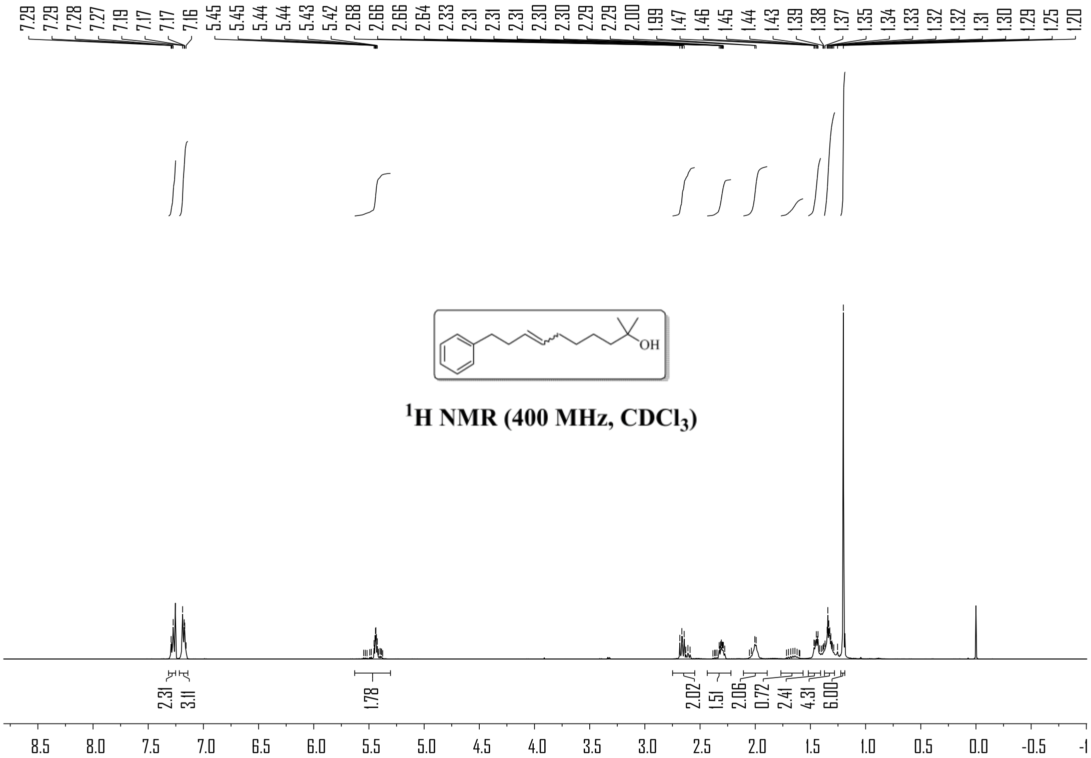

Supplementary Figure 113. ^{1}H NMR spectra for compound 62

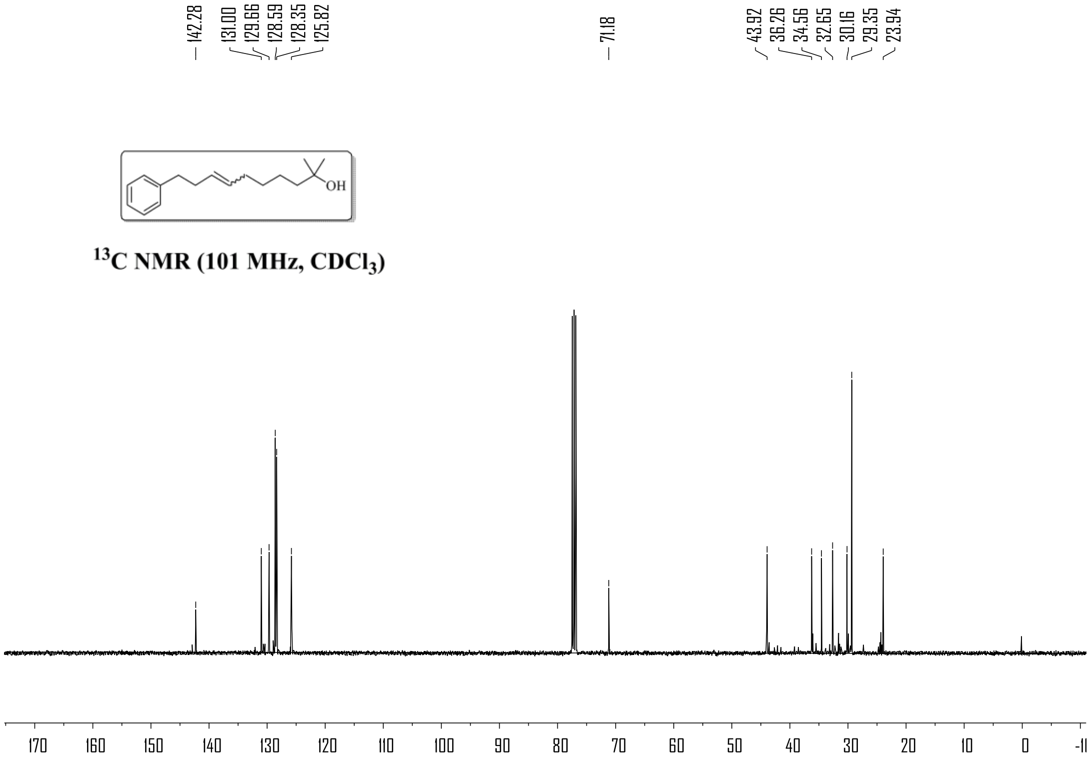

Supplementary Figure 114. ^{13}C NMR spectra for compound 62

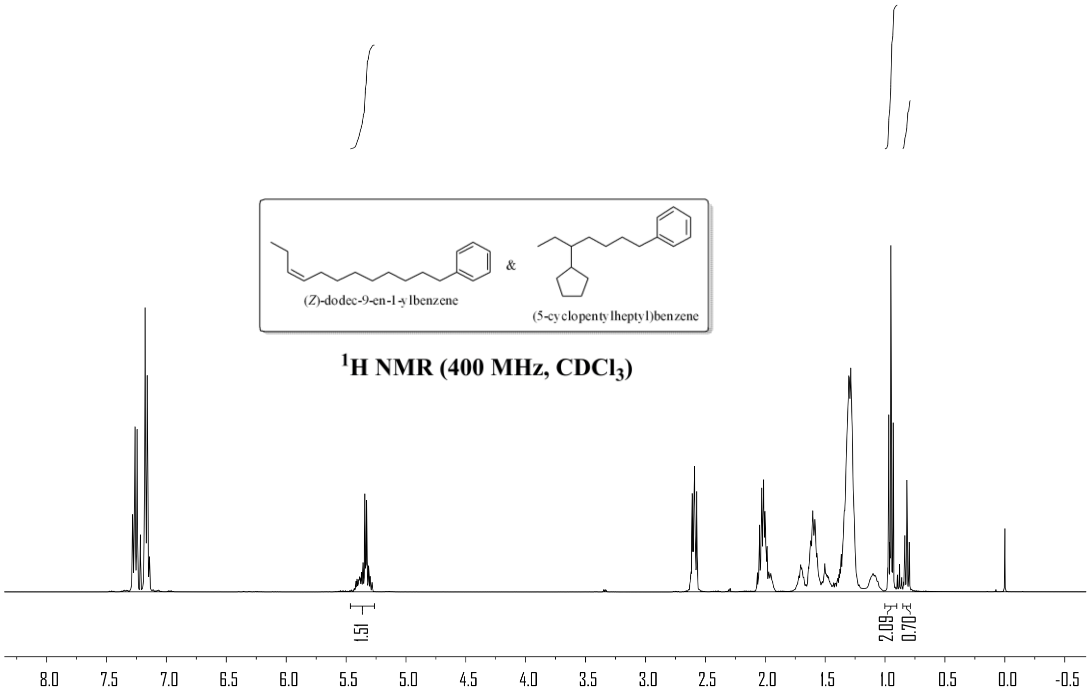

Supplementary Figure 115. ^{1}H NMR spectra for compound 64a & 64b

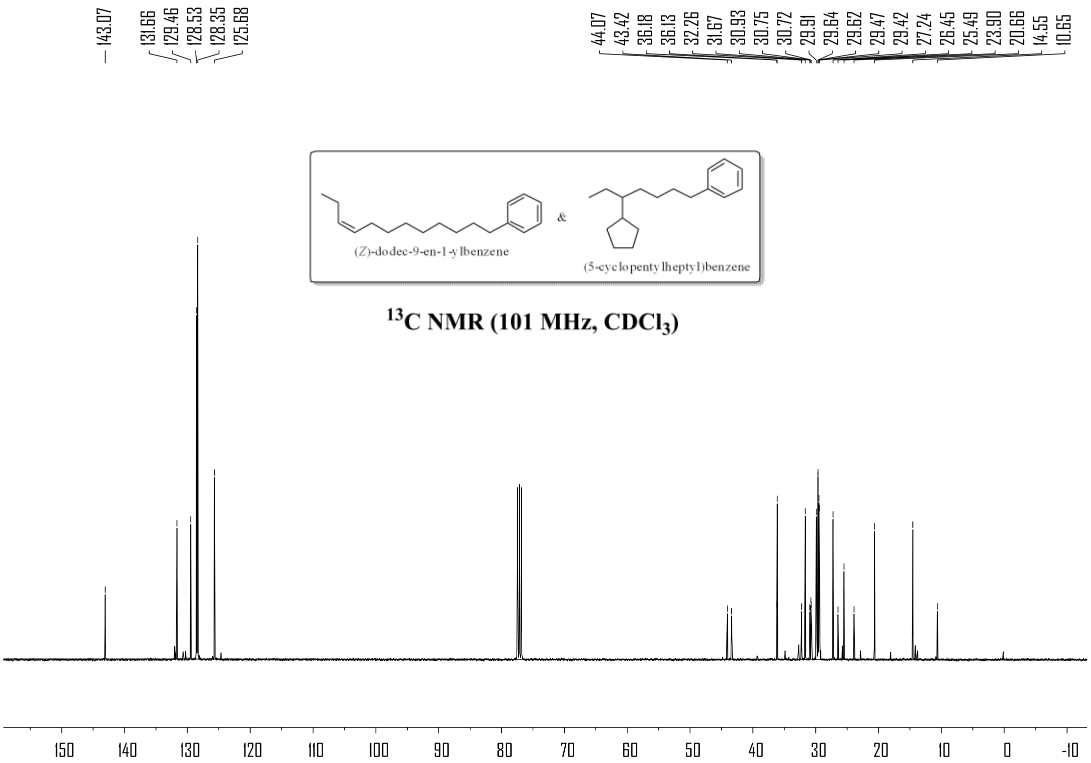

Supplementary Figure 116. ^{13}C NMR spectra for compound 64a & 64b

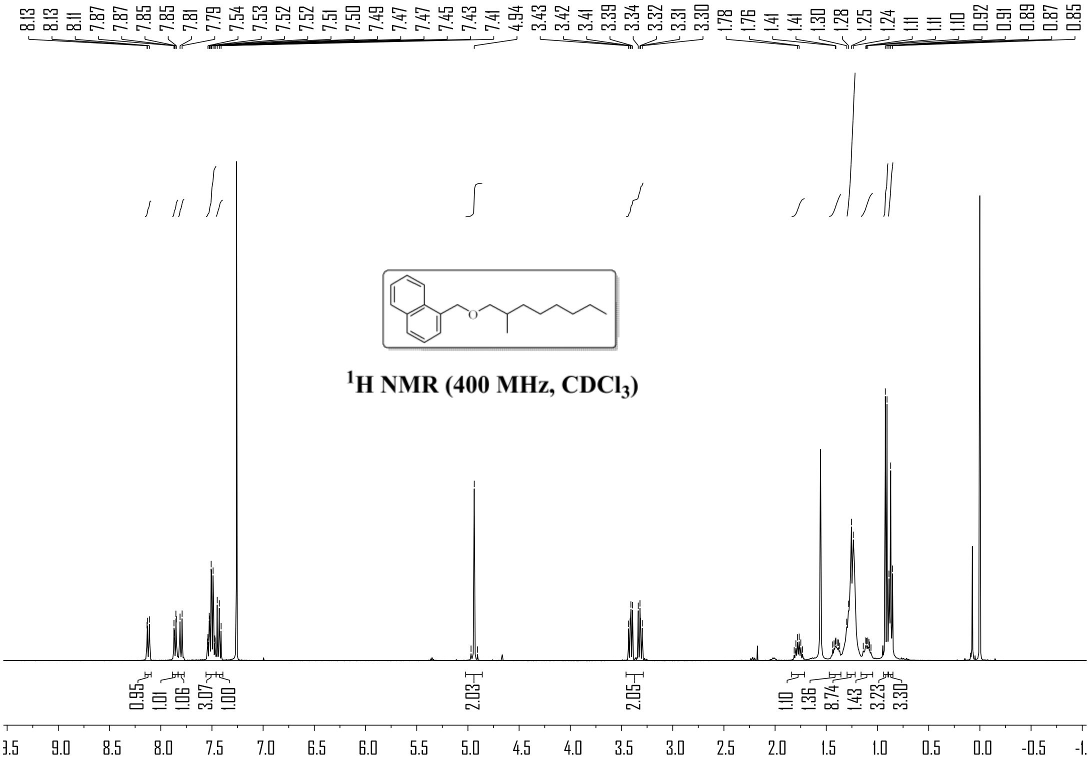

Supplementary Figure 117. ^{1}**H NMR spectra for compound 67**

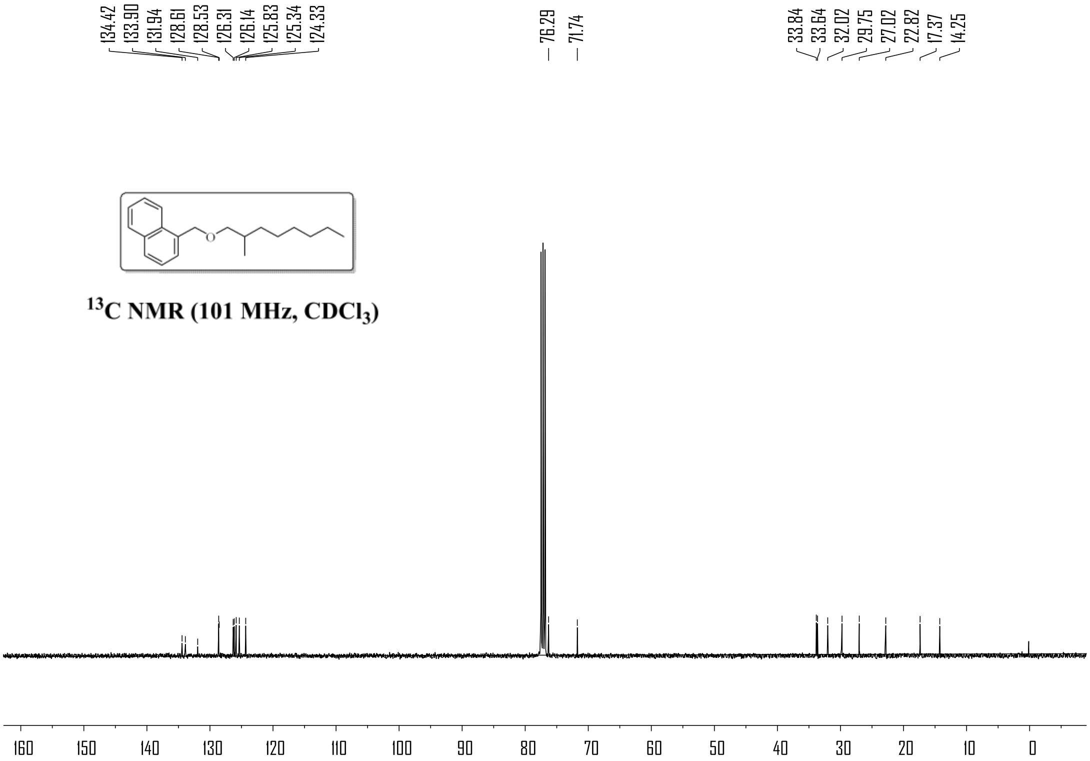

Supplementary Figure 118. ^{13}**C NMR spectra for compound 67**

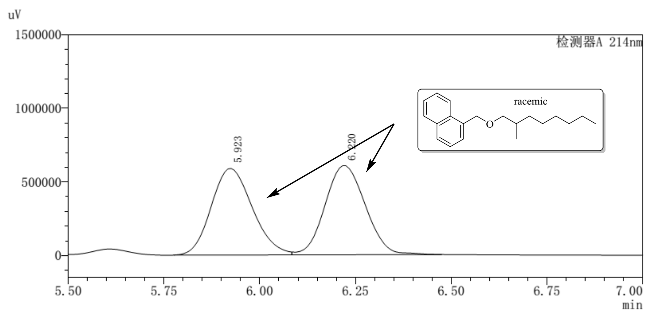

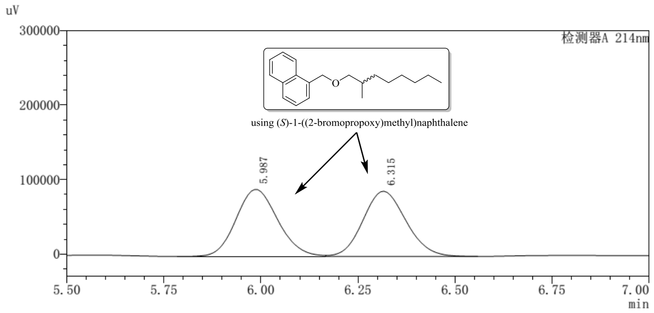

Supplementary Figure 119. HPLC spectra for compound 67

Supplementary Tables

Supplementary Table 1. Ligand screening for the coupling of 1 and 2.

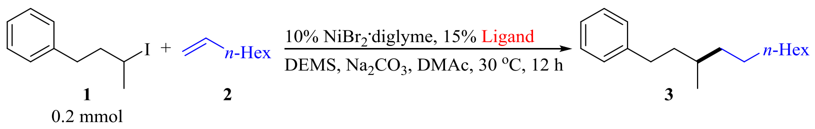

Entry	Ligand	GC yield of **3** (%)	Entry	Ligand	GC yield of **3** (%)
1	**L1** complex	<1[b]	7	**L7**	44[b]
2	**L2**	<1[b]	8	**L8**	***96[b] (93[b,d])***
3	**L3**	3[b]	9	PPh_2Cy	N.R.[b,c]
4	**L4**	12[b]	10	$PPhCy_2$	N.R.[b,c]
5	**L5**	18[b]	11	**L11**	N.R.[b]
6	**L6**	<1[b]	12	**L8**	***85[a] (82[a,d])***

[a] 1.5 equiv. Alkene, 2 equiv. DEMS, 2 equiv. Na_2CO_3, 0.6 mL DMAc were used. [b] 2.5 equiv. Alkene, 3 equiv. DEMS, 3 equiv. Na_2CO_3, 0.6 mL DMAc were used. [c] 20% ligand was uesd. [d] Isolated yield.

Supplementary Table 2. Nickel source screening for the coupling of 1 and 2.

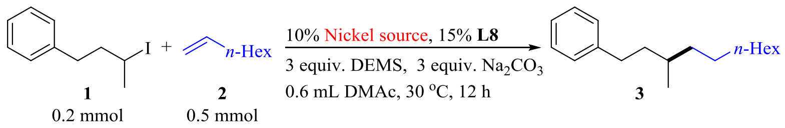

Entry	Nickel source	GC yield of **3** (%)	Entry	Nickel source	GC yield of **3** (%)
1	**$NiBr_2$·diglyme**	***96 (93[a])***	6	$NiCl_2(dppp)$	30
2	$NiBr_2$·glyme	88	7	$Ni(OTf)_2$	67
3	$NiCl_2$	23	8	$Ni(acac)_2$	6
4	NiI_2	57	9	$Ni(COD)_2$	13
5	$NiCl_2(PPh_3)_2$	25	10	--	N.R.

[a] Isolated yield.

Supplementary Table 3. Base screening for the coupling of 1 and 2.

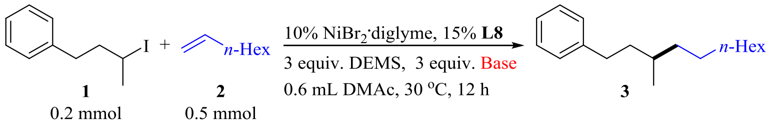

Entry	Base	GC yield of **3** (%)	Entry	Base	GC yield of **3** (%)
1	NaOAc	21	8	**Na_2CO_3**	***96 (93[a])***
2	K_3PO_4	94	9	CF_3COOK	22
3	CsF	85	10	$Mg(OAc)_2$	93
4	KF	64	11	$KHCO_3$	95
5	NaF	30	12	Cs_2CO_3	81
6	CsOAc	13	13	LiOMe	69
7	K_2CO_3	78	14	NaO^{t}Bu	4

[a] Isolated yields.

Supplementary Table 4. Solvent screening for the coupling of 1 and 2.

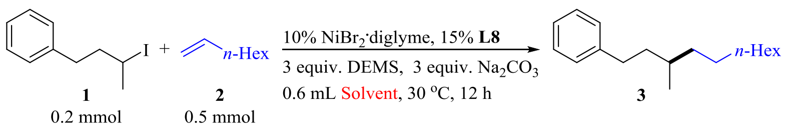

Entry	Solvent	GC yield of **3** (%)	Entry	Solvent	GC yield of **3** (%)
1	NMP	84	8	THF/NMP (v/v=5/1)	72
2	**DMAc**	***96 (93[a])***	9	THF/DMSO (v/v=5/1)	95
3	DMF	91	10	THF/CH_3CN (v/v=5/1)	33
4	DMSO	66	11	1,4-Dioxane/DMAc (v/v=5/1)	52
5	CH_3CN	38	12	Diglyme/DMAc (v/v=5/1)	70
6	THF/DMF (v/v=5/1)	64	13	DME/DMAc (v/v=5/1)	47
7	THF/DMAc (v/v=5/1)	37			

[a] Isolated yields. NMP = 1-Methyl-2-pyrrolidinone. DMAc = *N*,*N*-Dimethylacetamide. DMF = *N*,*N*-Dimethylformamide. DMSO = Dimethyl sulfoxide. THF = Tetrahydrofuran. Diglyme = 2-Methoxyethyl ether. DME = 1,2-Dimethoxyethane.

Supplementary Table 5. Silane screening for the coupling of 1 and 2.

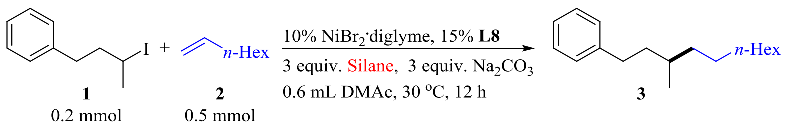

Entry	Silane	GC yield of **3** (%)	Entry	Silane	GC yield of **3** (%)
1	$(EtO)_3SiH$	12	4	Ph_2SiH_2	22
2	**DEMS**	***96 (93[a])***	5	PMHS	57
3	$PhSiH_3$	6	6	Et_3SiH	13

[a] Isolated yields. DEMS = Diethoxymethylsilane. PMHS = Polymethylhydrosiloxane.

Supplementary Discussion

TEMPO Radical Inhibite Reaction

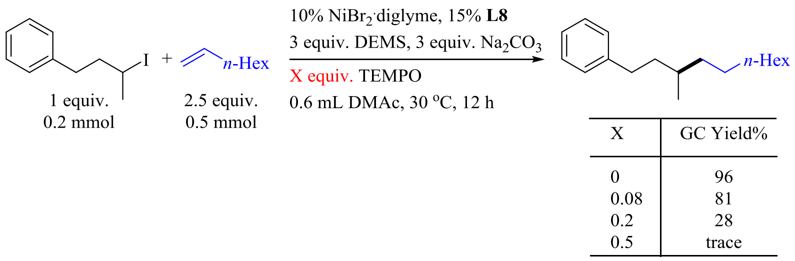

X	GC Yield%
0	96
0.08	81
0.2	28
0.5	trace

$NiBr_2$·diglyme (0.02 mmol, 10 mol%), 4,4'-di-*tert*-butyl-2,2'-bipyridine (0.03 mmol, 15 mol%), Na_2CO_3 (0.6 mmol, 3 equiv.) and X equiv. of TEMPO (2,2,6,6-Tetramethylpiperidinooxy) were added to a Schlenk tube equipped with a stir bar. The vessel was evacuated and filled with argon (three cycles). To these solids, 0.6 mL DMAc (*N*,*N*-Dimethylacetamide) was added under argon atmosphere. The reaction mixture was stirred at room temperature for 30 seconds. To the reaction mixture, electrophile (0.2 mmol, 1.0 equiv.), alkene (0.5 mmol, 2.5 equiv.) and DEMS (Diethoxymethylsilane) (0.6 mmol, 3 equiv.) were added under a positive flow of argon. The reaction mixture was stirred at 30 ℃ for 12 hours. The yield was determined by GC. The reaction was largely inhibited when 0.2 equiv. TEMPO was added.

Supplementary Methods

Materials and analytical methods.

All the reactions were carried out in oven-dried Schlenk tubes under argon atmosphere. The following chemicals were purchased and used as received: $NiBr_2$·diglyme (Aldrich), 4,4'-Di-*tert*-butyl-2,2'-bipyridine (Aldrich), Diethoxymethylsilane (Adamas), Na_2CO_3 (Sinopharm Chemical Reagent Co., Ltd), $KHCO_3$ (Sinopharm Chemical Reagent Co., Ltd), Iodocyclohexane (Alfa-Aesar), 1-Octene (Acros), 4-Phenyl-1-butene (TCI), 1,2-Epoxy-9-decene (J&K), 6-Hepten-3-ol (Alfa-Aesar), Ethyl 4-iodobenzoate (J&K), 4'-Iodoacetophenone (J&K), 2-Methyl-3-buten-2-ol (J&K), Calciferol (TCI), Sclareol (Heowns), Pleuromulin (J&K), Cyclohexene (J&K), Ethylene (Energy Chemical). *N,N*-Dimethylacetamide were purchased from Aldrich (anhydrous in a Sure-Seal® bottle).

^{1}H-NMR and ^{13}C-NMR spectra were recorded on a Bruker Avance 400 spectrometer at ambient temperature in $CDCl_3$ unless otherwise noted. Data for ^{1}H-NMR are reported as follows: chemical shift (δ ppm), multiplicity, coupling constant (Hz), and integration. Data for ^{13}C-NMR are reported in terms of chemical shift (δ ppm), multiplicity, and coupling constant (Hz). Gas chromatographic (GC) analysis was acquired on a Shimadzu GC-2014 Series GC system equipped with a flame-ionization detector. Organic solutions were concentrated under reduced pressure on a Buchi rotary evaporator. Flash column chromatographic purification of products was accomplished using forced-flow chromatography on Silica Gel (200-300 mesh). High performance liquid chromatography (HPLC) analysis were performed on Shimadzu instrument, using Daicel chiral columns.

General procedure for the synthesis of alkyl iodides.

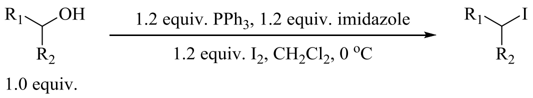

To a solution of the PPh_3 (1.2 equiv.), Imidazole (1.2 equiv.) and I_2 (1.2 equiv.) in dry CH_2Cl_2 (10 mL/ 0.8 mmol) was added a solution of corresponding alcohol (1 equiv.) in CH_2Cl_2 (15 mL/ 200 mg) at 0 ℃ via syringe. The reaction was stirred for additional 5 hours at room temperature. The mixture was washed with a solution of Na_2SO_3, H_2O, brine, dried over Na_2SO_4, filtered and concentrated under reduced pressure. The resulting iodide was purified by flash chromatography or recrystallization.

General procedure for the synthesis of alkyl tosylates.

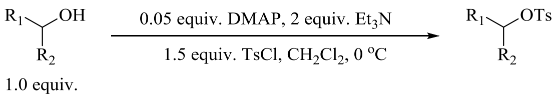

To a solution of corresponding alcohol (1 equiv.) in CH_2Cl_2 (15 mL/200 mg), Triethylamine (2 equiv.) and 4-(Dimethylamino)pyridine (0.05 equiv.) were added. The reaction mixture was cooled to 0 ℃, and added Tosyl chloride (1.5 equiv.) over 15 minutes. The reaction mixture was stirred for additional 5 hours at room temperature. After the reaction mixture was neutralized with ammonium chloride, the mixture was extracted with ethyl acetate, washed with saturated NaCl solution, dried over Na_2SO_4, filtered and concentrated under reduced pressure. The resulting mixture was isolated by flash chromatography or recrystallization.

General procedure for the synthesis of optical pure secondary alkyl bromides. [1]

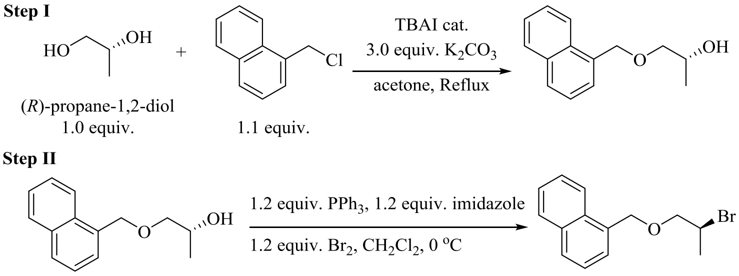

Step I: To a solution of (*R*)-propane-1,2-diol (1.0 equiv.) in acetone (3 mL/1 mmol) were added K_2CO_3 (3.0 equiv.), TBAI (30 mg/ 10 mmol) and 1-(chloromethyl)naphthalene (1.1 equiv.). The reaction mixture was refluxed for 12 h. The mixture was concentrated and dissolved in water. Extracted with ethyl acetate, washed with saturated NaCl solution, dried over Na_2SO_4, filtered and concentrated. The resulting mixture was purified by flash chromatography.

Step II: To a solution of the PPh_3 (1.2 equiv.), imidazole (1.2 equiv.) and Br_2 (1.2 equiv.) in dry CH_2Cl_2 (10 mL/ 0.8 mmol) was added a solution of corresponding alcohol (1 equiv.) in CH_2Cl_2 (15 mL/ 200 mg) at 0 ^{o}C via syringe. The reaction was stirred for additional 2 hours at room temperature. The mixture was washed with a solution of Na_2SO_3, H_2O, brine, dried over Na_2SO_4, filtered and concentrated under reduced pressure. The resulting bromide was purified by flash chromatography.

General procedure for the synthesis of alkenes. [2]

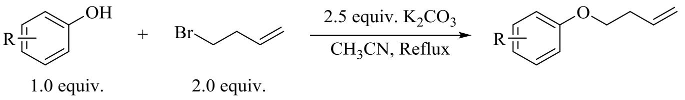

To a solution of phenol (1 equiv.) and K_2CO_3 (2.5 equiv.) in CH_3CN (5 mL/ 200 mg) was added 4-bromobut-1-ene (2 equiv.), and the mixture was refluxed for 12 hours. The reaction mixture was then cooled to room temperature, and the solvent was removed in vacuum. The residue was partitioned between CH_2Cl_2 and water, and the aqueous layer was extracted with CH_2Cl_2. The combined organic extracts were washed with water, dried over Na_2SO_4, and removed in vacuum. The resulting mixture was isolated by flash chromatography or recrystallization.

Experimental procedures for examples described in Table 1, Main Text.

$NiBr_2$·diglyme (0.02 mmol, 10 mol%), ligand (0.03 mmol, 15 mol%) and base (0.6 mmol, 3 equiv.) were added to a Schlenk tube equipped with a stir bar. The vessel was evacuated and filled with argon (three cycles). To these solids, 0.6 mL solvent was added under argon atmosphere. The reaction mixture was stirred at room temperature for 30 seconds. To the reaction mixture, **1** (0.2 mmol, 1.0 equiv.), **2** (0.5 mmol, 2.5 equiv.) and silane (0.6 mmol, 3 equiv.) were added under a positive flow of argon. The reaction mixture was stirred at 30 ℃ for 12 hours. The yield was determined by GC.

Experimental procedures for examples described in Table 2, Main Text.

Gengeral procedure A: $NiBr_2$·diglyme (0.02 mmol, 10 mol%), 4,4'-di-*tert*-butyl-2,2'-bipyridine (0.03 mmol, 15 mol%) and Na_2CO_3 (0.4 mmol, 2 equiv.) were added to a Schlenk tube equipped with a stir bar. The vessel was evacuated and filled with argon (three cycles). To these solids, 0.6 mL DMAc (*N*,*N*-Dimethylacetamide) was added under argon atmosphere. The reaction mixture was stirred at room temperature for 30 seconds. To the reaction mixture, electrophile (0.2 mmol, 1.0 equiv.), alkene

(0.3 mmol, 1.5 equiv.) and DEMS (Diethoxymethylsilane) (0.4 mmol, 2 equiv.) were added under a positive flow of argon. The reaction mixture was stirred at 30 ℃ for 12 hours. In order to remove the DMAc, the reaction mixture was poured into 50 mL of ice water and the resulting mixture was extracted with ethyl acetate (4 x 30 mL). The combined organic layer was dried over Na_2SO_4, filtered, concentrated in vacuum and purified by column chromatography.

Gengeral procedure B: $NiBr_2$·diglyme (0.02 mmol, 10 mol%), 4,4'-di-*tert*-butyl-2,2'-bipyridine (0.03 mmol, 15 mol%) and Na_2CO_3 (0.6 mmol, 3 equiv.) were added to a Schlenk tube equipped with a stir bar. The vessel was evacuated and filled with argon (three cycles). To these solids, 0.6 mL DMAc (*N*,*N*-Dimethylacetamide) was added under argon atmosphere. The reaction mixture was stirred at room temperature for 30 seconds. To the reaction mixture, electrophile (0.2 mmol, 1.0 equiv.), alkene (0.5 mmol, 2.5 equiv.) and DEMS (Diethoxymethylsilane) (0.6 mmol, 3 equiv.) were added under a positive flow of argon. The reaction mixture was stirred at 30 ℃ for 12 hours. In order to remove the DMAc, the reaction mixture was poured into 50 mL of ice water and the resulting mixture was extracted with ethyl acetate (4 x 30 mL). The combined organic layer was dried over Na_2SO_4, filtered, concentrated in vacuum and purified by column chromatography.

Experimental data for the described substances.

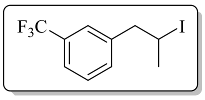

1-(2-iodopropyl)-3-(trifluoromethyl)benzene: Flash chromatography (Petroleum ether) as colorless oil.

^{1}H NMR (400 MHz, CDCl$_3$) δ 7.54 (d, *J* = 7.6 Hz, 1H), 7.48 – 7.36 (m, 3H), 4.40 – 4.25 (m, 1H), 3.22 (ddd, *J* = 21.1, 14.2, 7.3 Hz, 2H), 1.93 (d, *J* = 6.8 Hz, 3H).

^{13}C NMR (101 MHz, CDCl$_3$) δ 140.56, 132.55, 130.94 (q, *J* = 32.2 Hz), 129.04, 125.83 (q, *J* = 3.8 Hz), 124.22 (q, *J* = 272.3 Hz), 123.89 (q, *J* = 3.8 Hz), 49.04, 28.32, 27.30.

^{19}F NMR (376 MHz, CDCl$_3$) δ -62.58.

HRMS (ESI) calcd for $C_{10}H_{10}F_3INa^+$ [$(M+Na)^+$] 336.9672, found 336.9663.

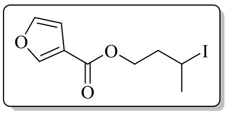

3-iodobutyl furan-3-carboxylate: Flash chromatography (EtOAc: Petroleum ether = 1:20) as colorless oil.

^{1}H NMR (400 MHz, CDCl$_3$) δ 8.00 (d, *J* = 0.7 Hz, 1H), 7.42 (t, *J* = 1.7 Hz, 1H), 6.78 – 6.67 (m, 1H), 4.49 – 4.40 (m, 1H), 4.35 – 4.21 (m, 2H), 2.28 – 2.13 (m, 1H), 2.12 – 2.01 (m, 1H), 1.98 (d, *J* = 6.9 Hz, 3H).

^{13}C NMR (101 MHz, CDCl$_3$) δ 162.96, 147.88, 143.90, 119.27, 109.89, 64.29, 41.44, 29.07, 24.19.

HRMS (APCI) calcd for $C_9H_{12}O_3I^+$ [$(M+H)^+$] 294.9826, found 294.9824.

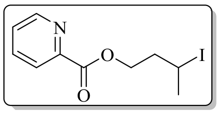

3-iodobutyl picolinate: Flash chromatography (EtOAc: Petroleum ether = 1:3) as yellow oil.

^{1}H NMR (400 MHz, CDCl$_3$) δ 8.73 (d, *J* = 4.1 Hz, 1H), 8.09 (d, *J* = 7.8 Hz, 1H), 7.82 (td, *J* = 7.7, 1.6 Hz, 1H), 7.46 (ddd, *J* = 7.5, 4.8, 0.8 Hz, 1H), 4.63 – 4.38 (m, 2H), 4.37 – 4.19 (m, 1H), 2.37 – 2.07 (m, 2H), 1.97 (d, *J* = 6.9 Hz, 3H).

^{13}C NMR (101 MHz, CDCl$_3$) δ 165.03, 149.93, 147.84, 137.18, 127.08, 125.29, 65.75, 41.25, 29.05, 23.99.

HRMS (APCI) calcd for $C_{10}H_{13}O_2NI^+$ [(M+H)$^+$] 305.9986, found 305.9983.

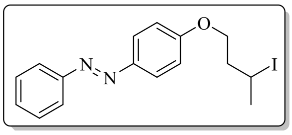

(*E*)-1-(4-(3-iodobutoxy)phenyl)-2-phenyldiazene: Flash chromatography (EtOAc: Petroleum ether = 1:100) as orange solid.

^{1}H NMR (400 MHz, CDCl$_3$) δ 7.96 – 7.91 (m, 2H), 7.90 – 7.86 (m, 2H), 7.55 – 7.47 (m, 2H), 7.47 – 7.41 (m, 1H), 7.05 – 6.99 (m, 2H), 4.45 (dqd, *J* = 13.8, 6.9, 4.4 Hz, 1H), 4.25 – 4.10 (m, 2H), 2.34 – 2.23 (m, 1H), 2.20 – 2.09 (m, 1H), 2.04 (d, *J* = 6.9 Hz, 3H).

^{13}C NMR (101 MHz, CDCl$_3$) δ 161.28, 152.89, 147.26, 130.55, 129.18, 124.92, 122.71, 114.89, 68.09, 42.04, 29.25, 25.27.

HRMS (APCI) calcd for $C_{16}H_{18}ON_2I^+$ [(M+H)$^+$] 381.0458, found 381.0458.

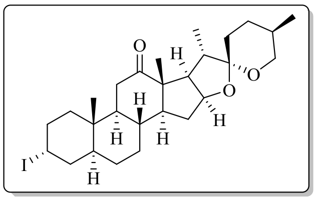

(2a*S*,4*R*,5'*R*,6a*S*,6b*S*,8a*S*,8b*R*,9*S*,10*R*,11a*S*,12a*S*,12b*R*)-4-iodo-5',6a,8a,9-tetramethylicosahydrospiro[naphtho[2',1':4,5]indeno[2,1-*b*]furan-10,2'-pyran]-8(2*H*)-one: Flash chromatography (EtOAc: Petroleum ether = 1:15) as white solid.

^{1}H NMR (400 MHz, CDCl$_3$) δ 4.90 (s, 1H), 4.46 – 4.23 (m, 1H), 3.47 (d, *J* = 8.4 Hz, 1H), 3.33 (t, *J* = 10.9 Hz, 1H), 2.59 – 2.45 (m, 1H), 2.35 (t, *J* = 13.7 Hz, 1H), 2.24 (dd, *J* = 14.2, 5.0 Hz, 1H), 2.11 (t, *J* = 6.9 Hz, 1H), 1.91 (d, *J* = 13.4 Hz, 2H), 1.83 – 1.26 (m, 18H), 1.14 – 0.98 (m, 7H), 0.87 (s, 3H), 0.77 (d, *J* = 6.3 Hz, 3H).

^{13}C NMR (101 MHz, CDCl$_3$) δ 213.38, 109.33, 79.29, 66.98, 55.80, 55.19, 55.03, 53.62, 42.30, 42.00, 38.64, 37.48, 37.19, 36.72, 34.37, 33.98, 32.54, 31.52, 31.41, 31.18, 30.30, 28.89, 27.45, 17.25, 16.14, 13.37, 13.13.

HRMS (APCI) calcd for $C_{27}H_{42}O_3I^+$ [(M+H)$^+$] 541.2173, found 541.2173.

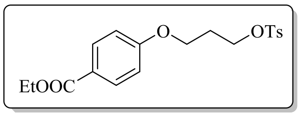

ethyl 4-(3-(tosyloxy)propoxy)benzoate: Flash chromatography (EtOAc: Petroleum ether = 1:3) as white solid.

^{1}H NMR (400 MHz, CDCl$_3$) δ 7.95 (d, *J* = 8.8 Hz, 2H), 7.73 (d, *J* = 8.2 Hz, 2H), 7.22 (d, *J* = 8.3 Hz,

2H), 6.75 (d, J = 8.8 Hz, 2H), 4.35 (q, J = 7.1 Hz, 2H), 4.24 (t, J = 5.9 Hz, 2H), 3.98 (t, J = 5.8 Hz, 2H), 2.35 (s, 3H), 2.13 (p, J = 5.8 Hz, 2H), 1.38 (t, J = 7.1 Hz, 3H).

^{13}C NMR (101 MHz, CDCl$_3$) δ 166.41, 162.19, 144.99, 132.76, 131.57, 129.94, 127.93, 123.24, 113.98, 66.84, 63.26, 60.81, 28.81, 21.72, 14.51.

HRMS (APCI) calcd for $C_{19}H_{23}O_6S^+$ [(M+H)$^+$] 379.1210, found 379.1210.

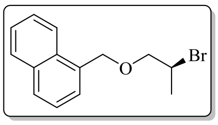

(*S*)-1-((2-bromopropoxy)methyl)naphthalene: Flash chromatography (EtOAc: Petroleum ether = 1:50) as colorless oil.

^{1}H NMR (400 MHz, CDCl$_3$) δ 8.14 (dd, J = 8.1, 0.9 Hz, 1H), 7.91 – 7.75 (m, 2H), 7.58 – 7.38 (m, 4H), 5.13 – 4.92 (m, 2H), 4.31 – 4.06 (m, 1H), 3.68 (ddd, J = 44.9, 10.2, 6.4 Hz, 2H), 1.66 (d, J = 6.7 Hz, 3H).

^{13}C NMR (101 MHz, CDCl$_3$) δ 133.91, 133.39, 131.83, 128.98, 128.67, 126.70, 126.37, 125.99, 125.28, 124.23, 75.76, 71.94, 46.93, 22.87.

HRMS (APCI) calcd for $C_{14}H_{16}OBr^+$ [(M+H)$^+$] 279.0379, found 279.0377.

HPLC analysis: The *ee* value was determined to be 99% by HPLC analysis on a Chiralpak OD-H column, λ = 254 nm, *n*-hexane/*i*-PrOH (99.5:0.5), flow rate = 1.0 mL/min; t_R = 12.0 min (major), 12.9 min (minor).

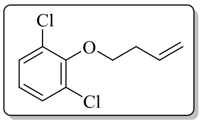

2-(but-3-en-1-yloxy)-1,3-dichlorobenzene: Flash chromatography (EtOAc: Petroleum ether = 1:100) as colorless oil.

^{1}H NMR (400 MHz, CDCl$_3$) δ 7.28 (d, J = 8.1 Hz, 2H), 6.98 (t, J = 8.1 Hz, 1H), 5.97 (ddt, J = 17.0, 10.2, 6.7 Hz, 1H), 5.21 (dd, J = 17.2, 1.6 Hz, 1H), 5.12 (d, J = 10.2 Hz, 1H), 4.07 (t, J = 6.8 Hz, 2H), 2.63 (q, J = 6.8 Hz, 2H).

^{13}C NMR (101 MHz, CDCl$_3$) δ 151.72, 134.40, 129.71, 129.00, 125.09, 117.17, 72.76, 34.58.

HRMS (APCI) calcd for $C_{10}H_{11}OCl_2^+$ [(M+H)$^+$] 217.0182, found 217.0178.

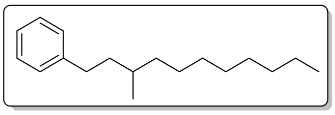

(3-methylundecyl)benzene: Following general procedure A, the product was isolated by flash chromatography (Petroleum ether) as colorless oil (40.5 mg, 82%). Following general procedure B, the product was isolated by flash chromatography (Petroleum ether) as colorless oil (45.7 mg, 93%).

^{1}H NMR (400 MHz, CDCl$_3$) δ 7.30 – 7.22 (m, 2H), 7.22 – 7.11 (m, 3H), 2.73 – 2.48 (m, 2H), 1.69 – 1.56 (m, 1H), 1.50 – 1.38 (m, 2H), 1.37 – 1.19 (m, 13H), 1.20 – 1.09 (m, 1H), 0.92 (d, J = 6.3 Hz, 3H), 0.88 (t, J = 6.8 Hz, 3H).

^{13}C NMR (101 MHz, CDCl$_3$) δ 143.37, 128.49, 128.39, 125.65, 39.14, 37.07, 33.65, 32.65, 32.09, 30.16, 29.84, 29.52, 27.13, 22.86, 19.78, 14.29.

HRMS (APCI) calcd for $C_{18}H_{31}^+$ [(M+H)$^+$] 247.2420, found 247.2415.

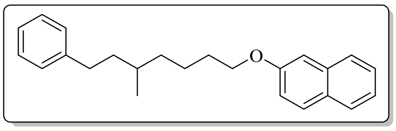

2-((5-methyl-7-phenylheptyl)oxy)naphthalene: Following general procedure A, the product was isolated by flash chromatography (Petroleum ether) as colorless oil (43.8 mg, 66%).

^{1}H NMR (400 MHz, CDCl$_3$) δ 7.79 – 7.69 (m, 3H), 7.46 – 7.39 (m, 1H), 7.35 – 7.25 (m, 3H), 7.21 – 7.11 (m, 5H), 4.07 (t, *J* = 6.5 Hz, 2H), 2.75 – 2.51 (m, 2H), 1.91 – 1.76 (m, 2H), 1.72 – 1.61 (m, 1H), 1.59 – 1.39 (m, 5H), 1.33 – 1.20 (m, 1H), 0.96 (d, *J* = 6.2 Hz, 3H).

^{13}C NMR (101 MHz, CDCl$_3$) δ 157.22, 143.21, 134.75, 129.44, 129.00, 128.49, 128.42, 127.77, 126.82, 126.42, 125.70, 123.59, 119.17, 106.66, 68.10, 39.04, 36.79, 33.62, 32.61, 29.69, 23.67, 19.70.

HRMS (APCI) calcd for $C_{24}H_{29}O^+$ [(M+H)$^+$] 333.2213, found 333.2214.

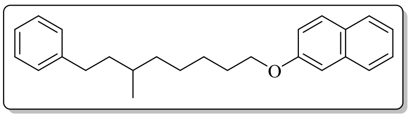

2-((6-methyl-8-phenyloctyl)oxy)naphthalene: Following general procedure A, the product was isolated by flash chromatography (Petroleum ether) as colorless oil (39.4 mg, 57%).

^{1}H NMR (400 MHz, CDCl$_3$) δ 7.71 – 7.59 (m, 3H), 7.33 (t, *J* = 7.3 Hz, 1H), 7.27 – 7.14 (m, 3H), 7.07 (dt, *J* = 19.0, 6.5 Hz, 5H), 3.96 (t, *J* = 6.5 Hz, 2H), 2.64 – 2.42 (m, 2H), 1.83 – 1.70 (m, 2H), 1.66 – 1.48 (m, 1H), 1.47 – 1.06 (m, 8H), 0.86 (d, *J* = 6.1 Hz, 3H).

^{13}C NMR (101 MHz, CDCl$_3$) δ 157.21, 143.25, 134.74, 129.43, 128.99, 128.47, 128.39, 127.76, 126.81, 126.40, 125.67, 123.57, 119.16, 106.64, 68.09, 39.07, 36.95, 33.62, 32.58, 29.40, 26.89, 26.56, 19.74.

HRMS (APCI) calcd for $C_{25}H_{31}O^+$ [(M+H)$^+$] 347.2369, found 347.2367.

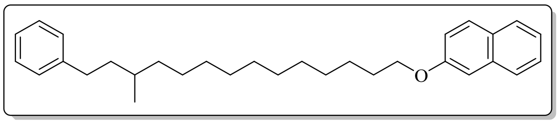

2-((12-methyl-14-phenyltetradecyl)oxy)naphthalene: Following general procedure A, the product was isolated by flash chromatography (Petroleum ether) as colorless oil (60.2 mg, 70%).

^{1}H NMR (400 MHz, CDCl$_3$) δ 7.80 – 7.66 (m, 3H), 7.42 (t, *J* = 7.4 Hz, 1H), 7.35 – 7.23 (m, 3H), 7.20 – 7.09 (m, 5H), 4.06 (t, *J* = 6.6 Hz, 2H), 2.76 – 2.46 (m, 2H), 1.92 – 1.79 (m, 2H), 1.71 – 1.12 (m, 21H), 0.92 (d, *J* = 6.1 Hz, 3H).

^{13}C NMR (101 MHz, CDCl$_3$) δ 157.24, 143.36, 134.75, 129.42, 128.99, 128.48, 128.38, 127.76, 126.81, 126.40, 125.64, 123.57, 119.17, 106.64, 68.14, 39.12, 37.06, 33.64, 32.64, 30.14, 29.85, 29.81, 29.76, 29.75, 29.58, 29.40, 27.12, 26.26, 19.77.

HRMS (APCI) calcd for $C_{31}H_{43}O^+$ [(M+H)$^+$] 431.3308, found 431.3305.

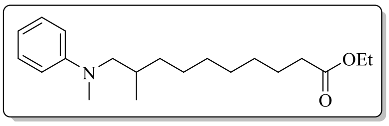

ethyl 9-methyl-10-(methyl(phenyl)amino)decanoate: Following general procedure A, the product was isolated by flash chromatography (EtOAc: Petroleum ether = 1:20) as colorless oil (42.7 mg, 67%).

^{1}H NMR (400 MHz, CDCl$_3$) δ 7.28 – 7.18 (m, 2H), 6.74 – 6.60 (m, 3H), 4.13 (q, *J* = 7.1 Hz, 2H), 3.12 (ddd, *J* = 22.6, 14.5, 7.3 Hz, 2H), 2.95 (s, 3H), 2.29 (t, *J* = 7.5 Hz, 2H), 1.95 – 1.84 (m, 1H), 1.67 – 1.57 (m, 2H), 1.43 – 1.21 (m, 12H), 1.17 – 1.04 (m, 1H), 0.90 (d, *J* = 6.6 Hz, 3H).

^{13}C NMR (101 MHz, CDCl$_3$) δ 174.03, 149.80, 129.18, 115.65, 111.88, 60.29, 59.99, 39.61, 34.78, 34.50, 32.33, 29.91, 29.37, 29.24, 27.11, 25.09, 17.89, 14.39.

HRMS (APCI) calcd for $C_{20}H_{34}O_2N^+$ [(M+H)$^+$] 320.2584, found 320.2579.

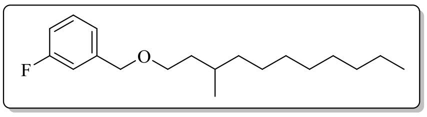

1-fluoro-3-(((3-methylundecyl)oxy)methyl)benzene: Following general procedure B, the product was isolated by flash chromatography (EtOAc: Petroleum ether = 1:100) as colorless oil (33.5 mg, 57%).

^{1}H NMR (400 MHz, CDCl$_3$) δ 7.33 – 7.25 (m, 1H), 7.13 – 7.03 (m, 2H), 6.96 (td, *J* = 8.5, 2.4 Hz, 1H), 4.49 (s, 2H), 3.57 – 3.44 (m, 2H), 1.73 – 1.61 (m, 1H), 1.61 – 1.52 (m, 1H), 1.49 – 1.37 (m, 1H), 1.35 – 1.19 (m, 13H), 1.16 – 1.07 (m, 1H), 0.96 – 0.80 (m, 6H).

^{13}C NMR (101 MHz, CDCl$_3$) δ 163.10 (d, *J* = 245.6 Hz), 141.60 (d, *J* = 7.1 Hz), 129.94 (d, *J* = 8.2 Hz), 122.98 (d, *J* = 2.8 Hz), 114.39 (d, *J* = 21.4 Hz), 72.26 (d, *J* = 1.8 Hz), 69.15, 37.25, 36.88, 32.07, 30.10, 29.99, 29.81, 29.50, 27.10, 22.84, 19.82, 14.28.

^{19}F NMR (376 MHz, CDCl$_3$) δ -113.46.

HRMS (APCI) calcd for $C_{19}H_{32}OF^+$ [(M+H)$^+$] 295.2432, found 295.2429.

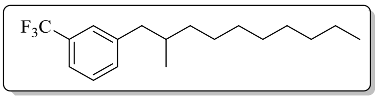

1-(2-methyldecyl)-3-(trifluoromethyl)benzene: Following general procedure B, the product was isolated by flash chromatography (Petroleum ether) as colorless oil (55.2 mg, 92%).

^{1}H NMR (400 MHz, CDCl$_3$) δ 7.47 – 7.27 (m, 4H), 2.54 (ddd, *J* = 21.7, 13.4, 7.2 Hz, 2H), 1.72 (dd, *J* = 11.1, 6.2 Hz, 1H), 1.40 – 1.09 (m, 14H), 0.88 (t, *J* = 6.8 Hz, 3H), 0.84 (d, *J* = 6.6 Hz, 3H).

^{13}C NMR (101 MHz, CDCl$_3$) δ142.72, 132.70, 130.54 (q, *J* = 31.8 Hz), 128.59, 125.91 (q, *J* = 3.7 Hz), 124.46 (q, *J* = 272.2 Hz), 122.66 (q, *J* = 3.8 Hz), 43.60, 36.79, 35.09, 32.07, 30.01, 29.79, 29.49, 27.20, 22.84, 19.42, 14.27.

^{19}F NMR (376 MHz, CDCl$_3$) δ -62.51.

HRMS (EI) calcd for $C_{18}H_{27}F_3$ (M) 300.2065, found 300.2046.

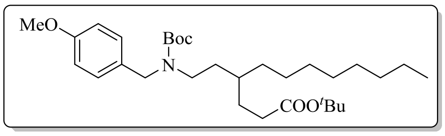

***tert*-butyl 4-(2-((*tert*-butoxycarbonyl)(4-methoxybenzyl)amino)ethyl)dodecanoate:** Following general procedure B, the product was isolated by flash chromatography (EtOAc: Petroleum ether = 1:15) as colorless oil (46.8 mg, 45%).

^{1}H NMR (400 MHz, CDCl$_3$) δ 7.15 (s, 2H), 6.93 – 6.72 (m, 2H), 4.34 (s, 2H), 3.78 (s, 3H), 3.11 (d, *J* = 34.2 Hz, 2H), 2.15 (t, *J* = 7.9 Hz, 1H), 1.55 – 1.36 (m, 22H), 1.31 – 1.16 (m, 16H), 0.87 (t, *J* = 6.9 Hz, 3H).

^{13}C NMR (101 MHz, CDCl$_3$) δ 173.25, 158.80, 130.64, 129.15, 113.84, 79.96, 79.44, 55.22, 49.27, 44.13, 35.01, 33.22, 32.90, 31.88, 31.29, 29.96, 29.59, 29.33, 28.73, 28.51, 28.10, 26.52, 22.66, 14.09.

HRMS (APCI) calcd for $C_{31}H_{54}O_5N^+$ [(M+H)$^+$] 520.3997, found 520.3992.

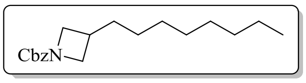

benzyl 3-octylazetidine-1-carboxylate: Following general procedure B, the product was isolated by flash chromatography (EtOAc: Petroleum ether = 1:8) as colorless oil (33.3 mg, 55%).

^{1}H NMR (400 MHz, CDCl$_3$) δ 7.39 – 7.27 (m, 5H), 5.09 (s, 2H), 4.07 (t, *J* = 8.4 Hz, 2H), 3.61 (dd, *J* = 8.5, 5.6 Hz, 2H), 2.63 – 2.43 (m, 1H), 1.57 (dd, *J* = 14.1, 7.4 Hz, 2H), 1.35 – 1.18 (m, 12H), 0.88 (t, *J* = 6.8 Hz, 3H).

^{13}C NMR (101 MHz, CDCl$_3$) δ 156.58, 136.96, 128.56, 128.06, 128.03, 66.57, 55.21, 54.56, 34.50, 31.96, 29.62, 29.51, 29.48, 29.34, 27.05, 22.78, 14.22.

HRMS (APCI) calcd for $C_{19}H_{30}O_2N^+$ [(M+H)$^+$] 304.2271, found 304.2272.

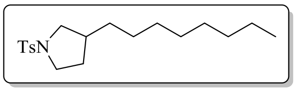

3-octyl-1-tosylpyrrolidine: Following general procedure B, the product was isolated by flash chromatography (EtOAc: Petroleum ether = 1:8) as colorless oil (59.4 mg, 88%).

^{1}H NMR (400 MHz, CDCl$_3$) δ 7.69 (d, *J* = 8.2 Hz, 2H), 7.30 (d, *J* = 8.0 Hz, 2H), 3.41 (dd, *J* = 9.6, 7.4 Hz, 1H), 3.37 – 3.26 (m, 1H), 3.22 – 3.12 (m, 1H), 2.80 – 2.70 (m, 1H), 2.42 (s, 3H), 2.01 – 1.83 (m, 2H), 1.40 – 1.12 (m, 15H), 0.86 (t, *J* = 6.9 Hz, 3H).

^{13}C NMR (101 MHz, CDCl$_3$) δ 143.33, 133.98, 129.68, 127.59, 53.38, 47.68, 38.93, 33.16, 31.93, 31.60, 29.69, 29.54, 29.32, 28.17, 22.74, 21.61, 14.19.

HRMS (APCI) calcd for $C_{19}H_{32}O_2NS^+$ [(M+H)$^+$] 338.2148, found 338.2146.

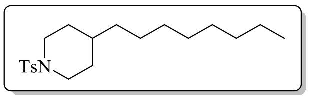

4-octyl-1-tosylpiperidine: Following general procedure B, the product was isolated by flash chromatography (EtOAc: Petroleum ether = 1:8) as white solid (44.1 mg, 63%).

^{1}H NMR (400 MHz, CDCl$_3$) δ 7.62 (d, *J* = 8.2 Hz, 2H), 7.30 (d, *J* = 8.1 Hz, 2H), 3.74 (d, *J* = 11.5 Hz, 2H), 2.42 (s, 3H), 2.18 (t, *J* = 11.8 Hz, 2H), 1.69 (d, *J* = 12.4 Hz, 2H), 1.37 – 1.05 (m, 17H), 0.85 (t, *J* = 6.8 Hz, 3H).

^{13}C NMR (101 MHz, CDCl$_3$) δ 143.40, 133.25, 129.63, 127.84, 46.67, 36.16, 35.19, 31.97, 31.65, 29.81, 29.66, 29.39, 26.67, 22.76, 21.63, 14.21.

HRMS (APCI) calcd for $C_{20}H_{34}O_2NS^+$ [(M+H)$^+$] 352.2305, found 352.2303.

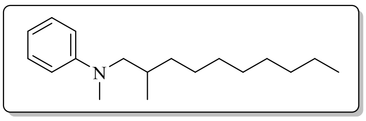

***N*-methyl-*N*-(2-methyldecyl)aniline:** Following general procedure B, the product was isolated by flash chromatography (Petroleum ether) as colorless oil (46.4 mg, 89%).

^{1}H NMR (400 MHz, $CDCl_3$) δ 7.28 – 7.16 (m, 2H), 6.73 – 6.60 (m, 3H), 3.11 (ddd, *J* = 22.7, 14.5, 7.3 Hz, 2H), 2.94 (s, 3H), 1.98 – 1.82 (m, 1H), 1.46 – 1.18 (m, 13H), 1.17 – 1.00 (m, 1H), 0.95 – 0.80 (m, 6H).

^{13}C NMR (101 MHz, $CDCl_3$) δ 149.83, 129.19, 115.64, 111.89, 60.02, 39.62, 34.86, 32.36, 32.05, 30.16, 29.77, 29.48, 27.21, 22.83, 17.94, 14.27.

HRMS (APCI) calcd for $C_{18}H_{32}N^+$ $[(M+H)^+]$ 262.2529, found 262.2526.

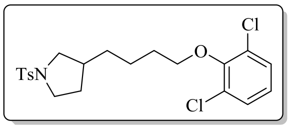

3-(4-(2,6-dichlorophenoxy)butyl)-1-tosylpyrrolidine: Following general procedure B, the product was isolated by flash chromatography (EtOAc: Petroleum ether = 1:5) as colorless oil (44.2 mg, 50%).

^{1}H NMR (400 MHz, $CDCl_3$) δ 7.72 (d, *J* = 8.2 Hz, 2H), 7.32 (d, *J* = 8.0 Hz, 2H), 7.28 (d, *J* = 8.1 Hz, 2H), 6.98 (t, *J* = 8.1 Hz, 1H), 3.96 (t, *J* = 6.3 Hz, 2H), 3.46 (dd, *J* = 9.6, 7.3 Hz, 1H), 3.39 – 3.28 (m, 1H), 3.26 – 3.16 (m, 1H), 2.80 (dd, *J* = 9.6, 8.2 Hz, 1H), 2.43 (s, 3H), 2.13 – 1.88 (m, 2H), 1.83 – 1.70 (m, 2H), 1.57 – 1.23 (m, 5H).

^{13}C NMR (101 MHz, $CDCl_3$) δ 151.69, 143.41, 133.97, 129.74, 129.64, 129.04, 127.65, 125.09, 73.27, 53.40, 47.72, 38.95, 32.94, 31.58, 30.13, 24.60, 21.66.

HRMS (APCI) calcd for $C_{21}H_{26}O_3NCl_2S^+$ $[(M+H)^+]$ 442.1005, found 442.1002.

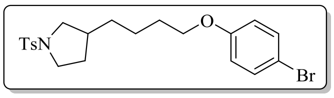

3-(4-(4-bromophenoxy)butyl)-1-tosylpyrrolidine: Following general procedure B, the product was isolated by flash chromatography (EtOAc: Petroleum ether = 1:4) as colorless oil (35.3 mg, 39%).

^{1}H NMR (400 MHz, $CDCl_3$) δ 7.71 (d, *J* = 8.2 Hz, 2H), 7.36 (d, *J* = 9.0 Hz, 2H), 7.32 (d, *J* = 8.0 Hz, 2H), 6.75 (d, *J* = 8.9 Hz, 2H), 3.87 (t, *J* = 6.3 Hz, 2H), 3.44 (dd, *J* = 9.6, 7.4 Hz, 1H), 3.40 – 3.29 (m, 1H), 3.27 – 3.12 (m, 1H), 2.86 – 2.73 (m, 1H), 2.43 (s, 3H), 2.09 – 1.87 (m, 2H), 1.78 – 1.64 (m, 2H), 1.50 – 1.21 (m, 5H).

^{13}C NMR (101 MHz, $CDCl_3$) δ 158.19, 143.44, 134.00, 132.36, 129.75, 127.66, 116.35, 112.86, 67.93, 53.33, 47.68, 38.91, 32.95, 31.59, 29.27, 24.78, 21.67.

HRMS (APCI) calcd for $C_{21}H_{27}O_3NBrS^+$ $[(M+H)^+]$ 452.0890, found 452.0888.

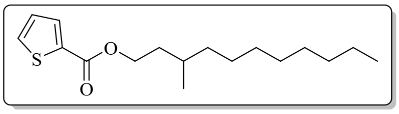

3-methylundecyl thiophene-2-carboxylate: Following general procedure A, the product was isolated by flash chromatography (EtOAc: Petroleum ether = 1:30) as colorless oil (47.4 mg, 80%).

^{1}H NMR (400 MHz, CDCl$_3$) δ 7.79 (dd, *J* = 3.7, 1.2 Hz, 1H), 7.54 (dd, *J* = 5.0, 1.2 Hz, 1H), 7.09 (dd, *J* = 4.9, 3.8 Hz, 1H), 4.37 – 4.28 (m, 2H), 1.85 – 1.71 (m, 1H), 1.66 – 1.48 (m, 2H), 1.35 – 1.15 (m, 14H), 0.94 (d, *J* = 6.5 Hz, 3H), 0.88 (t, *J* = 6.8 Hz, 3H).
^{13}C NMR (101 MHz, CDCl$_3$) δ 162.48, 134.27, 133.33, 132.27, 127.81, 63.91, 37.02, 35.64, 32.04, 30.05, 30.01, 29.77, 29.48, 27.03, 22.82, 19.75, 14.26.
HRMS (APCI) calcd for $C_{17}H_{29}O_2S^+$ [(M+H)$^+$] 297.1883, found 297.1882.

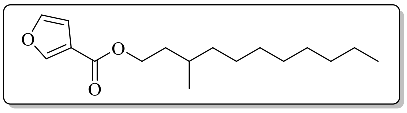

3-methylundecyl furan-3-carboxylate: Following general procedure A, the product was isolated by flash chromatography (EtOAc: Petroleum ether = 1:30) as colorless oil (33.0 mg, 59%).
^{1}H NMR (400 MHz, CDCl$_3$) δ 7.99 (s, 1H), 7.41 (t, *J* = 1.6 Hz, 1H), 6.73 (d, *J* = 1.3 Hz, 1H), 4.33 – 4.21 (m, 2H), 1.80 – 1.69 (m, 1H), 1.63 – 1.45 (m, 2H), 1.36 – 1.13 (m, 14H), 0.93 (d, *J* = 6.5 Hz, 3H), 0.87 (t, *J* = 6.8 Hz, 3H).
^{13}C NMR (101 MHz, CDCl$_3$) δ 163.38, 147.70, 143.77, 119.76, 109.96, 63.25, 37.04, 35.66, 32.04, 30.06, 30.03, 29.78, 29.48, 27.04, 22.82, 19.73, 14.25.
HRMS (APCI) calcd for $C_{17}H_{29}O_3^+$ [(M+H)$^+$] 281.2111, found 281.2111.

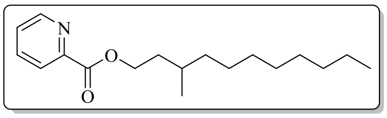

3-methylundecyl picolinate: 2.5 equiv. alkene, 3 equiv. DEMS, 3 equiv. $KHCO_3$, 0.6 mL DMAc were used, 30 ^{o}C, 24 hours. $KHCO_3$ was selected for the synthesis of this compound after slight modification based on general procedure. The product was isolated by flash chromatography (EtOAc: Petroleum ether = 1:3) as yellow oil (37.1 mg, 64%).
^{1}H NMR (400 MHz, CDCl$_3$) δ 8.77 (d, *J* = 3.9 Hz, 1H), 8.12 (d, *J* = 7.8 Hz, 1H), 7.84 (td, *J* = 7.7, 1.7 Hz, 1H), 7.47 (ddd, *J* = 7.6, 4.7, 1.1 Hz, 1H), 4.55 – 4.35 (m, 2H), 1.95 – 1.79 (m, 1H), 1.70 – 1.56 (m, 2H), 1.38 – 1.14 (m, 14H), 0.94 (d, *J* = 6.3 Hz, 3H), 0.87 (t, *J* = 6.8 Hz, 3H).
^{13}C NMR (101 MHz, CDCl$_3$) δ 165.38, 150.01, 148.39, 137.16, 126.94, 125.22, 64.83, 37.05, 35.62, 32.04, 30.06, 30.03, 29.77, 29.47, 27.00, 22.82, 19.75, 14.26.
HRMS (APCI) calcd for $C_{18}H_{30}O_2N^+$ [(M+H)$^+$] 292.2271, found 292.2267.

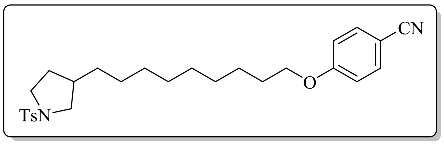

4-((9-(1-tosylpyrrolidin-3-yl)nonyl)oxy)benzonitrile: Following general procedure B, the product was isolated by flash chromatography (EtOAc: Petroleum ether = 1:4) as colorless oil (60.0 mg, 64%).
^{1}H NMR (400 MHz, CDCl$_3$) δ 7.70 (d, *J* = 8.2 Hz, 2H), 7.56 (d, *J* = 8.9 Hz, 2H), 7.31 (d, *J* = 8.0 Hz, 2H), 6.92 (d, *J* = 8.9 Hz, 2H), 3.98 (t, *J* = 6.5 Hz, 2H), 3.41 (dd, *J* = 9.5, 7.4 Hz, 1H), 3.37 – 3.27 (m, 1H), 3.22 – 3.12 (m, 1H), 2.76 (dd, *J* = 9.5, 8.1 Hz, 1H), 2.42 (s, 3H), 2.05 – 1.84 (m, 2H), 1.83 – 1.73 (m, 2H), 1.49 – 1.13 (m, 15H).
^{13}C NMR (101 MHz, CDCl$_3$) δ 162.54, 143.35, 134.05, 134.01, 129.69, 127.61, 119.44, 115.27,

103.70, 68.48, 53.38, 47.67, 38.93, 33.19, 31.62, 29.67, 29.54, 29.51, 29.38, 29.06, 28.19, 26.01, 21.64.
HRMS (APCI) calcd for $C_{27}H_{37}O_3N_2S^+$ [$(M+H)^+$] 469.2519, found 469.2520.

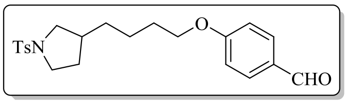

4-(4-(1-tosylpyrrolidin-3-yl)butoxy)benzaldehyde: Following general procedure B, the product was isolated by flash chromatography (EtOAc: Petroleum ether = 1:4) as colorless oil (38.5 mg, 48%).
^{1}H NMR (400 MHz, CDCl$_3$) δ 9.88 (s, 1H), 7.83 (d, *J* = 8.7 Hz, 2H), 7.71 (d, *J* = 8.2 Hz, 2H), 7.32 (d, *J* = 8.0 Hz, 2H), 6.97 (d, *J* = 8.6 Hz, 2H), 4.00 (t, *J* = 6.3 Hz, 2H), 3.44 (dd, *J* = 9.5, 7.4 Hz, 1H), 3.40 – 3.31 (m, 1H), 3.25 – 3.13 (m, 1H), 2.81 (dd, *J* = 9.5, 8.3 Hz, 1H), 2.43 (s, 3H), 2.12 – 1.89 (m, 2H), 1.83 – 1.68 (m, 2H), 1.48 – 1.28 (m, 5H).
^{13}C NMR (101 MHz, CDCl$_3$) δ 191.00, 164.18, 143.48, 133.92, 132.15, 129.93, 129.75, 127.63, 114.81, 68.10, 53.30, 47.65, 38.87, 32.92, 31.55, 29.15, 24.74, 21.66.
HRMS (APCI) calcd for $C_{22}H_{28}O_4NS^+$ [$(M+H)^+$] 402.1734, found 402.1736.

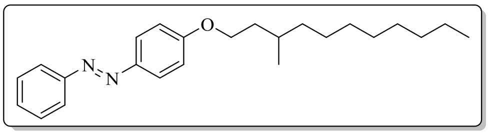

(*E*)-1-(4-((3-methylundecyl)oxy)phenyl)-2-phenyldiazene: Following general procedure B, the product was isolated by flash chromatography (EtOAc: Petroleum ether = 1:100) as orange solid (26.4 mg, 36%).
^{1}H NMR (400 MHz, CDCl$_3$) δ 7.95 – 7.90 (m, 2H), 7.90 – 7.85 (m, 2H), 7.54 – 7.47 (m, 2H), 7.46 – 7.41 (m, 1H), 7.04 – 6.98 (m, 2H), 4.15 – 4.02 (m, 2H), 1.94 – 1.79 (m, 1H), 1.77 – 1.60 (m, 2H), 1.38 – 1.17 (m, 14H), 0.96 (d, *J* = 6.5 Hz, 3H), 0.89 (t, *J* = 6.9 Hz, 3H).
^{13}C NMR (101 MHz, CDCl$_3$) δ 161.85, 152.93, 146.98, 130.43, 129.16, 124.90, 122.67, 114.85, 66.85, 37.18, 36.23, 32.07, 30.07, 29.96, 29.80, 29.50, 27.08, 22.84, 19.82, 14.28.
HRMS (APCI) calcd for $C_{24}H_{35}ON_2^+$ [$(M+H)^+$] 367.2744, found 367.2741.

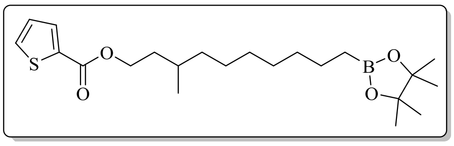

3-methyl-10-(4,4,5,5-tetramethyl-1,3,2-dioxaborolan-2-yl)decyl thiophene-2-carboxylate: Following general procedure B, the product was isolated by flash chromatography (EtOAc: Petroleum ether = 1:20) as colorless oil (50.6 mg, 62%).
^{1}H NMR (400 MHz, CDCl$_3$) δ 7.79 (dd, *J* = 3.7, 1.2 Hz, 1H), 7.54 (dd, *J* = 5.0, 1.2 Hz, 1H), 7.09 (dd, *J* = 4.9, 3.8 Hz, 1H), 4.38 – 4.25 (m, 2H), 1.84 – 1.70 (m, 1H), 1.65 – 1.47 (m, 2H), 1.39 – 1.16 (m, 24H), 0.93 (d, *J* = 6.4 Hz, 3H), 0.76 (t, *J* = 7.7 Hz, 2H).
^{13}C NMR (101 MHz, CDCl$_3$) δ 162.50, 134.27, 133.35, 132.29, 127.82, 82.97, 63.94, 37.06, 35.65, 32.57, 30.08, 29.93, 29.56, 27.03, 24.95, 24.15, 19.74.
^{11}B NMR (128 MHz, CDCl$_3$) δ 34.17.
HRMS (APCI) calcd for $C_{22}H_{38}O_4BS^+$ [$(M+H)^+$] 409.2578, found 409.2581.

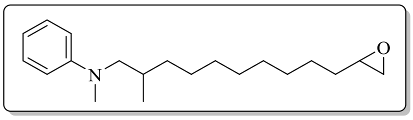

***N*-methyl-*N*-(2-methyl-10-(oxiran-2-yl)decyl)aniline:** Following general procedure A, Cs_2CO_3 was used instead of Na_2CO_3 to avoid the undesired ring-opening products. The product was isolated by flash chromatography (EtOAc: Petroleum ether = 1:20) as colorless oil (38.8 mg, 64%). NMR data for diastereoisomers.

^{1}H NMR (400 MHz, $CDCl_3$) δ 7.25 – 7.18 (m, 2H), 6.73 – 6.62 (m, 3H), 3.26 – 2.98 (m, 2H), 2.95 (s, 3H), 2.94 – 2.88 (m, 1H), 2.79 – 2.72 (m, 1H), 2.47 (dd, *J* = 5.0, 2.7 Hz, 1H), 1.97 – 1.83 (m, 1H), 1.61 – 1.21 (m, 15H), 1.10 (dd, *J* = 17.0, 9.2 Hz, 1H), 0.90 (d, *J* = 6.6 Hz, 3H).

^{13}C NMR (101 MHz, $CDCl_3$) δ 149.85, 129.18, 115.68, 111.92, 60.02, 52.53, 47.26, 39.61, 34.84, 32.63, 32.36, 30.08, 29.66, 29.57, 27.17, 26.10, 17.92.

HRMS (APCI) calcd for $C_{20}H_{34}ON^+$ [$(M+H)^+$] 304.2635, found 304.2630.

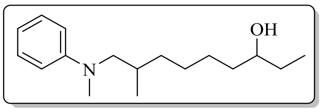

8-methyl-9-(methyl(phenyl)amino)nonan-3-ol: Following general procedure A, the product was isolated by flash chromatography (EtOAc: Petroleum ether = 1:5) as colorless oil (45.8 mg, 87%). NMR data for diastereoisomers.

^{1}H NMR (400 MHz, $CDCl_3$) δ 7.24 – 7.18 (m, 2H), 6.71 – 6.62 (m, 3H), 3.59 – 3.45 (m, 1H), 3.26 – 2.98 (m, 2H), 2.94 (s, 3H), 1.91 (s, 1H), 1.56 – 1.23 (m, 10H), 1.11 (dd, *J* = 17.4, 9.0 Hz, 1H), 0.93 (t, *J* = 7.4 Hz, 3H), 0.89 (d, *J* = 6.6 Hz, 3H).

^{13}C NMR (101 MHz, $CDCl_3$) δ 149.75, 129.18, 115.66, 111.87, 73.38, 73.36, 59.96, 39.64, 37.01, 36.99, 34.75, 34.72, 32.26, 30.28, 30.25, 27.23, 26.08, 17.88, 17.85, 10.02.

HRMS (APCI) calcd for $C_{17}H_{30}ON^+$ [$(M+H)^+$] 264.2322, found 264.2322.

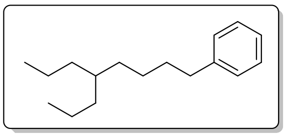

(5-propyloctyl)benzene: Following general procedure B, the product was isolated by flash chromatography (Petroleum ether) as colorless oil (26.4 mg, 57%).

^{1}H NMR (400 MHz, $CDCl_3$) δ 7.32 – 7.22 (m, 2H), 7.22 – 7.11 (m, 3H), 2.71 – 2.51 (m, 2H), 1.68 – 1.48 (m, 2H), 1.40 – 1.11 (m, 13H), 0.87 (t, *J* = 7.0 Hz, 6H).

^{13}C NMR (101 MHz, $CDCl_3$) δ 143.12, 128.53, 128.36, 125.68, 37.03, 36.21, 36.18, 33.64, 32.16, 26.56, 19.94, 14.70.

HRMS (EI) calcd for $C_{17}H_{28}$ (M) 232.2191, found 232.2161.

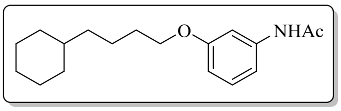

***N*-(3-(4-cyclohexylbutoxy)phenyl)acetamide:** Following general procedure A, the product was isolated by flash chromatography (EtOAc: Petroleum ether = 1:2) as colorless oil (40.5 mg, 70%).

^{1}H NMR (400 MHz, CDCl$_3$) δ 7.33 (brs, 1H), 7.25 (s, 1H), 7.18 (t, *J* = 8.1 Hz, 1H), 6.94 (d, *J* = 7.8 Hz, 1H), 6.64 (d, *J* = 8.0 Hz, 1H), 3.93 (t, *J* = 6.5 Hz, 2H), 2.16 (s, 3H), 1.83 – 1.59 (m, 7H), 1.49 – 1.38 (m, 2H), 1.32 – 1.07 (m, 6H), 0.97 – 0.77 (m, 2H).
^{13}C NMR (101 MHz, CDCl$_3$) δ 168.48, 159.85, 139.16, 129.72, 111.85, 110.78, 106.31, 68.18, 37.72, 37.32, 33.49, 29.65, 26.85, 26.55, 24.84, 23.42.
HRMS (APCI) calcd for $C_{18}H_{28}O_2N^+$ [(M+H)$^+$] 290.2115, found 290.2113.

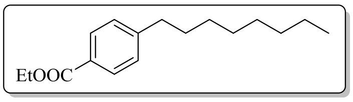

ethyl 4-octylbenzoate: Following general procedure B, the product was isolated by flash chromatography (EtOAc: Petroleum ether = 1:30) as colorless oil (28.8 mg, 55%).
^{1}H NMR (400 MHz, CDCl$_3$) δ 7.95 (d, *J* = 8.3 Hz, 2H), 7.23 (d, *J* = 8.3 Hz, 2H), 4.36 (q, *J* = 7.1 Hz, 2H), 2.65 (t, *J* = 7.9 Hz, 2H), 1.72 – 1.53 (m, 2H), 1.38 (t, *J* = 7.1 Hz, 3H), 1.36 – 1.18 (m, 10H), 0.88 (t, *J* = 6.9 Hz, 3H).
^{13}C NMR (101 MHz, CDCl$_3$) δ 166.88, 148.53, 129.69, 128.51, 128.06, 60.86, 36.13, 31.98, 31.30, 29.55, 29.38, 29.35, 22.78, 14.49, 14.22.
HRMS (APCI) calcd for $C_{17}H_{27}O_2^+$ [(M+H)$^+$] 263.2006, found 263.2005.

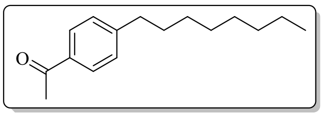

1-(4-octylphenyl)ethan-1-one: Following general procedure B, the product was isolated by flash chromatography (EtOAc: Petroleum ether = 1:20) as colorless oil (24.1 mg, 52%).
^{1}H NMR (400 MHz, CDCl$_3$) δ 7.88 (d, *J* = 8.3 Hz, 2H), 7.26 (d, *J* = 8.3 Hz, 2H), 2.65 (t, *J* = 7.6 Hz, 2H), 2.58 (s, 3H), 1.70 – 1.55 (m, 2H), 1.39 – 1.21 (m, 10H), 0.87 (t, *J* = 6.9 Hz, 3H).
^{13}C NMR (101 MHz, CDCl$_3$) δ 198.06, 149.00, 135.02, 128.74, 128.60, 36.14, 31.99, 31.27, 29.56, 29.40, 29.36, 26.69, 22.79, 14.24.
HRMS (APCI) calcd for $C_{16}H_{25}O^+$ [(M+H)$^+$] 233.1900, found 233.1902.

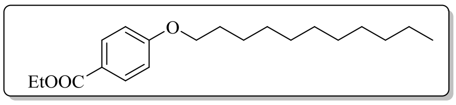

ethyl 4-(undecyloxy)benzoate: Following general procedure B, ethyl 4-(3-(tosyloxy)propoxy)benzoate was used, the product was isolated by flash chromatography (EtOAc: Petroleum ether = 1:15) as colorless oil (28.8 mg, 45%). Following general procedure B, ethyl 4-(3-iodopropoxy)benzoate was used, the product was isolated by flash chromatography (EtOAc: Petroleum ether = 1:15) as colorless oil (39.0 mg, 61%).
^{1}H NMR (400 MHz, CDCl$_3$) δ 7.98 (d, *J* = 8.9 Hz, 2H), 6.90 (d, *J* = 8.9 Hz, 2H), 4.34 (q, *J* = 7.1 Hz, 2H), 4.00 (t, *J* = 6.6 Hz, 2H), 1.85 – 1.74 (m, 2H), 1.51 – 1.22 (m, 19H), 0.88 (t, *J* = 6.8 Hz, 3H).
^{13}C NMR (101 MHz, CDCl$_3$) δ 166.60, 163.01, 131.64, 122.77, 114.13, 68.32, 60.72, 32.05, 29.75, 29.73, 29.69, 29.50, 29.48, 29.25, 26.12, 22.83, 14.53, 14.26.
HRMS (APCI) calcd for $C_{20}H_{33}O_3^+$ [(M+H)$^+$] 321.2424, found 321.2426.

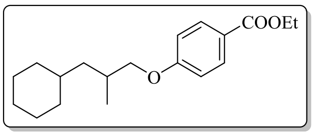

ethyl 4-(3-cyclohexyl-2-methylpropoxy)benzoate: Following general procedure A, the product was isolated by flash chromatography (EtOAc: Petroleum ether = 1:10) as colorless oil (33.4 mg, 55%).

^{1}H NMR (400 MHz, CDCl$_3$) δ 7.98 (d, *J* = 8.5 Hz, 2H), 6.90 (d, *J* = 8.5 Hz, 2H), 4.34 (q, *J* = 7.0 Hz, 2H), 3.84 (dd, *J* = 8.7, 5.5 Hz, 1H), 3.73 (t, *J* = 7.9 Hz, 1H), 2.14 – 2.01 (m, 1H), 1.78 – 1.58 (m, 4H), 1.42 – 1.08 (m, 10H), 1.01 (d, *J* = 6.6 Hz, 3H), 0.98 – 0.78 (m, 2H).

^{13}C NMR (101 MHz, CDCl$_3$) δ 166.62, 163.17, 131.62, 122.72, 114.16, 73.85, 60.73, 41.55, 34.91, 34.29, 33.18, 30.12, 26.80, 26.55, 26.44, 17.48, 14.53.

HRMS (APCI) calcd for $C_{19}H_{29}O_3^+$ [(M+H)$^+$] 305.2111, found 305.2110.

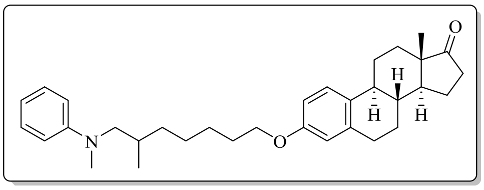

(8*R*,9*S*,13*S*,14*S*)-13-methyl-3-((6-methyl-7-(methyl(phenyl)amino)heptyl)oxy)-6,7,8,9,11,12,13,14,15,16-decahydro-17*H*-cyclopenta[*a*]phenanthren-17-one: Following general procedure A, the product was isolated by flash chromatography (EtOAc: Petroleum ether = 1:8) as white solid (71.2 mg, 73%). NMR data for diastereoisomers.

^{1}H NMR (400 MHz, CDCl$_3$) δ 7.27 – 7.16 (m, 3H), 6.75 – 6.60 (m, 5H), 3.91 (t, *J* = 6.5 Hz, 2H), 3.12 (ddd, *J* = 22.5, 14.5, 7.3 Hz, 2H), 2.94 (s, 3H), 2.92 – 2.84 (m, 2H), 2.50 (dd, *J* = 18.8, 8.5 Hz, 1H), 2.44 – 2.33 (m, 1H), 2.24 (t, *J* = 8.2 Hz, 1H), 2.19 – 1.86 (m, 5H), 1.82 – 1.70 (m, 2H), 1.68 – 1.27 (m, 11H), 1.19 – 1.05 (m, 1H), 0.97 – 0.85 (m, 6H).

^{13}C NMR (101 MHz, CDCl$_3$) δ 220.98, 157.30, 149.87, 137.82, 132.01, 129.21, 126.41, 115.77, 114.72, 112.28, 111.99, 67.99, 60.02, 50.60, 48.16, 44.15, 39.61, 38.56, 36.01, 34.78, 32.35, 31.76, 29.80, 29.46, 26.97, 26.73, 26.57, 26.08, 21.74, 17.92, 14.01.

HRMS (APCI) calcd for $C_{33}H_{46}O_2N^+$ [(M+H)$^+$] 488.3523, found 488.3517.

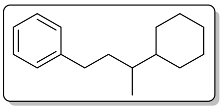

(3-cyclohexylbutyl)benzene: 4 equiv. cyclohexene, 6 equiv. DEMS, 6 equiv. $KHCO_3$, 0.6 mL DMAc were used, 30 ^{o}C, 24 h. $KHCO_3$ was selected for the synthesis of this compound after slight modification based on general procedure. A larger amount of cyclohexene as well as DEMS and $KHCO_3$ would further improve the cross-coupling yield. The product was isolated by flash chromatography (Petroleum ether) as colorless oil (13.0 mg, 30%).

^{1}H NMR (400 MHz, CDCl$_3$) δ 7.30 – 7.26 (m, 2H), 7.25 – 7.14 (m, 3H), 2.77 – 2.42 (m, 2H), 1.83 – 1.51 (m, 6H), 1.52 – 0.94 (m, 8H), 0.89 (d, *J* = 6.6 Hz, 3H).

^{13}C NMR (101 MHz, CDCl$_3$) δ 143.45, 128.49, 128.39, 125.65, 42.90, 37.92, 36.32, 34.10, 30.76, 28.83, 27.10, 27.02, 26.98, 16.21.

HRMS (EI) calcd for $C_{16}H_{24}$ (M) 216.1878, found 216.1846.

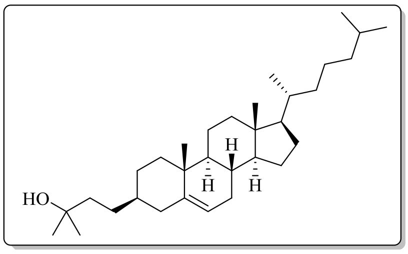

4-((3*R*,8*S*,9*S*,10*R*,13*R*,14*S*,17*R*)-10,13-dimethyl-17-((*R*)-6-methylheptan-2-yl)-2,3,4,7,8,9,10,11,12,13,14,15,16,17-tetradecahydro-1*H*-cyclopenta[*a*]phenanthren-3-yl)-2-methylbutan-2-ol: 0.2 mmol Cholesterol derivative, 0.5 mmol 2-Methyl-3-buten-2-ol, 10 mol% $NiBr_2$·diglyme, 15 mol% 4,4'-di-*tert*-butyl-2,2'-bipyridine, 3.0 equiv. DEMS, 3.0 equiv. Na_2CO_3, 2 mL DMAc were used, the product was isolated by flash chromatography (EtOAc: Petroleum ether = 1:7) as white solid (50.3 mg, 55%). The stereochemistry at carbon-carbon forming position was consistent with previously reported analogs. [3]

^{1}H NMR (400 MHz, $CDCl_3$) δ 5.30 – 5.25 (m, 1H), 2.09 – 1.76 (m, 6H), 1.62 – 1.23 (m, 18H), 1.20 (s, 6H), 1.19 – 0.99 (m, 10H), 0.97 (s, 3H), 0.91 (d, *J* = 6.5 Hz, 3H), 0.87 (d, *J* = 1.8 Hz, 3H), 0.85 (d, *J* = 1.8 Hz, 3H), 0.67 (s, 3H).

^{13}C NMR (101 MHz, $CDCl_3$) δ 143.51, 119.43, 71.21, 56.99, 56.31, 50.62, 42.45, 41.29, 40.03, 39.99, 39.86, 39.74, 39.67, 37.41, 36.34, 35.95, 32.05, 31.87, 29.38, 29.36, 29.32, 28.40, 28.16, 24.44, 23.98, 22.98, 22.71, 21.08, 19.65, 18.87, 12.01.

HRMS (APCI) calcd for $C_{32}H_{57}O^+$ $[(M+H)^+]$ 457.4404, found 457.4409.

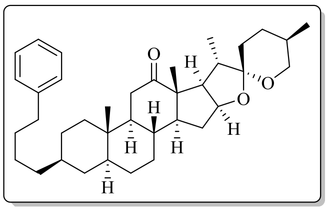

(2a*S*,4*S*,5'*R*,6a*S*,6b*S*,8a*S*,8b*R*,9*S*,10*R*,11a*S*,12a*S*,12b*R*)-5',6a,8a,9-tetramethyl-4-(4-phenylbutyl)icosahydrospiro[naphtho[2',1':4,5]indeno[2,1-*b*]furan-10,2'-pyran]-8(2*H*)-one: 0.2 mmol Hecogenin derivative, 0.5 mmol 4-Phenyl-1-butene, 10 mol% $NiBr_2$·diglyme, 15 mol% 4,4'-di-*tert*-butyl-2,2'-bipyridine, 3.0 equiv. DEMS, 3.0 equiv. Na_2CO_3, 2 mL DMAc were used, the product was isolated by flash chromatography (EtOAc: Petroleum ether = 1:20) as white solid (73.1 mg, 67%). The stereochemistry at carbon-carbon forming position was determined by single-crystal X-ray diffraction analysis.

^{1}H NMR (400 MHz, $CDCl_3$) δ 7.30 – 7.23 (m, 2H), 7.21 – 7.12 (m, 3H), 4.39 – 4.30 (m, 1H), 3.54 – 3.44 (m, 1H), 3.35 (t, *J* = 10.9 Hz, 1H), 2.63 – 2.56 (m, 2H), 2.52 (dd, *J* = 8.7, 6.7 Hz, 1H), 2.42 – 2.32 (m, 1H), 2.23 (dd, *J* = 14.4, 5.0 Hz, 1H), 2.15 – 2.08 (m, 1H), 1.96 – 1.83 (m, 1H), 1.80 – 1.39 (m, 13H), 1.37 – 1.17 (m, 8H), 1.17 – 0.99 (m, 9H), 0.98 – 0.86 (m, 3H), 0.83 (s, 3H), 0.79 (d, *J* = 6.4 Hz, 3H).

^{13}C NMR (101 MHz, $CDCl_3$) δ 214.08, 142.98, 128.49, 128.34, 125.68, 109.36, 79.38, 67.00, 56.07, 56.04, 55.23, 53.65, 46.63, 42.32, 38.28, 37.92, 37.82, 37.29, 36.89, 36.10, 35.66, 34.53, 31.91, 31.81, 31.56, 31.27, 30.33, 28.92, 28.82, 28.74, 26.71, 17.26, 16.15, 13.40, 12.08.

HRMS (APCI) calcd for $C_{37}H_{55}O_3^+$ $[(M+H)^+]$ 547.4146, found 547.4153.

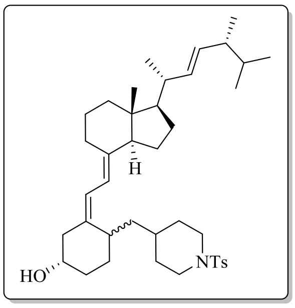

(1*S*,*Z*)-3-(2-((1*R*,3a*S*,7a*R*,*E*)-1-((2*R*,5*R*,*E*)-5,6-dimethylhept-3-en-2-yl)-7a-methyloctahydro-4*H*-inden-4-ylidene)ethylidene)-4-((1-tosylpiperidin-4-yl)methyl)cyclohexan-1-ol: 0.2 mmol 4-iodo-1-tosylpiperidine, 0.5 mmol Calciferol, 20 mol% $NiBr_2$·diglyme, 30 mol% 4,4'-di-*tert*-butyl-2,2'-bipyridine, 3.0 equiv. DEMS, 3.0 equiv. Na_2CO_3, 2 mL DMAc were used, the product was isolated by flash chromatography. Diastereoisomers were obtained. The less polar product (EtOAc: Petroleum ether = 1:3) as colorless oil (23.0 mg, 18%). The more polar product (EtOAc: Petroleum ether = 1:2.5) as colorless oil (19.4 mg, 15%).

The less polar product:

^{1}H NMR (400 MHz, $CDCl_3$) δ 7.60 (d, *J* = 8.2 Hz, 2H), 7.30 (d, *J* = 8.0 Hz, 2H), 6.14 (d, *J* = 11.3 Hz, 1H), 5.78 (d, *J* = 11.4 Hz, 1H), 5.30 (dd, *J* = 15.3, 7.7 Hz, 1H), 5.18 (dd, *J* = 15.3, 8.4 Hz, 1H), 4.02 (s, 1H), 3.73 (d, *J* = 11.4 Hz, 2H), 3.09 – 2.91 (m, 1H), 2.83 – 2.70 (m, 1H), 2.51 (d, *J* = 14.0 Hz, 1H), 2.42 (s, 3H), 2.15 (td, *J* = 11.9, 2.5 Hz, 1H), 2.08 – 1.94 (m, 4H), 1.90 – 1.71 (m, 4H), 1.70 – 1.57 (m, 6H), 1.54 – 1.11 (m, 13H), 1.09 – 0.80 (m, 13H), 0.37 (s, 3H).

^{13}C NMR (101 MHz, $CDCl_3$) δ 143.45, 142.48, 136.26, 135.57, 133.04, 132.38, 129.63, 127.88, 123.35, 115.28, 68.00, 56.46, 56.42, 46.73, 45.68, 43.00, 40.76, 40.47, 40.33, 37.75, 33.23, 32.70, 32.57, 31.62, 28.86, 28.10, 27.97, 26.35, 23.60, 22.49, 21.66, 21.27, 20.14, 19.82, 17.80, 12.25.

HRMS (APCI) calcd for $C_{40}H_{62}O_3NS^+$ [(M+H)$^+$] 636.4445, found 636.4448.

The more polar product:

^{1}H NMR (400 MHz, $CDCl_3$) δ 7.62 (d, *J* = 8.2 Hz, 2H), 7.30 (d, *J* = 8.0 Hz, 2H), 6.07 (d, *J* = 11.0 Hz, 1H), 5.67 (d, *J* = 11.1 Hz, 1H), 5.29 – 5.12 (m, 2H), 3.71 (d, *J* = 11.2 Hz, 2H), 3.63 – 3.51 (m, 1H), 2.88 – 2.78 (m, 1H), 2.72 (d, *J* = 10.2 Hz, 1H), 2.43 (s, 3H), 2.30 (dd, *J* = 12.7, 3.3 Hz, 1H), 2.23 – 1.85 (m, 7H), 1.83 – 1.73 (m, 2H), 1.66 – 1.42 (m, 12H), 1.37 – 1.21 (m, 8H), 1.02 (d, *J* = 6.6 Hz, 3H), 0.94 (d, *J* = 6.8 Hz, 3H), 0.85 (t, *J* = 6.6 Hz, 6H), 0.54 (s, 3H).

^{13}C NMR (101 MHz, $CDCl_3$) δ 143.43, 142.02, 138.47, 135.64, 133.32, 132.24, 129.65, 127.88, 121.26, 115.61, 71.85, 56.64, 56.49, 46.59, 46.52, 45.73, 42.98, 42.81, 40.56, 38.28, 33.25, 32.79, 32.19, 31.87, 31.50, 30.69, 28.93, 28.82, 27.95, 23.57, 22.36, 21.68, 21.25, 20.14, 19.82, 17.78, 12.43.

HRMS (APCI) calcd for $C_{40}H_{62}O_3NS^+$ [(M+H)$^+$] 636.4445, found 636.4448.

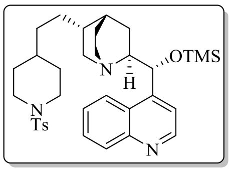

(1*S*,2*S*,4*S*,5*R*)-2-((*R*)-quinolin-4-yl((trimethylsilyl)oxy)methyl)-5-(2-(1-tosylpiperidin-4-yl)ethyl)quinuclidine: 0.2 mmol 4-iodo-1-tosylpiperidine, 0.3 mmol Cinchonidine-OTMS, 20 mol% $NiBr_2$·diglyme, 30 mol% 4,4'-di-*tert*-butyl-2,2'-bipyridine, 2.0 equiv. DEMS, 2.0 equiv. Na_2CO_3, 2 mL

THF/DMAc (v:v = 1:3) were used, the product was isolated by flash chromatography (first time: THF: Petroleum ether = 1:1; then: CH_2Cl_2: MeOH = 20:1) as white solid (57.0 mg, 47%).

^{1}H NMR (400 MHz, $CDCl_3$) δ 8.91 (d, *J* = 2.9 Hz, 1H), 8.70 (d, *J* = 7.5 Hz, 1H), 8.15 (d, *J* = 6.9 Hz, 1H), 7.85 – 7.69 (m, 2H), 7.57 (d, *J* = 8.1 Hz, 2H), 7.52 (d, *J* = 4.3 Hz, 1H), 7.28 (d, *J* = 6.8 Hz, 2H), 6.69 (s, 1H), 3.96 (s, 1H), 3.68 (d, *J* = 10.9 Hz, 2H), 3.44 (t, *J* = 11.7 Hz, 1H), 3.25 (t, *J* = 8.6 Hz, 1H), 3.12 (brs, 1H), 2.73 (d, *J* = 10.8 Hz, 1H), 2.40 (s, 3H), 2.28 – 2.17 (m, 1H), 2.15 – 1.95 (m, 4H), 1.82 (d, *J* = 32.4 Hz, 2H), 1.57 (d, *J* = 12.2 Hz, 2H), 1.44 – 1.03 (m, 8H), 0.14 (s, 9H).

^{13}C NMR (101 MHz, $CDCl_3$) δ 149.66, 148.40, 145.92, 143.50, 133.03, 130.21, 129.94, 129.63, 128.37, 127.73, 124.73, 123.78, 118.72, 68.44, 60.68, 56.88, 46.33, 46.30, 43.09, 35.10, 33.96, 33.46, 31.50, 31.31, 25.21, 25.11, 21.58, 18.19, 0.40.

HRMS (ESI) calcd for $C_{34}H_{48}O_3N_3SSi^+$ [$(M+H)^+$] 606.3180, found 606.3175.

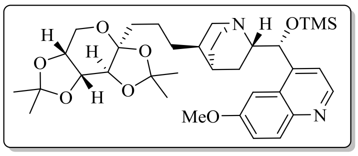

(1*S*,2*S*,4*S*,5*R*)-2-((*R*)-(6-methoxyquinolin-4-yl)((trimethylsilyl)oxy)methyl)-5-(3-((3a*S*,5a*R*,8a*R*,8b*S*)-2,2,7,7-tetramethyltetrahydro-3a*H*-bis([1,3]dioxolo)[4,5-*b*:4',5'-*d*]pyran-3a-yl)propyl)quinuclidine: 0.2 mmol Quinine-OTMS, 0.4 mmol Fructose derivative, 20 mol% $NiBr_2$·diglyme, 30 mol% 4,4'-di-*tert*-butyl-2,2'-bipyridine, 3.0 equiv. DEMS, 3.0 equiv. Na_2CO_3, 2 mL DMAc were used, the product was isolated by flash chromatography (first time: THF: Petroleum ether = 1:1; then: CH_2Cl_2: MeOH = 20:1) as colorless oil (43.5 mg, 34%).

^{1}H NMR (400 MHz, $CDCl_3$) δ 8.73 (d, *J* = 4.5 Hz, 1H), 8.01 (d, *J* = 9.2 Hz, 1H), 7.75 (s, 1H), 7.48 (d, *J* = 4.6 Hz, 1H), 7.40 (dd, *J* = 9.2, 2.5 Hz, 1H), 6.75 (s, 1H), 4.48 (dd, *J* = 8.0, 2.4 Hz, 1H), 4.19 – 4.12 (m, 4H), 4.01 (t, *J* = 13.1 Hz, 1H), 3.95 (d, *J* = 2.4 Hz, 1H), 3.75 (dd, *J* = 13.1, 1.7 Hz, 1H), 3.59 (d, *J* = 13.0 Hz, 1H), 3.40 (dd, *J* = 13.1, 10.7 Hz, 1H), 3.25 (t, *J* = 9.0 Hz, 1H), 3.14 – 3.02 (m, 1H), 2.79 – 2.69 (m, 1H), 2.29 – 2.18 (m, 1H), 2.06 (brs, 2H), 1.96 (brs, 1H), 1.83 – 1.65 (m, 2H), 1.60 – 1.50 (m, 2H), 1.47 – 1.41 (m, 4H), 1.34 (s, 3H), 1.30 – 1.22 (m, 9H), 0.15 (s, 9H).

^{13}C NMR (101 MHz, $CDCl_3$) δ 159.53, 146.78, 144.69, 144.13, 131.81, 126.00, 123.54, 119.12, 108.86, 107.61, 103.71, 100.96, 74.00, 70.82, 70.59, 68.15, 60.94, 60.41, 58.15, 56.95, 43.17, 40.69, 34.40, 34.03, 26.44, 25.94, 25.20, 25.08, 24.91, 24.05, 20.82, 18.26, 0.48.

HRMS (APCI) calcd for $C_{35}H_{53}O_7N_2Si^+$ [$(M+H)^+$] 641.3617, found 641.3616.

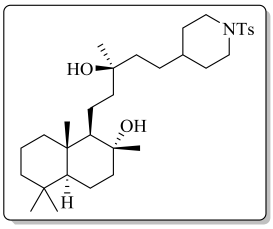

(1*R*,2*R*,4a*S*,8a*S*)-1-((*S*)-3-hydroxy-3-methyl-5-(1-tosylpiperidin-4-yl)pentyl)-2,5,5,8a-tetramethyldecahydronaphthalen-2-ol: 0.2 mmol Sclareol, 0.4 mmol 4-iodo-1-tosylpiperidine, 20 mol% $NiBr_2$·diglyme, 30 mol% 4,4'-di-*tert*-butyl-2,2'-bipyridine, 3.0 equiv. DEMS, 3.0 equiv. Na_2CO_3, 2 mL DMAc were used, the product was isolated by flash chromatography (EtOAc: Petroleum ether = 1:1) as colorless oil (39.3mg, 36%).

^{1}H NMR (400 MHz, $CDCl_3$) δ 7.62 (d, *J* = 8.2 Hz, 2H), 7.30 (d, *J* = 8.0 Hz, 2H), 3.74 (d, *J* = 11.5 Hz, 2H), 2.42 (s, 3H), 2.35 – 2.09 (m, 4H), 1.87 – 1.77 (m, 1H), 1.70 (d, *J* = 11.4 Hz, 2H), 1.64 – 1.19 (m, 18H), 1.15 – 1.05 (m, 8H), 0.98 – 0.86 (m, 2H), 0.84 (s, 3H), 0.76 (s, 3H), 0.76 (s, 3H).
^{13}C NMR (101 MHz, $CDCl_3$) δ 143.44, 133.20, 129.66, 127.84, 74.90, 73.01, 61.94, 56.14, 46.63, 44.54, 44.36, 42.07, 40.36, 39.74, 39.30, 35.89, 33.49, 33.32, 31.75, 31.71, 30.29, 26.09, 24.41, 21.64, 21.58, 20.59, 18.91, 18.51, 15.50.
HRMS (ESI) calcd for $C_{32}H_{53}O_4NNaS^+$ [$(M+Na)^+$] 570.3588, found 570.3587.

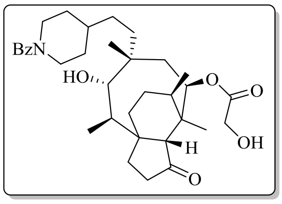

(3a*R*,5*R*,7*R*,8*S*,9*R*,12*R*)-7-(2-(1-benzoylpiperidin-4-yl)ethyl)-8-hydroxy-4,7,9,12-tetramethyl-3-oxodecahydro-4,9a-propanocyclopenta[8]annulen-5-yl 2-hydroxyacetate: 0.2 mmol Pleuromulin, 0.6 mmol (4-iodopiperidin-1-yl)(phenyl)methanone, 20 mol% $NiBr_2$·diglyme, 30 mol% 4,4'-di-*tert*-butyl-2,2'-bipyridine, 4.0 equiv. DEMS, 4.0 equiv. Na_2CO_3, 2 mL DMAc were used, the product was isolated by flash chromatography (first time: THF: Petroleum ether = 1:1; then: CH_2Cl_2: MeOH = 20:1) as colorless oil (31.8 mg, 28%).
^{1}H NMR (400 MHz, $CDCl_3$) δ 7.46 – 7.32 (m, 5H), 5.71 (d, *J* = 8.1 Hz, 1H), 4.68 (s, 1H), 4.12 – 3.90 (m, 2H), 3.72 (s, 1H), 3.39 (d, *J* = 6.0 Hz, 1H), 2.87 (d, *J* = 76.8 Hz, 2H), 2.39 (p, *J* = 6.7 Hz, 1H), 2.29 – 2.12 (m, 2H), 2.08 (s, 1H), 1.88 (s, 1H), 1.83 – 1.70 (m, 3H), 1.69 – 1.42 (m, 8H), 1.39 (s, 3H), 1.36 – 1.23 (m, 3H), 1.19 – 1.01 (m, 5H), 0.95 (d, *J* = 8.0 Hz, 6H), 0.66 (d, *J* = 6.9 Hz, 3H).
^{13}C NMR (101 MHz, $CDCl_3$) δ 216.90, 172.54, 170.41, 136.67, 129.49, 128.50, 126.96, 76.48, 70.26, 61.45, 58.58, 45.66, 42.05, 41.82, 40.82, 37.14, 36.66, 34.62, 34.54, 30.56, 30.34, 27.12, 27.01, 25.98, 25.08, 16.49, 14.87, 11.27.
^{13}C NMR (101 MHz, $CDCl_3$, DEPT-90) δ 129.49, 128.49, 126.94, 76.46, 70.23, 58.56, 37.12, 36.64, 34.59.
^{13}C NMR (101 MHz, $CDCl_3$, DEPT-135) δ 129.49, 128.49, 126.95, 76.46, 70.24, 61.43, 58.56, 41.77, 37.12, 36.64, 34.59, 34.53, 30.54, 30.32, 27.12, 26.99, 25.94, 25.06, 16.49, 14.86, 11.28.
HRMS (APCI) calcd for $C_{34}H_{50}O_6N^+$ [$(M+H)^+$] 568.3633, found 568.3631.

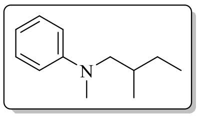

***N*-methyl-*N*-(2-methylbutyl)aniline:** Balloon of ethylene was used instead of argon atmosphere, 10 mol% $NiBr_2$·diglyme, 15 mol% 4,4'-di-*tert*-butyl-2,2'-bipyridine, 6.0 equiv. DEMS, 6.0 equiv. Na_2CO_3 were used, the product was isolated by flash chromatography (Petroleum ether) as colorless oil (21.9 mg, 62%).
^{1}H NMR (400 MHz, $CDCl_3$) δ 7.23 (t, *J* = 7.9 Hz, 2H), 6.75 – 6.62 (m, 3H), 3.14 (ddd, *J* = 22.4, 14.6, 7.3 Hz, 2H), 2.96 (s, 3H), 1.94 – 1.76 (m, 1H), 1.53 – 1.41 (m, 1H), 1.21 – 1.05 (m, 1H), 1.00 – 0.85 (m, 6H).
^{13}C NMR (101 MHz, $CDCl_3$) δ 149.83, 129.20, 115.66, 111.89, 59.69, 39.59, 33.99, 27.43, 17.37, 11.61.

HRMS (ESI) calcd for $C_{12}H_{20}N^+$ $[(M+H)^+]$ 178.1590, found 178.1587.

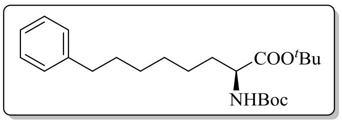

***tert*-butyl (*S*)-2-((*tert*-butoxycarbonyl)amino)-8-phenyloctanoate:** Following general procedure B, the product was isolated by flash chromatography (EtOAc: Petroleum ether = 1:20) as colorless oil (43.8 mg, 56%).

^{1}H NMR (400 MHz, $CDCl_3$) δ 7.30 – 7.24 (m, 2H), 7.21 – 7.13 (m, 3H), 4.99 (d, *J* = 8.1 Hz, 1H), 4.16 (dd, *J* = 13.2, 7.4 Hz, 1H), 2.67 – 2.55 (m, 2H), 1.81 – 1.69 (m, 1H), 1.67 – 1.55 (m, 3H), 1.45 (s, 9H), 1.44 (s, 9H), 1.38 – 1.25 (m, 6H).

^{13}C NMR (101 MHz, $CDCl_3$) δ 172.29, 155.51, 142.88, 128.52, 128.37, 125.73, 81.75, 79.67, 54.07, 36.03, 33.05, 31.49, 29.25, 29.21, 28.48, 28.16, 25.16.

HRMS (ESI) calcd for $C_{23}H_{38}O_4N^+$ $[(M+H)^+]$ 392.2795, found 392.2795.

HPLC analysis: The *ee* value was determined to be 99% by HPLC analysis on a Chiralpak OD-H column, λ = 214 nm, *n*-hexane/*i*-PrOH (98:2), flow rate = 0.5 mL/min; t_R = 10.2 min (minor), 12.4 min (major).

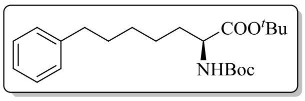

***tert*-butyl (*S*)-2-((*tert*-butoxycarbonyl)amino)-7-phenylheptanoate:** Following general procedure B, the product was isolated by flash chromatography (EtOAc: Petroleum ether = 1:20) as colorless oil (26.4 mg, 35%).

^{1}H NMR (400 MHz, $CDCl_3$) δ 7.33 – 7.22 (m, 2H), 7.22 – 7.12 (m, 3H), 5.00 (d, *J* = 7.8 Hz, 1H), 4.16 (d, *J* = 5.7 Hz, 1H), 2.65 – 2.54 (m, 2H), 1.81 – 1.68 (m, 1H), 1.67 – 1.55 (m, 3H), 1.46 (s, 9H), 1.44 (s, 9H), 1.41 – 1.28 (m, 4H).

^{13}C NMR (101 MHz, $CDCl_3$) δ 172.25, 155.51, 142.73, 128.51, 128.39, 125.78, 81.78, 79.67, 54.06, 35.93, 33.04, 31.40, 29.05, 28.48, 28.15, 25.12.

HRMS (APCI) calcd for $C_{22}H_{36}O_4N^+$ $[(M+H)^+]$ 378.2639, found 378.2637.

HPLC analysis: The *ee* value was determined to be 99% by HPLC analysis on a Chiralpak OD-H column, λ = 214 nm, *n*-hexane/*i*-PrOH (98:2), flow rate = 0.5 mL/min; t_R = 9.4 min (minor), 10.6 min (major).

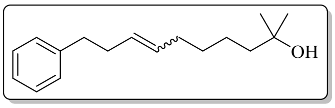

2-methyl-10-phenyldec-7-en-2-ol: Following general procedure B, the product was isolated by flash chromatography (EtOAc: Petroleum ether = 1:7) as colorless oil (16.7 mg, 34%).

^{1}H NMR (400 MHz, $CDCl_3$) δ 7.31 – 7.25 (m, 2H), 7.22 – 7.14 (m, 3H), 5.63 – 5.31 (m, 2H), 2.74 – 2.55 (m, 2H), 2.43 – 2.23 (m, 2H), 2.12 – 1.90 (m, 2H), 1.76 – 1.56 (m, 1H), 1.49 – 1.40 (m, 2H), 1.38 – 1.28 (m, 4H), 1.20 (s, 6H).

^{13}C NMR (101 MHz, $CDCl_3$) δ 142.28, 131.00, 129.66, 128.59, 128.35, 125.82, 71.18, 43.92, 36.26, 34.56, 32.65, 30.16, 29.35, 23.94.

HRMS (APCI) calcd for $C_{17}H_{27}O^+$ $[(M+H)^+]$ 247.2056, found 247.2060.

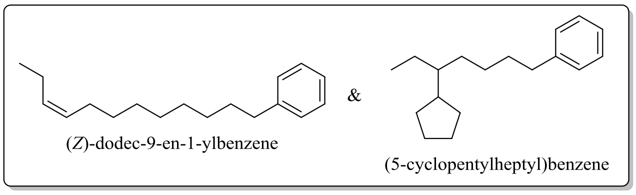

(*Z*)-dodec-9-en-1-ylbenzene & (5-cyclopentylheptyl)benzene: Following general procedure B, the product was isolated by flash chromatography (Petroleum ether) as colorless oil (19.5 mg, 40%). The ratio of (*Z*)-dodec-9-en-1-ylbenzene/(5-cyclopentylheptyl)benzene (~3:1) was determined by ^{1}H NMR and GC-MS. ^{1}H NMR and ^{13}C NMR for the inseparable mixture were carried out. For (*Z*)-dodec-9-en-1-ylbenzene and (5-cyclopentylheptyl)benzene, [M] = 244 ($C_{18}H_{28}$) were detected by GC-MS analysis. The starting material (*Z*)-8-Iodooct-3-ene was *Z/E* mixture (ratio ~ 9:1).

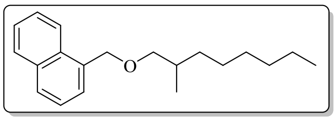

1-(((2-methyloctyl)oxy)methyl)naphthalene: Following general procedure B, (*S*)-1-((2-bromopropoxy)methyl)naphthalene was used, the product was isolated by flash chromatography (EtOAc: Petroleum ether = 1:100) as colorless oil (21.0 mg, 37%).

^{1}H NMR (400 MHz, CDCl$_3$) δ 8.16 – 8.08 (m, 1H), 7.89 – 7.83 (m, 1H), 7.80 (d, *J* = 8.2 Hz, 1H), 7.56 – 7.46 (m, 3H), 7.46 – 7.40 (m, 1H), 5.01 – 4.88 (m, 2H), 3.36 (ddd, *J* = 38.5, 9.0, 6.4 Hz, 2H), 1.77 (td, *J* = 13.0, 6.6 Hz, 1H), 1.48 – 1.36 (m, 1H), 1.32 – 1.22 (m, 8H), 1.15 – 1.05 (m, 1H), 0.92 (d, *J* = 6.7 Hz, 3H), 0.87 (t, *J* = 6.9 Hz, 3H).

^{13}C NMR (101 MHz, CDCl$_3$) δ 134.42, 133.90, 131.94, 128.61, 128.53, 126.31, 126.14, 125.83, 125.34, 124.33, 76.29, 71.74, 33.84, 33.64, 32.02, 29.75, 27.02, 22.82, 17.37, 14.25.

HRMS (APCI) calcd for $C_{20}H_{29}O^+$ $[(M+H)^+]$ 285.2213, found 285.2216.

HPLC analysis: The *ee* value was determined to be 0% by HPLC analysis on a Chiralpak OD-H column, λ = 214 nm, *n*-hexane/*i*-PrOH (99.5:0.5), flow rate = 1.0 mL/min; t_R = 6.0 min, 6.3 min.

Supplementary References

[1] Yang, C.-T. *et al.* Copper-catalyzed cross-coupling of nonactivated secondary alkyl halides and tosylates with secondary alkyl Grignard reagents. *J. Am. Chem. Soc.* **134**, 11124-11127 (2012).

[2] Xu, J. *et al.* Copper-catalyzed trifluoromethylation of terminal alkenes through allylic C–H bond activation. *J. Am. Chem. Soc.* **133**, 15300-15303 (2011).

[3] Lu, Z. & Fu, G. C. Alkyl-alkyl Suzuki cross-coupling of unactivated secondary alkyl chlorides. *Angew. Chem. Int. Ed.* **49**, 6676-6678 (2010).
